# Molecular Structure, Antioxidant Potential, and Pharmacokinetic Properties of Plant Flavonoid Blumeatin and Investigating Its Inhibition Mechanism on Xanthine Oxidase for Hyperuricemia by Molecular Modeling

**DOI:** 10.1021/acsomega.3c10083

**Published:** 2024-03-11

**Authors:** Cisem Altunayar-Unsalan, Ozan Unsalan

**Affiliations:** † Graduate School of Natural and Applied Sciences, Ege University, 35100 Bornova, Izmir, Turkey; ‡ Central Research Testing and Analysis Laboratory Research and Application Center, Ege University, 35100 Bornova, Izmir, Turkey; § Department of Physics, Faculty of Science, Ege University, 35100 Bornova, Izmir, Turkey

## Abstract

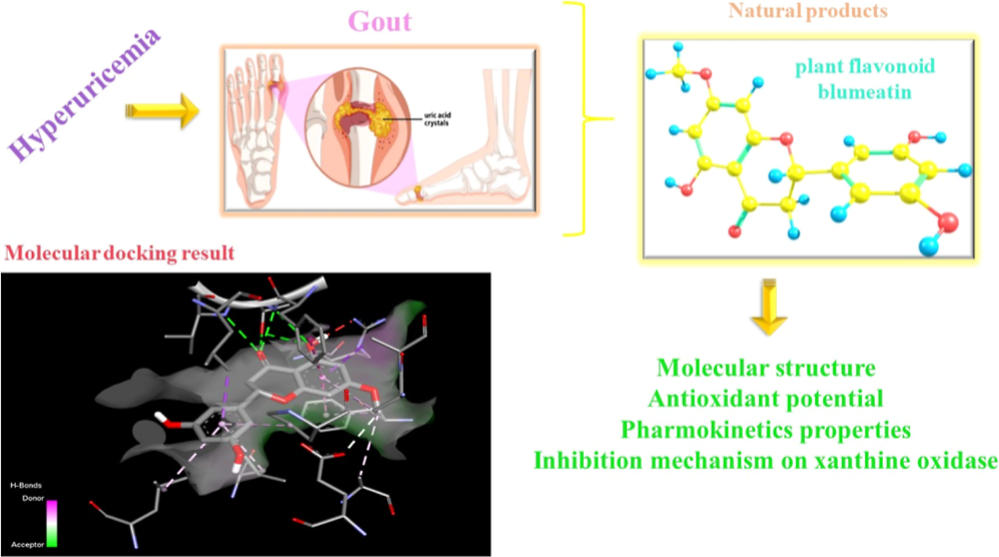

Hyperuricemia, which usually results in metabolic syndrome symptoms, is increasing rapidly all over the world and becoming a global public health issue. Xanthine oxidase (XO) is regarded as a key drug target for the treatment of this disease. Therefore, finding natural, nontoxic, and highly active XO inhibitors is quite important. To get insights into inhibitory potential toward XO and determine antioxidant action mechanism depending on the molecular structure, plant flavonoid blumeatin was investigated for the first time by Fourier transform infrared (FTIR) spectroscopy, density functional theory (DFT), ADME/Tox (absorption, distribution, metabolism, excretion, and toxicity) analysis, and molecular docking study. Theoretical findings indicated that blumeatin has high radical scavenging activity due to its noncoplanarity and over twisted torsion angle (−94.64°) with respect to its flavanone skeleton could explain that there might be a correlation between antioxidant activity and planarity of blumeatin. Based on the ADME/Tox analysis, it is determined that blumeatin has a high absorption profile in the human intestine (81.93%), and this plant flavonoid is not carcinogenic or mutagenic. A molecular docking study showed that Thr1010, Val1011, Phe914, and Ala1078 are the main amino acid residues participating in XO’s interaction with blumeatin via hydrogen bonds.

## 1 Introduction

Hyperuricemia (HUA) is the fourth most prevalent metabolic disease following hyperglycemia, hyperlipidemia, and hypertension.^1^ It is described as a serum urate level that surpasses the usual range of 2.5–7.0 mg/dL in men and 1.5–6.0 mg/dL in women.^2^ HUA arises from hepatic overproduction and/or renal underexcretion of uric acid (UA), which leads to UA deposition at the kidneys and joints, appearing mainly as kidney stone-related diseases and gout, respectively.^3^ HUA is not only the molecular base for gout, but it is also linked to the onset and progression of diabetes, hypertension, coronary heart disease, and other illnesses.^4^

One of the most significant HUA treatment approaches is to successfully inhibit UA production. Xanthine oxidase (XO) is an essential target for the therapy of HUA.^1^ XO is a multifunctional molybdoflavoprotein enzyme and found in many mammalian tissues, particularly the intestine tract and liver.^5^ It catalyzes the oxidation of hypoxanthine and xanthine to generate UA and reactive oxygen species (ROS) such as superoxide anion radical and hydrogen peroxide, which plays an essential role in HUA development.^6^ Allopurinol, an efficient XO inhibitor, is still the primary drug for HUA, but it has a number of adverse effects, including Stevens-Johnson syndrome, hypersensitivity syndrome, renal toxicity, vasculitis, and ROS-induced diseases.^7^ As a result, the search for new XO inhibitors with fewer adverse effects and greater therapeutic action as alternatives to current drugs for the avoidance and therapy of HUA is critical.^5^ Naturally derived XO inhibitors have emerged as one of the research areas for drugs owing to the high safety of naturally derived substances.^8^

**Figure 1:**
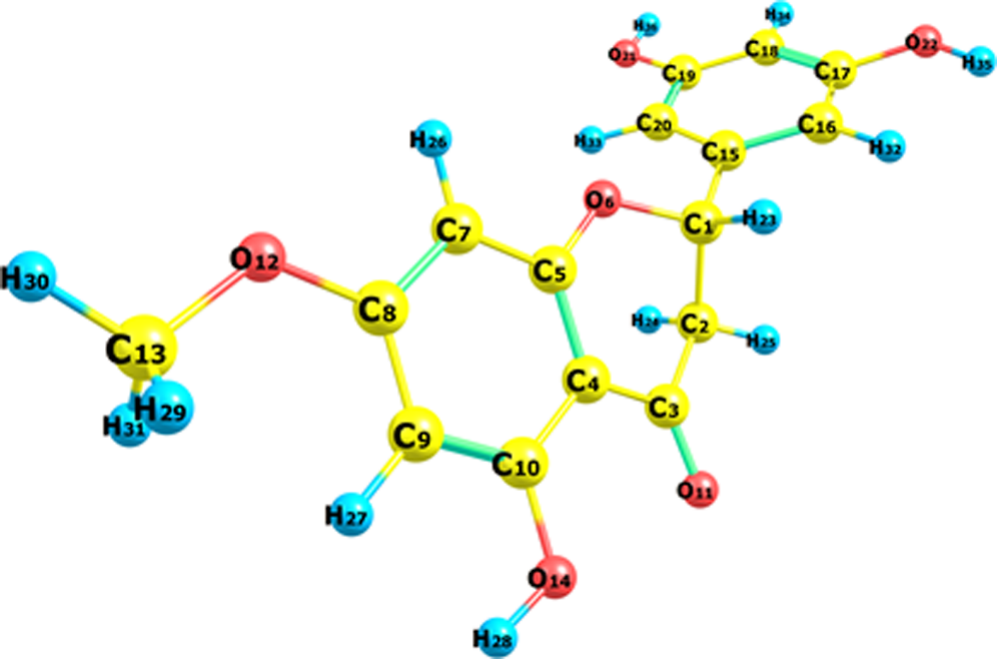
Blumeatin and its corresponding optimized geometric structure.

Flavonoids are an extensive group of polyphenolic natural products found in a variety of plant-based foods and beverages.^9^ They possess a numerous range of biological activities such as anticancer,^10,11^ antiviral,^12−15^ antifungal,^16^ antibacterial,^17^ and antioxidant activities.^18−21^ Their bioactivity arises from structural characteristics which provide them powerful ability to inhibit enzymes, decrease oxidized chemical entities, scavenge free radicals, and bring metal chelation.^22^ However, finding both robust experimental and theoretical links/correlations between the structure and activities of flavonoids is still a challenging ongoing process. In our previous studies, we focused on the experimental and theoretical vibrational spectral results of the molecular structures of some flavonoid derivatives.^23−32^ While some flavonoids show activity with the presence of both a double bond (C_2_–C_3_) and a catechol moiety, and additionally C_3_ hydroxyl, some others lacking the C_3_–OH group still show activity.^33^ From this point of view, a highly systematic theoretical and experimental effort is crucial to further explain how such structural parameters affect the antioxidant and radical scavenging activities of these natural plant-based compounds. While the presence and number of hydroxyl groups at certain positions were considered in some previous studies,^34,35^ the interaction between the groups was not taken into account. Thus, this study targets one step further in the evaluation of these correlations by investigating a very rarely studied flavonoid family member, blumeatin. Blumeatin is a naturally occurring flavonoid found in the sembung plant (*Blumea balsamifera* L.).^36^ It has hydroxyl groups at positions C-3′ and 5′ and lacks a C_2_–C_3_ double bond (Figure 1). Compared to flavanol tamarixetin, blumeatin has a higher level of antioxidant activity.^37^ Blumeatin also possesses a number of biological properties, including superoxide radical scavenging, hepatoprotective, antioxidant, and antityrosinase activities.^36,38,39^ However, no detailed studies have been reported to elucidate the molecular mechanism of action of blumeatin so far. To clarify the relationship between the structure and molecular activity of this compound, first, attenuated total reflection-Fourier transform infrared (ATR-FTIR) spectroscopy and density functional theory (DFT) calculations were carried out. Thus, blumeatin’s quantum chemical descriptors such as chemical potential (μ = −χ), global chemical hardness (η), electrophilicity index (ω), electronegativity (χ), global softness (s), and nucleophilicity (ε) were found. Second, ADME/Tox (absorption, distribution, metabolism, excretion, and toxicity) analysis on this flavonoid was performed for the earlier prediction of pharmacokinetic properties, and it was evaluated in terms of selected molecular descriptors including drug-likeness, tumorigenicity, mutagenicity, and toxicity. Then, molecular docking studies against XO were conducted. The interaction of blumeatin as an inhibitor with XO is critical to understanding drug–protein interactions and possible therapeutic uses. These interactions can also give important knowledge about the mechanism of binding of blumeatin to XO at the molecule level. Finally, it is clear that further understanding the molecular structure and biological activity relationship of such particular flavonoids would help to design potential similar compounds that could exhibit enhanced antioxidant activity and give people suffering from HUA hope.

## 2 Materials and Methods

### 2.1 FTIR Spectroscopy

Blumeatin was purchased from Cayman Chemical (Ann Arbor, MI, USA). 1 mg of blumeatin was inserted on the attenuated total reflection (ATR) diamond crystal which is located in the PerkinElmer spectrum two FTIR spectrometer (PerkinElmer Inc., Waltham, MA, USA). This spectrometer has a deuterium triglycine sulfide (DTGS) detector. FTIR spectra for blumeatin were collected in 4000–400 cm^–1^ range at ambient temperature. An average of 64 scans were taken during the spectral collection process. The spectral resolution of the spectrometer was adjusted to 2 cm^–1^ to better distinguish the FTIR spectral peaks. Water and carbon dioxide contributions were eliminated from the spectra by subtracting the initial background spectrum. The spectra were evaluated by the spectrometer’s PerkinElmer Spectrum Version 10.5.4. software (PerkinElmer Inc., Waltham, MA, USA), and ATR-FTIR spectra were plotted with OriginPro 2022 b (Academic Version).^40^

### 2.2 Computational Details

Quantum chemical computation on blumeatin was performed by Gaussian 09^41^ using the B3LYP functional that consists of Becke’s gradient exchange correction;^42,43^ the Lee, Yang, and Parr correlation functional;^44^ and the Vosko, Wilk, and Nusair^45^ correlation functional with the 6-311++G(d,p) basis set.^46^ Computed wavenumbers were scaled by double factors of 0.967 and 0.955 for the wavenumbers below and over 1800 cm^–1^, respectively. Optimizations were performed by the geometry direct inversion of the invariant subspace method.^47^ The optimized molecular structure of blumeatin was found to be a minimum energy conformation. Moreover, for the conformational flexibility determination purpose, a scanning procedure was performed over the dihedral angle (C_2_–C_1_–C_15_–C_20_) between rings C and B. For this purpose, the same level of basis set and correlation functionals mentioned above were used with 12 steps of 30° increments.

### 2.3 ADME/Tox Analysis

To obtain the ADME/Tox data for blumeatin, the graph-based structural signatures concept was utilized.^48^ For this purpose, SMILES (simplified molecular-input line-entry system) code (COC1=CC(=C2C(=O)CC(OC2=C1)C3=CC(=CC(=C3)O)O)O) for blumeatin was introduced into pkCSM, and the ADME/Tox results of blumeatin were obtained.

### 2.4 Molecular Docking

**Figure 2:**
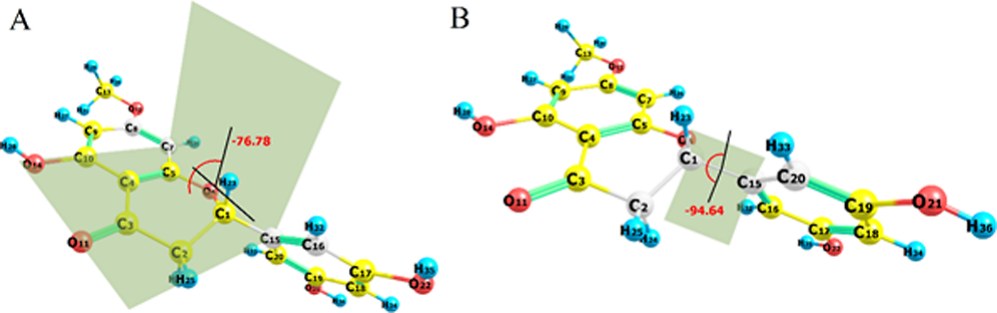
Optimized dihedral angle (C_8_–C_7_–C_15_–C_16_) between rings A and B of blumeatin (A). Scanned dihedral coordinate (C_2_–C_1_–C_15_–C_20_) of blumeatin (B).

**Figure 3:**
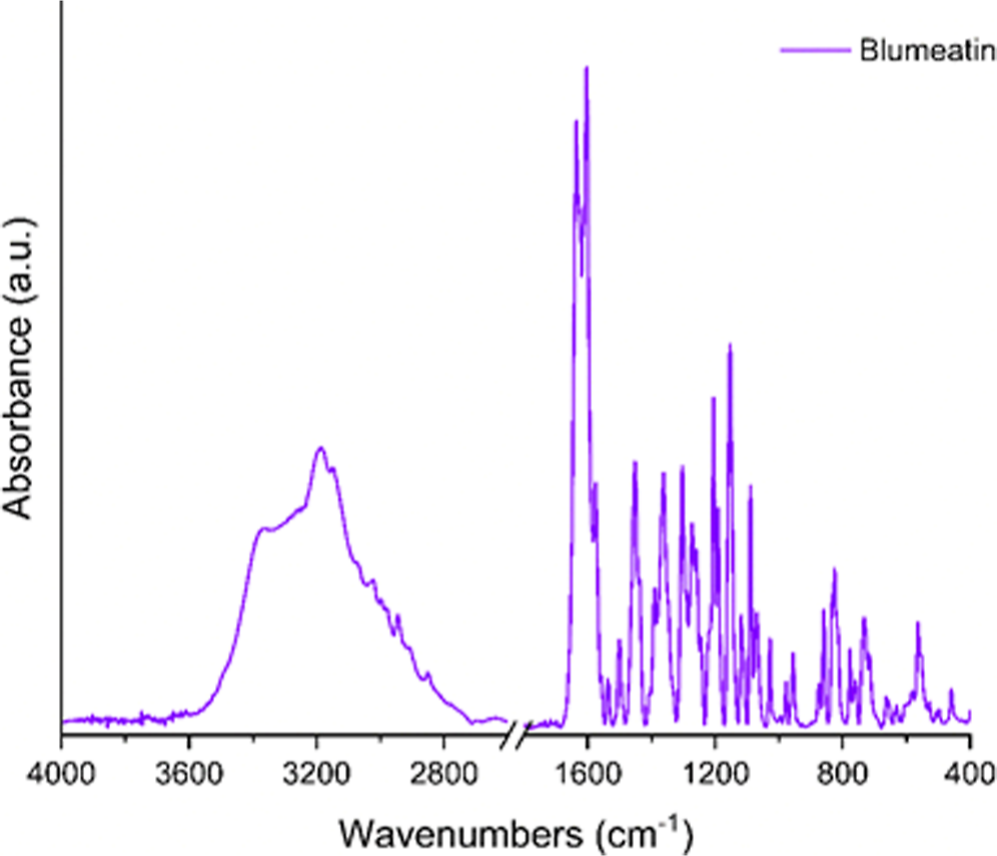
Experimental FTIR spectrum of blumeatin.

**Figure 4:**
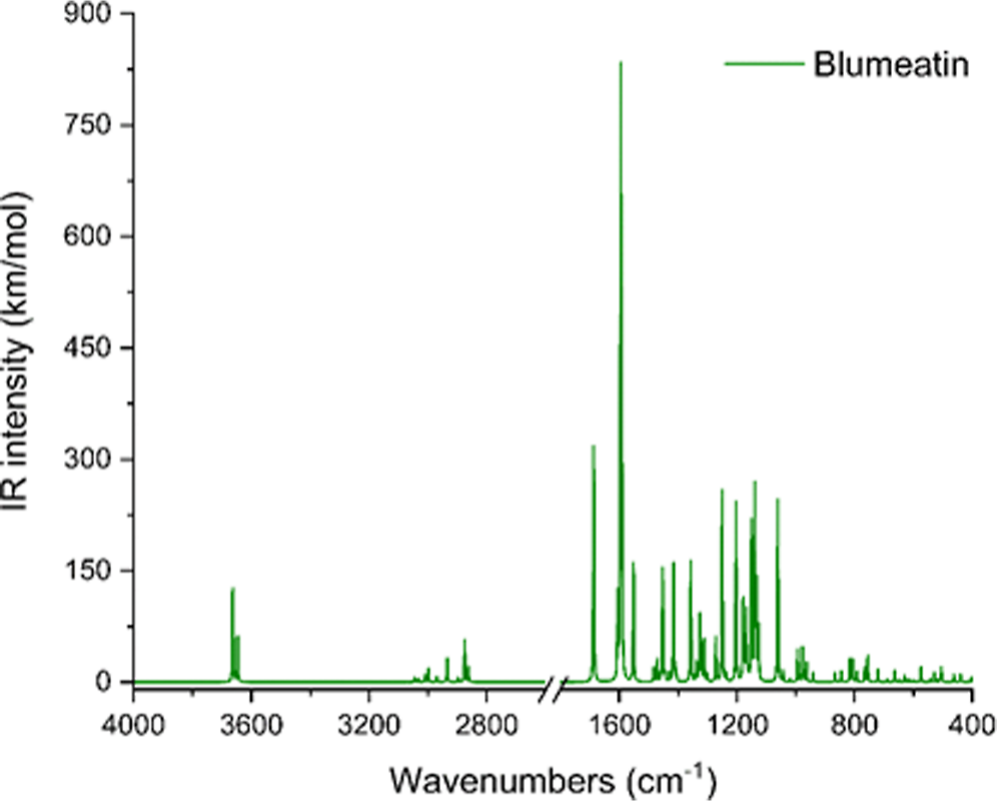
Computed IR spectrum of blumeatin.

**Figure 5:**
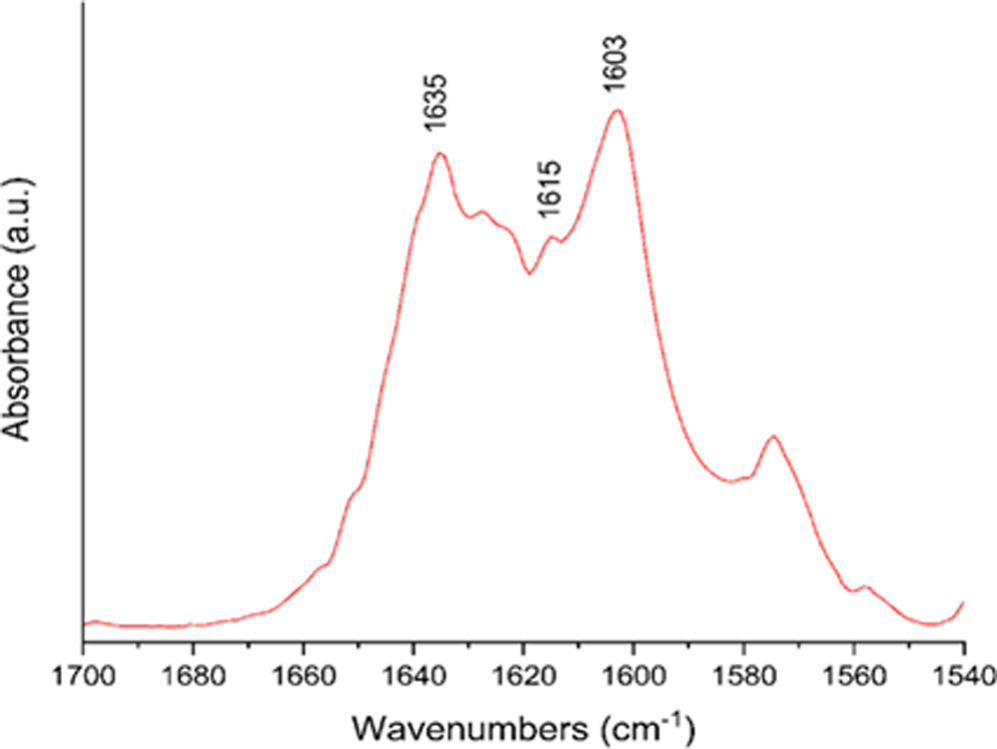
Fermi resonance with its satellite peaks in blumeatin’s FTIR spectrum.

**Figure 6:**
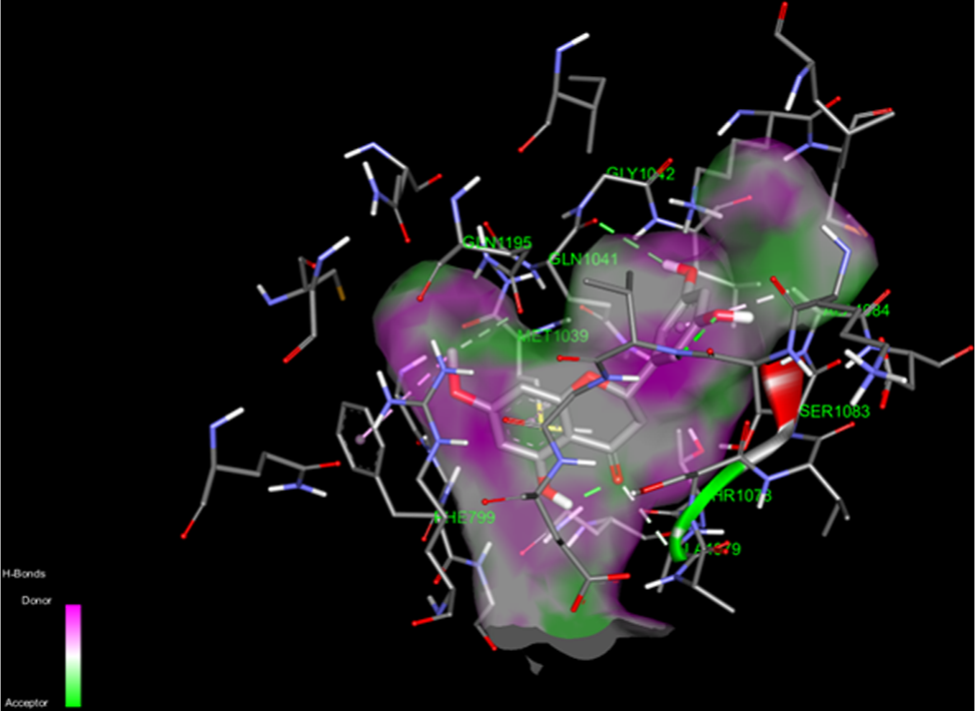
Docked pose of blumeatin into XO.

**Figure 7:**
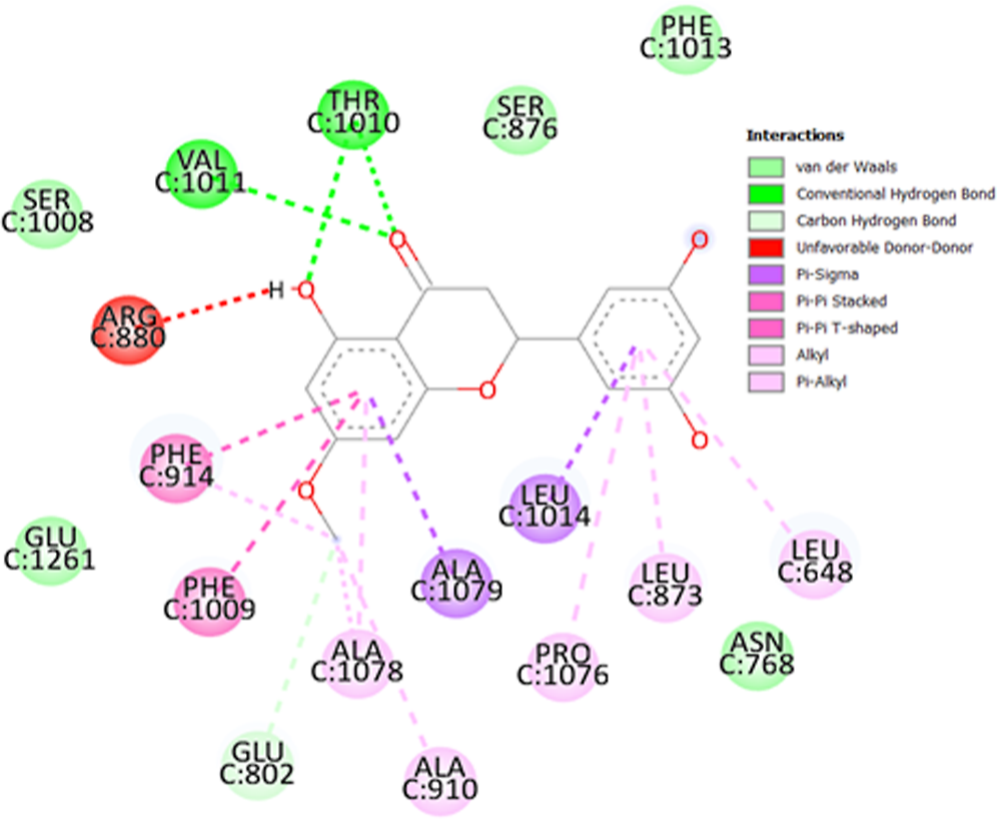
Binding mode of blumeatin on XO and its interaction with the surrounding residues.

For molecular docking study, blumeatin and XO were prepared according to the procedure that was previously reported by Altunayar-Unsalan et al.^31^ The X-ray crystallographic structure of bovine XO with the complex of flavonoid quercetin with 2.0 Å resolution was downloaded from the protein data bank (PDB) (https://www.rcsb.org/) (PDB ID: 3NVY). Optimized molecular structure of blumeatin was used for docking this flavonoid into the site where the cocrystallized ligand (quercetin) of XO resided naturally. Afterward, whole XO protein was sterilized by removing other natural ligands and water using PyMol Molecular Visualization software.^49^ As the next step, both blumeatin and XO were converted to “pdbqt” file format in PyRx 0.8^50^ to get these structures prepared for docking. In order to visualize and make a conclusion which interactions and residues would be involved for blumeatin, BIOVIA Discovery Studio Visualizer v21.1.0.20298^51^ was used. After these interaction residues were determined for blumeatin, they were selected in the search grid box in PyRx by manually toggling them one by one manually. The active site of the protein containing amino acids interacting with blumeatin was predicted by using this grid box. Then, the docking procedure was started by the Auto Dock Vina algorithm which was embedded in PyRx. Binding affinities were obtained in kcal/mol, and resultant docking poses of blumeatin that bound to the previously removed natural ligand’s (quercetin) vicinity was saved in pdb file format for further visualization. In addition, we also applied a homology modeling study using the human protein sequence (NCBI Accession number: NP_000370) and carrying out the molecular docking study with the human 3D structure for the PDB code 2E1Q. Our findings are presented in the Supporting Information as Figures 1–9 and Table S4.

## 3 Results and Discussion

### 3.1 Molecular Structure, Antioxidant Properties, and Quantum Chemical Descriptors

Blumeatin is composed of a flavanone skeleton structure with three OH moieties at three carbon positions (5′, 3′, and 5) and a methoxy group located at the seventh carbon. Blumeatin is composed of three rings (A, C, and B, respectively), and the C_1_ symmetry group was obtained for this compound. There are six different types of atom distances (CC, OC, CH, OO, OH, and HH) and 597 distances in total for blumeatin (Table S1). Computed optimized molecular structure of blumeatin is presented in Figure 1, and related data are given in Table S2. A and C rings are connected together with a C_4_–C_5_ double bond, and the C ring has a single C=O (C_3_–O_11_) group attached to the C_3_ atom. One OH group is attached to the C_10_ atom in ring A. The B ring has two OH groups which make blumeatin support possible H-bond acceptors and donors via the O_21_ and O_22_ atoms. Blumeatin has only one methyl group connected via the O_12_ atom in ring A. van Acker et al.^52^ emphasized that the existence of an OH group at 3-carbon position, C_2_=C_3_ double bond (C_1_–C_2_ single bond for blumeatin’s numbering scheme), and catechol moiety is responsible for the increasing radical scavenging activity of flavonoids. The authors in that study also focused on the torsion angles of ring B and tried to explain the structure–activity relation based on the planarity. They also questioned why this double bond without a hydroxyl group exhibits increasing activity by means of quantum chemical investigation. In this study, the B and C rings of blumeatin are not in perfect planar orientation. The torsion angles are −36.27° (O_6_–C_1_–C_15_–C_20_) for ring B and 51.28° (C_5_–O_6_–C_1_–C_2_) for ring C. Geometrically, rings A and C of flavonoids favor to be in coplanar conformation. For blumeatin, the torsion angle (C_4_–C_3_–C_2_–C_1_) of ring C was computed to be 33.62° as distorted from planarity. However, the torsion angle (C_8_–C_7_–C_15_–C_16_) of ring B compared to the plane where rings C and A located was computed to be −76.78° (Figure 2A) compared to the plane where rings A and B located. This deviation from coplanarity with regard to rings A and C could be an explanation or responsible for blumeatin’s better antioxidant activity as explained by previous papers,^52−55^ and it is obvious that further clarification by systematic theoretical and experimental studies data is required. A computational and crystal diffraction study^53^ is carried out where the most stable conformations were found approximately at 140° for morin and myricetin. Based on the conformation analysis performed on blumeatin, the scanned torsion angle (C_2_–C_1_–C_15_–C_20_) of ring B was found to be −94.64° as it is given in Figure 2B. This deviation from the coplanarity is much more than previously reported^53^ dihedral angles that vary between −42.73 (naringenin) and 35.64° ((+)-catechin), and blumeatin’s better antioxidant activity could also be interpreted by this higher deviation with respect to the most flavonoids.^53^ It was previously discussed that 3-OH portion is important in the antioxidant activity of flavones by its interaction with ring B via a H bond,^53^ and researchers in that study stated that this moiety fixes the position of ring B as coplanar with the C and A rings by forming such a H bond. Blumeatin has only two CH bonds in this moiety, and construction of such a hydrogen bond does not seem possible. Although blumeatin does not have a 3-OH moiety, it is interesting to note that blumeatin exhibits better antioxidant activity compared to most flavonoids. Moreover, it is not possible to suggest at the moment why blumeatin shows such a high activity even it lacks both 3-OH moiety and C_2_=C_3_ double bond which are known as extreme active scavengers together.^55^

A recent investigation on the antioxidant activities of *Alyssum virgatum* by Koç et al.^56^ demonstrated that ionization potential, bond dissociation energies, single electron transfer followed by proton transfer (SET-PT), hydrogen atom transfer (HAT), and proton dissociation enthalpies are indicators of antioxidant reactivities. In that work, hydroxy radicals in ferulic acid have a better antioxidant property than those in cinnamic acid determined in *A.. virgatum*. As it was mentioned in their work, DFT-extracted parameters were shown to be effective in understanding the antioxidant mechanism of compounds under study.^56^ These mechanisms were also investigated for five monophenols (thymohydroquinone, thymol, carvacrol, *para-*cymene-2,3-diol, and dihydroxyphenol) because of their well-known antioxidant characteristics depending on the locations of OH groups in the compound, and these structures were planar.^57^ To date, there has not been any specific work dealing with the correlation of antioxidant activity with the planarity of the flavonoids.

The most detailed correlation by means of evaluation of hydroxyl positions and bond dissociation energies in both aqueous solution and the gas phase was studied on myricetin. It was shown by Hu-Jun et al.^58^ that myricetin and its antioxidant activity are due to the high antioxidation activity of the 4′-OH group in myricetin. This was attributed to very stable H-deleted radical species, conjugation phenomena, and delocalization as well as by an internal hydrogen bond, constructed between the adjacent hydroxyl groups and radicalized O·. For (−)catechin, it was reported that intramolecular hydrogen bonds occurred between OH moieties of the ring B and OH groups of 3- and 5-OH groups with the 4-keto group of ring C.^52^ However, our results revealed no intramolecular hydrogen bonds for blumeatin, and this compound does not have a 3-OH group. Interestingly, it exhibits more radical scavenging activity (RSA) compared to morin, foeniculin, procyanidin, and gallic acid even with the lack of this specific group. Unlikely to the results of van Acker et al.^52^ that suggested 3-OH moiety takes a dominant role in the antioxidant activity via a hydrogen bond, it is obvious that more intense work is required to explain this contraction, particularly the correlation of the existence of the 3-OH group and intramolecular hydrogen bonds.

A large number of flavonoids were investigated for their theoretical and experimental radical scavenging activities (RSA) (in %) by developed the quantitative structure–activity relationship (QSAR) model and equation.^33^ They used the correlation between the number of free phenolic OH groups and the indicator variable, *I*, developed by Lien and co-workers.^59^ According to the updated equation by Amić et al.^33^ (given below), indicator *I* was included that contains the existence (*I* = 1) of *I*_3′_, 4′-diOH or 3-OH or *I*_5-OH_ or 2,3-double bond, otherwise (*I* = 0). This equation was applied to blumeatin and found the RSA to be 88.403%. Our finding is an exact match (88.403%) of 11 flavonoids (quercetin, taxifolin, morin, galangin, kaempferol, luteolin 7-glycosyl, rutin, laricytrin quercetin 3,7-diglycosyl, and laricytrin-3′-glycosyl, myricetin) where at least one of those three criteria met. In contrast, we were not able to compare our prediction to the experimental RSA, but this is planned for our future work. Amić and his co-workers^33^ also reported that the C_2_–C_3_ double bond might not be required for high activity, but a 3-hydroxyl group highly improves the antioxidant activity. Blumeatin lacks both portions but has 3′-5′-diOH groups instead. This contributes to its higher predicted RSA and is in agreement with previous findings.
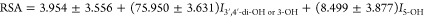


van Acker et al.^52^ suggested that flavonoids (diosmin, kaempferol, galangin, and apigenin) with a 3-OH moiety are planar, whereas flavonoids lacking 3-OH are twisted. As was suggested in that study, blumeatin is also twisted as it lacks a 3-OH moiety. In their frontier work, they demonstrated that the ring B torsion angle with the rest of the compound is in correlation with the scavenging activity owing to the enhanced conjugation. In their work, they presented that rutin with a sugar moiety makes ring B lose its coplanarity, and this was proposed as an explanation of rutin’s less active scavenging profile because it is not able to use its full delocalization potential compared to quercetin which is almost in planar orientation. They also explained that luteolin exhibits a good antioxidant profile because it possesses a twisted torsional angle of 16.29° without a 3-OH moiety. Blumeatin also lacks the 3-OH moiety and twisted torsional angle and exhibited acceptable computed RSA.

Sadasivan and co-workers^60,61^ discussed the intramolecular hydrogen bonds between ring A and the keto group in ring C and the planarity of mearnsetin and myricetin (in their both neutral and radical forms) which is an indication of possible extended conjugation and their antioxidant activity. Based on the bond dissociation energy (BDE) computations, they found that H atom transfer (HAT) from the B ring is easier compared to that from the A ring (C_5_- and C_7_–OH) and C ring (C_3_–OH) for mearnsetin and myricetin. It was also shown that HAT is energetically more favorable from the B ring compared to the C and A rings.^61^ The uniform charge density distribution among the rings was also explained by the planar arrangement of the rings. To the best of our knowledge, their work is the only detailed and systematic work that performed the conformational analysis together with the discussion of a possible correlation between the planarity, charge distribution, BDEs, HAT mechanism, and spin densities of selected flavonoids.

A systematic report on calculations of antioxidant action mechanisms of selected nine isoflavones focused primarily on revealing ionization potentials (IPs), proton dissociation enthalpies, proton affinities, hydroxyl bond dissociation enthalpies (BDEs), and electron-transfer enthalpies in both gas and solution phases.^62^ In that study, authors demonstrated that the lowest BDEs were determined for 3′- and 4′-OH groups in ring B. For the ionization potentials, it was emphasized that ring B possesses an essential role, particularly if it has a 4′-*O*-methyl group.^62^

Attempts have been made to the determination of hydrogen abstraction energies for selected flavonols by DFT methods together with experimental techniques for antioxidant and antiradical activities using ABTS [2,2′-azino-bis(3-ethylbenzothiazoline-6-sulfonic acid)] and DPPH (1,1-diphenyl-2-picrylhydrazyl) radicals and ferric ions (FRAP) tests.^63^ Sroka and co-workers^63^ focused on the 10 different flavonols including luteolin, kaempferol, isorhamnetin, myricetin, rhamnetin, quercetin, apigenin, genkwanin, diosmetin, and chrysoeriol. In their study, they showed that the most active antiradical compounds with a C_3_–OH group were present. Flavones without that group were found to be less active. They discussed the positions of the atoms and their activity in detail and suggested that the positions related to the activity were mostly the positions of the OH groups located at C_4_′, C_3_, and C_3_′. Based on the work published by Rice-Evans and coinvestigators,^64^ Sroka et al.^63^ agreed that OH groups in ring A which are located at C_5_ and C_7_ are not important.

Semiempirical methods like PM6 (Parameterization Method 6) implemented in MOPAC (Molecular Orbital PACkage) software were used to demonstrate the correlation between the electrochemical oxidation potential (EOP), antioxidant activity, and spin population of flavonoid radicals in water. The author tried to show the correlation between flavonoid and antioxidant activity by checking atomic orbital spin populations over the skeleton atoms of a radical molecule and stated that there is an excellent correlation.^65^ In a study where hydrogen bonding formation was discussed, a series of aminophenyl derivatives of kaempferol were synthesized, and intermolecular hydrogen bonding was shown to play a crucial role in primary aminomethyl product formation of kaempferol. Moderate to potent cytotoxic activity against HeLa, HCC1954, and SK-OV-3 human cancer cell lines has also been demonstrated in that work.^66^

A group of flavonoids’ ability to scavenge the DPPH radical was elucidated by means of DFT-based quantum chemical descriptors such as frontier electron density, group philicity, and hardness. It was stated that the obtained QSAR by DFT showed that the lower the global hardness, the higher the activity. Furthermore, four new flavonoids were proposed in that study based on a QSAR model they suggested.^67^ According to the literature, there is no study attempting to correlate the antioxidant profiles together with atomic charges, bond lengths, and bond angles. Previous studies demonstrated that the structural properties are crucial for the high antioxidant activity of flavonoids.^54^ The radical scavenging ability of flavonoids is known to depend on the structures and substituents of the heterocyclic A ring. Furthermore, major determinants of antioxidant activities are the existence of a double bond between C_2_ and C_3_ conjugated with the C_4_-oxo group carbonyl group at C_4_ and the C_3_ hydroxyl group present in flavonols.^54,68,69^

Global quantum chemical descriptors are chemical potential (μ = −χ), global chemical hardness (η), electrophilicity index (ω), electronegativity (χ), global softness (s), and nucleophilicity (ε). These descriptors are calculated from E_HOMO_ (energy of the highest occupied molecular orbital) and E_LUMO_ (energy of the lowest unoccupied molecular orbital) values obtained by quantum chemical computations.^70−75^Table 1 presents the comparison of blumeatin’s quantum chemical descriptors with other selected flavonoids. If calculated band gap energies are small, this suggests that they are highly reactive.^57^

**Table 1:** Comparison of Quantum Chemical Descriptors^a^ (in eV) of Blumeatin to Various Flavonoids

compd	*E*_HOMO_	*E*_LUMO_	Δ*E*	*I*	*A*	χ	η	μ	ω	ε	*s*	ref
blumeatin	–6.56	–1.73	–4.84	6.32	1.48	3.90	2.42	–3.90	3.14	–9.44	0.21	this work
quercetin	–5.03	–2.49	–2.54	5.03	2.49	3.76	1.27	–3.76	5.57	–4.78	0.39	(77)
quercetin	–5.88	–2.10	–3.78	5.88	2.10	3.99	1.89	–3.99	4.21	–7.54	0.26	(78)
taxifolin	–5.44	–2.46	–2.98	5.44	2.46	3.95	1.49	–3.95	5.24	–5.89	0.34	(78)
luteolin	–5.93	–1.67	–4.26	5.93	1.67	3.80	2.13	–3.80	3.39	–8.09	0.23	(79)
gallic acid	–1.23	0.24	–1.47	1.23	–0.24	0.50	0.74	–0.50	0.17	–0.36	0.68	(80)
betulinic acid	–6.47	0.17	–6.64	6.47	–0.17	3.15	3.32	–3.15	1.49	–10.46	0.15	(80)
lupeol	–6.31	0.76	–7.07	6.31	–0.76	2.78	3.54	–2.78	1.09	–9.81	0.14	(80)
procyanidin	–0.04	0.02	–0.06	0.04	–0.02	0.01	0.03	–0.01	0.00	0.00	16.67	(80)
foeniculin	–0.34	–0.16	–0.18	0.34	0.16	0.25	0.09	–0.25	0.35	–0.02	5.56	(80)
morin	–7.17	–0.68	–0.49	7.17	6.68	6.93	0.25	–6.93	97.87	–1.70	2.04	(81)

a *I* = −*E*_HOMO_; *A* = −*E*_LUMO_, stability measure: Δ*E* = *E*_HOMO_ – *E*_LUMO_, χ = (*I* + *A*)/2, chemical potential: μ = −(*I* + *A*)/2, electrophilicity index: ω = μ^2^/2η, global hardness: η = −1/2 (EHOMO-ELUMO), and global softness: *s* = 1/η.

HOMO–LUMO gap energy for blumeatin was found to be −4.84 eV. Based on the computational results, it can be safely suggested that blumeatin exhibits better antioxidant activity than some of the previously reported flavonoids and flavonoid-related derivatives in decreasing order as follows: lupeol > betulinic acid > blumeatin > luteolin > quercetin > taxifolin > gallic acid > morin > foeniculin > procyanidin. However, chemical potential μ is a measure of escaping nature of an electron; thus, the more negative the μ, the more difficult to lose an electron. According to Table 1, blumeatin has higher stability than morin and taxifolin (thus less reactive) but is less stable (more reactive) than the rest of the compounds given in Table 1.

Electronegativity (χ) corresponds to the electron attraction capability of compounds, and based on the results in Table 1, blumeatin has the fourth biggest electronegativity property among other compounds. Hardness (η) and softness (s) descriptors are of importance particularly in defining the behavior of chemical systems since hard compounds have larger energy gap, whereas soft compounds have small energy gap.^76^ As a consequence, soft compounds are more polarizable. Blumeatin has the third highest hardness value (η = 2.42 eV) compared to the first two hardest compounds (lupeol (3.54 eV) and betulinic acid (3.32 eV)). Furthermore, based on the s values, lupeol is the softest compound (0.14 eV), whereas procyanidin has the highest s value (16.67 eV) and blumeatin has the third least softness (0.21 eV) according to our findings and comparison. Another descriptor electrophilicity (ω) is a measure of the energy stabilization upon saturation by the electrons from the external surrounding. It is a hint for the charge-donating capability. A good and more reactive nucleophile is defined by a lower value of ω, whereas the higher values show the existence of a good electrophile. Our comparison suggests that blumeatin exhibits medium nucleophile character due to its electrophilicity value of 3.14 eV among others. The most nucleophile compound is procyanidin, whereas the highest ω value is morin.

### 3.2 Vibrational Assignments

Blumeatin consists of 36 atoms with corresponding 102 vibrational wavenumbers. Normal modes of blumeatin have been assigned based on the individual detailed motion of the atoms. Computed unscaled and scaled FTIR wavenumbers and their assignments for blumeatin are given in Table 2. Experimental and computational IR spectra of blumeatin were plotted as in Figures 3 and 4, respectively. Normal modes used to determine the individual contributions for the vibrational modes are listed in Table S3.

**Table 2:** Vibrational Wavenumbers (cm^–1^), Mode Assignments^a^, and Potential Energy Distributions (%) (PED) of Blumeatin

mode no.	wavenumbers (computed)	wavenumbers (scaled)	wavenumbers (experimental)	notation	assignment with PED (%)^b^
1	14	14	–	τ_ring_	ring torsion CCCC (22) (ring C–B) + torsion OCCC (21) (ring C–B) + torsion HCC = C (11) (ring C–B) + torsion HCCC (11) (ring C–B)
2	36	35	–	τ_ring_	ring torsion (12) (ring C–B)
3	54	52	–	τ_ring_	ring torsion (19) (ring C–B)
4	59	57	–	τ_ring_	ring torsion CCOC (12) (ring A-methyl) + torsion COCH (10) (methoxy)
5	81	78	–	τ_ring_	ring torsion (26) (ring C)
6	105	102	–	γ_methyl_	(CH_3_ twist + ring torsion) (11) (ring A) + ring torsion (10) (A-C)
7	162	157	–	τ_ring_	ring torsion (21) (ring C)
8	180	174	–	ω_CH_^b^	CH_2_ wagging (ring A)
9	189	183	–	γ_CH_	CH_2_ twist (methyl) (27) (ring A)
10	212	205	–	γ_CH_	CH_2_ twist (methyl) (12) (ring A)
11	222	215	–	γ_OH_	OH o.o.p. bending (20) (ring B) + ring torsion (16) (ring B)
12	233	225	–	τ_ring_	ring torsion (87) (ring B)
13	236	228	–	γ_CH_	CH_2_ twist (methyl) (27) (ring A)
14	248	240	–	τ_ring_	ring torsion (23) (ring B)
15	255	247	–	τ_ring_	ring torsion (21) (ring A-C) + methyl torsion (15) (ring A)
16	290	280	–	τ_ring_	CCOH torsion (15) (ring A)
17	297	287	–	τ_ring_	CCOH torsion (10) (ring A)
18	310	300	–	γ_OH_	OH o.o.p. bending (69) (ring B)
19	329	318	–	γ_OH_	OH o.o.p. bending (52) (ring B)
20	338	327	–	δ_COH_	COH bending (16) (ring B)
21	348	337	–	τ_ring_^b^	CCOH torsion (ring A)
22	392	379	–	γ_OH_	OH o.o.p. bending (46) (ring A)
23	417	403	419	τ_methyl_	(methyl + CCOH) torsion (11) (ring A)
24	455	440	–	δ_ring_	ring bending (12) (ring C)
25	478	462	459	τ_CH_	CH_2_ twist (16) (ring C)
26	522	505	499	τ_ring_^b^	ring torsion (ring B)
27	526	509	507	τ_ring_^b^	ring torsion (ring A + B)
28	536	518	–	γ_CH_^b^	CH_2_ twist (ring C) + ring torsion (ring C)
29	546	528	528	γ_CH_^b^	CH_2_ twist (ring C)
30	561	542	553	γ_CH_^b^	CH_2_ twist (ring C)
31	594	574	563	ω_CH3_^b^	CH_3_ wagging (ring A) + ring torsion (ring B)
32	615	595	597	γ_CH_	CH o.o.p. bending (ring B) (30) + ring torsion (17) (ring B)
33	620	600	582	γ_CH_	CCH o.o.p. bending (14) (ring A) + ring torsion (11) (ring A)
34	631	610	606	γ_CH_^b^	CCH o.o.p. bending (ring B)
35	638	617	621	γ_CH_^b^	CCH o.o.p. bending (ring A)
36	652	630	631	τ_ring_	ring torsion (17) (ring A)
37	671	649	656	τ_ring_^b^	ring torsion (ring C)
38	687	664	664	γ_CH_	CH o.o.p. bending (26) (ring B)
39	713	689	688	γ_CH_	CCH o.o.p. bending (12) (ring B)
40	746	721	715	γ_CH_	CCH o.o.p. bending (16) (ring A)
41	781	755	759	γ_CH_	CCH o.o.p. bending (13) (ring A)
42	792	766	–	γ_CH_	CCH o.o.p. bending (21) (ring A)
43	819	792	–	γ_CH_	CH o.o.p. bending (41) (ring B)
44	834	806	–	γ_CH_	CH o.o.p. bending (42) (ring B)
45	844	816	815	γ_CH_	CH o.o.p. bending (28) (ring A)
46	875	846	858	γ_CH_	CH o.o.p. bending (38) (ring A)
47	896	866	874	γ_CH_	CH_2_ twist (20) (ring C)
48	974	942	939	τ_ring_^b^	ring torsion (ring C) + ring bending (ring A) + OC stretching (methyl)
49	996	963	964	τ_ring_^b^	ring torsion (ring C) + OC stretching (methyl)
50	1007	974	–	δ_ring_^b^	ring bending (ring B)
51	1011	978	977	δ_ring_^b^	ring bending (ring B)
52	1027	993	998	δ_CCH_^b^	CCH bending (ring B) + ring bending (ring B)
53	1054	1019	1028	ν_ring_^b^	ring breathing (ring A) + OC stretching (methyl)
54	1079	1043	–	ν_CC_^b^	CC stretching (ring C) + CO stretching (ring C)
55	1088	1052	–	ν_CO_^b^	C–O stretching (methyl) + CCH bending (ring A) + CC stretching (ring C) + CCH bending (ring C)
56	1097	1061	1068	δ_CCH_^b^	CCH bending (ring A) + COH bending (ring A) + CO stretching (ring C)
57	1165	1127	1118	δ_CCH_^b^	CCH bending (ring B) + COH bending (ring B)
58	1167	1128	–	δ_CCH_	CH rocking (methyl) (36) (ring A)
59	1172	1133	–	δ_COH_	CCH bending (23) (ring B) + COH bending (11) (ring B)
60	1178	1139	–	δ_CCH_	CCH bending (19) (ring A + C)
61	1189	1150	1151	δ_CCH_	CCH + COH bending (15) (ring B)
62	1201	1161	–	δ_CCH_	CCH bending (24) (ring A + C + B)
63	1208	1168	–	δ_COH_^b^	COH bending (ring A)
64	1215	1175	–	δ_COH_	COH bending (19) (ring B)
65	1220	1180	1189	δ_CCH_	CCH bending (21) (ring A + C)
66	1244	1203	1204	δ_CCH_	CCH bending (28) (ring A + C + B)
67	1278	1236	1244	δ_HCH_	CH wagging (19) (ring C)
68	1293	1250	1257	δ_CCH_^b^	CH bending (ring A)+ OH bending (ring A) + ring breathing (ring A)
69	1314	1271	1271	δ_COH_	COH bending (41) (ring B)
70	1345	1301	1302	δ_CCH_^b^	CH bending (ring C + ring B) + ring stretching (ring B)
71	1356	1311	–	δ_OCH_	CH bending (19) (ring C)
72	1366	1321	–	ν_ring_^b^	ring stretching (ring A) + OCH bending (ring C) + ring stretching (ring B) + COH bending (ring B)
73	1372	1327	1331	δ_OCH_^b^	OCH bending (ring C) + COH bending (ring A) + COH bending (ring B)
74	1384	1338	1346	δ_OCH_^b^	OCH bending (ring C) + CO stretching (ring A) + methyl umbrella
75	1403	1357	1361	δ_CCH_	CH bending (10) (ring C)
76	1454	1406	1407	δ_CCH_	CH_2_ scissoring (22) (ring C)
77	1463	1415	1419	δ_CCH_	methyl umbrella (24) + OCH bending (25) (ring A)
78	1477	1428	1436	δ_CCH_	methyl umbrella (49)
79	1493	1444	1439	δ_HCH_	HCH bending (methyl) (82) (ring A)
80	1502	1452	1450	δ_CCH_	CCH bending (14) (ring B) + HCH bending (methyl) (10) (ring A)
81	1503	1453	–	δ_HCH_	HCH bending (methyl) (24) (ring A) + COCH torsion (13) (ring A)
82	1521	1471	–	δ_CCH_	CCH bending (17) (ring A) + OCH bending (10) (ring A)
83	1533	1482	1499	δ_CCH_	CCH bending (23) (ring B) + ring stretching (12) (ring B)
84	1604	1551	1553	ν_ring_	ring stretching (18) (ring B)
85	1645	1591	1559	ν_CC_	CC stretching (11) (ring B)
86	1649	1595	1601	ν_CC_	CC stretching (11) (ring A)
87	1660	1605	–	δ_CCH_	CCH bending (19) (ring B) + CC stretching (12) (ring B)
88	1744	1686	1615	ν_C=O_	C=O stretching (15) (ring C) + CC stretching (10) (ring C)
89	2997	2862	2851	ν_CH_	CH stretching (52) (ring C)
90	3011	2876	2872	ν_CH_	methyl symmetric stretching (79) (ring A-methyl)
91	3033	2897	2909	ν_CH_	CH_2_ symmetric stretching (69) (ring C)
92	3073	2935	2944	ν_CH_	methyl asymmetric stretching (81) (ring A-methyl)
93	3110	2970	2980	ν_CH_	CH asymmetric stretching (58) (ring C)
94	3140	2999	2997	ν_CH_	CH stretching (79) (ring B)
95	3152	3010	–	ν_CH_	CH stretching (70) (ring B)
96	3175	3032	3023	ν_CH_	CH stretching (74) (ring B)
97	3190	3046	–	ν_CH_	CH stretching (71) (ring A)
98	3214	3069	–	ν_CH_	CH stretching (73) (ring B)
99	3217	3072	3076	ν_CH_	CH stretching (74) (ring A)
100	3818	3646	–	ν_OH_	OH stretching (90) (ring A)
101	3835	3662	–	ν_OH_	OH stretching (91) (ring B)
102	3836	3663	–	ν_OH_	OH stretching (91) (ring B)

a ν: stretching; δ: in-plane bending; γ: out-of-plane bending; τ: torsion; o.o.p.: out-of-plane; and ω: wagging.

b Individual PED contributions ≤10% were not taken into consideration since their individual contributions are ≤1–3% and do not reach 10% when summed. In this case, tentative assignments that were visually obtained by GaussView were presented.

Three O–H stretching vibrations of blumeatin were computed at 3663, 3662, and 3646 cm^–1^, respectively. These wavenumbers were previously reported to be between 3645 and 3600 cm^–1^ (narrow) for nonbonded hydroxy groups, 3645 and 3630 cm^–1^ for primary alcohols, 3635 and 3620 cm^–1^ for secondary alcohols, 3620 and 3540 cm^–1^ for tertiary alcohols, and 3640 and 3530 cm^–1^ for phenols.^82^ The wavenumber for O–H stretching computed at 3646 cm^–1^ for ring A was seen to be in agreement for hydroxy groups (3645 cm^–1^). Nevertheless, we could not observe the O–H stretching vibrations in the experimental IR spectrum of blumeatin. However, we observed a band at 3367 cm^–1^ as a shoulder profile, and we attributed this band to OH stretching due to the nonbonded hydroxyl group. The experimental 3189 cm^–1^ peak was assigned as C–H stretching, and this is in agreement with the unscaled wavenumber computed at 3190 cm^–1^. It should be kept in mind that since scale factors do not sufficiently predict the experimental wavenumbers for these types of flavonoids, some researchers proposed different scale factors for different regions, and thus, some scaled wavenumbers are not consistent with the experimental ones as in our case. For example, Merrick and co-workers^83^ suggested four different scale factors for such chromone derivatives. Thus, such overestimation between the theoretical and experimental data of blumeatin for the O–H vibrations might be overcome by using multiscaling factors, which is not in our scope in this study. In addition, our computational findings did not yield any intramolecular hydrogen bonding motif.

C–H stretching vibrations are generally known to be observed between 2800 and 3100 cm^–1^,^84^ and particularly, in-plane bending C–H vibrations were previously reported in the 1100–1500 cm^–1^ region while out-of-plane bendings are generally observed in the 800–1000 cm^–1^ region.^85,86^ Based on the computational infrared data of blumeatin (Table 2), we observed C–H stretching vibrations between 3072 and 2999 cm^–1^ and 3076 and 2851 cm^–1^, respectively, in the computed and experimental IR spectra, which are in line with the previous findings for C–H vibrations.^85,86^ Only the C=O stretching vibration of blumeatin is expected to have a very strong intensity band around 1615 cm^–1^, but instead, we observed two satellite peaks (Figure 5) at 1635 and 1603 cm^–1^ at both sides of a split peak at 1615 cm^–1^. This was attributed to a Fermi resonance which occurs when an overtone or a combination band has the same wavenumber or a similar wavenumber to a fundamental vibration.^87^ This splitting here is not a consequence of any fundamental band that might be associated with ν, 2ν, or 3ν that gives rise to 1615 cm^–1^. Instead, we found that two combination bands observed at 1089 and 528 cm^–1^ (1089 + 528 = 1617 cm^–1^) contributed to this Fermi resonance that caused a split at ∼1615 cm^–1^ and yielded two satellites at 1635 and 1603 cm^–1^, respectively.

C–H bending vibrations were computed at 1482, 1471, 1453, 1452, 1444, 1428, 1415, 1406, 1357, 1311, 1301, 1250, 1203, 1180, 1161, 1150, and 1139 cm^–1^ in the predicted IR spectrum. Experimental IR spectra (Table 2) of these computed bending modes can be seen in good agreement with the reported spectra.^85,86^ Methyl asymmetric and symmetric bending vibrations were computed at 1471 (1475 cm^–1^^87^) and 1406 cm^–1^ (1380 cm^–1^^87^), respectively.

In the literature, the ring in-plane C–C bending modes cause relatively weak bands below 1000 cm^–1^.^88^ The ring in-plane-bending modes (ring B) for blumeatin were experimentally observed at 977 cm^–1^ and computed at 978 cm^–1^. Besides, the ring out-of-plane modes mostly appear as a group of weak bands in the experimental IR spectral range 700–100 cm^–1^.^88^ The bands below ∼800 cm^–1^ were mostly attributed to torsional movements of the atoms in blumeatin. We observed the γ_CH_ out-of-plane modes between 874 and 528 cm^–1^ in the experimental IR spectrum, and these data were found to be in agreement compared to our computed vibrational wavenumbers. Our results demonstrated that in-plane and out-of-plane C–H bands agree with each other based on the previous research.^85,86^ Ring torsions were computed below 509 cm^–1^ together with the other torsional modes presented in Table 2.

### 3.3 Pharmacokinetic Properties of Blumeatin

Blumeatin was evaluated for its pharmacokinetics properties, including Lipinski’s rule of five,^89,90^ drug-likeness, and ADME/Tox analysis. No violation of Lipinski’s rule of five parameters (the molecular weight is less than 500 g/mol, MLOGP (Moriguchi octanol–water partition coefficient) ≤ 4.15, the number of oxygen atoms is less than 10, and the number of NH or OH groups is less than 5) was found for blumeatin. Its molecular weight is 302.28 g/mol; it has six oxygens and has no NH group and less than five OH groups. pkCSM web tool^48^ and SwissADME interface^91^ were utilized to obtain the ADME/Tox data for blumeatin, and the results are presented in Table 3.

**Table 3:**
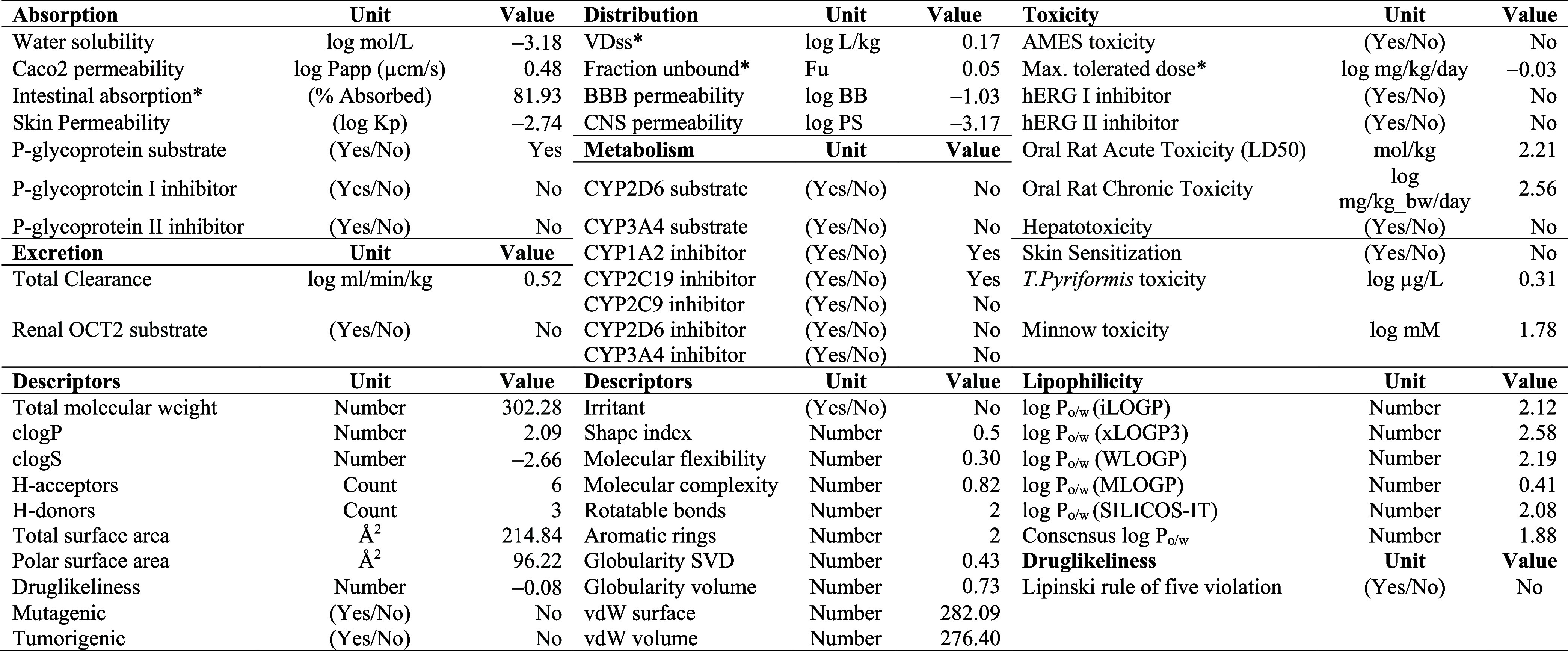
ADME/Tox Evaluation and Molecular Descriptors for Blumeatin (*: for Human)

#### 3.3.1 Absorption

Absorption was computed via Caco_2_ permeability, water solubility, skin permeability, human intestinal absorption, and whether the molecule is a P-glycoprotein substrate or an inhibitor. The water solubility of the compound reflects at 25 °C. The LogS (aqueous solubility) value of blumeatin was calculated as −3.53, which shows that this compound is moderately soluble in water (LogS between −3 and −4).^91^ The ultimate bioavailability (a drug having a value of more than 0.90 is considered readily permeable) is determined by Caco_2_ permeability and human intestinal absorption. Blumeatin showed poor permeability (0.48). The drugs are known to be absorbed primarily in the human intestine. Hydrophilic compounds absorbed rapidly, and a compound with greater than 30% absorbance is regarded as readily absorbed. The human intestine absorption value for blumeatin was calculated to be 81.93%, and it can be considered that blumeatin has a high absorption profile in the human intestine. Blumeatin is the substrate for P-glycoprotein; however, this flavonoid was found as neither P-glycoprotein I nor P-glycoprotein II inhibitors.

#### 3.3.2 Distribution

pKCSM tool allows us to calculate distribution by the human volume of distribution, central nervous system permeability, blood–brain barrier, and human fraction unbound in plasma. The volume of distribution is a theoretical volume that describes the drug’s overall dose. The more the VDss, the higher a drug is distributed in tissue. For antibiotics and antivirals, a more extensive tissue dispersion is preferred. VDss is low if the log VDss value is less than −0.15, whereas the value higher than 0.45 is regarded as high.^92−94^ This value for blumeatin is 0.17, and this indicates that blumeatin exhibits a high VD_ss_ effect.^48,93^ In the case of serum proteins, many plasma drugs will appear in symmetry between the unbound and bound states. Fraction unbound to human plasma must be between 0.2 and 1.0. Blumeatin exhibits a value of 0.05, which is not in this region. The more the compounds bind to the plasma, the less effective they can transverse cellular membranes or diffuse. This means that blumeatin does not bind to the plasma, and thus it cannot transverse cellular membranes or diffuse.^48^

#### 3.3.3 Metabolism

The drug metabolism depends on the compound being a cytochrome p450 substrate or an inhibitor. Cytochrome p450 exists in the liver and acts as a detoxifying enzyme. It is of importance to confirm whether a molecule is capable of inhibiting cytochrome p450. This cytochrome p450s inhibitors’ isoforms are CYP2C9, CYP1A2, CYP2D6, CYP2C19, and CYP3A4.^48,93^ Blumeatin is the only inhibitor of CYP1A2 and CYP2C19 cytochrome enzymes, which demonstrates that it will be metabolized by the enzyme’s action, suggesting that they will not be hampered through the body’s biological transformation. Blumeatin is a noninhibitor of the rest of the cytochrome enzymes given in Table 3.

#### 3.3.4 Excretion

Excretion is expressed by total clearance and whether a compound is a renal OCT2 substrate. Organic cation transporter 2 (OCT2) is a renal uptake transporter that deposits drugs into the kidney. Based on our results, blumeatin is not a renal OCT2 substrate, and it showed a total renal clearance of 0.52 which is less than 1 mL/min/kg, and it is found not to be a renal OCT2 substrate.

#### 3.3.5 Toxicity

The AMES test with a negative value shows that the compound is nonmutagenic and noncarcinogenic. Blumeatin showed no AMES toxicity. Besides, the maximum recommended tolerance dose (MRTD) gives a prediction for the toxic dose in humans. MRTD ≤ log 0.477 (mg/kg/day) is known as low^48^ and blumeatin was found to have low toxicity to humans (Table 3). hERG (human ether-a-go-go gene) is in charge of blocking potassium channels.^48,93,95^ Blumeatin is a noninhibitor of both hERG1 and hERG2. It is nonskin sensitive, and it also does not induce hepatotoxicity. For a certain compound, the LD_50_ value corresponds to the amount that kills 50% of the test animals. Blumeatin showed an oral rat acute toxicity of 2.56 mol/kg, which was considered to be not toxic.

### 3.4 Molecular Docking Study of Blumeatin–Xanthine Oxidase System

It is recognized that hydrogen bonding is critical for secondary and tertiary structural protein structures. In molecular dynamics calculations, hydrogen bond interaction between a ligand and a protein region reflects the binding ability of a drug toward a protein target; therefore, the bigger the amount of H-bonds, the greater the interactions.^96,97^ Blumeatin has six hydrogen bond acceptors and three bond donors, and it is a relatively good candidate for potential hydrogen bonding source. Data obtained from molecular docking investigation are given in Table 4. Detailed docking orientation of blumeatin with its neighbor residues is shown in Figure 6. The two-dimensional representation of the docking environment of blumeatin into XO is demonstrated in Figure 7. Binding affinity for blumeatin to XO is found to be −9.2 kcal/mol, and this is in line with and close to several flavonoids and their binding affinities for XO (−9.993, −9.987, −9.563, −9.289, −9.252, −9.221, −9.158, −9.145, −8.857, −8.842, −8.791, −8.775, and −8.860 kcal/mol for quercetin, luteolin, tectochrysin, kaempferol, naringenin, genkwanin, myricetin, acacetin, apigenin, hesperetin, luteolin-3′-methyl ether, quercetin-3-methyl ether, and eriodictyol, respectively).^98^ Any interactions with crucial catalytic residues such as Glu1261 were not observed. This finding supports that the previously reported observation states that the existence of Glu1261 is crucial to block the XO activity.^99−102^

**Table 4:** Comparison of the Polar (Hydrogen Bonds) and Nonpolar Interactions (Hydrophobic, van der Waals Contacts) of Blumeatin and Quercetin (Natural Ligand) with the Residues from the Active Site of XO

ligand	conventional hydrogen bonds, bond lengths (in Å in parentheses), and types^a^	residues in van der Waals contact or hydrophobic interaction
blumeatin	via O atom of **Thr1010**: 2.70:C=O and 3.26:O–H; via N atom of **Thr1010**: 2.93:C=O and 2.97:O–H; **Val1011** (3.08:C=O); **Arg880** (2.03:O–H); **Phe914** (3.48:π–π and 4.04:(O–H) π–σ); **Phe1009** (π–π); Ala1078 (3.58:O–H and 5.22:π–alkyl); **Ala1079** (3.96:π–σ); **Leu1014** (3.47:π–σ); **Leu873** (4.74:π–alkyl), **Leu648** (5.05:π–alkyl), Ala910 (4.37:π–alkyl)	Glu802, Asn768, Ser1008, Glu1261, Phe1013, Phe1150, Ser876, Lys771, Thr803
quercetin	**Arg880**, **Thr1010**, **Glu802**, **Phe914**, **Ala1079**, **Phe1009**, **Leu873**, **Leu1014**, **Leu648**, **Val1011**	Ser1008, Phe1150, Ser876, Phe1013, Lys771, Pro1076, Ala1078

a Bold text denotes mutual residues that are in interaction with both blumeatin and quercetin.

As can be seen in Table 4, the natural ligand (quercetin) of XO exhibits a hydrophobic bond type with Ser1008, Phe1150, Ser876, Phe1013, Lys771, Pro1076, and Ala1078 residues, whereas it is in interaction with Arg880, Thr1010, Glu802, Phe914, Ala1079, Phe1009, Leu873, Leu1014, Leu648, and Val1011. It is interesting to note that no hydrogen-bonded interaction was observed between quercetin and Ala 910 and Ala1078, whereas blumeatin is in contact with these two residues by pi-alkyl type interaction. According to Table 4, eight conventional hydrogen bonds formed between Thr1010 (C=O and O–H), Val1011 (C=O), Arg880 (O–H), Phe914 (O–H), and Ala1078 (O–H) are 2.70, 3.26, 2.93, 2.97, 3.08, 2.03, 4.04, and 3.58 Å, respectively. Strong (almost covalent), moderate (mostly electrostatic), and weak electrostatic hydrogen bonds refer to the distances between 2.2 and 2.5, 2.5–3.2, and 3.2–4.0 Å, respectively.^103,104^ Based on these definitions, blumeatin binds to XO with very strong (almost covalent) bonds (2.03 Å) via Arg880, moderate hydrogen bonds (2.70, 2.93, and 2.97 Å) via Thr1010, and weak hydrogen bonds (3.26, 3.08, 4.04, and 3.58 Å) via Thr1010, Val1011, Phe914, and Ala1078, respectively. Finally, the rest of the residues in XO surrounding blumeatin formed hydrophobic interactions via Glu802, Asn768, Ser1008, Glu1261, Phe1013, Phe1150, Ser876, Lys771, and Thr803 (mainly van der Waals and hydrophobic).

Besides, based on our human 3D structure (2E1Q) study, the first nine binding structures and the interactions of the surrounding residues for blumeatin are also given in Figures S1–S9 and Table S4. Accordingly, blumeatin pose 1 (hereafter, blumeatin 1) interacts with its surroundings via two conventional hydrogen bonds over Lys1046 and Ala1080. In this docking conformation, it is also in contact with various forms of interaction like alkyl and pi-alkyl as shown in Figures S1 and S2. Blumeatin 1 has a binding affinity of −9.6 kcal/mol, which is the best docked ligand for 2E1Q. Blumeatin 2 shows conventional hydrogen bonds over Gln112, Arg913, and Ser1083. Its docking geometry and other interactions as depicted in Figures S3 and S4 are via Gly1040, Phe799, Met1039, Cys150, Gln1195, and Gln1041 residues with the binding affinity of −9.4 kcal/mol. Blumeatin 2 also has two pi–sulfur interaction types over Met1039 and Cys 150. This is the only docked pose among nine poses by pi-sulfur, amide-pi stacked, and pi–pi stacked interaction types we found for blumeatin, and even this configuration is not the best binding affinity. At this point, we have to admit that pi-sulfur, amide-pi stacked, and pi–pi stacked types of interactions are subject of a different type of study that requires more attention. Blumeatin 3 (Figure S5) is in interaction with Asn261 and Leu404 hydrogen bonds and other types of interactions by Ile353, Ile264, Leu257, Val259, Gly350, and Ser347 residual neighbors (Figure S6). We found its binding affinity to be −9.2 kcal/mol. Fourth hit after our docking effort is blumeatin 4, and this ligand exhibits conventional hydrogen bonds over Leu404 and Ile264 residues. Other types of interactions for this pose of blumeatin 4 were seen between Val259, Ile353, Leu257, val259, and Glu402 residues with a binding affinity of −9.2 kcal/mol. Blumeatin 5 (Figure S9) was observed to construct hydrogen bonds with Gly260, Asn261, and other bonds via Leu257, Ile403, Ile 353, Val259, Ala346, Pro281, Val258, Ser347, and Thr262 residues (Figure S10). It binds to the natural ligand’s pocket of 2E1Q with a binding affinity of −9.0 kcal/mol. Our findings demonstrated that blumeatin 6 (Figure S11) is in interaction with the binding affinity of −9.0 kcal/mol with its environment via hydrogen bonds over Leu404, Lys256, Lys249, Gly350, and Ala301 but other interaction types with Val259, Gly349, Glu402, Ser399, Ile353, and Leu257 residues (Figure S12). This ligand conformation is the one that has the largest number of hydrogen bond contacts among other docked poses. Besides, when blumeatin 7 (−9.0 kcal/mol of binding affinity) is considered (Figure S13), it was seen that it binds to the protein via conventional hydrogen bonding over Leu404, Glu263, and Gly260. Other residues that are shown in Figure S14 (Thr262, Val259, Asn261, Ala346, Ser347, Gly350, Ley257, Ile353, Leu287, Pro281, and Ile403) were seen to be in contact with the pi-donor hydrogen bond, alkyl, pi-alkyl, and van der Waals types of interactions. Blumeatin 8 (Figure S15) exhibits three hydrogen bonding interactions and six various types of interactions as given in Figure S16. These residues are given in Table S4. This conformation’s binding affinity was found to be −8.8 kcal/mol. Two hydrogen bonds were seen to form between Val1260 and Gln1041 for blumeatin 9 (Figure S17) docking pose with a binding affinity of −8.7 kcal/mol. Ala1079, Gln1195, Arg913, Gly1261, Lys1046, Leu1403, Met1039, Phe799, and Ala1084 residues are in different types of interactions rather than hydrogen bonding as presented in Figure S18.

In addition to the discussion above, recent studies showed that blumeatin was also studied by docking strategies, and it was shown that it was docked with the highest score against *N*-myristoyltransferase as an inhibitor together with other various flavonoids including naringin. In their study, Hadi and Nastiti also demonstrated that blumeatin interacted with Tyr, Leu, Asn, and Cys residues.^103^ Another study focused on a detailed review on selected particular flavonoids including blumeatin and their chemical constituents, and bioactivities were evaluated, but their study did not give any detailed information on blumeatin.^104^ On the other hand, two selected flavonoids (blumeatin and luteolin) were also docked against caspase-1 by optimizing these two ligands by semiempiric optimization method (AM1), and it was considered that blumeatin and luteolin were suggested to be anti-inflammatory agents.^105^ Another recent publication by Xia et al. in 2023 stated that blumeatin was synthesized and confirmed its hydroxyl groups at positions C-3′ and C-5′ by experimental NMR spectroscopy, and they only reported selected IR peaks rather than detailed individual vibrational modes as we present here. They also mentioned that the isolation procedure of this flavonoid from plants has limitations of prolonged duration and high cost.^106^

## 4 Conclusions

In this study, the first computed and experimental IR spectra of blumeatin were presented. Computational results indicated no intramolecular hydrogen bonding for blumeatin. Many investigations separately focused on the geometric structures of flavonoids and tried to reveal structure–activity relationships only based on selected several structural parameters and charge-transfer mechanisms. To the best of our knowledge, there are not many attempts to correlate the planarity, antioxidant activities, and experimental and theoretical IR spectra at the same time. Blumeatin showed better theoretical radical scavenging activity compared with several flavonoids. Our attempt on constructing a link between the geometric structure and the activity of blumeatin would provide a stimulus for further systematic and detailed experimental and theoretical research that is required to better reveal the robust connections between action mechanisms and geometric descriptors of flavonoids. To figure out the roles of several quantum chemical descriptors/parameters in the antioxidant activities of both neutral and radical flavonoids and better understand their action mechanism and nature, some questions are crucial to ask. How do (i) the positions of the hydroxyl and other functional groups (methyl, carbonyl, etc.), (ii) spin densities and localizations on the individual atoms in the flavonoid, and (iii) existing intra/intermolecular bonding and the numbers of these bonds affect the nature of the action of flavonoids? For these purposes, both systematic theoretical (conformational analyses, determination of spin densities, HOMO–LUMO study, etc.) and experimental future work are required to unravel the whole picture.

## Supporting Information Available

Distance types and distances in blumeatin, optimized geometric structure of blumeatin, normal modes used in the determination of vibrational modes and their contribution, comparison of the polar (hydrogen bonds), and nonpolar interactions (hydrophobic, van der Waal’s contacts) of blumeatin with the residues from the active site of 2E1Q PDF
