# Supplementary material for: Molecular Structure, Antioxidant Potential, and Pharmacokinetic Properties of Plant Flavonoid Blumeatin and Investigating Its Inhibition Mechanism on Xanthine Oxidase for Hyperuricemia by Molecular Modeling
Source: ACS Omega. 2024 Mar 11;9(11):13284–97. doi: 10.1021/acsomega.3c10083 (PMC10956095; doi:10.1021/acsomega.3c10083)
Supplement: Supplementary file 1 — ao3c10083_si_001.pdf [file ao3c10083_si_001.pdf]

Molecular structure, antioxidant potential and pharmacokinetics properties of plant flavonoid blumeatin and investigating its inhibition mechanism on xanthine oxidase for hyperuricemia by molecular modeling

Cisem Altunayar-Unsalan[a,b], Ozan Unsalan[c,*]

[a]*Ege University, Graduate School of Natural and Applied Sciences, 35100, Bornova, Izmir, Türkiye*

[b]*Ege University Central Research Testing and Analysis Laboratory Research and Application Center, 35100, Bornova, Izmir, Türkiye*

[c]*Ege University, Faculty of Science, Department of Physics, 35100, Bornova, Izmir, Türkiye*

*Corresponding author. E-mail address: ozan.unsalan@ege.edu.tr (O. Unsalan)

Postal address: Ege University, Faculty of Science, Department of Physics, 35100, Bornova, Izmir, Turkey

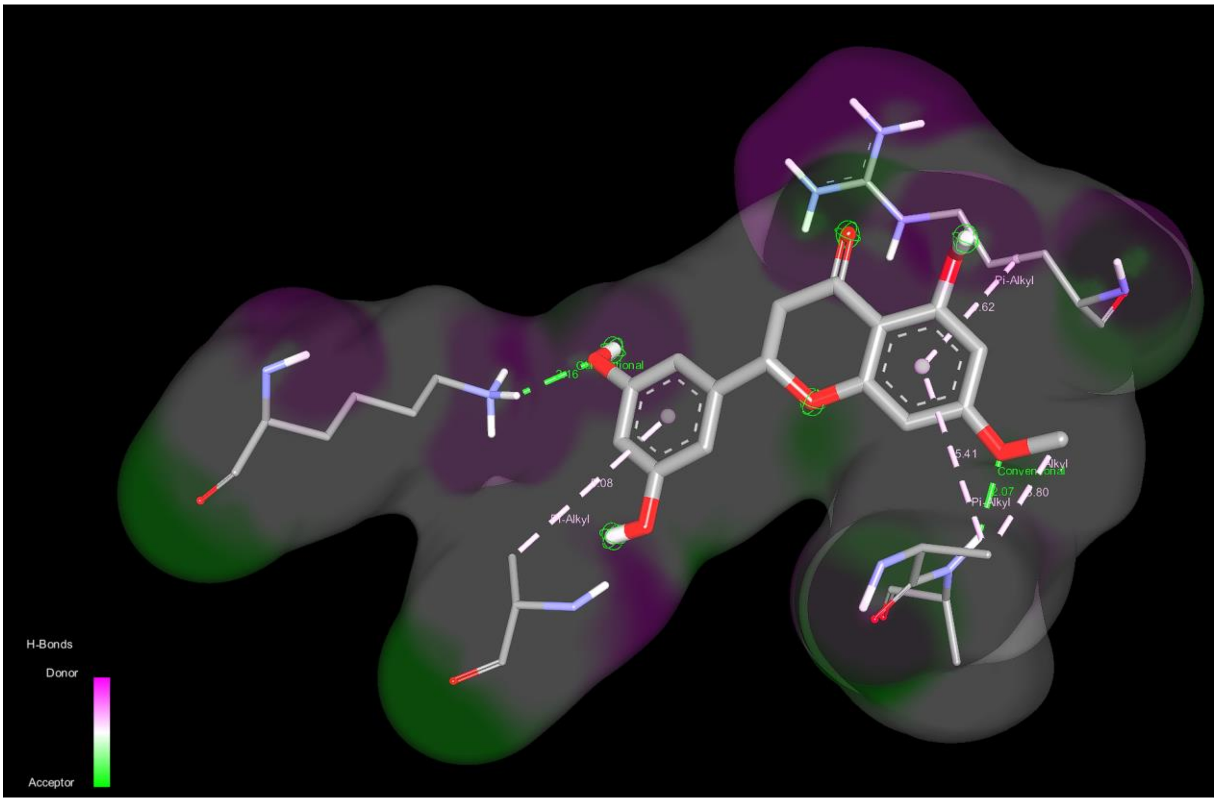

Figure S1. Docked pose 1 of blumeatin into 2E1Q.

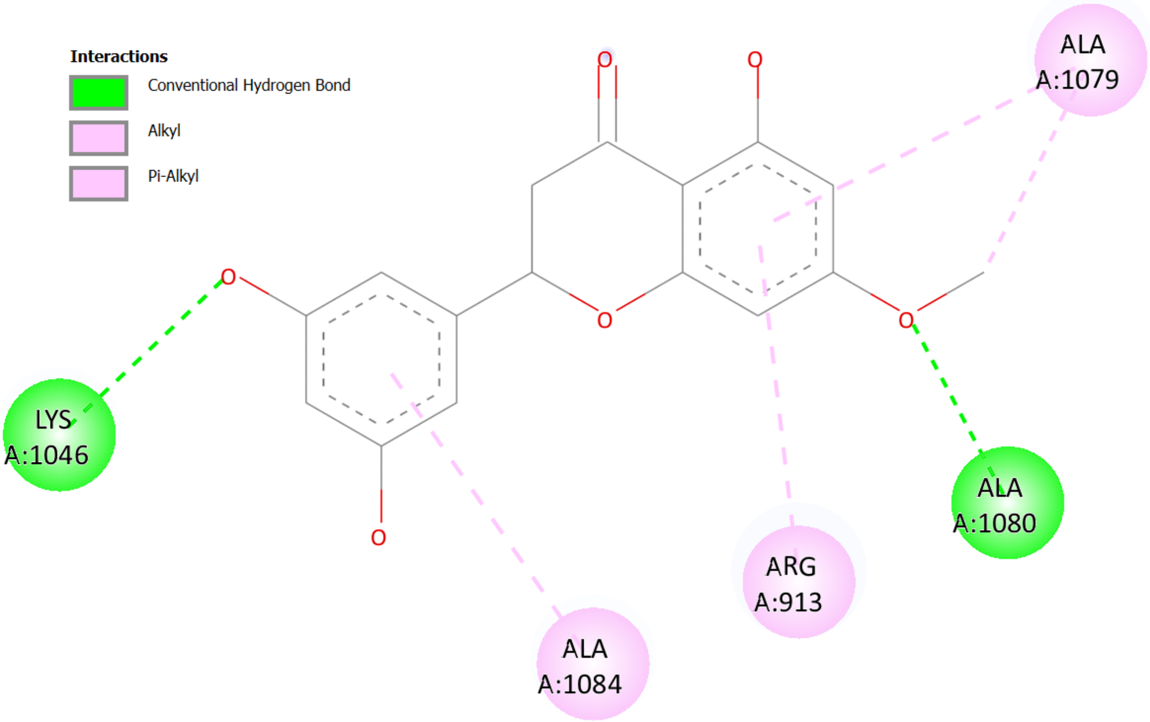

Figure S2. Surrounding residual interactions of blumeatin's docked pose 1 on 2E1Q.

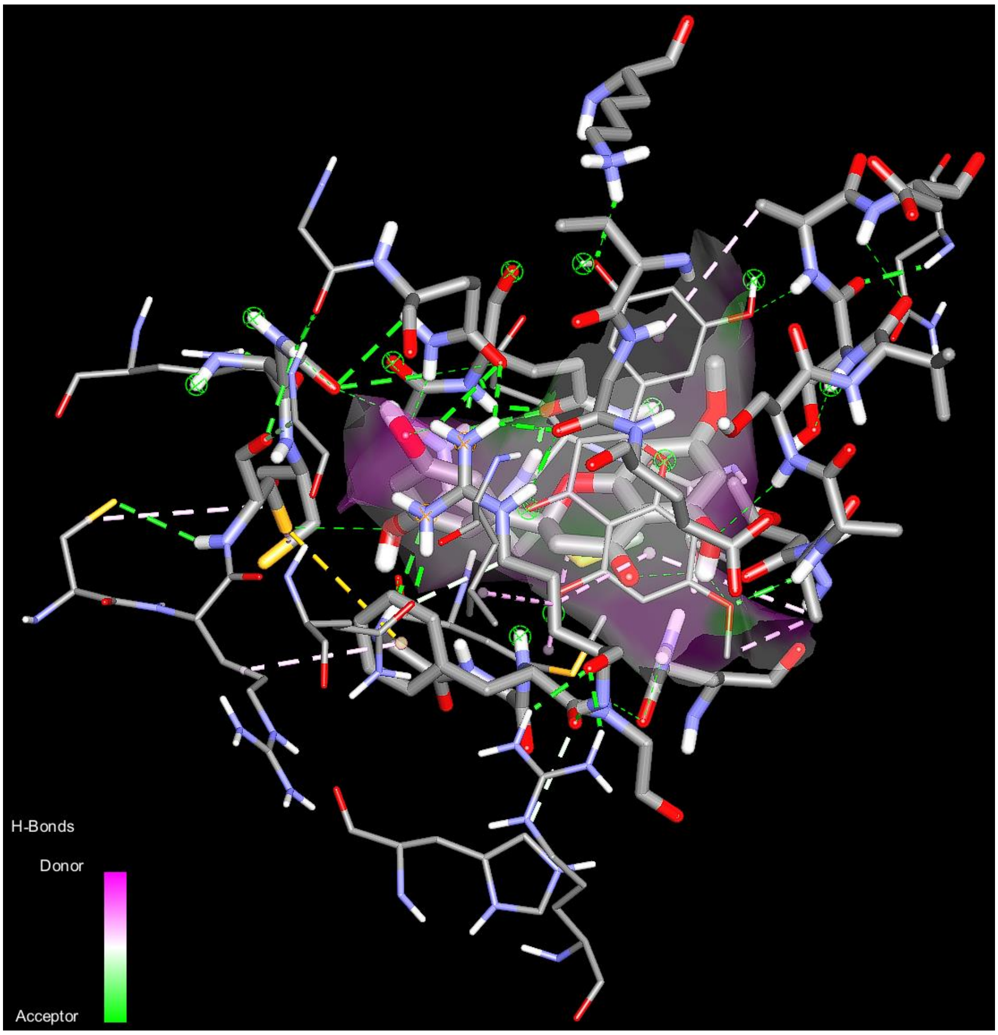

Figure S3. Docked pose 2 of blumeatin into 2E1Q.

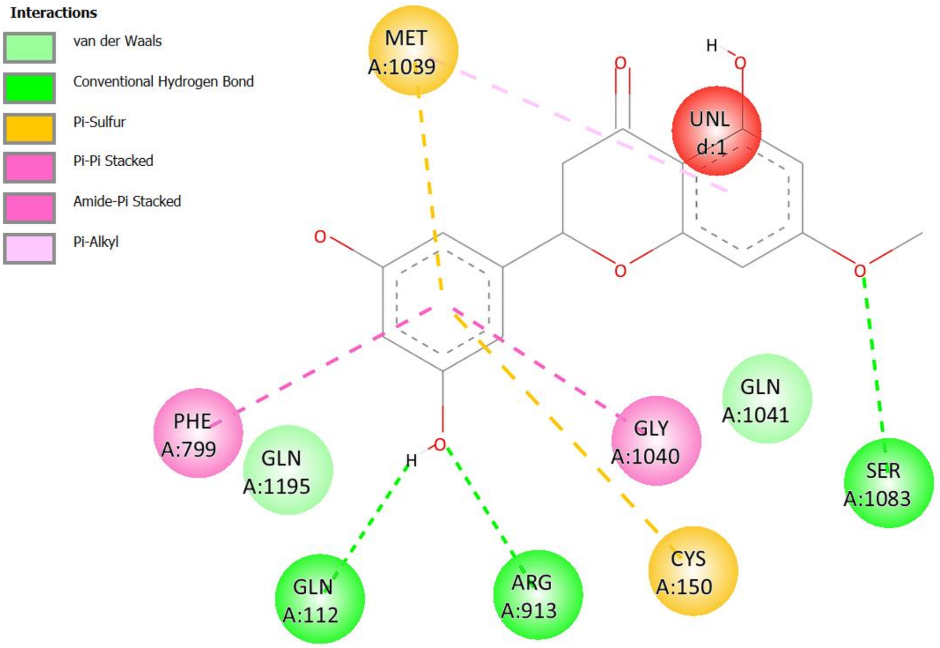

Figure S4. Surrounding residual interactions of blumeatin's docked pose 2 on 2E1Q.

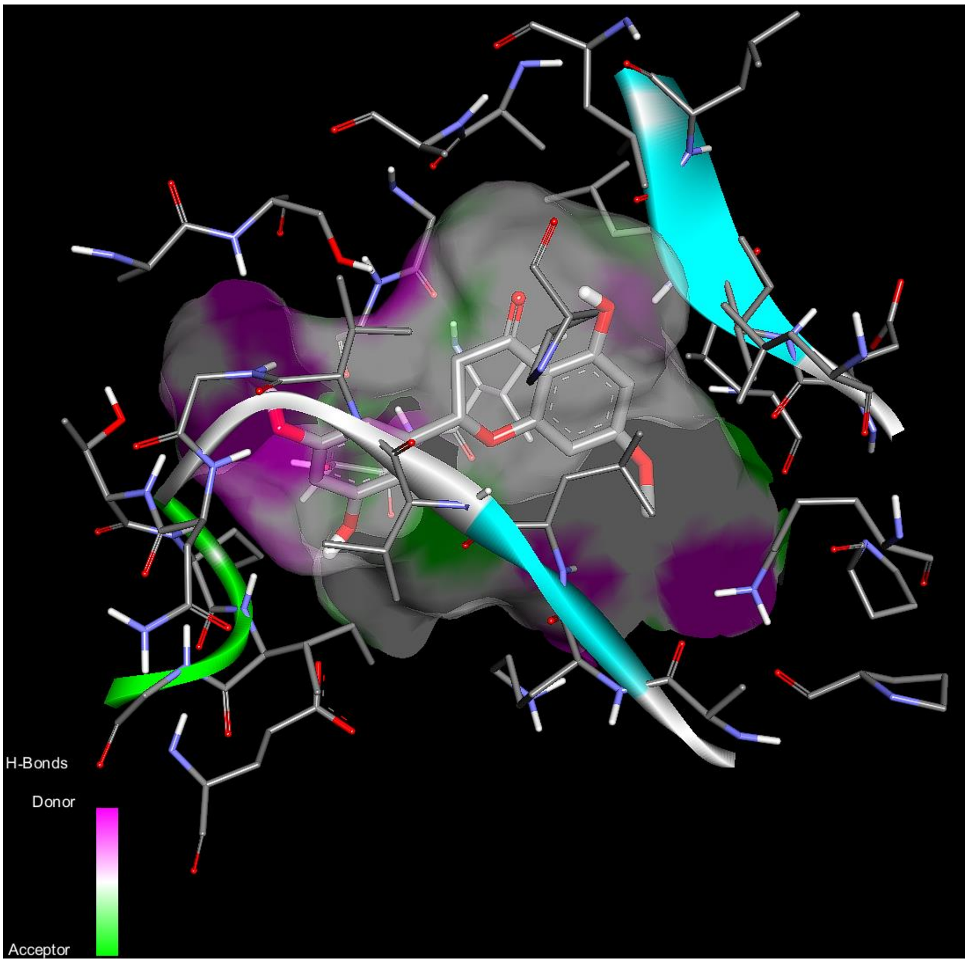

Figure S5. Docked pose 3 of blumeatin into 2E1Q.

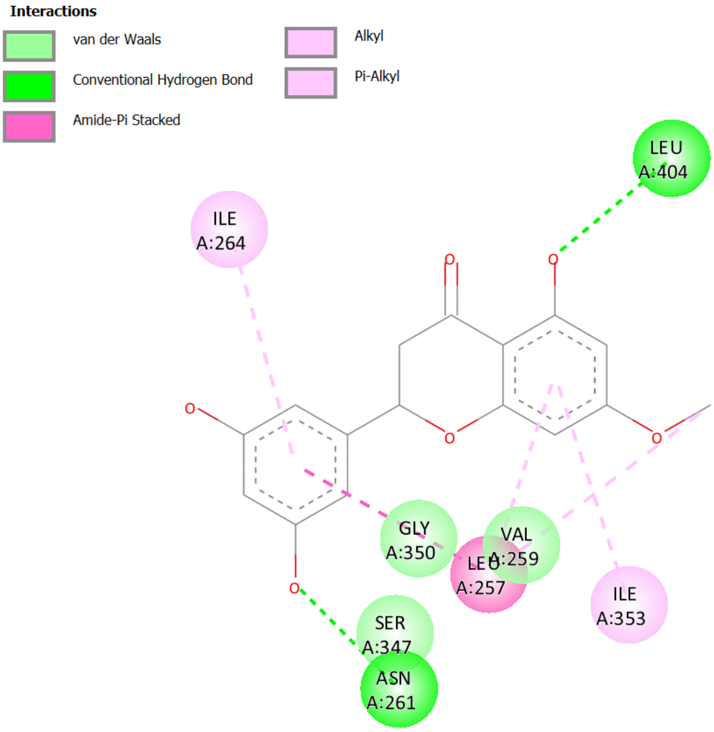

Figure S6. Surrounding residual interactions of blumeatin's docked pose 3 on 2E1Q.

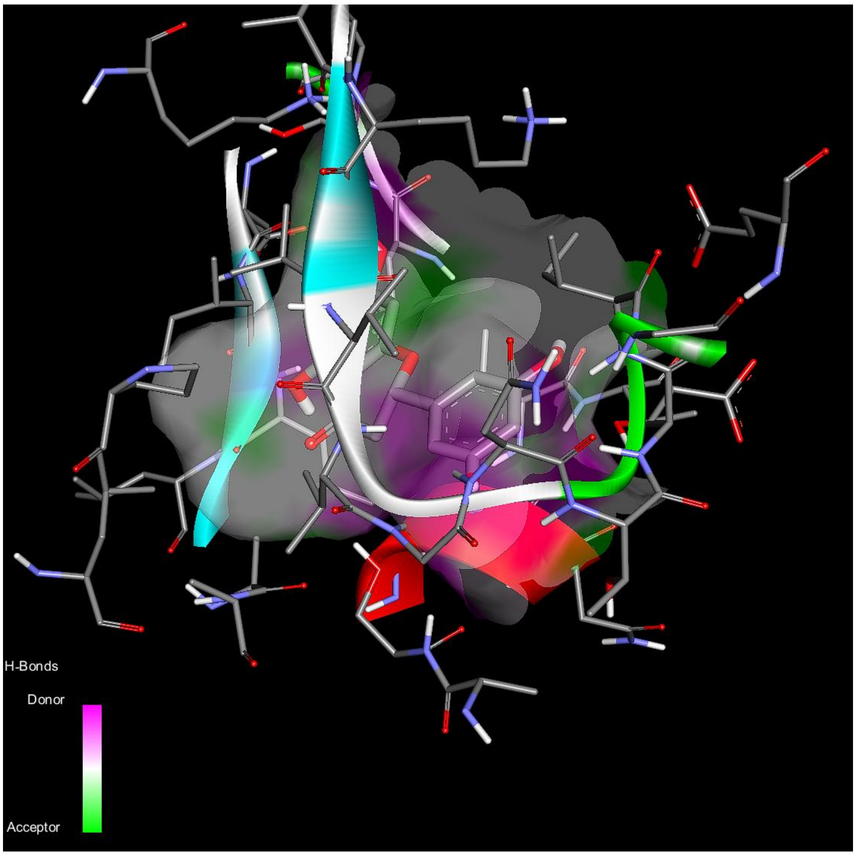

Figure S7. Docked pose 4 of blumeatin into 2E1Q.

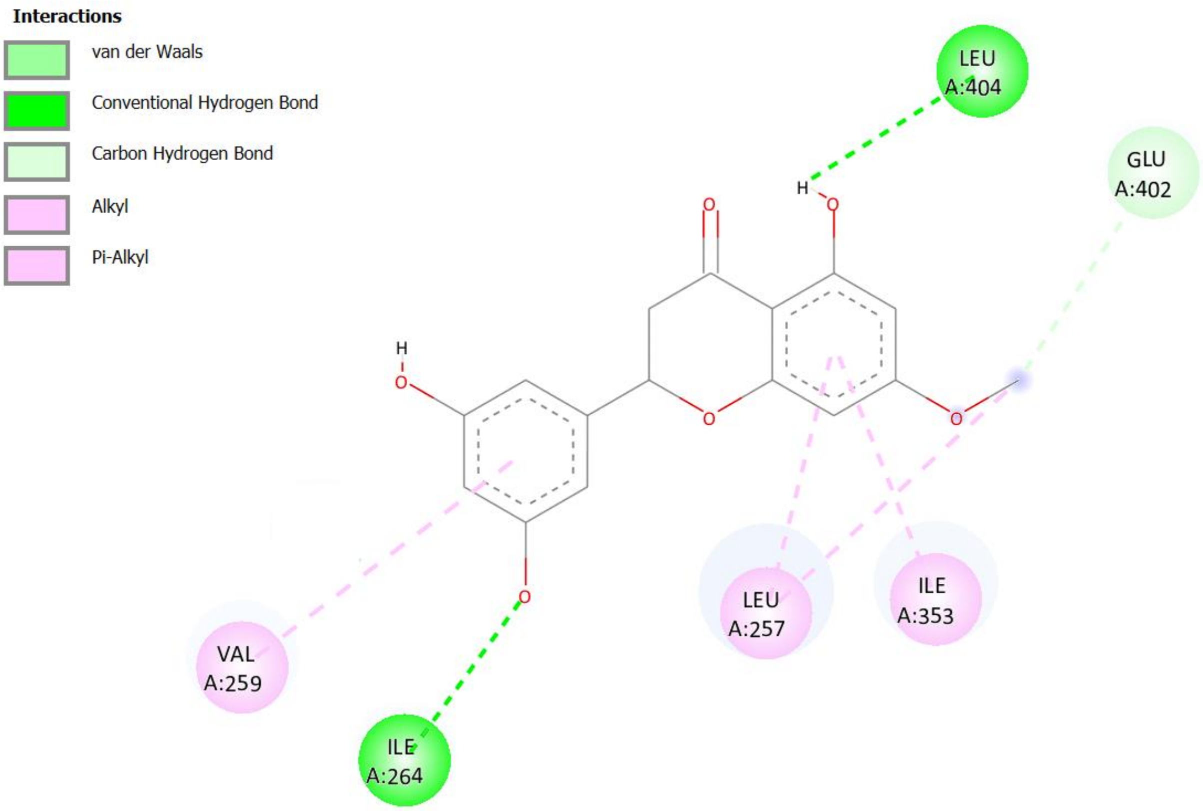

Figure S8. Surrounding residual interactions of blumeatin's docked pose 4 on 2E1Q.

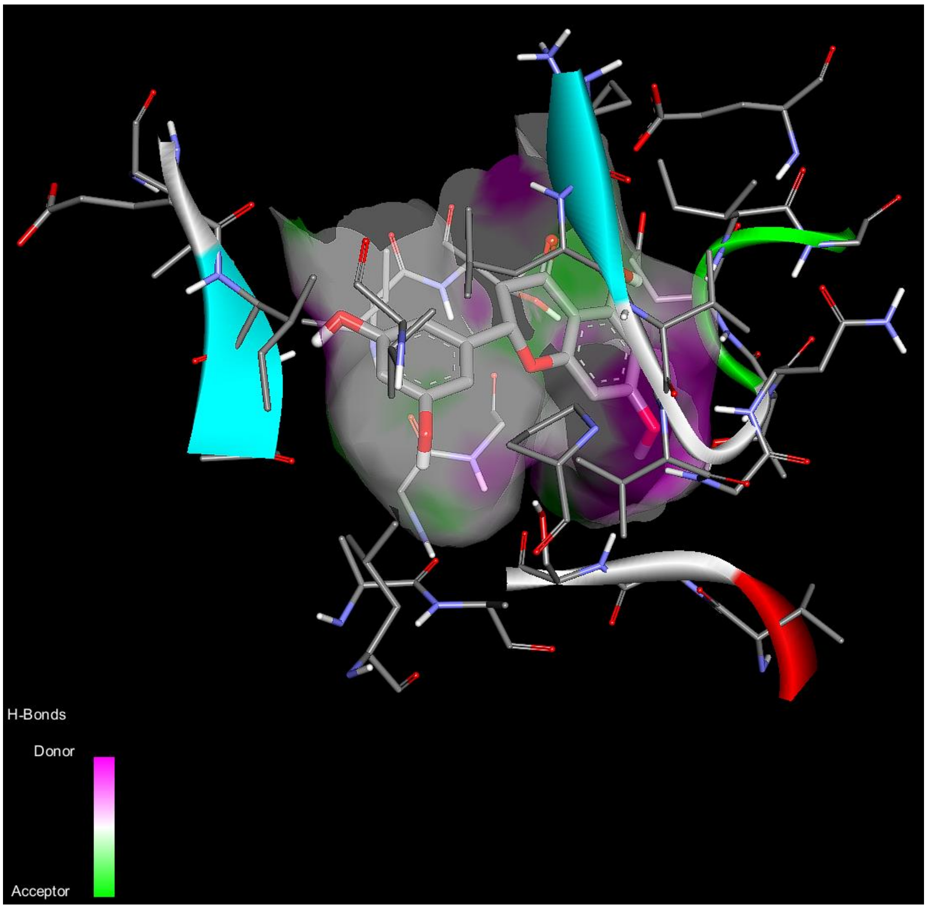

Figure S9. Docked pose 5 of blumeatin into 2E1Q.

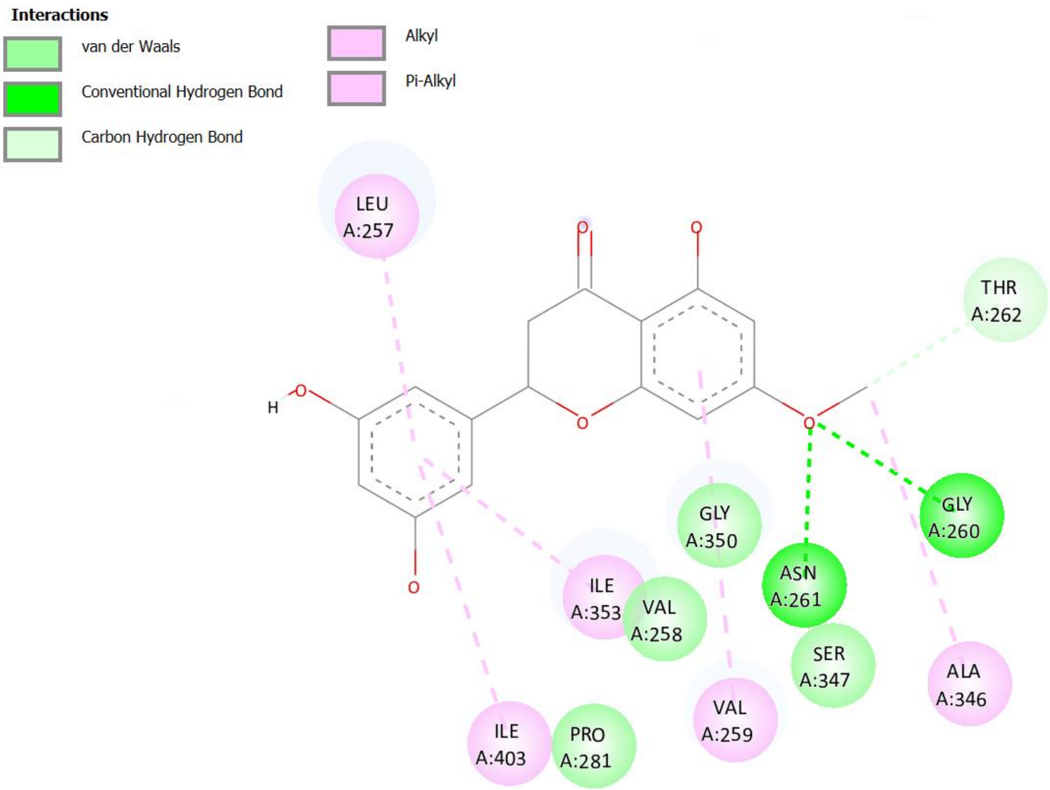

Figure S10. Surrounding residual interactions of blumeatin's docked pose 5 on 2E1Q.

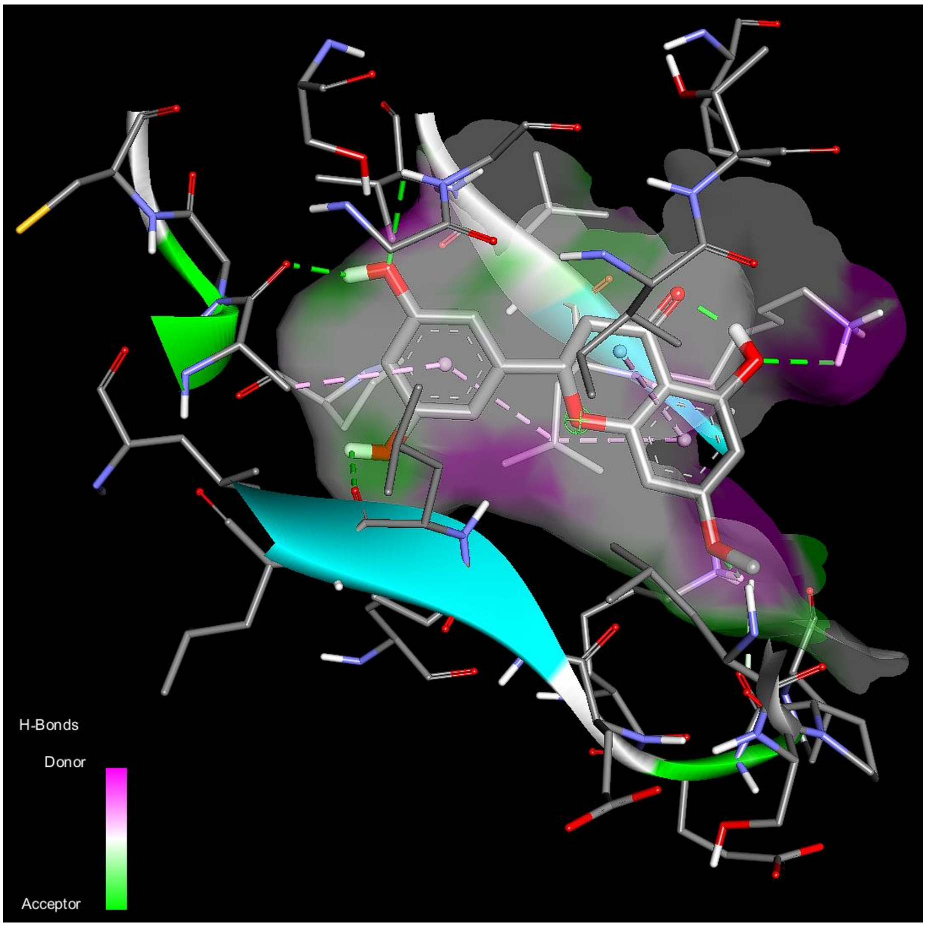

Figure S11. Docked pose 6 of blumeatin into 2E1Q.

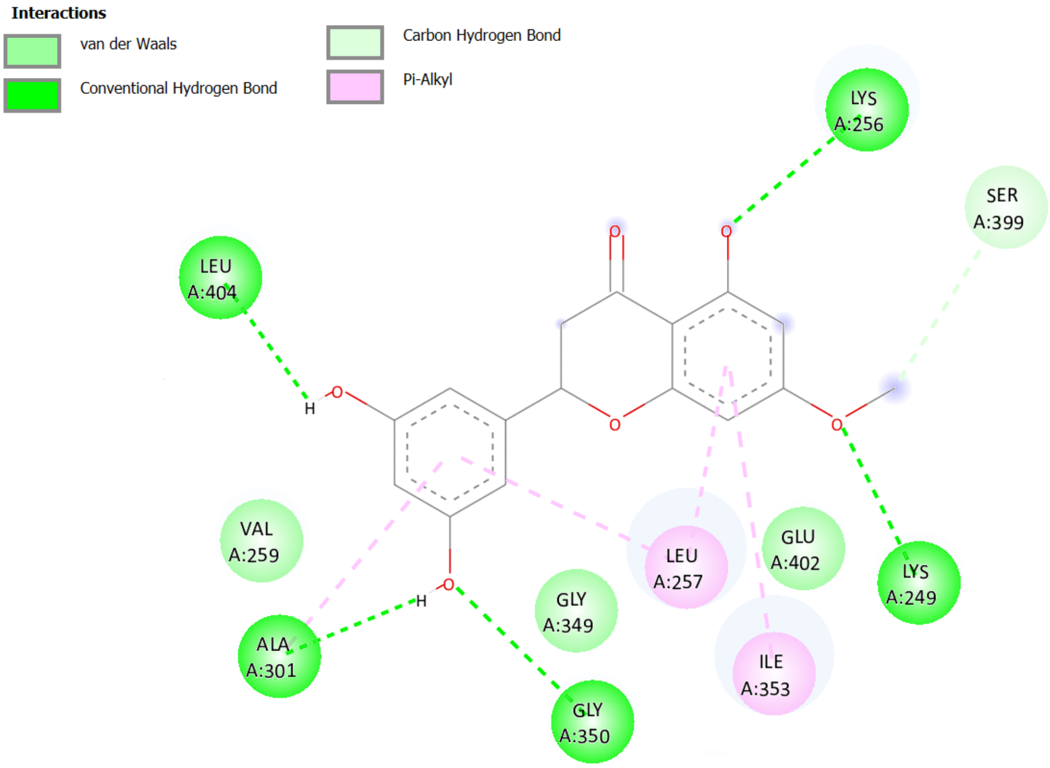

Figure S12. Surrounding residual interactions of blumeatin's docked pose 6 on 2E1Q.

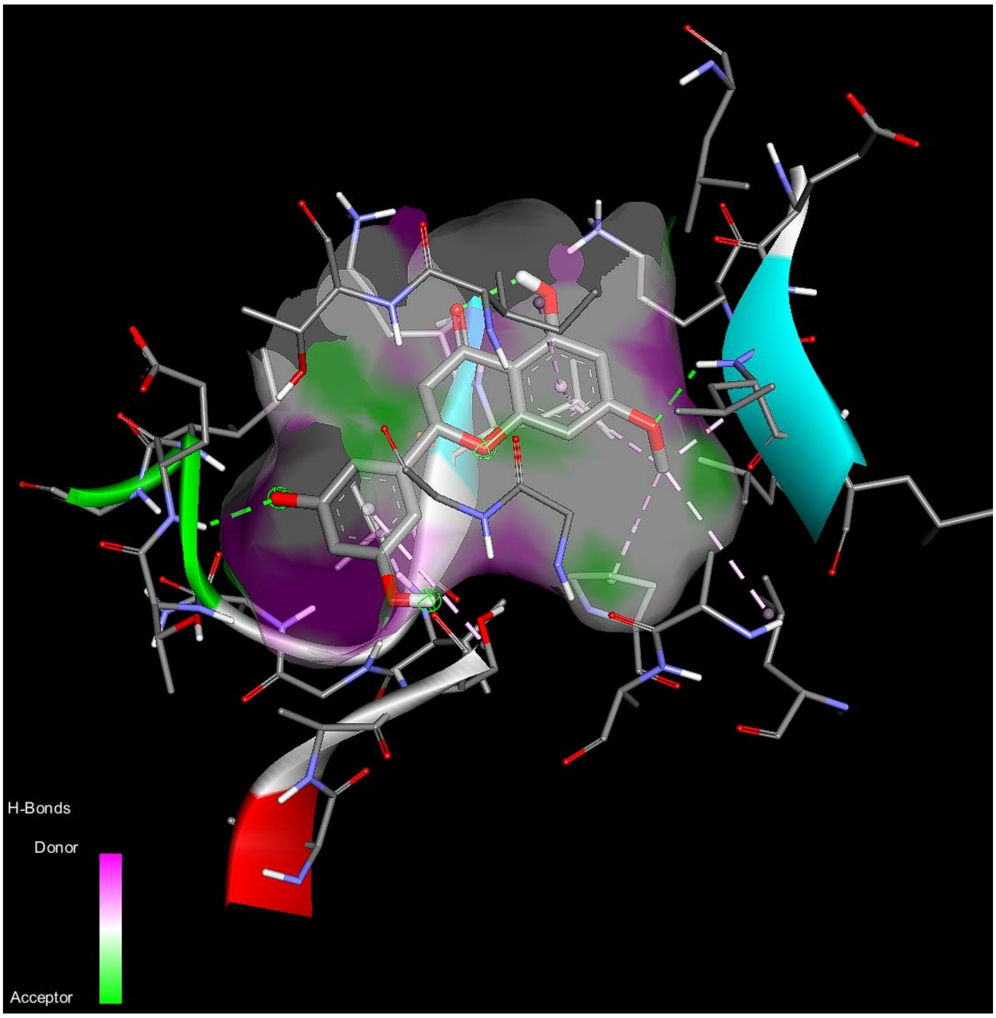

Figure S13. Docked pose 7 of blumeatin into 2E1Q.

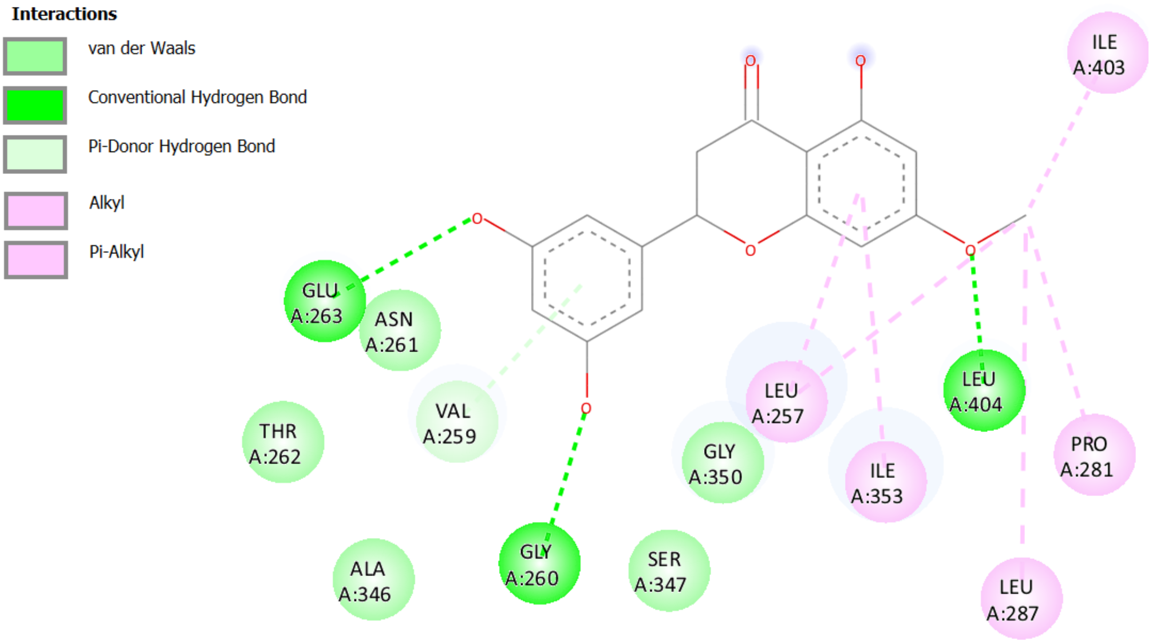

Figure S14. Surrounding residual interactions of blumeatin's docked pose 7 on 2E1Q.

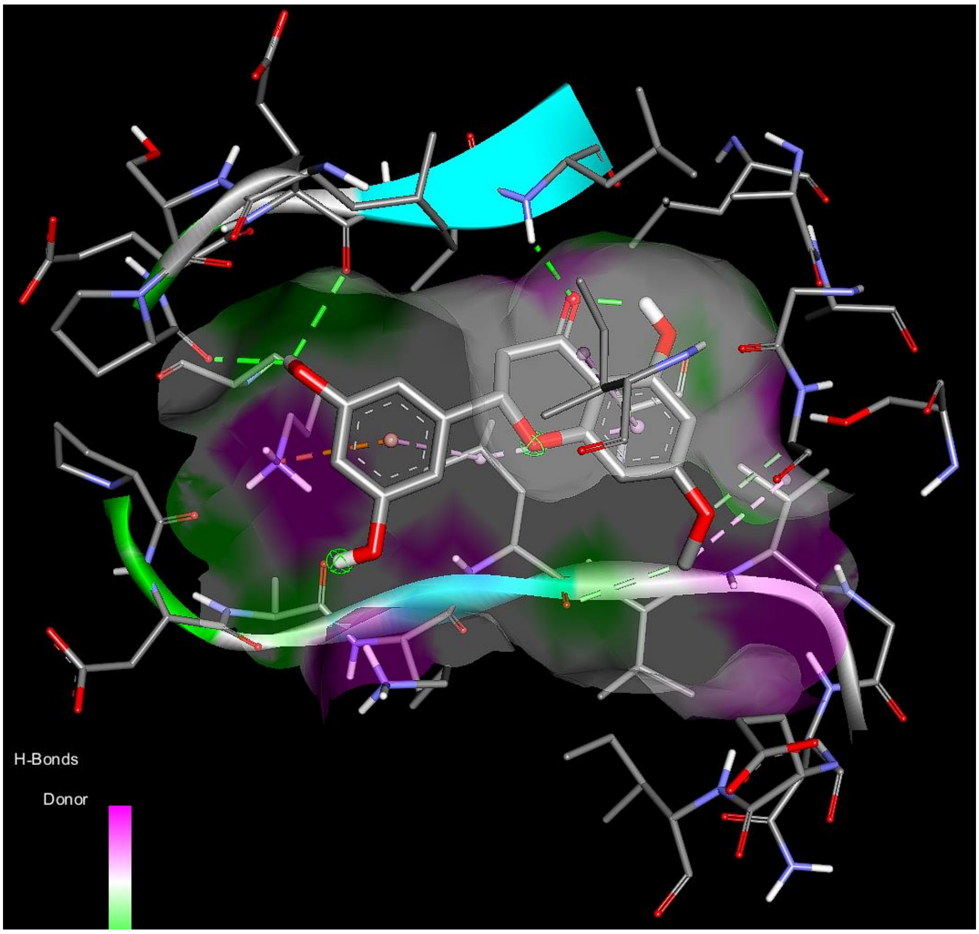

Figure S15. Docked pose 8 of blumeatin into 2E1Q.

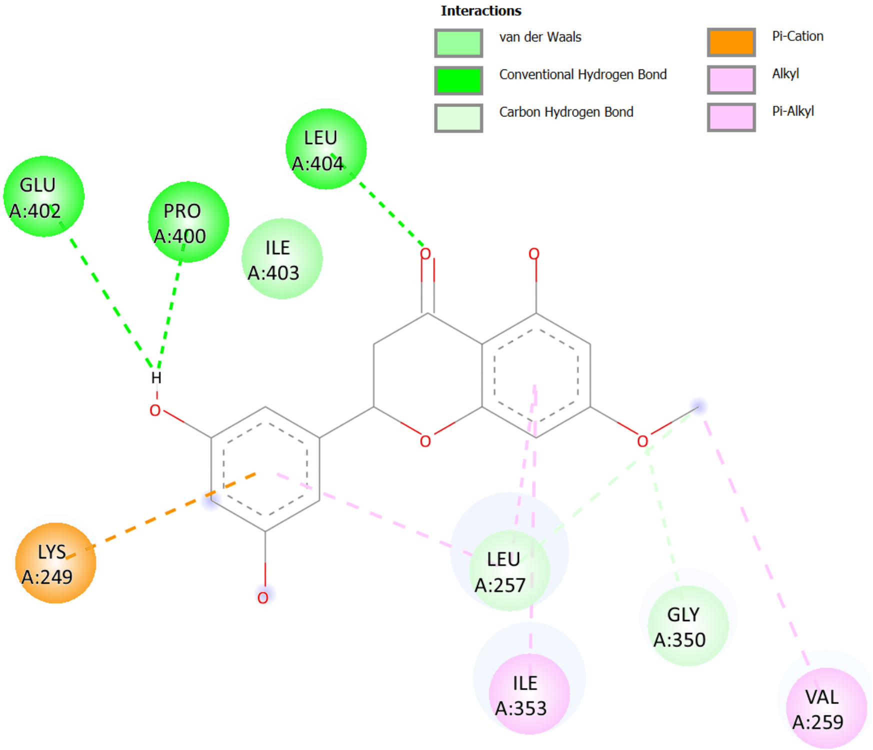

Figure S16. Surrounding residual interactions of blumeatin's docked pose 8 on 2E1Q.

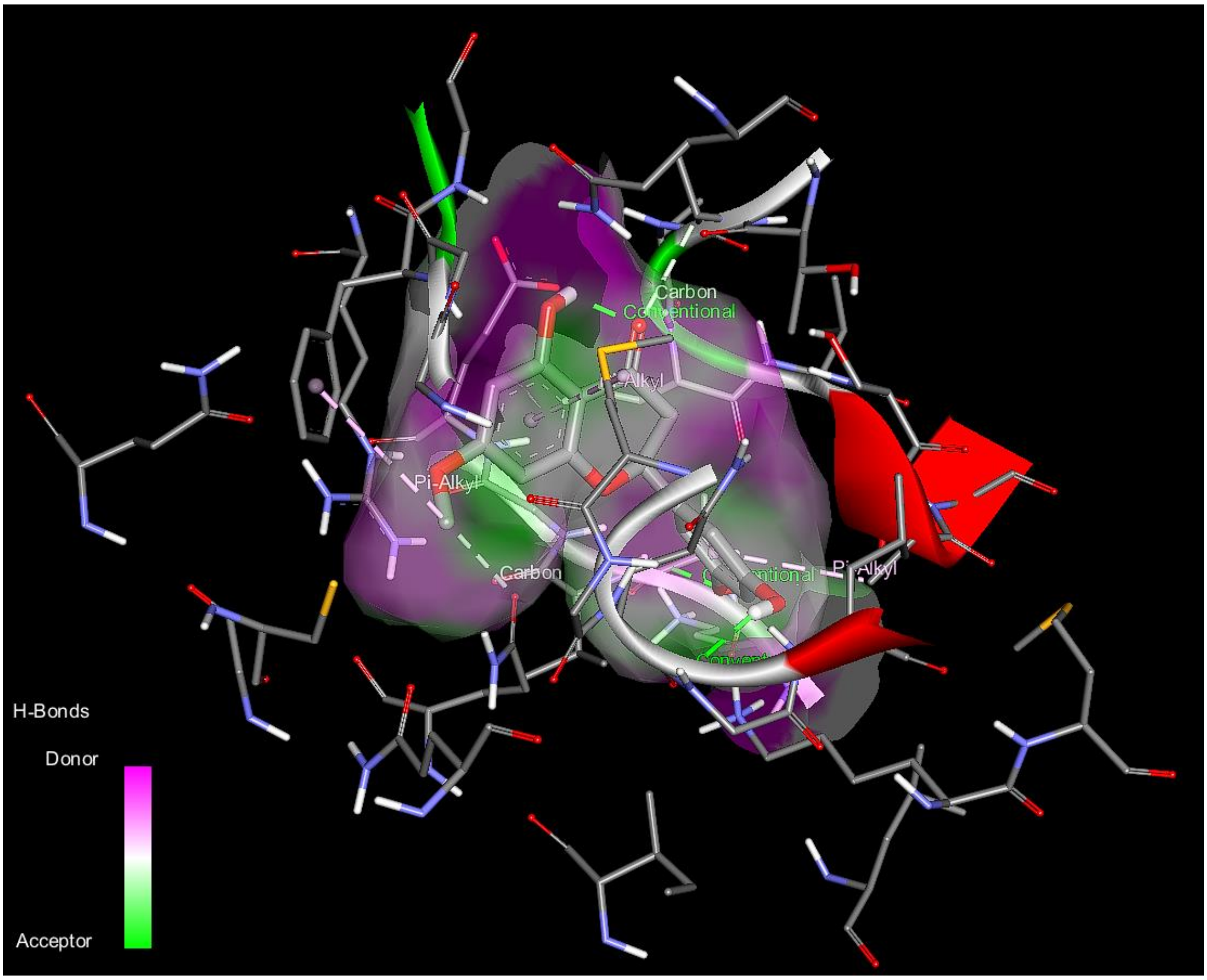

Figure S17. Docked pose 9 of blumeatin into 2E1Q.

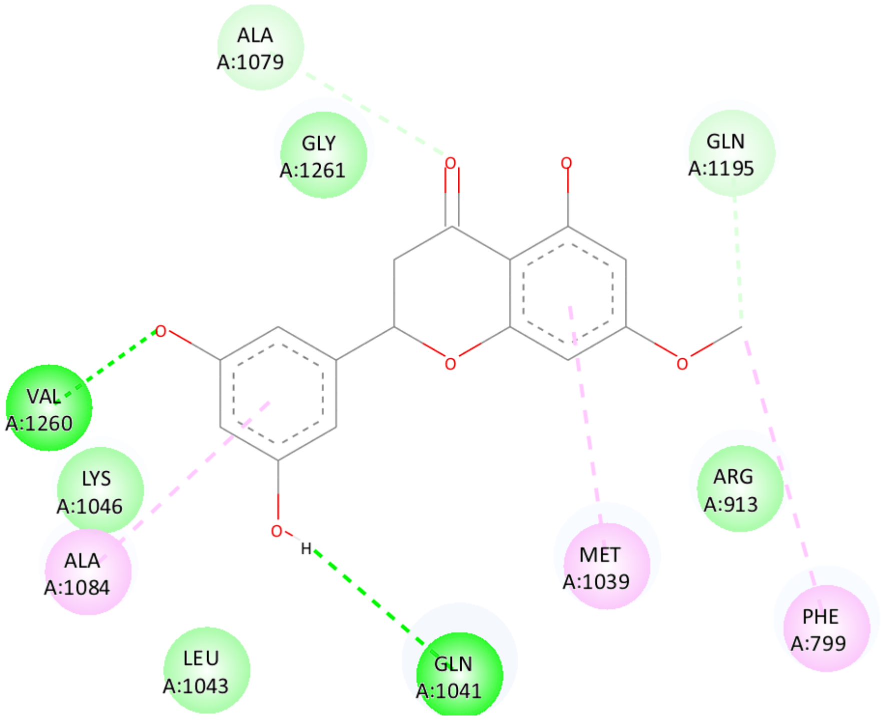

Figure S18. Surrounding residual interactions of blumeatin's docked pose 9 on 2E1Q.

**Table S1.** Distance types and distances (Å) in blumeatin.				
**6 distance types**				
**Number**	**Type**			
**1**	CC			
**2**	OC			
**3**	CH			
**4**	OO			
**5**	OH			
**6**	HH			
**597 distances in molecule**				
**Number**	**Name**	**Atom1**	**Atom2**	**Distance**
1	OO	11	14	27.070
2	OO	6	11	40.987
3	OO	6	12	46.476
4	OO	21	22	47.665
5	OO	6	14	48.091
6	OO	12	14	48.111
7	OO	6	21	49.672
8	OO	6	22	59.903
9	OO	11	12	64.744
10	OO	11	22	76.643
11	OO	11	21	78.618
12	OO	12	21	88.536
13	OO	14	21	93.885
14	OO	14	22	97.478
15	OO	12	22	104.572
16	OC	11	3	12.166
17	OC	14	10	13.540
18	OC	12	8	13.561
19	OC	6	5	13.604
20	OC	21	19	13.674
21	OC	22	17	13.684
22	OC	12	13	14.238
23	OC	6	1	14.406
24	OC	6	7	23.207
25	OC	12	7	23.283
26	OC	22	18	23.528
27	OC	21	20	23.561
28	OC	14	4	23.780
29	OC	11	2	23.833
30	OC	11	4	23.948
31	OC	6	15	23.959
32	OC	21	18	24.139

33	OC	6	2	24.210
34	OC	22	16	24.225
35	OC	12	9	24.302
36	OC	6	4	24.360
37	OC	6	20	28.010
38	OC	14	3	28.761
39	OC	6	3	28.844
40	OC	11	10	29.692
41	OC	11	1	35.956
42	OC	12	5	36.035
43	OC	6	8	36.061
44	OC	6	16	36.252
45	OC	22	19	36.293
46	OC	21	15	36.314
47	OC	11	5	36.433
48	OC	14	5	36.494
49	OC	14	8	36.545
50	OC	21	17	36.693
51	OC	12	10	36.832
52	OC	22	15	36.840
53	OC	6	9	41.406
54	OC	21	16	41.478
55	OC	14	7	41.526
56	OC	22	20	41.580
57	OC	6	19	41.671
58	OC	12	4	41.827
59	OC	11	9	43.594
60	OC	14	2	43.839
61	OC	6	17	47.585
62	OC	11	7	48.437
63	OC	11	15	48.617
64	OC	21	1	48.799
65	OC	22	1	49.270
66	OC	6	18	49.871
67	OC	14	1	50.545
68	OC	14	13	50.720
69	OC	11	8	51.250
70	OC	11	16	55.065
71	OC	21	2	55.324
72	OC	22	2	56.379
73	OC	12	3	56.536
74	OC	11	20	56.967
75	OC	12	1	59.162
76	OC	6	13	59.548
77	OC	21	5	62.221
78	OC	12	2	63.718

79	OC	14	15	65.360
80	OC	11	17	67.692
81	OC	21	7	68.151
82	OC	21	3	68.891
83	OC	11	19	69.268
84	OC	22	3	70.105
85	OC	12	15	70.183
86	OC	21	4	71.136
87	OC	11	13	71.794
88	OC	12	20	71.834
89	OC	22	5	72.198
90	OC	14	20	72.255
91	OC	11	18	74.029
92	OC	14	16	74.078
93	OC	22	4	76.917
94	OC	21	8	81.495
95	OC	12	16	81.559
96	OC	22	7	82.031
97	OC	21	10	84.141
98	OC	12	19	84.454
99	OC	14	19	85.987
100	OC	14	17	87.495
101	OC	21	9	88.722
102	OC	22	10	90.684
103	OC	12	17	92.829
104	OC	14	18	92.888
105	OC	12	18	94.218
106	OC	22	8	94.901
107	OC	22	9	98.799
108	OC	21	13	102.356
109	OC	22	13	117.860
110	OH	22	35	0.9632
111	OH	12	30	20.121
112	OH	6	23	20.548
113	OH	12	29	20.878
114	OH	6	33	25.255
115	OH	11	25	25.274
116	OH	6	26	25.307
117	OH	12	26	25.467
118	OH	22	34	25.561
119	OH	21	33	25.638
120	OH	14	27	26.098
121	OH	22	32	26.828
122	OH	6	24	26.902
123	OH	21	34	26.966
124	OH	12	27	27.233

125	OH	11	24	29.045
126	OH	6	25	33.757
127	OH	11	28	36.392
128	OH	11	23	36.985
129	OH	6	32	39.628
130	OH	22	36	47.133
131	OH	12	28	47.469
132	OH	14	24	47.901
133	OH	14	29	48.486
134	OH	14	31	48.512
135	OH	22	23	49.305
136	OH	14	25	49.547
137	OH	11	27	50.299
138	OH	14	23	50.333
139	OH	21	24	50.451
140	OH	6	27	52.232
141	OH	22	25	52.267
142	OH	14	26	52.337
143	OH	22	33	52.387
144	OH	11	32	52.661
145	OH	21	35	55.495
146	OH	6	28	55.645
147	OH	11	33	56.348
148	OH	21	23	57.005
149	OH	11	26	57.705
150	OH	6	36	58.569
151	OH	21	25	58.589
152	OH	22	24	59.373
153	OH	12	23	60.084
154	OH	6	35	60.330
155	OH	6	34	60.445
156	OH	14	30	61.592
157	OH	6	29	62.454
158	OH	6	31	62.556
159	OH	21	26	64.246
160	OH	12	33	65.189
161	OH	12	24	65.605
162	OH	6	30	66.547
163	OH	14	33	68.756
164	OH	11	31	70.744
165	OH	11	29	71.024
166	OH	14	32	71.919
167	OH	11	35	73.702
168	OH	12	25	73.819
169	OH	22	26	80.988
170	OH	11	30	82.155

171	OH	12	32	82.963
172	OH	11	34	84.325
173	OH	11	36	86.722
174	OH	14	35	94.622
175	OH	12	36	97.695
176	OH	21	27	99.259
177	OH	21	28	101.978
178	OH	14	36	102.959
179	OH	14	34	103.645
180	OH	12	34	104.116
181	OH	12	35	104.242
182	OH	21	31	104.519
183	OH	22	28	106.704
184	OH	21	30	106.780
185	OH	21	29	107.945
186	OH	22	27	109.274
187	OH	22	29	119.212
188	OH	22	31	122.032
189	OH	22	30	124.081
190	CC	5	7	13.865
191	CC	15	20	13.912
192	CC	17	18	13.939
193	CC	9	10	13.960
194	CC	15	16	13.987
195	CC	4	10	14.152
196	CC	4	5	14.207
197	CC	3	4	14.765
198	CC	1	15	15.116
199	CC	1	2	15.259
200	CC	1	5	23.886
201	CC	8	13	23.990
202	CC	17	19	24.020
203	CC	15	19	24.049
204	CC	15	17	24.130
205	CC	8	10	24.162
206	CC	16	20	24.209
207	CC	16	18	24.224
208	CC	7	9	24.255
209	CC	18	20	24.284
210	CC	4	9	24.531
211	CC	4	7	24.599
212	CC	1	16	25.054
213	CC	1	3	25.217
214	CC	2	4	25.242
215	CC	2	15	25.366
216	CC	3	10	25.481

217	CC	16	19	27.813
218	CC	5	9	27.825
219	CC	17	20	27.906
220	CC	15	18	27.950
221	CC	7	10	27.986
222	CC	1	4	28.108
223	CC	2	5	28.167
224	CC	4	8	28.288
225	CC	9	13	28.315
226	CC	2	20	33.331
227	CC	2	16	33.679
228	CC	1	7	36.107
229	CC	7	13	36.468
230	CC	5	15	36.641
231	CC	1	17	37.884
232	CC	3	7	37.931
233	CC	1	19	37.996
234	CC	3	9	38.227
235	CC	2	10	38.624
236	CC	3	15	38.826
237	CC	5	20	41.120
238	CC	2	7	41.927
239	CC	1	10	42.072
240	CC	10	13	42.264
241	CC	4	15	42.756
242	CC	3	8	43.014
243	CC	1	18	43.053
244	CC	2	19	45.482
245	CC	2	17	45.718
246	CC	3	20	46.707
247	CC	7	15	47.016
248	CC	3	16	47.062
249	CC	1	8	47.657
250	CC	5	13	47.964
251	CC	5	16	48.149
252	CC	4	20	49.166
253	CC	7	20	49.437
254	CC	2	9	49.621
255	CC	1	9	50.100
256	CC	2	18	50.681
257	CC	4	13	50.815
258	CC	2	8	50.871
259	CC	4	16	52.833
260	CC	5	19	54.945
261	CC	10	15	56.778
262	CC	7	16	58.669

263	CC	3	19	59.711
264	CC	8	15	59.910
265	CC	3	17	59.967
266	CC	5	17	60.360
267	CC	7	19	62.573
268	CC	10	20	62.765
269	CC	8	20	62.839
270	CC	4	19	63.044
271	CC	5	18	63.308
272	CC	9	15	64.055
273	CC	3	13	65.363
274	CC	3	18	65.464
275	CC	4	17	65.918
276	CC	10	16	66.671
277	CC	9	20	68.598
278	CC	7	17	70.065
279	CC	4	18	70.374
280	CC	8	16	71.236
281	CC	1	13	71.519
282	CC	7	18	71.842
283	CC	2	13	74.560
284	CC	9	16	74.718
285	CC	8	19	76.177
286	CC	10	19	76.710
287	CC	10	17	79.907
288	CC	9	19	82.451
289	CC	8	17	83.205
290	CC	13	15	83.398
291	CC	10	18	84.399
292	CC	8	18	85.473
293	CC	13	20	85.492
294	CC	9	17	87.575
295	CC	9	18	91.086
296	CC	13	16	94.588
297	CC	13	19	98.330
298	CC	13	17	106.295
299	CC	13	18	108.081
300	CH	20	33	10.812
301	CH	9	27	10.826
302	CH	18	34	10.846
303	CH	16	32	10.861
304	CH	13	30	10.883
305	CH	2	25	10.909
306	CH	13	29	10.947
307	CH	2	24	10.966
308	CH	1	23	10.983

309	CH	10	28	19.029
310	CH	19	36	19.210
311	CH	3	25	21.390
312	CH	10	27	21.414
313	CH	3	24	21.439
314	CH	19	33	21.457
315	CH	1	24	21.529
316	CH	17	32	21.544
317	CH	15	33	21.600
318	CH	15	32	21.615
319	CH	19	34	21.685
320	CH	1	25	21.730
321	CH	9	28	23.904
322	CH	18	36	24.479
323	CH	16	35	24.580
324	CH	13	27	25.404
325	CH	16	23	26.165
326	CH	5	23	26.950
327	CH	1	32	27.040
328	CH	8	29	27.298
329	CH	8	31	27.327
330	CH	1	33	27.525
331	CH	3	23	27.573
332	CH	15	24	27.671
333	CH	15	25	28.141
334	CH	9	29	28.257
335	CH	9	31	28.318
336	CH	4	23	29.808
337	CH	4	24	29.838
338	CH	20	24	31.016
339	CH	5	24	31.464
340	CH	18	35	31.647
341	CH	20	36	31.689
342	CH	4	28	31.834
343	CH	16	25	32.081
344	CH	8	30	32.626
345	CH	20	23	33.739
346	CH	16	34	33.979
347	CH	18	33	34.006
348	CH	18	32	34.043
349	CH	20	32	34.061
350	CH	16	33	34.081
351	CH	7	27	34.129
352	CH	2	33	34.168
353	CH	20	34	34.188
354	CH	4	27	34.229

355	CH	4	26	34.353
356	CH	2	32	34.585
357	CH	5	33	36.954
358	CH	20	25	37.645
359	CH	16	24	37.788
360	CH	8	28	37.812
361	CH	7	23	38.247
362	CH	3	28	38.299
363	CH	5	25	38.338
364	CH	15	35	38.450
365	CH	5	27	38.650
366	CH	19	32	38.674
367	CH	17	33	38.717
368	CH	15	34	38.795
369	CH	9	30	38.921
370	CH	1	26	39.230
371	CH	13	26	39.691
372	CH	17	23	39.948
373	CH	7	29	40.199
374	CH	7	31	40.294
375	CH	10	29	41.592
376	CH	10	31	41.646
377	CH	10	24	42.503
378	CH	10	23	42.513
379	CH	19	24	42.665
380	CH	5	28	43.152
381	CH	19	35	43.192
382	CH	15	36	43.230
383	CH	7	30	43.385
384	CH	17	25	43.467
385	CH	7	33	43.783
386	CH	7	24	44.181
387	CH	19	23	45.211
388	CH	3	33	45.553
389	CH	7	28	45.634
390	CH	16	36	45.900
391	CH	4	33	45.924
392	CH	3	32	45.994
393	CH	3	26	46.497
394	CH	10	25	46.541
395	CH	3	27	46.860
396	CH	15	26	47.271
397	CH	13	28	47.320
398	CH	19	25	47.718
399	CH	2	26	47.737
400	CH	17	24	47.752

401	CH	18	23	47.812
402	CH	1	35	48.657
403	CH	8	23	48.722
404	CH	5	32	49.668
405	CH	18	24	49.873
406	CH	18	25	50.236
407	CH	5	29	50.319
408	CH	5	31	50.412
409	CH	9	23	50.493
410	CH	4	29	51.420
411	CH	4	31	51.441
412	CH	7	25	52.175
413	CH	4	32	52.485
414	CH	9	24	52.682
415	CH	10	30	52.809
416	CH	2	28	53.264
417	CH	8	24	53.278
418	CH	1	34	53.895
419	CH	2	35	55.348
420	CH	5	30	56.047
421	CH	1	36	56.737
422	CH	8	33	56.821
423	CH	9	25	58.533
424	CH	10	33	58.607
425	CH	16	26	58.871
426	CH	2	27	59.349
427	CH	19	26	59.785
428	CH	4	30	60.484
429	CH	7	32	60.640
430	CH	1	27	60.652
431	CH	8	25	60.797
432	CH	2	34	61.137
433	CH	9	33	63.223
434	CH	2	36	63.283
435	CH	3	31	65.319
436	CH	3	29	65.621
437	CH	10	32	65.644
438	CH	3	35	67.995
439	CH	17	26	68.922
440	CH	18	26	69.418
441	CH	5	36	71.389
442	CH	5	35	71.671
443	CH	13	23	71.876
444	CH	8	32	72.189
445	CH	1	29	73.111
446	CH	5	34	73.940

447	CH	15	28	74.212
448	CH	1	31	74.283
449	CH	9	32	74.379
450	CH	15	27	74.781
451	CH	3	30	75.165
452	CH	4	35	75.246
453	CH	2	31	75.304
454	CH	2	29	75.967
455	CH	3	34	76.064
456	CH	13	24	76.566
457	CH	3	36	77.342
458	CH	7	36	77.360
459	CH	13	33	78.714
460	CH	1	30	79.229
461	CH	20	27	79.360
462	CH	4	36	80.189
463	CH	20	28	80.688
464	CH	4	34	81.170
465	CH	7	35	81.929
466	CH	7	34	82.056
467	CH	16	28	83.182
468	CH	2	30	83.434
469	CH	13	25	84.323
470	CH	16	27	85.234
471	CH	15	29	85.840
472	CH	15	31	86.474
473	CH	20	31	87.997
474	CH	10	35	88.589
475	CH	20	29	89.579
476	CH	15	30	90.152
477	CH	8	36	90.826
478	CH	20	30	91.359
479	CH	19	27	93.213
480	CH	10	36	93.386
481	CH	8	35	94.176
482	CH	19	28	94.530
483	CH	10	34	95.189
484	CH	13	32	95.313
485	CH	8	34	95.789
486	CH	16	29	96.030
487	CH	17	28	96.636
488	CH	9	35	97.177
489	CH	9	36	98.138
490	CH	17	27	98.227
491	CH	16	31	98.287
492	CH	19	31	101.033

493	CH	16	30	101.376
494	CH	9	34	101.739
495	CH	18	28	101.826
496	CH	18	27	101.894
497	CH	19	29	102.704
498	CH	19	30	103.591
499	CH	17	29	108.354
500	CH	17	31	110.056
501	CH	18	31	111.396
502	CH	18	29	111.475
503	CH	13	36	111.599
504	CH	17	30	112.485
505	CH	18	30	113.589
506	CH	13	35	117.071
507	CH	13	34	118.094
508	HH	24	25	17.673
509	HH	30	31	17.809
510	HH	29	31	17.912
511	HH	27	28	21.919
512	HH	32	35	23.018
513	HH	34	36	23.156
514	HH	27	29	23.407
515	HH	27	31	23.490
516	HH	23	32	23.972
517	HH	23	25	25.082
518	HH	24	33	29.758
519	HH	23	24	30.579
520	HH	25	32	31.138
521	HH	34	35	35.002
522	HH	33	36	35.078
523	HH	27	30	36.227
524	HH	23	33	37.313
525	HH	25	33	40.897
526	HH	24	32	41.265
527	HH	23	26	41.727
528	HH	26	33	41.874
529	HH	32	34	42.895
530	HH	33	34	43.047
531	HH	32	33	43.093
532	HH	26	27	43.108
533	HH	28	31	44.158
534	HH	26	30	44.208
535	HH	28	29	44.263
536	HH	26	29	44.614
537	HH	26	31	44.789
538	HH	23	35	46.785

539	HH	24	26	49.298
540	HH	25	35	50.261
541	HH	35	36	55.871
542	HH	26	28	56.276
543	HH	32	36	56.609
544	HH	33	35	56.695
545	HH	24	28	56.912
546	HH	25	26	57.908
547	HH	28	30	58.125
548	HH	23	34	58.415
549	HH	24	36	58.683
550	HH	23	28	58.921
551	HH	25	28	59.180
552	HH	24	34	59.846
553	HH	24	35	59.931
554	HH	25	34	60.124
555	HH	23	27	60.588
556	HH	26	32	61.976
557	HH	24	27	62.316
558	HH	23	36	64.287
559	HH	25	36	65.500
560	HH	25	27	67.786
561	HH	23	29	71.934
562	HH	26	36	73.149
563	HH	27	33	73.776
564	HH	23	31	75.698
565	HH	24	31	76.243
566	HH	28	33	76.595
567	HH	26	34	79.053
568	HH	24	29	79.345
569	HH	23	30	79.664
570	HH	31	33	80.540
571	HH	28	32	81.002
572	HH	26	35	81.720
573	HH	29	33	83.793
574	HH	30	33	84.304
575	HH	27	32	84.461
576	HH	25	29	84.984
577	HH	25	31	85.108
578	HH	24	30	85.130
579	HH	25	30	93.422
580	HH	29	32	95.515
581	HH	31	32	99.342
582	HH	30	32	102.552
583	HH	28	35	103.809
584	HH	27	35	107.359

585	HH	27	36	108.720
586	HH	28	36	111.199
587	HH	27	34	112.554
588	HH	28	34	112.602
589	HH	31	36	113.964
590	HH	30	36	115.801
591	HH	29	36	117.132
592	HH	29	35	117.547
593	HH	31	35	121.449
594	HH	31	34	121.475
595	HH	29	34	121.723
596	HH	30	34	123.238
597	HH	30	35	123.672

Table S2. Optimized geometric structure of blumeatin.*

R(1-2)	1.526	R(19-20)	1.396	A(8-7-26)	120.5	A(19-21-36)	109.9	D(3,4,10,9)	–174.4	D(15,16,17,18)	0.4
R(1-6)	1.441	R(19-21)	1.367	A(7-8-9)	120.5	D(6,1,2,3)	–56.9	D(3,4,10,14)	4.8	D(15,16,17,22)	179.9
R(1-15)	1.512	R(20-33)	1.081	A(7-8-12)	115.7	D(6,1,2,24)	62.9	D(5,4,10,9)	1.3	D(32,16,17,18)	–179.1
R(1-23)	1.098	R(21-36)	0.963	A(9-8-12)	123.8	D(6,1,2,25)	–178.1	D(5,4,10,14)	–179.3	D(32,16,17,22)	0.3
R(2-3)	1.526	R(22-35)	0.963	A(8-9-10)	119.7	D(15,1,2,3)	–178.1	D(4,5,6,1)	–20.8	D(16,17,18,19)	0.0
R(2-24)	1.097	A(2-1-6)	109.4	A(8-9-27)	121.3	D(15,1,2,24)	–58.1	D(7,5,6,1)	159.8	D(16,17,18,34)	179.4
R(2-25)	1.091	A(2-1-15)	113.3	A(8-12-13)	119.3	D(15,1,2,25)	60.7	D(4,5,7,8)	0.4	D(22,17,18,19)	–179.5
R(3-4)	1.477	A(2-1-23)	109.2	A(10-9-27)	119.0	D(23,1,2,3)	60.1	D(4,5,7,26)	179.5	D(22,17,18,34)	-0.02
R(3-11)	1.217	A(1-2-3)	111.4	A(9-10-14)	120.1	D(23,1,2,24)	–179.8	D(6,5,7,8)	179.6	D(16,17,22,35)	0.9
R(4-5)	1.421	A(1-2-24)	109.3	A(10-14-28)	109.2	D(23,1,2,25)	–61.0	D(6,5,7,26)	–1.1	D(18,17,22,35)	–179.5
R(4-10)	1.415	A(1-2-25)	111.2	A(12-13-29)	111.3	D(2,1,6,5)	51.2	D(5,7,8,9)	0.8	D(17,18,19,20)	-0.4
R(5-6)	1.360	A(6-1-15)	108.5	A(12-13-30)	105.7	D(15,1,6,5)	175.2	D(5,7,8,12)	–179.9	D(17,18,19,21)	179.6
R(5-7)	1.387	A(6-1-23)	107.3	A(12-13-31)	111.3	D(23,1,6,5)	–67.0	D(26,7,8,9)	–178.3	D(34,18,19,20)	–179.9
R(7-8)	1.394	A(1-6-5)	117.0	A(29-13-30)	109.3	D(2,1,15,16)	–91.7	D(26,7,8,12)	0.8	D(34,18,19,21)	0.1
R(7-26)	1.081	A(15-1-23)	109.1	A(29-13-31)	109.8	D(2,1,15,20)	85.3	D(7,8,9,10)	–0.9	D(18,19,20,15)	0.4
R(8-9)	1.399	A(1-15-16)	118.8	A(30-13-31)	109.3	D(6,1,15,16)	146.5	D(7,8,9,27)	179.1	D(18,19,20,33)	179.2
R(8-12)	1.356	A(1-15-20)	120.8	A(16-15-20)	120.4	D(6,1,15,20)	–36.2	D(12,8,9,10)	179.9	D(21,19,20,15)	–179.6
R(9-10)	1.396	A(3-2-24)	108.5	A(15-16-17)	119.6	D(23,1,15,16)	30.0	D(12,8,9,27)	-0.0	D(21,19,20,33)	-0.8
R(9-27)	1.083	A(3-2-25)	108.5	A(15-16-32)	120.4	D(23,1,15,20)	–152.8	D(7,8,12,13)	–179.2	D(18,19,21,36)	–0.4
R(10-14)	1.354	A(2-3-4)	114.4	A(15-20-19)	119.3	D(1,2,3,4)	33.6	D(9,8,12,13)	-0.0	D(20,19,21,36)	179.6
R(12-13)	1.424	A(2-3-11)	120.3	A(15-20-33)	121.2	D(1,2,3,11)	–148.0	D(8,9,10,4)	-0.2	D(3,4,5,6)	–4.71
R(13-29)	1.095	A(24-2-25)	107.8	A(17-16-32)	120.1	D(24,2,3,4)	–86.7	D(8,9,10,14)	–179.4	D(3,4,5,7)	174.4

R(13-30)	1.088	A(4-3-11)	125.3	A(16-17-18)	120.6	D(24,2,3,11)	91.5	D(27,9,10,4)	179.7	D(10,4,5,6)	179.3
R(13-31)	1.095	A(3-4-5)	119.7	A(16-17-22)	122.5	D(25,2,3,4)	156.3	D(27,9,10,14)	0.4	D(8,12,13,31)	−61.6
R(14-28)	0.964	A(3-4-10)	123.6	A(18-17-22)	116.8	D(25,2,3,11)	−25.2	D(4,10,14,28)	−177.5		
R(15-16)	1.399	A(5-4-10)	116.6	A(17-18-19)	119.1	D(2,3,4,5)	−3.2	D(9,10,14,28)	1.7		
R(15-20)	1.391	A(4-5-6)	122.3	A(17-18-34)	119.3	D(2,3,4,10)	172.3	D(8,12,13,29)	61.2		
R(16-17)	1.394	A(4-5-7)	122.4	A(17-22-35)	109.7	D(11,3,4,5)	178.4	D(8,12,13,30)	179.8		
R(16-32)	1.086	A(4-10-9)	121.5	A(19-18-34)	121.7	D(11,3,4,10)	−5.9	D(20,15,16,32)	179.1		
R(17-18)	1.394	A(4-10-14)	118.3	A(18-19-20)	121.1	D(1,15,16,17)	176.6	D(1,15,20,19)	−177.0		
R(17-22)	1.368	A(6-5-7)	115.3	A(18-19-21)	122.0	D(1,15,16,32)	−3.7	D(1,15,20,33)	4.1		
R(18-19)	1.393	A(5-7-8)	119.2	A(20-19-21)	117.0	D(20,15,16,17)	−0.4	D(16,15,20,19)	0.0		
R(18-34)	1.085	A(5-7-26)	120.3	A(19-20-33)	119.5	D(10,4,5,7)	−1.4	D(16,15,20,33)	−178.7		

*R: bond lengths (Å); A: angles (˚); D: dihedral angles (˚).

Table S3. Normal modes used in the determination of vibrational modes and their contribution.

! Normal Mode 1 !

! Name	Definition	Value	Relative Weight (%)	!
! A43	A(1,15,16)	-0.0033	0.4	!
! A44	A(1,15,20)	0.0034	0.4	!
! D1	D(6,1,2,3)	-0.0027	0.3	!
! D2	D(6,1,2,24)	-0.0027	0.3	!
! D4	D(15,1,2,3)	-0.0044	0.5	!
! D5	D(15,1,2,24)	-0.0044	0.5	!
! D6	D(15,1,2,25)	-0.0039	0.5	!
! D10	D(2,1,6,5)	-0.0069	0.9	!
! D11	D(15,1,6,5)	-0.0076	0.9	!
! D12	D(23,1,6,5)	-0.0068	0.8	!
! D13	D(2,1,15,16)	0.0889	11.1	!
! D14	D(2,1,15,20)	0.0891	11.1	!
! D15	D(6,1,15,16)	0.0881	11.0	!
! D16	D(6,1,15,20)	0.0883	11.0	!
! D17	D(23,1,15,16)	0.087	10.8	!
! D18	D(23,1,15,20)	0.0872	10.8	!
! D19	D(1,2,3,4)	0.0092	1.1	!
! D20	D(1,2,3,11)	0.0093	1.2	!
! D21	D(24,2,3,4)	0.0103	1.3	!
! D22	D(24,2,3,11)	0.0103	1.3	!
! D23	D(25,2,3,4)	0.009	1.1	!
! D24	D(25,2,3,11)	0.009	1.1	!
! D25	D(2,3,4,5)	-0.0075	0.9	!
! D26	D(2,3,4,10)	-0.0094	1.2	!
! D27	D(11,3,4,5)	-0.0076	0.9	!
! D28	D(11,3,4,10)	-0.0094	1.2	!
! D30	D(3,4,5,7)	-0.0026	0.3	!
! D33	D(3,4,10,9)	0.0032	0.4	!
! D37	D(4,5,6,1)	0.0097	1.2	!
! D38	D(7,5,6,1)	0.0098	1.2	!
! D51	D(7,8,12,13)	-0.0028	0.4	!
! D57	D(4,10,14,28)	0.0029	0.4	!
! D59	D(8,12,13,29)	0.004	0.5	!
! D60	D(8,12,13,30)	0.004	0.5	!
! D61	D(8,12,13,31)	0.004	0.5	!

! Normal Mode 2 !

! Name	Definition	Value	Relative Weight (%)	!
! A1	A(2,1,6)	0.0074	0.5	!

```
! A2    A(2,1,15)              -0.0083              0.5                !
! A4    A(6,1,15)              -0.0073              0.4                !
! A22   A(1,6,5)                0.0163              1.0                !
! A43   A(1,15,16)              0.0071              0.4                !
! A44   A(1,15,20)             -0.0076              0.5                !
! D1    D(6,1,2,3)              0.018               1.1                !
! D2    D(6,1,2,24)             0.0175              1.1                !
! D3    D(6,1,2,25)             0.0152              0.9                !
! D4    D(15,1,2,3)             0.0276              1.7                !
! D5    D(15,1,2,24)            0.0271              1.6                !
! D6    D(15,1,2,25)            0.0248              1.5                !
! D7    D(23,1,2,3)             0.0253              1.5                !
! D8    D(23,1,2,24)            0.0248              1.5                !
! D9    D(23,1,2,25)            0.0225              1.4                !
! D10   D(2,1,6,5)             -0.0588              3.6                !
! D11   D(15,1,6,5)            -0.069               4.2                !
! D12   D(23,1,6,5)            -0.0665              4.0                !
! D13   D(2,1,15,16)           -0.039               2.4                !
! D14   D(2,1,15,20)           -0.0464              2.8                !
! D15   D(6,1,15,16)           -0.0378              2.3                !
! D16   D(6,1,15,20)           -0.0452              2.8                !
! D17   D(23,1,15,16)          -0.038               2.3                !
! D18   D(23,1,15,20)          -0.0454              2.8                !
! D19   D(1,2,3,4)              0.0398              2.4                !
! D20   D(1,2,3,11)             0.0388              2.4                !
! D21   D(24,2,3,4)             0.0397              2.4                !
! D22   D(24,2,3,11)            0.0387              2.4                !
! D23   D(25,2,3,4)             0.0392              2.4                !
! D24   D(25,2,3,11)            0.0382              2.3                !
! D25   D(2,3,4,5)             -0.0586              3.6                !
! D26   D(2,3,4,10)            -0.0583              3.5                !
! D27   D(11,3,4,5)            -0.0576              3.5                !
! D28   D(11,3,4,10)           -0.0572              3.5                !
! D29   D(3,4,5,6)              0.0204              1.2                !
! D30   D(3,4,5,7)              0.0165              1.0                !
! D31   D(10,4,5,6)             0.0201              1.2                !
! D32   D(10,4,5,7)             0.0163              1.0                !
! D33   D(3,4,10,9)            -0.0091              0.6                !
! D34   D(3,4,10,14)           -0.0156              0.9                !
! D35   D(5,4,10,9)            -0.0088              0.5                !
! D36   D(5,4,10,14)           -0.0153              0.9                !
! D37   D(4,5,6,1)              0.0395              2.4                !
! D38   D(7,5,6,1)              0.043               2.6                !
! D39   D(4,5,7,8)             -0.0127              0.8                !
! D41   D(6,5,7,8)             -0.0162              1.0                !
! D42   D(6,5,7,26)            -0.0064              0.4                !
! D44   D(5,7,8,12)             0.0053              0.3                !
! D45   D(26,7,8,9)            -0.0085              0.5                !
! D47   D(7,8,9,10)             0.0058              0.4                !
! D51   D(7,8,12,13)           -0.0213              1.3                !
```

D52	D(9,8,12,13)	-0.0171	1.0
D56	D(27,9,10,14)	0.0057	0.3
D57	D(4,10,14,28)	0.0076	0.5
D59	D(8,12,13,29)	0.0056	0.3
D60	D(8,12,13,30)	0.0055	0.3
D61	D(8,12,13,31)	0.0056	0.3
D62	D(1,15,16,17)	-0.0082	0.5
D63	D(1,15,16,32)	-0.0062	0.4
D66	D(1,15,20,19)	0.0088	0.5
D67	D(1,15,20,33)	0.0069	0.4

! Normal Mode 3 !

Name	Definition	Value	Relative Weight (%)
R5	R(2,3)	-0.0066	0.6
R12	R(5,6)	0.005	0.4
A2	A(2,1,15)	-0.0257	2.2
A4	A(6,1,15)	0.0214	1.8
A17	A(3,4,10)	-0.005	0.4
A21	A(6,5,7)	0.0037	0.3
A22	A(1,6,5)	-0.0065	0.5
A43	A(1,15,16)	-0.0109	0.9
A44	A(1,15,20)	0.0085	0.7
D3	D(6,1,2,25)	-0.0052	0.4
D4	D(15,1,2,3)	-0.0145	1.2
D5	D(15,1,2,24)	-0.0136	1.1
D6	D(15,1,2,25)	-0.0166	1.4
D9	D(23,1,2,25)	-0.0039	0.3
D10	D(2,1,6,5)	0.005	0.4
D11	D(15,1,6,5)	-0.0117	1.0
D13	D(2,1,15,16)	0.0397	3.3
D14	D(2,1,15,20)	-0.0088	0.7
D15	D(6,1,15,16)	0.0391	3.3
D16	D(6,1,15,20)	-0.0094	0.8
D17	D(23,1,15,16)	0.0273	2.3
D18	D(23,1,15,20)	-0.0212	1.8
D19	D(1,2,3,4)	0.0038	0.3
D20	D(1,2,3,11)	0.0096	0.8
D21	D(24,2,3,4)	0.0054	0.5
D22	D(24,2,3,11)	0.0112	0.9
D23	D(25,2,3,4)	0.0043	0.4
D24	D(25,2,3,11)	0.0101	0.8
D25	D(2,3,4,5)	-0.0127	1.1
D27	D(11,3,4,5)	-0.0188	1.6
D28	D(11,3,4,10)	-0.007	0.6
D29	D(3,4,5,6)	0.0199	1.7
D30	D(3,4,5,7)	0.023	1.9
D31	D(10,4,5,6)	0.0093	0.8

! D32	D(10,4,5,7)	0.0124	1.0	!
! D33	D(3,4,10,9)	-0.0242	2.0	!
! D34	D(3,4,10,14)	-0.0248	2.1	!
! D35	D(5,4,10,9)	-0.0128	1.1	!
! D36	D(5,4,10,14)	-0.0134	1.1	!
! D37	D(4,5,6,1)	-0.0152	1.3	!
! D38	D(7,5,6,1)	-0.018	1.5	!
! D40	D(4,5,7,26)	-0.0062	0.5	!
! D43	D(5,7,8,9)	-0.009	0.8	!
! D44	D(5,7,8,12)	-0.0086	0.7	!
! D45	D(26,7,8,9)	-0.0046	0.4	!
! D46	D(26,7,8,12)	-0.0043	0.4	!
! D47	D(7,8,9,10)	0.0085	0.7	!
! D48	D(7,8,9,27)	0.0078	0.7	!
! D49	D(12,8,9,10)	0.0081	0.7	!
! D50	D(12,8,9,27)	0.0073	0.6	!
! D51	D(7,8,12,13)	0.018	1.5	!
! D52	D(9,8,12,13)	0.0184	1.5	!
! D56	D(27,9,10,14)	0.0041	0.3	!
! D59	D(8,12,13,29)	-0.0142	1.2	!
! D60	D(8,12,13,30)	-0.0138	1.2	!
! D61	D(8,12,13,31)	-0.0141	1.2	!
! D62	D(1,15,16,17)	-0.0608	5.1	!
! D63	D(1,15,16,32)	-0.0427	3.6	!
! D64	D(20,15,16,17)	-0.0128	1.1	!
! D65	D(20,15,16,32)	0.0053	0.4	!
! D66	D(1,15,20,19)	0.0617	5.2	!
! D67	D(1,15,20,33)	0.0416	3.5	!
! D68	D(16,15,20,19)	0.0121	1.0	!
! D69	D(16,15,20,33)	-0.0079	0.7	!
! D70	D(15,16,17,18)	0.0057	0.5	!
! D71	D(15,16,17,22)	0.0135	1.1	!
! D72	D(32,16,17,18)	-0.0123	1.0	!
! D73	D(32,16,17,22)	-0.0045	0.4	!
! D75	D(16,17,18,34)	0.0057	0.5	!
! D76	D(22,17,18,19)	-0.0055	0.5	!
! D79	D(18,17,22,35)	0.0041	0.3	!
! D81	D(17,18,19,21)	0.0071	0.6	!
! D82	D(34,18,19,20)	-0.0064	0.5	!
! D84	D(18,19,20,15)	-0.0044	0.4	!
! D85	D(18,19,20,33)	0.0153	1.3	!
! D86	D(21,19,20,15)	-0.0136	1.1	!
! D87	D(21,19,20,33)	0.0061	0.5	!
! D88	D(18,19,21,36)	-0.0048	0.4	!
! D89	D(20,19,21,36)	0.0045	0.4	!

! Normal Mode 4 !

! Name	Definition	Value	Relative Weight (%)	!

```
 ------------------------------------------------------------------------------
 ! A2    A(2,1,15)             -0.0078                 0.3                    !
 ! D1    D(6,1,2,3)             0.0092                 0.4                    !
 ! D2    D(6,1,2,24)            0.0093                 0.4                    !
 ! D4    D(15,1,2,3)            0.0109                 0.5                    !
 ! D5    D(15,1,2,24)           0.011                  0.5                    !
 ! D7    D(23,1,2,3)            0.0104                 0.5                    !
 ! D8    D(23,1,2,24)           0.0104                 0.5                    !
 ! D13   D(2,1,15,16)          -0.0279                 1.2                    !
 ! D14   D(2,1,15,20)          -0.0427                 1.9                    !
 ! D15   D(6,1,15,16)          -0.0242                 1.1                    !
 ! D16   D(6,1,15,20)          -0.039                  1.7                    !
 ! D17   D(23,1,15,16)         -0.0277                 1.2                    !
 ! D18   D(23,1,15,20)         -0.0425                 1.9                    !
 ! D19   D(1,2,3,4)            -0.0445                 2.0                    !
 ! D20   D(1,2,3,11)           -0.0655                 2.9                    !
 ! D21   D(24,2,3,4)           -0.0471                 2.1                    !
 ! D22   D(24,2,3,11)          -0.0681                 3.0                    !
 ! D23   D(25,2,3,4)           -0.0407                 1.8                    !
 ! D24   D(25,2,3,11)          -0.0616                 2.7                    !
 ! D25   D(2,3,4,5)             0.0702                 3.1                    !
 ! D26   D(2,3,4,10)            0.0286                 1.3                    !
 ! D27   D(11,3,4,5)            0.0924                 4.1                    !
 ! D28   D(11,3,4,10)           0.0509                 2.3                    !
 ! D29   D(3,4,5,6)            -0.0605                 2.7                    !
 ! D30   D(3,4,5,7)            -0.0734                 3.2                    !
 ! D31   D(10,4,5,6)           -0.0217                 1.0                    !
 ! D32   D(10,4,5,7)           -0.0346                 1.5                    !
 ! D33   D(3,4,10,9)            0.0805                 3.6                    !
 ! D34   D(3,4,10,14)           0.0824                 3.6                    !
 ! D35   D(5,4,10,9)            0.0402                 1.8                    !
 ! D36   D(5,4,10,14)           0.042                  1.9                    !
 ! D37   D(4,5,6,1)             0.0219                 1.0                    !
 ! D38   D(7,5,6,1)             0.0339                 1.5                    !
 ! D40   D(4,5,7,26)            0.0219                 1.0                    !
 ! D41   D(6,5,7,8)            -0.0133                 0.6                    !
 ! D42   D(6,5,7,26)            0.0099                 0.4                    !
 ! D43   D(5,7,8,9)             0.0332                 1.5                    !
 ! D44   D(5,7,8,12)            0.0332                 1.5                    !
 ! D45   D(26,7,8,9)            0.01                   0.4                    !
 ! D46   D(26,7,8,12)           0.01                   0.4                    !
 ! D47   D(7,8,9,10)           -0.0276                 1.2                    !
 ! D48   D(7,8,9,27)           -0.0268                 1.2                    !
 ! D49   D(12,8,9,10)          -0.0276                 1.2                    !
 ! D50   D(12,8,9,27)          -0.0269                 1.2                    !
 ! D51   D(7,8,12,13)          -0.1092                 4.8                    !
 ! D52   D(9,8,12,13)          -0.1092                 4.8                    !
 ! D53   D(8,9,10,4)           -0.0104                 0.5                    !
 ! D54   D(8,9,10,14)          -0.0123                 0.5                    !
 ! D55   D(27,9,10,4)          -0.0111                 0.5                    !
```

D56	D(27,9,10,14)	-0.013	0.6
D59	D(8,12,13,29)	0.0701	3.1
D60	D(8,12,13,30)	0.0689	3.0
D61	D(8,12,13,31)	0.0703	3.1
D62	D(1,15,16,17)	-0.0174	0.8
D63	D(1,15,16,32)	-0.0131	0.6
D66	D(1,15,20,19)	0.0182	0.8
D67	D(1,15,20,33)	0.0126	0.6

! Normal Mode 5 !

Name	Definition	Value	Relative Weight (%)
A2	A(2,1,15)	-0.0133	0.4
A7	A(1,2,3)	0.0296	0.9
A10	A(3,2,24)	-0.0149	0.4
A13	A(2,3,4)	0.0114	0.3
A43	A(1,15,16)	0.0122	0.4
A44	A(1,15,20)	-0.0132	0.4
D1	D(6,1,2,3)	0.0696	2.0
D2	D(6,1,2,24)	0.0693	2.0
D3	D(6,1,2,25)	0.0558	1.6
D4	D(15,1,2,3)	0.0885	2.6
D5	D(15,1,2,24)	0.0882	2.6
D6	D(15,1,2,25)	0.0747	2.2
D7	D(23,1,2,3)	0.0783	2.3
D8	D(23,1,2,24)	0.0781	2.3
D9	D(23,1,2,25)	0.0645	1.9
D10	D(2,1,6,5)	0.0135	0.4
D13	D(2,1,15,16)	0.0587	1.7
D14	D(2,1,15,20)	0.0442	1.3
D15	D(6,1,15,16)	0.071	2.1
D16	D(6,1,15,20)	0.0565	1.6
D17	D(23,1,15,16)	0.0671	2.0
D18	D(23,1,15,20)	0.0526	1.5
D19	D(1,2,3,4)	-0.122	3.5
D20	D(1,2,3,11)	-0.1467	4.3
D21	D(24,2,3,4)	-0.13	3.8
D22	D(24,2,3,11)	-0.1547	4.5
D23	D(25,2,3,4)	-0.1083	3.2
D24	D(25,2,3,11)	-0.1331	3.9
D25	D(2,3,4,5)	0.1073	3.1
D26	D(2,3,4,10)	0.0968	2.8
D27	D(11,3,4,5)	0.1336	3.9
D28	D(11,3,4,10)	0.1231	3.6
D29	D(3,4,5,6)	-0.0235	0.7
D30	D(3,4,5,7)	-0.0275	0.8
D31	D(10,4,5,6)	-0.0139	0.4
D32	D(10,4,5,7)	-0.0179	0.5

! D33	D(3,4,10,9)	0.0134	0.4	!
! D34	D(3,4,10,14)	0.0253	0.7	!
! D36	D(5,4,10,14)	0.0152	0.4	!
! D37	D(4,5,6,1)	-0.0419	1.2	!
! D38	D(7,5,6,1)	-0.0382	1.1	!
! D39	D(4,5,7,8)	0.0213	0.6	!
! D41	D(6,5,7,8)	0.0176	0.5	!
! D44	D(5,7,8,12)	-0.0138	0.4	!
! D51	D(7,8,12,13)	0.1217	3.5	!
! D52	D(9,8,12,13)	0.1174	3.4	!
! D57	D(4,10,14,28)	-0.0209	0.6	!
! D59	D(8,12,13,29)	-0.0585	1.7	!
! D60	D(8,12,13,30)	-0.0577	1.7	!
! D61	D(8,12,13,31)	-0.0587	1.7	!
! D62	D(1,15,16,17)	-0.0211	0.6	!
! D63	D(1,15,16,32)	-0.0131	0.4	!
! D66	D(1,15,20,19)	0.0204	0.6	!
! D67	D(1,15,20,33)	0.0122	0.4	!

! Normal Mode 6 !

! Name	Definition	Value	Relative Weight (%)	!
! A7	A(1,2,3)	-0.0234	0.6	!
! A13	A(2,3,4)	-0.014	0.3	!
! D1	D(6,1,2,3)	-0.0638	1.5	!
! D2	D(6,1,2,24)	-0.0647	1.6	!
! D3	D(6,1,2,25)	-0.0558	1.4	!
! D4	D(15,1,2,3)	-0.0646	1.6	!
! D5	D(15,1,2,24)	-0.0655	1.6	!
! D6	D(15,1,2,25)	-0.0567	1.4	!
! D7	D(23,1,2,3)	-0.0647	1.6	!
! D8	D(23,1,2,24)	-0.0656	1.6	!
! D9	D(23,1,2,25)	-0.0567	1.4	!
! D10	D(2,1,6,5)	-0.0655	1.6	!
! D11	D(15,1,6,5)	-0.0648	1.6	!
! D12	D(23,1,6,5)	-0.0636	1.5	!
! D13	D(2,1,15,16)	-0.0909	2.2	!
! D14	D(2,1,15,20)	-0.0951	2.3	!
! D15	D(6,1,15,16)	-0.0904	2.2	!
! D16	D(6,1,15,20)	-0.0946	2.3	!
! D17	D(23,1,15,16)	-0.0917	2.2	!
! D18	D(23,1,15,20)	-0.0959	2.3	!
! D19	D(1,2,3,4)	0.111	2.7	!
! D20	D(1,2,3,11)	0.1078	2.6	!
! D21	D(24,2,3,4)	0.1188	2.9	!
! D22	D(24,2,3,11)	0.1156	2.8	!
! D23	D(25,2,3,4)	0.1016	2.5	!
! D24	D(25,2,3,11)	0.0984	2.4	!

D25	D(2,3,4,5)	-0.0453	1.1
D26	D(2,3,4,10)	-0.0884	2.1
D27	D(11,3,4,5)	-0.0419	1.0
D28	D(11,3,4,10)	-0.085	2.1
D29	D(3,4,5,6)	-0.0893	2.2
D30	D(3,4,5,7)	-0.0784	1.9
D31	D(10,4,5,6)	-0.049	1.2
D32	D(10,4,5,7)	-0.0381	0.9
D33	D(3,4,10,9)	0.0754	1.8
D34	D(3,4,10,14)	0.0682	1.7
D35	D(5,4,10,9)	0.0334	0.8
D36	D(5,4,10,14)	0.0263	0.6
D37	D(4,5,6,1)	0.1464	3.6
D38	D(7,5,6,1)	0.1363	3.3
D39	D(4,5,7,8)	0.0141	0.3
D41	D(6,5,7,8)	0.0243	0.6
D42	D(6,5,7,26)	0.0154	0.4
D43	D(5,7,8,9)	0.0163	0.4
D44	D(5,7,8,12)	0.0164	0.4
D45	D(26,7,8,9)	0.0252	0.6
D46	D(26,7,8,12)	0.0253	0.6
D47	D(7,8,9,10)	-0.0207	0.5
D49	D(12,8,9,10)	-0.0208	0.5
D51	D(7,8,12,13)	0.1988	4.8
D52	D(9,8,12,13)	0.199	4.8
D55	D(27,9,10,4)	-0.0134	0.3
D57	D(4,10,14,28)	0.0291	0.7
D58	D(9,10,14,28)	0.0221	0.5
D59	D(8,12,13,29)	-0.0851	2.1
D60	D(8,12,13,30)	-0.0838	2.0
D61	D(8,12,13,31)	-0.0853	2.1

! Normal Mode 7 !

Name	Definition	Value	Relative Weight (%)
R3	R(1,15)	0.0315	0.7
A1	A(2,1,6)	0.0157	0.3
A4	A(6,1,15)	0.0237	0.5
A6	A(15,1,23)	-0.0274	0.6
A7	A(1,2,3)	0.0445	1.0
A10	A(3,2,24)	-0.0253	0.5
A13	A(2,3,4)	0.0167	0.4
A22	A(1,6,5)	0.0199	0.4
A43	A(1,15,16)	-0.0348	0.8
A44	A(1,15,20)	0.0526	1.1
A45	A(16,15,20)	-0.0159	0.3
A46	A(15,16,17)	0.0152	0.3
A56	A(18,19,21)	-0.0192	0.4

```
! A57   A(20,19,21)            0.0146              0.3              !
! D1    D(6,1,2,3)             0.122               2.6              !
! D2    D(6,1,2,24)            0.1242              2.7              !
! D3    D(6,1,2,25)            0.1082              2.3              !
! D4    D(15,1,2,3)            0.0737              1.6              !
! D5    D(15,1,2,24)           0.0759              1.6              !
! D6    D(15,1,2,25)           0.0598              1.3              !
! D7    D(23,1,2,3)            0.1091              2.4              !
! D8    D(23,1,2,24)           0.1112              2.4              !
! D9    D(23,1,2,25)           0.0952              2.1              !
! D10   D(2,1,6,5)            -0.0567              1.2              !
! D11   D(15,1,6,5)           -0.0187              0.4              !
! D12   D(23,1,6,5)           -0.0457              1.0              !
! D13   D(2,1,15,16)           0.0208              0.4              !
! D14   D(2,1,15,20)           0.0656              1.4              !
! D15   D(6,1,15,16)          -0.0226              0.5              !
! D16   D(6,1,15,20)           0.0222              0.5              !
! D18   D(23,1,15,20)          0.0401              0.9              !
! D19   D(1,2,3,4)            -0.1079              2.3              !
! D20   D(1,2,3,11)           -0.1498              3.2              !
! D21   D(24,2,3,4)           -0.1297              2.8              !
! D22   D(24,2,3,11)          -0.1716              3.7              !
! D23   D(25,2,3,4)           -0.0985              2.1              !
! D24   D(25,2,3,11)          -0.1404              3.0              !
! D25   D(2,3,4,5)             0.0486              1.0              !
! D26   D(2,3,4,10)           -0.0145              0.3              !
! D27   D(11,3,4,5)            0.0928              2.0              !
! D28   D(11,3,4,10)           0.0297              0.6              !
! D29   D(3,4,5,6)             0.0227              0.5              !
! D30   D(3,4,5,7)            -0.0215              0.5              !
! D31   D(10,4,5,6)            0.0823              1.8              !
! D32   D(10,4,5,7)            0.0381              0.8              !
! D33   D(3,4,10,9)            0.0406              0.9              !
! D34   D(3,4,10,14)           0.0202              0.4              !
! D35   D(5,4,10,9)           -0.0209              0.5              !
! D36   D(5,4,10,14)          -0.0413              0.9              !
! D37   D(4,5,6,1)            -0.022               0.5              !
! D38   D(7,5,6,1)             0.0194              0.4              !
! D39   D(4,5,7,8)            -0.0454              1.0              !
! D41   D(6,5,7,8)            -0.0868              1.9              !
! D42   D(6,5,7,26)           -0.0298              0.6              !
! D43   D(5,7,8,9)             0.0346              0.7              !
! D44   D(5,7,8,12)            0.0624              1.3              !
! D45   D(26,7,8,9)           -0.0224              0.5              !
! D47   D(7,8,9,10)           -0.0185              0.4              !
! D49   D(12,8,9,10)          -0.0489              1.1              !
! D50   D(12,8,9,27)          -0.0226              0.5              !
! D51   D(7,8,12,13)           0.1248              2.7              !
! D52   D(9,8,12,13)           0.1537              3.3              !
! D54   D(8,9,10,14)           0.0328              0.7              !
```

!	D57	D(4,10,14,28)	0.0146	0.3
!	D59	D(8,12,13,29)	-0.0294	0.6
!	D60	D(8,12,13,30)	-0.0281	0.6
!	D61	D(8,12,13,31)	-0.0296	0.6
!	D62	D(1,15,16,17)	0.074	1.6
!	D63	D(1,15,16,32)	0.0407	0.9
!	D64	D(20,15,16,17)	0.0273	0.6
!	D66	D(1,15,20,19)	-0.0761	1.6
!	D67	D(1,15,20,33)	-0.0396	0.9
!	D68	D(16,15,20,19)	-0.031	0.7
!	D71	D(15,16,17,22)	-0.032	0.7
!	D72	D(32,16,17,18)	0.0215	0.5
!	D75	D(16,17,18,34)	-0.015	0.3
!	D76	D(22,17,18,19)	0.0194	0.4
!	D81	D(17,18,19,21)	-0.0247	0.5
!	D84	D(18,19,20,15)	0.0195	0.4
!	D85	D(18,19,20,33)	-0.0164	0.4
!	D86	D(21,19,20,15)	0.039	0.8
!	D89	D(20,19,21,36)	-0.0177	0.4

! Normal Mode 8 !

! Name	Definition	Value	Relative Weight (%)
! R2	R(1,6)	0.024	1.0
! R5	R(2,3)	-0.0126	0.5
! R8	R(3,4)	-0.01	0.4
! R11	R(4,10)	-0.0099	0.4
! R12	R(5,6)	0.0084	0.4
! R13	R(5,7)	0.0154	0.7
! R14	R(7,8)	0.0135	0.6
! R18	R(9,10)	-0.0162	0.7
! R21	R(12,13)	0.0094	0.4
! A2	A(2,1,15)	-0.0126	0.5
! A6	A(15,1,23)	0.0078	0.3
! A7	A(1,2,3)	-0.0104	0.4
! A14	A(2,3,11)	0.0104	0.4
! A15	A(4,3,11)	-0.0113	0.5
! A16	A(3,4,5)	0.0194	0.8
! A17	A(3,4,10)	-0.0314	1.3
! A18	A(5,4,10)	0.0136	0.6
! A19	A(4,5,6)	-0.0209	0.9
! A20	A(4,5,7)	-0.0093	0.4
! A21	A(6,5,7)	0.0304	1.3
! A23	A(5,7,8)	-0.0097	0.4
! A27	A(7,8,12)	0.0458	2.0
! A28	A(9,8,12)	-0.0499	2.1
! A31	A(10,9,27)	-0.0073	0.3
! A33	A(4,10,14)	0.0094	0.4

```
! A35   A(8,12,13)            -0.0362                 1.6                   !
! A43   A(1,15,16)             0.0343                 1.5                   !
! A44   A(1,15,20)            -0.0318                 1.4                   !
! A50   A(16,17,22)            0.0096                 0.4                   !
! A51   A(18,17,22)           -0.0118                 0.5                   !
! A56   A(18,19,21)            0.009                  0.4                   !
! A57   A(20,19,21)           -0.0081                 0.3                   !
! D1    D(6,1,2,3)            -0.0093                 0.4                   !
! D2    D(6,1,2,24)           -0.0075                 0.3                   !
! D7    D(23,1,2,3)           -0.0098                 0.4                   !
! D8    D(23,1,2,24)          -0.0079                 0.3                   !
! D11   D(15,1,6,5)           -0.0142                 0.6                   !
! D13   D(2,1,15,16)          -0.0097                 0.4                   !
! D14   D(2,1,15,20)           0.0144                 0.6                   !
! D16   D(6,1,15,20)           0.0169                 0.7                   !
! D18   D(23,1,15,20)          0.0177                 0.8                   !
! D19   D(1,2,3,4)             0.0135                 0.6                   !
! D20   D(1,2,3,11)            0.0213                 0.9                   !
! D21   D(24,2,3,4)            0.0149                 0.6                   !
! D22   D(24,2,3,11)           0.0227                 1.0                   !
! D23   D(25,2,3,4)            0.0078                 0.3                   !
! D24   D(25,2,3,11)           0.0156                 0.7                   !
! D26   D(2,3,4,10)            0.0223                 1.0                   !
! D27   D(11,3,4,5)           -0.0128                 0.5                   !
! D28   D(11,3,4,10)           0.0136                 0.6                   !
! D29   D(3,4,5,6)            -0.0109                 0.5                   !
! D30   D(3,4,5,7)             0.0093                 0.4                   !
! D31   D(10,4,5,6)           -0.0335                 1.4                   !
! D32   D(10,4,5,7)           -0.0134                 0.6                   !
! D33   D(3,4,10,9)           -0.0134                 0.6                   !
! D35   D(5,4,10,9)            0.0126                 0.5                   !
! D36   D(5,4,10,14)           0.028                  1.2                   !
! D37   D(4,5,6,1)             0.0174                 0.7                   !
! D39   D(4,5,7,8)             0.0227                 1.0                   !
! D40   D(4,5,7,26)           -0.0124                 0.5                   !
! D41   D(6,5,7,8)             0.0412                 1.8                   !
! D43   D(5,7,8,9)            -0.0311                 1.3                   !
! D44   D(5,7,8,12)           -0.0535                 2.3                   !
! D46   D(26,7,8,12)          -0.0183                 0.8                   !
! D47   D(7,8,9,10)            0.0303                 1.3                   !
! D49   D(12,8,9,10)           0.0538                 2.3                   !
! D50   D(12,8,9,27)           0.0261                 1.1                   !
! D51   D(7,8,12,13)          -0.0576                 2.5                   !
! D52   D(9,8,12,13)          -0.0803                 3.4                   !
! D53   D(8,9,10,4)           -0.0212                 0.9                   !
! D54   D(8,9,10,14)          -0.037                  1.6                   !
! D56   D(27,9,10,14)         -0.0099                 0.4                   !
! D57   D(4,10,14,28)         -0.0171                 0.7                   !
! D59   D(8,12,13,29)         -0.0349                 1.5                   !
! D60   D(8,12,13,30)         -0.0348                 1.5                   !
```

Name	Definition	Value	Relative Weight (%)
D61	D(8,12,13,31)	-0.0326	1.4
D62	D(1,15,16,17)	0.0596	2.6
D63	D(1,15,16,32)	0.0273	1.2
D64	D(20,15,16,17)	0.0364	1.6
D66	D(1,15,20,19)	-0.0589	2.5
D67	D(1,15,20,33)	-0.0245	1.0
D68	D(16,15,20,19)	-0.0334	1.4
D70	D(15,16,17,18)	-0.0211	0.9
D71	D(15,16,17,22)	-0.04	1.7
D72	D(32,16,17,18)	0.0111	0.5
D73	D(32,16,17,22)	-0.0079	0.3
D75	D(16,17,18,34)	-0.0123	0.5
D76	D(22,17,18,19)	0.0208	0.9
D79	D(18,17,22,35)	-0.0178	0.8
D81	D(17,18,19,21)	-0.0215	0.9
D82	D(34,18,19,20)	0.0156	0.7
D84	D(18,19,20,15)	0.0152	0.7
D85	D(18,19,20,33)	-0.0186	0.8
D86	D(21,19,20,15)	0.0356	1.5
D89	D(20,19,21,36)	-0.0164	0.7

! Normal Mode 9 !

Name	Definition	Value	Relative Weight (%)
R3	R(1,15)	0.0076	0.3
A7	A(1,2,3)	0.0073	0.3
A22	A(1,6,5)	0.0093	0.4
A27	A(7,8,12)	-0.0071	0.3
A35	A(8,12,13)	0.008	0.4
A43	A(1,15,16)	-0.0076	0.3
A44	A(1,15,20)	0.0114	0.5
D1	D(6,1,2,3)	0.0132	0.6
D2	D(6,1,2,24)	0.0129	0.6
D3	D(6,1,2,25)	0.0109	0.5
D7	D(23,1,2,3)	0.0109	0.5
D8	D(23,1,2,24)	0.0107	0.5
D9	D(23,1,2,25)	0.0086	0.4
D10	D(2,1,6,5)	-0.0336	1.5
D11	D(15,1,6,5)	-0.0265	1.2
D12	D(23,1,6,5)	-0.031	1.4
D15	D(6,1,15,16)	-0.0122	0.5
D16	D(6,1,15,20)	-0.0115	0.5
D17	D(23,1,15,16)	-0.0101	0.4
D18	D(23,1,15,20)	-0.0095	0.4
D20	D(1,2,3,11)	-0.0082	0.4
D22	D(24,2,3,11)	-0.0106	0.5
D23	D(25,2,3,4)	0.0092	0.4
D26	D(2,3,4,10)	-0.0147	0.6

!	D27	D(11,3,4,5)	0.0107	0.5
!	D29	D(3,4,5,6)	-0.0136	0.6
!	D32	D(10,4,5,7)	0.0091	0.4
!	D33	D(3,4,10,9)	0.0566	2.5
!	D34	D(3,4,10,14)	0.0785	3.5
!	D35	D(5,4,10,9)	0.0476	2.1
!	D36	D(5,4,10,14)	0.0695	3.1
!	D37	D(4,5,6,1)	0.0317	1.4
!	D38	D(7,5,6,1)	0.0181	0.8
!	D39	D(4,5,7,8)	-0.0223	1.0
!	D40	D(4,5,7,26)	-0.0382	1.7
!	D41	D(6,5,7,8)	-0.0087	0.4
!	D42	D(6,5,7,26)	-0.0246	1.1
!	D43	D(5,7,8,9)	-0.0205	0.9
!	D44	D(5,7,8,12)	-0.055	2.4
!	D46	D(26,7,8,12)	-0.0391	1.7
!	D47	D(7,8,9,10)	0.0758	3.3
!	D48	D(7,8,9,27)	0.0085	0.4
!	D49	D(12,8,9,10)	0.1134	5.0
!	D50	D(12,8,9,27)	0.0461	2.0
!	D52	D(9,8,12,13)	-0.0379	1.7
!	D53	D(8,9,10,4)	-0.0901	4.0
!	D54	D(8,9,10,14)	-0.1124	5.0
!	D55	D(27,9,10,4)	-0.0244	1.1
!	D56	D(27,9,10,14)	-0.0467	2.1
!	D57	D(4,10,14,28)	-0.0174	0.8
!	D59	D(8,12,13,29)	-0.2027	8.9
!	D60	D(8,12,13,30)	-0.205	9.0
!	D61	D(8,12,13,31)	-0.204	9.0

! Normal Mode 10 !

! Name	Definition	Value	Relative Weight (%)
! R2	R(1,6)	0.0281	0.6
! R3	R(1,15)	0.0568	1.3
! R5	R(2,3)	0.0295	0.7
! R11	R(4,10)	0.0152	0.3
! R12	R(5,6)	0.0208	0.5
! R26	R(15,16)	0.0277	0.6
! R28	R(16,17)	0.0203	0.5
! A1	A(2,1,6)	-0.0472	1.1
! A2	A(2,1,15)	0.0355	0.8
! A4	A(6,1,15)	0.0213	0.5
! A16	A(3,4,5)	-0.0282	0.6
! A17	A(3,4,10)	0.0255	0.6
! A20	A(4,5,7)	-0.0152	0.3
! A22	A(1,6,5)	0.0293	0.7
! A35	A(8,12,13)	0.0181	0.4

```
! A43   A(1,15,16)              0.0488                 1.1                    !
! A44   A(1,15,20)             -0.0211                 0.5                    !
! A45   A(16,15,20)            -0.0287                 0.6                    !
! A50   A(16,17,22)             0.0343                 0.8                    !
! A51   A(18,17,22)            -0.0378                 0.8                    !
! A58   A(15,20,19)             0.0204                 0.5                    !
! D1    D(6,1,2,3)             -0.0234                 0.5                    !
! D2    D(6,1,2,24)            -0.0286                 0.6                    !
! D3    D(6,1,2,25)            -0.0227                 0.5                    !
! D4    D(15,1,2,3)            -0.0407                 0.9                    !
! D5    D(15,1,2,24)           -0.0459                 1.0                    !
! D6    D(15,1,2,25)           -0.04                   0.9                    !
! D7    D(23,1,2,3)            -0.0507                 1.1                    !
! D8    D(23,1,2,24)           -0.0559                 1.2                    !
! D9    D(23,1,2,25)           -0.05                   1.1                    !
! D10   D(2,1,6,5)             -0.0258                 0.6                    !
! D14   D(2,1,15,20)           -0.0351                 0.8                    !
! D18   D(23,1,15,20)          -0.0173                 0.4                    !
! D19   D(1,2,3,4)              0.0748                 1.7                    !
! D20   D(1,2,3,11)             0.0878                 2.0                    !
! D21   D(24,2,3,4)             0.0851                 1.9                    !
! D22   D(24,2,3,11)            0.098                  2.2                    !
! D23   D(25,2,3,4)             0.0869                 1.9                    !
! D24   D(25,2,3,11)            0.0999                 2.2                    !
! D25   D(2,3,4,5)             -0.0362                 0.8                    !
! D26   D(2,3,4,10)             0.0246                 0.5                    !
! D27   D(11,3,4,5)            -0.05                   1.1                    !
! D29   D(3,4,5,6)             -0.0199                 0.4                    !
! D31   D(10,4,5,6)            -0.0775                 1.7                    !
! D32   D(10,4,5,7)            -0.0577                 1.3                    !
! D33   D(3,4,10,9)            -0.0658                 1.5                    !
! D34   D(3,4,10,14)           -0.0507                 1.1                    !
! D37   D(4,5,6,1)              0.0383                 0.9                    !
! D38   D(7,5,6,1)              0.0199                 0.4                    !
! D39   D(4,5,7,8)              0.0823                 1.8                    !
! D40   D(4,5,7,26)             0.0209                 0.5                    !
! D41   D(6,5,7,8)              0.1009                 2.2                    !
! D42   D(6,5,7,26)             0.0396                 0.9                    !
! D43   D(5,7,8,9)             -0.04                   0.9                    !
! D44   D(5,7,8,12)            -0.053                  1.2                    !
! D45   D(26,7,8,9)             0.0214                 0.5                    !
! D47   D(7,8,9,10)            -0.0237                 0.5                    !
! D51   D(7,8,12,13)           -0.0905                 2.0                    !
! D52   D(9,8,12,13)           -0.1041                 2.3                    !
! D53   D(8,9,10,4)             0.0481                 1.1                    !
! D54   D(8,9,10,14)            0.0326                 0.7                    !
! D55   D(27,9,10,4)            0.0384                 0.9                    !
! D56   D(27,9,10,14)           0.0228                 0.5                    !
! D57   D(4,10,14,28)          -0.0146                 0.3                    !
! D59   D(8,12,13,29)           0.1367                 3.0                    !
```

```
! D60   D(8,12,13,30)          0.1353                 3.0                    !
! D61   D(8,12,13,31)          0.1341                 3.0                    !
! D62   D(1,15,16,17)         -0.0344                 0.8                    !
! D65   D(20,15,16,32)         0.0223                 0.5                    !
! D66   D(1,15,20,19)          0.0468                 1.0                    !
! D68   D(16,15,20,19)         0.027                  0.6                    !
! D69   D(16,15,20,33)        -0.024                  0.5                    !
! D70   D(15,16,17,18)         0.0207                 0.5                    !
! D71   D(15,16,17,22)         0.0599                 1.3                    !
! D72   D(32,16,17,18)        -0.0145                 0.3                    !
! D73   D(32,16,17,22)         0.0246                 0.5                    !
! D74   D(16,17,18,19)        -0.0418                 0.9                    !
! D75   D(16,17,18,34)         0.0211                 0.5                    !
! D76   D(22,17,18,19)        -0.0792                 1.8                    !
! D77   D(22,17,18,34)        -0.0163                 0.4                    !
! D79   D(18,17,22,35)         0.0264                 0.6                    !
! D80   D(17,18,19,20)         0.0564                 1.3                    !
! D81   D(17,18,19,21)         0.0978                 2.2                    !
! D83   D(34,18,19,21)         0.0333                 0.7                    !
! D84   D(18,19,20,15)        -0.0491                 1.1                    !
! D86   D(21,19,20,15)        -0.0885                 2.0                    !
! D87   D(21,19,20,33)        -0.0384                 0.9                    !
! D89   D(20,19,21,36)         0.0398                 0.9                    !
--------------------------------------------------------------------------------
                          ----------------------------
                          ! Normal Mode    11        !
--------------------------                            --------------------------
! Name  Definition             Value          Relative Weight (%)            !
--------------------------------------------------------------------------------
! A2    A(2,1,15)             -0.0244                 0.6                    !
! A4    A(6,1,15)              0.0222                 0.5                    !
! A27   A(7,8,12)             -0.0191                 0.5                    !
! A28   A(9,8,12)              0.0195                 0.5                    !
! A35   A(8,12,13)             0.0266                 0.7                    !
! A43   A(1,15,16)             0.0134                 0.3                    !
! D6    D(15,1,2,25)          -0.0154                 0.4                    !
! D10   D(2,1,6,5)            -0.0222                 0.5                    !
! D11   D(15,1,6,5)           -0.0443                 1.1                    !
! D12   D(23,1,6,5)           -0.027                  0.7                    !
! D13   D(2,1,15,16)          -0.015                  0.4                    !
! D17   D(23,1,15,16)         -0.0169                 0.4                    !
! D19   D(1,2,3,4)             0.0233                 0.6                    !
! D20   D(1,2,3,11)            0.0281                 0.7                    !
! D21   D(24,2,3,4)            0.0271                 0.7                    !
! D22   D(24,2,3,11)           0.0318                 0.8                    !
! D23   D(25,2,3,4)            0.028                  0.7                    !
! D24   D(25,2,3,11)           0.0327                 0.8                    !
! D26   D(2,3,4,10)            0.0227                 0.6                    !
! D28   D(11,3,4,10)           0.018                  0.4                    !
! D29   D(3,4,5,6)            -0.0183                 0.5                    !
```

D31	D(10,4,5,6)	-0.0461	1.1
D32	D(10,4,5,7)	-0.0319	0.8
D33	D(3,4,10,9)	-0.0357	0.9
D34	D(3,4,10,14)	-0.0249	0.6
D37	D(4,5,6,1)	0.03	0.7
D38	D(7,5,6,1)	0.0168	0.4
D39	D(4,5,7,8)	0.0501	1.2
D41	D(6,5,7,8)	0.0634	1.6
D42	D(6,5,7,26)	0.0239	0.6
D43	D(5,7,8,9)	-0.0283	0.7
D44	D(5,7,8,12)	-0.0375	0.9
D51	D(7,8,12,13)	-0.0518	1.3
D52	D(9,8,12,13)	-0.0616	1.5
D53	D(8,9,10,4)	0.0281	0.7
D54	D(8,9,10,14)	0.0171	0.4
D55	D(27,9,10,4)	0.0246	0.6
D56	D(27,9,10,14)	0.0136	0.3
D59	D(8,12,13,29)	0.0741	1.8
D60	D(8,12,13,30)	0.0727	1.8
D61	D(8,12,13,31)	0.0714	1.8
D62	D(1,15,16,17)	-0.0204	0.5
D63	D(1,15,16,32)	-0.0639	1.6
D64	D(20,15,16,17)	-0.0332	0.8
D65	D(20,15,16,32)	-0.0767	1.9
D66	D(1,15,20,19)	0.0173	0.4
D67	D(1,15,20,33)	0.0674	1.7
D68	D(16,15,20,19)	0.0309	0.8
D69	D(16,15,20,33)	0.081	2.0
D70	D(15,16,17,18)	-0.0717	1.8
D71	D(15,16,17,22)	-0.15	3.7
D72	D(32,16,17,18)	-0.0283	0.7
D73	D(32,16,17,22)	-0.1066	2.6
D74	D(16,17,18,19)	0.1761	4.3
D76	D(22,17,18,19)	0.2499	6.1
D77	D(22,17,18,34)	0.0698	1.7
D79	D(18,17,22,35)	-0.0803	2.0
D80	D(17,18,19,20)	-0.1791	4.4
D81	D(17,18,19,21)	-0.2561	6.3
D83	D(34,18,19,21)	-0.0715	1.8
D84	D(18,19,20,15)	0.0765	1.9
D85	D(18,19,20,33)	0.0273	0.7
D86	D(21,19,20,15)	0.1499	3.7
D87	D(21,19,20,33)	0.1006	2.5
D89	D(20,19,21,36)	-0.0839	2.1

! Normal Mode 12 !

Name	Definition	Value	Relative Weight (%)

! D13	D(2,1,15,16)	0.1397	3.4	!
! D14	D(2,1,15,20)	0.1384	3.4	!
! D15	D(6,1,15,16)	0.1371	3.4	!
! D16	D(6,1,15,20)	0.1359	3.3	!
! D17	D(23,1,15,16)	0.1365	3.4	!
! D18	D(23,1,15,20)	0.1352	3.3	!
! D62	D(1,15,16,17)	0.1119	2.7	!
! D64	D(20,15,16,17)	0.113	2.8	!
! D66	D(1,15,20,19)	0.0936	2.3	!
! D68	D(16,15,20,19)	0.0922	2.3	!
! D70	D(15,16,17,18)	-0.2121	5.2	!
! D71	D(15,16,17,22)	-0.2392	5.9	!
! D72	D(32,16,17,18)	-0.099	2.4	!
! D73	D(32,16,17,22)	-0.126	3.1	!
! D74	D(16,17,18,19)	0.1032	2.5	!
! D75	D(16,17,18,34)	0.0862	2.1	!
! D76	D(22,17,18,19)	0.1287	3.2	!
! D77	D(22,17,18,34)	0.1117	2.7	!
! D78	D(16,17,22,35)	-0.0468	1.1	!
! D79	D(18,17,22,35)	-0.0728	1.8	!
! D80	D(17,18,19,20)	0.1066	2.6	!
! D81	D(17,18,19,21)	0.1215	3.0	!
! D82	D(34,18,19,20)	0.124	3.0	!
! D83	D(34,18,19,21)	0.1389	3.4	!
! D84	D(18,19,20,15)	-0.2037	5.0	!
! D85	D(18,19,20,33)	-0.0942	2.3	!
! D86	D(21,19,20,15)	-0.2179	5.4	!
! D87	D(21,19,20,33)	-0.1083	2.7	!
! D88	D(18,19,21,36)	0.0505	1.2	!
! D89	D(20,19,21,36)	0.0648	1.6	!

! Normal Mode 13 !

! Name	Definition	Value	Relative Weight (%)	!
! D1	D(6,1,2,3)	0.0139	0.4	!
! D2	D(6,1,2,24)	0.0175	0.5	!
! D3	D(6,1,2,25)	0.0174	0.5	!
! D4	D(15,1,2,3)	0.0136	0.4	!
! D5	D(15,1,2,24)	0.0172	0.5	!
! D6	D(15,1,2,25)	0.0171	0.5	!
! D7	D(23,1,2,3)	0.0148	0.5	!
! D8	D(23,1,2,24)	0.0184	0.6	!
! D9	D(23,1,2,25)	0.0183	0.6	!
! D19	D(1,2,3,4)	0.0135	0.4	!
! D20	D(1,2,3,11)	0.0257	0.8	!
! D21	D(24,2,3,4)	0.0108	0.3	!
! D22	D(24,2,3,11)	0.023	0.7	!
! D23	D(25,2,3,4)	0.0103	0.3	!

!	D24	D(25,2,3,11)	0.0225	0.7
!	D25	D(2,3,4,5)	-0.0565	1.7
!	D26	D(2,3,4,10)	0.029	0.9
!	D27	D(11,3,4,5)	-0.0695	2.1
!	D28	D(11,3,4,10)	0.0161	0.5
!	D29	D(3,4,5,6)	0.0785	2.4
!	D30	D(3,4,5,7)	0.098	3.0
!	D32	D(10,4,5,7)	0.0181	0.6
!	D33	D(3,4,10,9)	0.011	0.3
!	D34	D(3,4,10,14)	0.0526	1.6
!	D35	D(5,4,10,9)	0.0942	2.9
!	D36	D(5,4,10,14)	0.1358	4.2
!	D37	D(4,5,6,1)	-0.0504	1.6
!	D38	D(7,5,6,1)	-0.0686	2.1
!	D39	D(4,5,7,8)	-0.0871	2.7
!	D40	D(4,5,7,26)	-0.0449	1.4
!	D41	D(6,5,7,8)	-0.0689	2.1
!	D42	D(6,5,7,26)	-0.0267	0.8
!	D43	D(5,7,8,9)	0.0457	1.4
!	D44	D(5,7,8,12)	0.0435	1.3
!	D47	D(7,8,9,10)	0.0634	2.0
!	D48	D(7,8,9,27)	-0.0501	1.5
!	D49	D(12,8,9,10)	0.0658	2.0
!	D50	D(12,8,9,27)	-0.0477	1.5
!	D51	D(7,8,12,13)	0.0413	1.3
!	D52	D(9,8,12,13)	0.039	1.2
!	D53	D(8,9,10,4)	-0.1356	4.2
!	D54	D(8,9,10,14)	-0.178	5.5
!	D55	D(27,9,10,4)	-0.0247	0.8
!	D56	D(27,9,10,14)	-0.0671	2.1
!	D57	D(4,10,14,28)	-0.0631	2.0
!	D58	D(9,10,14,28)	-0.0221	0.7
!	D59	D(8,12,13,29)	0.2854	8.8
!	D60	D(8,12,13,30)	0.2895	8.9
!	D61	D(8,12,13,31)	0.2862	8.8

! Normal Mode 14 !

!	Name	Definition	Value	Relative Weight (%)
!	R1	R(1,2)	-0.0183	0.5
!	R2	R(1,6)	0.0189	0.5
!	R5	R(2,3)	-0.0276	0.7
!	A1	A(2,1,6)	0.0217	0.6
!	A2	A(2,1,15)	-0.0536	1.4
!	A4	A(6,1,15)	0.0278	0.7
!	A7	A(1,2,3)	0.0127	0.3
!	A16	A(3,4,5)	0.0125	0.3
!	A17	A(3,4,10)	-0.0257	0.7

```
! A18   A(5,4,10)              0.0141                   0.4                   !
! A20   A(4,5,7)              -0.0223                   0.6                   !
! A21   A(6,5,7)               0.0257                   0.7                   !
! A23   A(5,7,8)               0.0153                   0.4                   !
! A25   A(8,7,26)             -0.018                    0.5                   !
! A27   A(7,8,12)             -0.0386                   1.0                   !
! A28   A(9,8,12)              0.0354                   0.9                   !
! A29   A(8,9,10)             -0.0203                   0.5                   !
! A31   A(10,9,27)             0.0171                   0.4                   !
! A33   A(4,10,14)            -0.0384                   1.0                   !
! A34   A(9,10,14)             0.0282                   0.7                   !
! A35   A(8,12,13)             0.0597                   1.5                   !
! D1    D(6,1,2,3)             0.0249                   0.6                   !
! D2    D(6,1,2,24)            0.0353                   0.9                   !
! D3    D(6,1,2,25)            0.0226                   0.6                   !
! D5    D(15,1,2,24)           0.0202                   0.5                   !
! D7    D(23,1,2,3)            0.0318                   0.8                   !
! D8    D(23,1,2,24)           0.0421                   1.1                   !
! D9    D(23,1,2,25)           0.0294                   0.8                   !
! D10   D(2,1,6,5)            -0.041                    1.0                   !
! D11   D(15,1,6,5)           -0.0754                   1.9                   !
! D12   D(23,1,6,5)           -0.0604                   1.5                   !
! D14   D(2,1,15,20)           0.0214                   0.5                   !
! D19   D(1,2,3,4)            -0.0186                   0.5                   !
! D20   D(1,2,3,11)           -0.0222                   0.6                   !
! D21   D(24,2,3,4)           -0.0284                   0.7                   !
! D22   D(24,2,3,11)          -0.032                    0.8                   !
! D23   D(25,2,3,4)           -0.024                    0.6                   !
! D24   D(25,2,3,11)          -0.0277                   0.7                   !
! D25   D(2,3,4,5)             0.0129                   0.3                   !
! D26   D(2,3,4,10)            0.0278                   0.7                   !
! D27   D(11,3,4,5)            0.0172                   0.4                   !
! D28   D(11,3,4,10)           0.0321                   0.8                   !
! D29   D(3,4,5,6)            -0.0167                   0.4                   !
! D31   D(10,4,5,6)           -0.0289                   0.7                   !
! D32   D(10,4,5,7)           -0.0162                   0.4                   !
! D33   D(3,4,10,9)           -0.0203                   0.5                   !
! D37   D(4,5,6,1)             0.0371                   0.9                   !
! D38   D(7,5,6,1)             0.0256                   0.7                   !
! D39   D(4,5,7,8)             0.0357                   0.9                   !
! D41   D(6,5,7,8)             0.0473                   1.2                   !
! D42   D(6,5,7,26)            0.0133                   0.3                   !
! D43   D(5,7,8,9)            -0.0329                   0.8                   !
! D44   D(5,7,8,12)           -0.0431                   1.1                   !
! D49   D(12,8,9,10)           0.0232                   0.6                   !
! D51   D(7,8,12,13)          -0.0409                   1.0                   !
! D52   D(9,8,12,13)          -0.0518                   1.3                   !
! D55   D(27,9,10,4)           0.0237                   0.6                   !
! D59   D(8,12,13,29)          0.0953                   2.4                   !
! D60   D(8,12,13,30)          0.0927                   2.4                   !
```

D61	D(8,12,13,31)	0.089	2.3
D62	D(1,15,16,17)	0.1332	3.4
D63	D(1,15,16,32)	0.0732	1.9
D64	D(20,15,16,17)	0.1235	3.2
D65	D(20,15,16,32)	0.0635	1.6
D66	D(1,15,20,19)	-0.1369	3.5
D67	D(1,15,20,33)	-0.0668	1.7
D68	D(16,15,20,19)	-0.1267	3.2
D69	D(16,15,20,33)	-0.0566	1.4
D71	D(15,16,17,22)	-0.0197	0.5
D72	D(32,16,17,18)	0.0523	1.3
D73	D(32,16,17,22)	0.04	1.0
D74	D(16,17,18,19)	-0.1025	2.6
D75	D(16,17,18,34)	-0.0439	1.1
D76	D(22,17,18,19)	-0.0909	2.3
D77	D(22,17,18,34)	-0.0323	0.8
D80	D(17,18,19,20)	0.0992	2.5
D81	D(17,18,19,21)	0.0801	2.0
D82	D(34,18,19,20)	0.0392	1.0
D83	D(34,18,19,21)	0.0201	0.5
D84	D(18,19,20,15)	0.0146	0.4
D85	D(18,19,20,33)	-0.0542	1.4
D86	D(21,19,20,15)	0.0328	0.8
D87	D(21,19,20,33)	-0.036	0.9

! Normal Mode 15 !

Name	Definition	Value	Relative Weight (%)
R2	R(1,6)	0.0213	0.3
A1	A(2,1,6)	-0.0407	0.6
A19	A(4,5,6)	-0.0239	0.4
A21	A(6,5,7)	0.0289	0.4
A22	A(1,6,5)	-0.0432	0.6
A35	A(8,12,13)	0.0283	0.4
A43	A(1,15,16)	0.0572	0.8
A44	A(1,15,20)	-0.0551	0.8
A50	A(16,17,22)	0.0245	0.4
A51	A(18,17,22)	-0.0278	0.4
D1	D(6,1,2,3)	-0.0667	1.0
D2	D(6,1,2,24)	-0.0695	1.0
D3	D(6,1,2,25)	-0.0583	0.9
D4	D(15,1,2,3)	-0.065	1.0
D5	D(15,1,2,24)	-0.0677	1.0
D6	D(15,1,2,25)	-0.0565	0.8
D7	D(23,1,2,3)	-0.0839	1.2
D8	D(23,1,2,24)	-0.0867	1.3
D9	D(23,1,2,25)	-0.0755	1.1
D10	D(2,1,6,5)	0.2107	3.1

! D11	D(15,1,6,5)	0.2027	3.0	!
! D12	D(23,1,6,5)	0.2226	3.3	!
! D13	D(2,1,15,16)	0.0331	0.5	!
! D14	D(2,1,15,20)	0.0243	0.4	!
! D15	D(6,1,15,16)	0.0684	1.0	!
! D16	D(6,1,15,20)	0.0596	0.9	!
! D17	D(23,1,15,16)	0.052	0.8	!
! D18	D(23,1,15,20)	0.0432	0.6	!
! D19	D(1,2,3,4)	-0.023	0.3	!
! D22	D(24,2,3,11)	0.0263	0.4	!
! D27	D(11,3,4,5)	-0.0424	0.6	!
! D28	D(11,3,4,10)	-0.0494	0.7	!
! D29	D(3,4,5,6)	0.1473	2.2	!
! D30	D(3,4,5,7)	0.0951	1.4	!
! D31	D(10,4,5,6)	0.1543	2.3	!
! D32	D(10,4,5,7)	0.1022	1.5	!
! D33	D(3,4,10,9)	0.044	0.6	!
! D35	D(5,4,10,9)	0.0372	0.5	!
! D37	D(4,5,6,1)	-0.2513	3.7	!
! D38	D(7,5,6,1)	-0.2023	3.0	!
! D39	D(4,5,7,8)	-0.2092	3.1	!
! D41	D(6,5,7,8)	-0.2583	3.8	!
! D42	D(6,5,7,26)	-0.0637	0.9	!
! D43	D(5,7,8,9)	0.1766	2.6	!
! D44	D(5,7,8,12)	0.2407	3.5	!
! D46	D(26,7,8,12)	0.0457	0.7	!
! D47	D(7,8,9,10)	-0.0423	0.6	!
! D49	D(12,8,9,10)	-0.1118	1.6	!
! D50	D(12,8,9,27)	-0.0577	0.8	!
! D51	D(7,8,12,13)	0.1877	2.8	!
! D52	D(9,8,12,13)	0.2541	3.7	!
! D53	D(8,9,10,4)	-0.0659	1.0	!
! D55	D(27,9,10,4)	-0.1187	1.7	!
! D56	D(27,9,10,14)	-0.0668	1.0	!
! D58	D(9,10,14,28)	-0.0362	0.5	!
! D59	D(8,12,13,29)	-0.3231	4.7	!
! D60	D(8,12,13,30)	-0.3258	4.8	!
! D61	D(8,12,13,31)	-0.3266	4.8	!

! Normal Mode 16 !

! Name	Definition	Value	Relative Weight (%)	!
! R1	R(1,2)	-0.024	0.4	!
! R8	R(3,4)	0.0344	0.5	!
! A2	A(2,1,15)	-0.0618	0.9	!
! A4	A(6,1,15)	0.0276	0.4	!
! A7	A(1,2,3)	0.0267	0.4	!
! A14	A(2,3,11)	-0.0474	0.7	!

```
! A15   A(4,3,11)            0.0398        0.6        !
! A16   A(3,4,5)            -0.0254        0.4        !
! A17   A(3,4,10)            0.0754        1.1        !
! A18   A(5,4,10)           -0.0357        0.5        !
! A21   A(6,5,7)            -0.0314        0.5        !
! A26   A(7,8,9)            -0.0252        0.4        !
! A27   A(7,8,12)            0.0358        0.5        !
! A29   A(8,9,10)            0.0233        0.3        !
! A33   A(4,10,14)           0.033         0.5        !
! A34   A(9,10,14)          -0.0402        0.6        !
! D1    D(6,1,2,3)           0.0469        0.7        !
! D2    D(6,1,2,24)          0.069         1.0        !
! D3    D(6,1,2,25)          0.0556        0.8        !
! D4    D(15,1,2,3)          0.0457        0.7        !
! D5    D(15,1,2,24)         0.0678        1.0        !
! D6    D(15,1,2,25)         0.0545        0.8        !
! D7    D(23,1,2,3)          0.0639        0.9        !
! D8    D(23,1,2,24)         0.086         1.3        !
! D9    D(23,1,2,25)         0.0727        1.1        !
! D10   D(2,1,6,5)          -0.0429        0.6        !
! D11   D(15,1,6,5)         -0.095         1.4        !
! D12   D(23,1,6,5)         -0.0714        1.1        !
! D15   D(6,1,15,16)         0.0248        0.4        !
! D20   D(1,2,3,11)          0.0709        1.0        !
! D22   D(24,2,3,11)         0.0573        0.8        !
! D24   D(25,2,3,11)         0.0655        1.0        !
! D25   D(2,3,4,5)          -0.0803        1.2        !
! D26   D(2,3,4,10)          0.1484        2.2        !
! D27   D(11,3,4,5)         -0.1357        2.0        !
! D28   D(11,3,4,10)         0.093         1.4        !
! D29   D(3,4,5,6)           0.0977        1.4        !
! D30   D(3,4,5,7)           0.1219        1.8        !
! D31   D(10,4,5,6)         -0.1202        1.8        !
! D32   D(10,4,5,7)         -0.096         1.4        !
! D33   D(3,4,10,9)         -0.1485        2.2        !
! D34   D(3,4,10,14)        -0.1254        1.9        !
! D35   D(5,4,10,9)          0.0738        1.1        !
! D36   D(5,4,10,14)         0.0969        1.4        !
! D37   D(4,5,6,1)          -0.0318        0.5        !
! D38   D(7,5,6,1)          -0.0548        0.8        !
! D40   D(4,5,7,26)          0.0402        0.6        !
! D41   D(6,5,7,8)           0.0289        0.4        !
! D42   D(6,5,7,26)          0.0631        0.9        !
! D43   D(5,7,8,9)           0.1106        1.6        !
! D44   D(5,7,8,12)          0.1314        1.9        !
! D45   D(26,7,8,9)          0.0765        1.1        !
! D46   D(26,7,8,12)         0.0973        1.4        !
! D47   D(7,8,9,10)         -0.1318        1.9        !
! D48   D(7,8,9,27)         -0.0429        0.6        !
! D49   D(12,8,9,10)        -0.1547        2.3        !
```

! D50	D(12,8,9,27)	-0.0658	1.0	!
! D51	D(7,8,12,13)	0.0628	0.9	!
! D52	D(9,8,12,13)	0.0842	1.2	!
! D53	D(8,9,10,4)	0.0365	0.5	!
! D55	D(27,9,10,4)	-0.0504	0.7	!
! D56	D(27,9,10,14)	-0.0744	1.1	!
! D57	D(4,10,14,28)	-0.1614	2.4	!
! D58	D(9,10,14,28)	-0.1383	2.0	!
! D59	D(8,12,13,29)	-0.203	3.0	!
! D60	D(8,12,13,30)	-0.2056	3.0	!
! D61	D(8,12,13,31)	-0.2018	3.0	!
! D62	D(1,15,16,17)	0.0819	1.2	!
! D63	D(1,15,16,32)	0.027	0.4	!
! D64	D(20,15,16,17)	0.0978	1.4	!
! D65	D(20,15,16,32)	0.043	0.6	!
! D66	D(1,15,20,19)	-0.0789	1.2	!
! D68	D(16,15,20,19)	-0.0959	1.4	!
! D69	D(16,15,20,33)	-0.0377	0.6	!
! D70	D(15,16,17,18)	-0.024	0.4	!
! D71	D(15,16,17,22)	-0.0484	0.7	!
! D72	D(32,16,17,18)	0.0306	0.5	!
! D74	D(16,17,18,19)	-0.0499	0.7	!
! D75	D(16,17,18,34)	-0.0365	0.5	!
! D76	D(22,17,18,19)	-0.0268	0.4	!
! D79	D(18,17,22,35)	-0.0404	0.6	!
! D80	D(17,18,19,20)	0.0517	0.8	!
! D81	D(17,18,19,21)	0.0223	0.3	!
! D82	D(34,18,19,20)	0.038	0.6	!
! D85	D(18,19,20,33)	-0.0365	0.5	!
! D86	D(21,19,20,15)	0.0488	0.7	!
! D89	D(20,19,21,36)	-0.0237	0.4	!

! Normal Mode 17 !

! Name	Definition	Value	Relative Weight (%)	!
! R1	R(1,2)	0.0286	0.4	!
! R8	R(3,4)	-0.0328	0.4	!
! A1	A(2,1,6)	0.0469	0.6	!
! A2	A(2,1,15)	0.0284	0.4	!
! A4	A(6,1,15)	-0.0393	0.5	!
! A14	A(2,3,11)	0.037	0.5	!
! A15	A(4,3,11)	-0.0407	0.5	!
! A16	A(3,4,5)	0.0327	0.4	!
! A17	A(3,4,10)	-0.0425	0.6	!
! A18	A(5,4,10)	0.0276	0.4	!
! A21	A(6,5,7)	0.0242	0.3	!
! A22	A(1,6,5)	0.0298	0.4	!
! A33	A(4,10,14)	-0.0243	0.3	!

```
! A34   A(9,10,14)              0.0308                  0.4                     !
! A44   A(1,15,20)              0.0263                  0.3                     !
! D1    D(6,1,2,3)              0.0952                  1.3                     !
! D2    D(6,1,2,24)             0.1147                  1.5                     !
! D3    D(6,1,2,25)             0.1153                  1.5                     !
! D4    D(15,1,2,3)             0.093                   1.2                     !
! D5    D(15,1,2,24)            0.1125                  1.5                     !
! D6    D(15,1,2,25)            0.113                   1.5                     !
! D7    D(23,1,2,3)             0.1021                  1.3                     !
! D8    D(23,1,2,24)            0.1216                  1.6                     !
! D9    D(23,1,2,25)            0.1222                  1.6                     !
! D10   D(2,1,6,5)             -0.1192                  1.6                     !
! D11   D(15,1,6,5)            -0.0796                  1.0                     !
! D12   D(23,1,6,5)            -0.1139                  1.5                     !
! D15   D(6,1,15,16)           -0.0633                  0.8                     !
! D16   D(6,1,15,20)           -0.0473                  0.6                     !
! D17   D(23,1,15,16)          -0.0301                  0.4                     !
! D20   D(1,2,3,11)             0.082                   1.1                     !
! D22   D(24,2,3,11)            0.0573                  0.8                     !
! D24   D(25,2,3,11)            0.0519                  0.7                     !
! D25   D(2,3,4,5)             -0.1178                  1.5                     !
! D26   D(2,3,4,10)             0.1707                  2.2                     !
! D27   D(11,3,4,5)            -0.1844                  2.4                     !
! D28   D(11,3,4,10)            0.1041                  1.4                     !
! D29   D(3,4,5,6)              0.0881                  1.2                     !
! D30   D(3,4,5,7)              0.1329                  1.7                     !
! D31   D(10,4,5,6)            -0.1776                  2.3                     !
! D32   D(10,4,5,7)            -0.1328                  1.7                     !
! D33   D(3,4,10,9)            -0.1853                  2.4                     !
! D34   D(3,4,10,14)           -0.1533                  2.0                     !
! D35   D(5,4,10,9)             0.0951                  1.2                     !
! D36   D(5,4,10,14)            0.1271                  1.7                     !
! D37   D(4,5,6,1)              0.0383                  0.5                     !
! D39   D(4,5,7,8)              0.0305                  0.4                     !
! D40   D(4,5,7,26)             0.0473                  0.6                     !
! D41   D(6,5,7,8)              0.0722                  0.9                     !
! D42   D(6,5,7,26)             0.089                   1.2                     !
! D43   D(5,7,8,9)              0.1147                  1.5                     !
! D44   D(5,7,8,12)             0.1312                  1.7                     !
! D45   D(26,7,8,9)             0.0977                  1.3                     !
! D46   D(26,7,8,12)            0.1143                  1.5                     !
! D47   D(7,8,9,10)            -0.1509                  2.0                     !
! D48   D(7,8,9,27)            -0.0573                  0.8                     !
! D49   D(12,8,9,10)           -0.1686                  2.2                     !
! D50   D(12,8,9,27)           -0.075                   1.0                     !
! D51   D(7,8,12,13)            0.0552                  0.7                     !
! D52   D(9,8,12,13)            0.0724                  1.0                     !
! D53   D(8,9,10,4)             0.0427                  0.6                     !
! D55   D(27,9,10,4)           -0.0487                  0.6                     !
! D56   D(27,9,10,14)          -0.0809                  1.1                     !
```

D57	D(4,10,14,28)	-0.1946	2.6
D58	D(9,10,14,28)	-0.1633	2.1
D59	D(8,12,13,29)	-0.1921	2.5
D60	D(8,12,13,30)	-0.1966	2.6
D61	D(8,12,13,31)	-0.194	2.5
D62	D(1,15,16,17)	-0.0507	0.7
D64	D(20,15,16,17)	-0.0676	0.9
D65	D(20,15,16,32)	-0.0355	0.5
D66	D(1,15,20,19)	0.0524	0.7
D68	D(16,15,20,19)	0.0682	0.9
D69	D(16,15,20,33)	0.0291	0.4
D71	D(15,16,17,22)	0.0299	0.4
D74	D(16,17,18,19)	0.0337	0.4
D75	D(16,17,18,34)	0.0275	0.4
D78	D(16,17,22,35)	0.0597	0.8
D79	D(18,17,22,35)	0.073	1.0
D80	D(17,18,19,20)	-0.033	0.4
D82	D(34,18,19,20)	-0.0267	0.4
D86	D(21,19,20,15)	-0.0397	0.5

! Normal Mode 18 !

Name	Definition	Value	Relative Weight (%)
D14	D(2,1,15,20)	0.0155	0.6
D16	D(6,1,15,20)	0.0175	0.7
D63	D(1,15,16,32)	-0.028	1.1
D64	D(20,15,16,17)	-0.0222	0.9
D65	D(20,15,16,32)	-0.05	2.0
D68	D(16,15,20,19)	0.0229	0.9
D69	D(16,15,20,33)	0.0215	0.9
D71	D(15,16,17,22)	-0.0367	1.5
D72	D(32,16,17,18)	0.0277	1.1
D74	D(16,17,18,19)	0.0211	0.9
D75	D(16,17,18,34)	0.0409	1.7
D76	D(22,17,18,19)	0.0557	2.3
D77	D(22,17,18,34)	0.0755	3.1
D78	D(16,17,22,35)	0.6267	25.6
D79	D(18,17,22,35)	0.5914	24.2
D80	D(17,18,19,20)	-0.0204	0.8
D82	D(34,18,19,20)	-0.0407	1.7
D86	D(21,19,20,15)	-0.0281	1.2
D87	D(21,19,20,33)	-0.0268	1.1
D88	D(18,19,21,36)	-0.2443	10.0
D89	D(20,19,21,36)	-0.2173	8.9

! Normal Mode 19 !

Name	Definition	Value	Relative Weight (%)
A57	A(20,19,21)	-0.0074	0.3
D14	D(2,1,15,20)	-0.0113	0.5
D15	D(6,1,15,16)	-0.0077	0.3
D16	D(6,1,15,20)	-0.0121	0.5
D17	D(23,1,15,16)	-0.0075	0.3
D18	D(23,1,15,20)	-0.0118	0.5
D62	D(1,15,16,17)	-0.0111	0.5
D63	D(1,15,16,32)	-0.0153	0.6
D65	D(20,15,16,32)	-0.0111	0.5
D66	D(1,15,20,19)	-0.0095	0.4
D67	D(1,15,20,33)	-0.0139	0.6
D68	D(16,15,20,19)	-0.0138	0.6
D69	D(16,15,20,33)	-0.0182	0.8
D70	D(15,16,17,18)	0.023	1.0
D71	D(15,16,17,22)	-0.0145	0.6
D72	D(32,16,17,18)	0.0271	1.2
D73	D(32,16,17,22)	-0.0104	0.4
D74	D(16,17,18,19)	-0.0179	0.8
D75	D(16,17,18,34)	-0.0433	1.8
D76	D(22,17,18,19)	0.0176	0.7
D77	D(22,17,18,34)	-0.0079	0.3
D78	D(16,17,22,35)	0.2459	10.5
D79	D(18,17,22,35)	0.2097	8.9
D81	D(17,18,19,21)	-0.0355	1.5
D82	D(34,18,19,20)	0.0228	1.0
D83	D(34,18,19,21)	-0.0094	0.4
D84	D(18,19,20,15)	0.0191	0.8
D85	D(18,19,20,33)	0.0234	1.0
D86	D(21,19,20,15)	0.0497	2.1
D87	D(21,19,20,33)	0.0541	2.3
D88	D(18,19,21,36)	0.6242	26.5
D89	D(20,19,21,36)	0.5932	25.2

! Normal Mode 20 !

Name	Definition	Value	Relative Weight (%)
R1	R(1,2)	-0.0095	0.3
R2	R(1,6)	-0.0159	0.6
R3	R(1,15)	-0.0157	0.6
R5	R(2,3)	-0.0199	0.7
R12	R(5,6)	-0.0125	0.4
R13	R(5,7)	-0.0099	0.4
R14	R(7,8)	-0.0123	0.4
R26	R(15,16)	0.0161	0.6
R27	R(15,20)	0.0218	0.8
R30	R(17,18)	-0.0419	1.5

```
! R32   R(18,19)              -0.0416                  1.5                    !
! R35   R(19,21)              -0.0128                  0.5                    !
! A2    A(2,1,15)             -0.0291                  1.0                    !
! A3    A(2,1,23)              0.0096                  0.3                    !
! A6    A(15,1,23)             0.0139                  0.5                    !
! A13   A(2,3,4)              -0.017                   0.6                    !
! A15   A(4,3,11)              0.0203                  0.7                    !
! A20   A(4,5,7)               0.0134                  0.5                    !
! A21   A(6,5,7)              -0.0178                  0.6                    !
! A22   A(1,6,5)              -0.0224                  0.8                    !
! A33   A(4,10,14)            -0.0118                  0.4                    !
! A34   A(9,10,14)             0.0125                  0.4                    !
! A35   A(8,12,13)            -0.0272                  1.0                    !
! A43   A(1,15,16)            -0.0224                  0.8                    !
! A44   A(1,15,20)            -0.0225                  0.8                    !
! A45   A(16,15,20)            0.0444                  1.6                    !
! A46   A(15,16,17)           -0.0422                  1.5                    !
! A48   A(17,16,32)            0.0347                  1.2                    !
! A50   A(16,17,22)            0.1268                  4.5                    !
! A51   A(18,17,22)           -0.119                   4.2                    !
! A52   A(17,18,19)            0.0588                  2.1                    !
! A53   A(17,18,34)           -0.0363                  1.3                    !
! A54   A(19,18,34)           -0.0225                  0.8                    !
! A55   A(18,19,20)           -0.0188                  0.7                    !
! A56   A(18,19,21)           -0.1175                  4.2                    !
! A57   A(20,19,21)            0.1363                  4.8                    !
! A58   A(15,20,19)           -0.0341                  1.2                    !
! A60   A(19,20,33)            0.0383                  1.4                    !
! A61   A(19,21,36)           -0.0387                  1.4                    !
! A62   A(17,22,35)            0.0395                  1.4                    !
! D1    D(6,1,2,3)            -0.0282                  1.0                    !
! D2    D(6,1,2,24)           -0.0288                  1.0                    !
! D3    D(6,1,2,25)           -0.0349                  1.2                    !
! D4    D(15,1,2,3)           -0.0089                  0.3                    !
! D5    D(15,1,2,24)          -0.0095                  0.3                    !
! D6    D(15,1,2,25)          -0.0157                  0.6                    !
! D7    D(23,1,2,3)           -0.014                   0.5                    !
! D8    D(23,1,2,24)          -0.0146                  0.5                    !
! D9    D(23,1,2,25)          -0.0207                  0.7                    !
! D10   D(2,1,6,5)             0.0209                  0.7                    !
! D11   D(15,1,6,5)           -0.0131                  0.5                    !
! D15   D(6,1,15,16)           0.0171                  0.6                    !
! D22   D(24,2,3,11)           0.009                   0.3                    !
! D37   D(4,5,6,1)            -0.0102                  0.4                    !
! D38   D(7,5,6,1)            -0.0161                  0.6                    !
! D39   D(4,5,7,8)            -0.0099                  0.3                    !
! D44   D(5,7,8,12)            0.0096                  0.3                    !
! D59   D(8,12,13,29)         -0.0093                  0.3                    !
! D62   D(1,15,16,17)          0.0253                  0.9                    !
! D64   D(20,15,16,17)         0.0365                  1.3                    !
```

D65	D(20,15,16,32)	0.0122	0.4
D66	D(1,15,20,19)	-0.0293	1.0
D68	D(16,15,20,19)	-0.0407	1.4
D69	D(16,15,20,33)	-0.0183	0.6
D71	D(15,16,17,22)	-0.0281	1.0
D72	D(32,16,17,18)	0.0205	0.7
D74	D(16,17,18,19)	-0.024	0.8
D75	D(16,17,18,34)	-0.0255	0.9
D78	D(16,17,22,35)	0.1018	3.6
D79	D(18,17,22,35)	0.0789	2.8
D80	D(17,18,19,20)	0.0194	0.7
D82	D(34,18,19,20)	0.021	0.7
D84	D(18,19,20,15)	0.0126	0.4
D85	D(18,19,20,33)	-0.01	0.4
D86	D(21,19,20,15)	0.0309	1.1
D88	D(18,19,21,36)	0.1322	4.7
D89	D(20,19,21,36)	0.1137	4.0

! Normal Mode 21 !

Name	Definition	Value	Relative Weight (%)
R8	R(3,4)	-0.0772	1.6
R13	R(5,7)	-0.061	1.2
R14	R(7,8)	-0.0409	0.8
R17	R(8,12)	-0.0389	0.8
R18	R(9,10)	-0.0169	0.3
R21	R(12,13)	-0.0435	0.9
A1	A(2,1,6)	-0.053	1.1
A2	A(2,1,15)	-0.0347	0.7
A3	A(2,1,23)	0.0339	0.7
A4	A(6,1,15)	0.0447	0.9
A7	A(1,2,3)	0.0439	0.9
A8	A(1,2,24)	-0.0394	0.8
A10	A(3,2,24)	-0.0178	0.4
A14	A(2,3,11)	0.03	0.6
A15	A(4,3,11)	-0.0281	0.6
A16	A(3,4,5)	-0.0529	1.1
A17	A(3,4,10)	-0.0166	0.3
A18	A(5,4,10)	0.0727	1.5
A19	A(4,5,6)	0.0732	1.5
A20	A(4,5,7)	-0.033	0.7
A21	A(6,5,7)	-0.0401	0.8
A22	A(1,6,5)	-0.0416	0.8
A23	A(5,7,8)	-0.0281	0.6
A24	A(5,7,26)	0.0347	0.7
A26	A(7,8,9)	0.0792	1.6
A27	A(7,8,12)	-0.0903	1.8
A29	A(8,9,10)	-0.0339	0.7

```
! A31   A(10,9,27)            0.0221              0.4                 !
! A32   A(4,10,9)            -0.0572              1.2                 !
! A33   A(4,10,14)            0.0509              1.0                 !
! A35   A(8,12,13)           -0.0752              1.5                 !
! A37   A(12,13,30)           0.0176              0.4                 !
! A43   A(1,15,16)            0.0205              0.4                 !
! A56   A(18,19,21)           0.0275              0.6                 !
! A57   A(20,19,21)          -0.0308              0.6                 !
! D1    D(6,1,2,3)           -0.0362              0.7                 !
! D2    D(6,1,2,24)          -0.0565              1.2                 !
! D3    D(6,1,2,25)          -0.0788              1.6                 !
! D4    D(15,1,2,3)          -0.0322              0.7                 !
! D5    D(15,1,2,24)         -0.0525              1.1                 !
! D6    D(15,1,2,25)         -0.0748              1.5                 !
! D7    D(23,1,2,3)          -0.039               0.8                 !
! D8    D(23,1,2,24)         -0.0593              1.2                 !
! D9    D(23,1,2,25)         -0.0816              1.7                 !
! D11   D(15,1,6,5)          -0.0417              0.8                 !
! D15   D(6,1,15,16)          0.0529              1.1                 !
! D16   D(6,1,15,20)          0.0434              0.9                 !
! D17   D(23,1,15,16)         0.0177              0.4                 !
! D19   D(1,2,3,4)            0.0406              0.8                 !
! D20   D(1,2,3,11)           0.027               0.5                 !
! D21   D(24,2,3,4)           0.074               1.5                 !
! D22   D(24,2,3,11)          0.0603              1.2                 !
! D23   D(25,2,3,4)           0.086               1.8                 !
! D24   D(25,2,3,11)          0.0723              1.5                 !
! D25   D(2,3,4,5)           -0.0243              0.5                 !
! D26   D(2,3,4,10)           0.0294              0.6                 !
! D28   D(11,3,4,10)          0.0426              0.9                 !
! D29   D(3,4,5,6)            0.0382              0.8                 !
! D30   D(3,4,5,7)            0.051               1.0                 !
! D33   D(3,4,10,9)          -0.036               0.7                 !
! D34   D(3,4,10,14)         -0.0341              0.7                 !
! D37   D(4,5,6,1)           -0.0467              1.0                 !
! D38   D(7,5,6,1)           -0.0586              1.2                 !
! D39   D(4,5,7,8)           -0.0271              0.6                 !
! D43   D(5,7,8,9)            0.0338              0.7                 !
! D44   D(5,7,8,12)           0.0442              0.9                 !
! D46   D(26,7,8,12)          0.0233              0.5                 !
! D47   D(7,8,9,10)          -0.0185              0.4                 !
! D49   D(12,8,9,10)         -0.029               0.6                 !
! D51   D(7,8,12,13)          0.0155              0.3                 !
! D52   D(9,8,12,13)          0.0268              0.5                 !
! D55   D(27,9,10,4)         -0.0216              0.4                 !
! D56   D(27,9,10,14)        -0.0238              0.5                 !
! D57   D(4,10,14,28)        -0.1172              2.4                 !
! D58   D(9,10,14,28)        -0.1157              2.4                 !
! D59   D(8,12,13,29)        -0.0366              0.7                 !
! D60   D(8,12,13,30)        -0.0306              0.6                 !
```

D61	D(8,12,13,31)	-0.0238	0.5
D62	D(1,15,16,17)	0.0787	1.6
D63	D(1,15,16,32)	0.0264	0.5
D64	D(20,15,16,17)	0.0882	1.8
D65	D(20,15,16,32)	0.036	0.7
D66	D(1,15,20,19)	-0.08	1.6
D67	D(1,15,20,33)	-0.0245	0.5
D68	D(16,15,20,19)	-0.0888	1.8
D69	D(16,15,20,33)	-0.0334	0.7
D70	D(15,16,17,18)	-0.0218	0.4
D71	D(15,16,17,22)	-0.055	1.1
D72	D(32,16,17,18)	0.0303	0.6
D74	D(16,17,18,19)	-0.0424	0.9
D75	D(16,17,18,34)	-0.038	0.8
D78	D(16,17,22,35)	0.0441	0.9
D80	D(17,18,19,20)	0.0417	0.8
D82	D(34,18,19,20)	0.0372	0.8
D84	D(18,19,20,15)	0.0236	0.5
D85	D(18,19,20,33)	-0.0308	0.6
D86	D(21,19,20,15)	0.0566	1.2
D88	D(18,19,21,36)	0.03	0.6

! Normal Mode 22 !

Name	Definition	Value	Relative Weight (%)
A10	A(3,2,24)	0.0133	0.5
A11	A(3,2,25)	-0.009	0.3
A13	A(2,3,4)	0.0135	0.5
A15	A(4,3,11)	-0.0104	0.4
A33	A(4,10,14)	0.0089	0.3
D1	D(6,1,2,3)	0.0211	0.7
D2	D(6,1,2,24)	0.0383	1.4
D3	D(6,1,2,25)	0.0385	1.4
D4	D(15,1,2,3)	0.022	0.8
D5	D(15,1,2,24)	0.0392	1.4
D6	D(15,1,2,25)	0.0394	1.4
D7	D(23,1,2,3)	0.0238	0.8
D8	D(23,1,2,24)	0.041	1.4
D9	D(23,1,2,25)	0.0412	1.5
D19	D(1,2,3,4)	-0.0111	0.4
D20	D(1,2,3,11)	0.0288	1.0
D21	D(24,2,3,4)	-0.0229	0.8
D22	D(24,2,3,11)	0.0171	0.6
D23	D(25,2,3,4)	-0.026	0.9
D24	D(25,2,3,11)	0.0139	0.5
D26	D(2,3,4,10)	0.0383	1.4
D27	D(11,3,4,5)	-0.0505	1.8
D29	D(3,4,5,6)	0.0205	0.7

D30	D(3,4,5,7)	0.017	0.6
D31	D(10,4,5,6)	-0.0226	0.8
D32	D(10,4,5,7)	-0.0261	0.9
D33	D(3,4,10,9)	-0.0229	0.8
D34	D(3,4,10,14)	0.0178	0.6
D35	D(5,4,10,9)	0.0216	0.8
D36	D(5,4,10,14)	0.0624	2.2
D37	D(4,5,6,1)	-0.0138	0.5
D38	D(7,5,6,1)	-0.0105	0.4
D43	D(5,7,8,9)	0.0331	1.2
D44	D(5,7,8,12)	0.0135	0.5
D45	D(26,7,8,9)	0.0273	1.0
D47	D(7,8,9,10)	-0.0373	1.3
D48	D(7,8,9,27)	-0.0938	3.3
D49	D(12,8,9,10)	-0.016	0.6
D50	D(12,8,9,27)	-0.0725	2.6
D51	D(7,8,12,13)	0.0124	0.4
D53	D(8,9,10,4)	0.0092	0.3
D54	D(8,9,10,14)	-0.0324	1.1
D55	D(27,9,10,4)	0.0643	2.3
D56	D(27,9,10,14)	0.0228	0.8
D57	D(4,10,14,28)	0.6218	22.0
D58	D(9,10,14,28)	0.662	23.4
D59	D(8,12,13,29)	-0.0101	0.4
D60	D(8,12,13,30)	-0.011	0.4
D61	D(8,12,13,31)	-0.0098	0.3

! Normal Mode 23 !

Name	Definition	Value	Relative Weight (%)
R3	R(1,15)	0.0534	1.2
R8	R(3,4)	0.0503	1.1
R11	R(4,10)	-0.0347	0.8
R14	R(7,8)	-0.02	0.4
R16	R(8,9)	0.0371	0.8
R18	R(9,10)	0.0224	0.5
R20	R(10,14)	-0.016	0.4
R26	R(15,16)	0.0151	0.3
R28	R(16,17)	0.0146	0.3
A1	A(2,1,6)	-0.02	0.4
A4	A(6,1,15)	0.0199	0.4
A6	A(15,1,23)	-0.0165	0.4
A7	A(1,2,3)	0.0795	1.8
A8	A(1,2,24)	-0.0268	0.6
A10	A(3,2,24)	-0.0445	1.0
A13	A(2,3,4)	-0.0962	2.1
A14	A(2,3,11)	-0.036	0.8
A15	A(4,3,11)	0.1309	2.9

!	A16	A(3,4,5)	0.0292	0.6	!
!	A17	A(3,4,10)	0.0152	0.3	!
!	A18	A(5,4,10)	-0.0408	0.9	!
!	A19	A(4,5,6)	0.0339	0.8	!
!	A20	A(4,5,7)	0.0145	0.3	!
!	A21	A(6,5,7)	-0.0482	1.1	!
!	A22	A(1,6,5)	-0.0189	0.4	!
!	A23	A(5,7,8)	0.0235	0.5	!
!	A24	A(5,7,26)	-0.027	0.6	!
!	A26	A(7,8,9)	-0.0226	0.5	!
!	A28	A(9,8,12)	0.0159	0.4	!
!	A29	A(8,9,10)	-0.0292	0.6	!
!	A30	A(8,9,27)	0.0317	0.7	!
!	A32	A(4,10,9)	0.0542	1.2	!
!	A33	A(4,10,14)	-0.1815	4.0	!
!	A34	A(9,10,14)	0.1276	2.8	!
!	A35	A(8,12,13)	-0.1429	3.2	!
!	A36	A(12,13,29)	-0.0148	0.3	!
!	A37	A(12,13,30)	0.0309	0.7	!
!	A38	A(12,13,31)	-0.0165	0.4	!
!	A42	A(10,14,28)	0.0555	1.2	!
!	A43	A(1,15,16)	0.0297	0.7	!
!	A44	A(1,15,20)	0.0209	0.5	!
!	A45	A(16,15,20)	-0.0495	1.1	!
!	A46	A(15,16,17)	0.0271	0.6	!
!	A48	A(17,16,32)	-0.0222	0.5	!
!	A52	A(17,18,19)	-0.0307	0.7	!
!	A54	A(19,18,34)	0.0167	0.4	!
!	A56	A(18,19,21)	0.023	0.5	!
!	A57	A(20,19,21)	-0.0366	0.8	!
!	A58	A(15,20,19)	0.0294	0.7	!
!	A60	A(19,20,33)	-0.0286	0.6	!
!	D1	D(6,1,2,3)	-0.0254	0.6	!
!	D2	D(6,1,2,24)	-0.0483	1.1	!
!	D3	D(6,1,2,25)	-0.0792	1.8	!
!	D4	D(15,1,2,3)	-0.0448	1.0	!
!	D5	D(15,1,2,24)	-0.0677	1.5	!
!	D6	D(15,1,2,25)	-0.0986	2.2	!
!	D7	D(23,1,2,3)	-0.0373	0.8	!
!	D8	D(23,1,2,24)	-0.0602	1.3	!
!	D9	D(23,1,2,25)	-0.0911	2.0	!
!	D10	D(2,1,6,5)	-0.046	1.0	!
!	D11	D(15,1,6,5)	-0.0308	0.7	!
!	D12	D(23,1,6,5)	-0.0419	0.9	!
!	D14	D(2,1,15,20)	0.0173	0.4	!
!	D16	D(6,1,15,20)	0.0205	0.5	!
!	D18	D(23,1,15,20)	0.0232	0.5	!
!	D19	D(1,2,3,4)	0.0391	0.9	!
!	D21	D(24,2,3,4)	0.0522	1.2	!
!	D23	D(25,2,3,4)	0.0873	1.9	!

D24	D(25,2,3,11)	0.0433	1.0
D25	D(2,3,4,5)	-0.0394	0.9
D26	D(2,3,4,10)	0.018	0.4
D28	D(11,3,4,10)	0.068	1.5
D29	D(3,4,5,6)	0.0334	0.7
D30	D(3,4,5,7)	0.0556	1.2
D31	D(10,4,5,6)	-0.0223	0.5
D33	D(3,4,10,9)	-0.0561	1.2
D34	D(3,4,10,14)	-0.0287	0.6
D36	D(5,4,10,14)	0.0298	0.7
D38	D(7,5,6,1)	-0.0224	0.5
D39	D(4,5,7,8)	-0.0155	0.3
D42	D(6,5,7,26)	0.0157	0.3
D43	D(5,7,8,9)	0.0297	0.7
D44	D(5,7,8,12)	0.035	0.8
D45	D(26,7,8,9)	0.0201	0.4
D46	D(26,7,8,12)	0.0253	0.6
D47	D(7,8,9,10)	-0.0275	0.6
D48	D(7,8,9,27)	-0.0279	0.6
D49	D(12,8,9,10)	-0.0332	0.7
D50	D(12,8,9,27)	-0.0335	0.7
D52	D(9,8,12,13)	0.0149	0.3
D54	D(8,9,10,14)	-0.0147	0.3
D56	D(27,9,10,14)	-0.0143	0.3
D57	D(4,10,14,28)	0.1378	3.1
D58	D(9,10,14,28)	0.1642	3.6
D59	D(8,12,13,29)	-0.0377	0.8
D60	D(8,12,13,30)	-0.0248	0.6
D62	D(1,15,16,17)	0.0187	0.4
D63	D(1,15,16,32)	0.0211	0.5
D66	D(1,15,20,19)	-0.0193	0.4
D67	D(1,15,20,33)	-0.0204	0.5

! Normal Mode 24 !

Name	Definition	Value	Relative Weight (%)
R1	R(1,2)	-0.0858	1.3
R9	R(3,11)	-0.0219	0.3
R10	R(4,5)	-0.073	1.1
R16	R(8,9)	0.0486	0.7
R18	R(9,10)	0.0238	0.4
A1	A(2,1,6)	-0.097	1.4
A2	A(2,1,15)	0.0235	0.4
A3	A(2,1,23)	0.0331	0.5
A5	A(6,1,23)	0.0299	0.4
A7	A(1,2,3)	-0.0841	1.3
A9	A(1,2,25)	0.0641	1.0
A10	A(3,2,24)	0.0616	0.9

!	A11	A(3,2,25)	-0.0733	1.1	!
!	A13	A(2,3,4)	0.1889	2.8	!
!	A14	A(2,3,11)	-0.1515	2.3	!
!	A15	A(4,3,11)	-0.0348	0.5	!
!	A16	A(3,4,5)	-0.123	1.8	!
!	A17	A(3,4,10)	0.057	0.9	!
!	A18	A(5,4,10)	0.063	0.9	!
!	A19	A(4,5,6)	-0.0696	1.0	!
!	A21	A(6,5,7)	0.0754	1.1	!
!	A22	A(1,6,5)	0.1387	2.1	!
!	A23	A(5,7,8)	-0.0229	0.3	!
!	A24	A(5,7,26)	0.0282	0.4	!
!	A26	A(7,8,9)	0.0336	0.5	!
!	A27	A(7,8,12)	-0.0292	0.4	!
!	A29	A(8,9,10)	-0.0308	0.5	!
!	A32	A(4,10,9)	-0.0364	0.5	!
!	A33	A(4,10,14)	-0.0754	1.1	!
!	A34	A(9,10,14)	0.1114	1.7	!
!	A35	A(8,12,13)	-0.0982	1.5	!
!	A37	A(12,13,30)	0.0234	0.3	!
!	A42	A(10,14,28)	0.033	0.5	!
!	A43	A(1,15,16)	-0.0234	0.4	!
!	A50	A(16,17,22)	-0.045	0.7	!
!	A51	A(18,17,22)	0.0551	0.8	!
!	A56	A(18,19,21)	-0.0541	0.8	!
!	A57	A(20,19,21)	0.0511	0.8	!
!	A60	A(19,20,33)	0.0213	0.3	!
!	D1	D(6,1,2,3)	0.0621	0.9	!
!	D2	D(6,1,2,24)	0.0956	1.4	!
!	D3	D(6,1,2,25)	0.1695	2.5	!
!	D4	D(15,1,2,3)	0.1246	1.9	!
!	D5	D(15,1,2,24)	0.1581	2.4	!
!	D6	D(15,1,2,25)	0.232	3.5	!
!	D7	D(23,1,2,3)	0.0616	0.9	!
!	D8	D(23,1,2,24)	0.0951	1.4	!
!	D9	D(23,1,2,25)	0.169	2.5	!
!	D10	D(2,1,6,5)	0.1325	2.0	!
!	D11	D(15,1,6,5)	0.095	1.4	!
!	D12	D(23,1,6,5)	0.1289	1.9	!
!	D13	D(2,1,15,16)	-0.0436	0.7	!
!	D14	D(2,1,15,20)	-0.0778	1.2	!
!	D15	D(6,1,15,16)	0.0692	1.0	!
!	D16	D(6,1,15,20)	0.0351	0.5	!
!	D17	D(23,1,15,16)	0.0277	0.4	!
!	D19	D(1,2,3,4)	-0.0234	0.3	!
!	D20	D(1,2,3,11)	0.0687	1.0	!
!	D21	D(24,2,3,4)	-0.03	0.4	!
!	D22	D(24,2,3,11)	0.0621	0.9	!
!	D23	D(25,2,3,4)	-0.0476	0.7	!
!	D24	D(25,2,3,11)	0.0444	0.7	!

D25	D(2,3,4,5)	0.0467	0.7
D27	D(11,3,4,5)	-0.0488	0.7
D28	D(11,3,4,10)	-0.0948	1.4
D30	D(3,4,5,7)	-0.0733	1.1
D31	D(10,4,5,6)	0.0506	0.8
D32	D(10,4,5,7)	-0.0308	0.5
D33	D(3,4,10,9)	0.0651	1.0
D34	D(3,4,10,14)	0.0334	0.5
D37	D(4,5,6,1)	-0.1128	1.7
D38	D(7,5,6,1)	-0.0363	0.5
D39	D(4,5,7,8)	0.0449	0.7
D40	D(4,5,7,26)	0.0371	0.6
D41	D(6,5,7,8)	-0.0323	0.5
D42	D(6,5,7,26)	-0.04	0.6
D43	D(5,7,8,9)	-0.0415	0.6
D44	D(5,7,8,12)	-0.0514	0.8
D45	D(26,7,8,9)	-0.034	0.5
D46	D(26,7,8,12)	-0.0439	0.7
D47	D(7,8,9,10)	0.025	0.4
D48	D(7,8,9,27)	0.0286	0.4
D49	D(12,8,9,10)	0.0358	0.5
D50	D(12,8,9,27)	0.0395	0.6
D52	D(9,8,12,13)	-0.0222	0.3
D54	D(8,9,10,14)	0.0234	0.3
D57	D(4,10,14,28)	-0.1127	1.7
D58	D(9,10,14,28)	-0.145	2.2
D60	D(8,12,13,30)	0.0216	0.3
D61	D(8,12,13,31)	0.031	0.5
D62	D(1,15,16,17)	-0.0422	0.6
D63	D(1,15,16,32)	-0.0299	0.4
D66	D(1,15,20,19)	0.0443	0.7
D67	D(1,15,20,33)	0.0326	0.5

! Normal Mode 25 !

Name	Definition	Value	Relative Weight (%)
R26	R(15,16)	0.0251	0.4
R27	R(15,20)	-0.0271	0.4
A1	A(2,1,6)	0.1183	1.7
A3	A(2,1,23)	-0.0866	1.3
A4	A(6,1,15)	0.0342	0.5
A5	A(6,1,23)	-0.0432	0.6
A6	A(15,1,23)	-0.0404	0.6
A7	A(1,2,3)	-0.0364	0.5
A8	A(1,2,24)	0.1138	1.7
A9	A(1,2,25)	-0.0817	1.2
A10	A(3,2,24)	0.0606	0.9
A11	A(3,2,25)	-0.0497	0.7

```
! A13   A(2,3,4)                0.0517                  0.8                    !
! A14   A(2,3,11)              -0.0397                  0.6                    !
! A27   A(7,8,12)              -0.0381                  0.6                    !
! A28   A(9,8,12)               0.0309                  0.5                    !
! A35   A(8,12,13)             -0.0591                  0.9                    !
! A48   A(17,16,32)             0.0254                  0.4                    !
! A50   A(16,17,22)             0.0937                  1.4                    !
! A51   A(18,17,22)            -0.0906                  1.3                    !
! A53   A(17,18,34)            -0.0239                  0.3                    !
! A56   A(18,19,21)             0.09                    1.3                    !
! A57   A(20,19,21)            -0.0897                  1.3                    !
! A61   A(19,21,36)             0.0299                  0.4                    !
! A62   A(17,22,35)             0.0306                  0.4                    !
! D1    D(6,1,2,3)              0.1385                  2.0                    !
! D2    D(6,1,2,24)             0.2656                  3.9                    !
! D3    D(6,1,2,25)             0.2838                  4.1                    !
! D5    D(15,1,2,24)            0.1274                  1.9                    !
! D6    D(15,1,2,25)            0.1455                  2.1                    !
! D7    D(23,1,2,3)             0.1044                  1.5                    !
! D8    D(23,1,2,24)            0.2316                  3.4                    !
! D9    D(23,1,2,25)            0.2497                  3.6                    !
! D10   D(2,1,6,5)             -0.1232                  1.8                    !
! D12   D(23,1,6,5)            -0.0598                  0.9                    !
! D13   D(2,1,15,16)            0.0653                  1.0                    !
! D14   D(2,1,15,20)            0.0877                  1.3                    !
! D15   D(6,1,15,16)           -0.1192                  1.7                    !
! D16   D(6,1,15,20)           -0.0969                  1.4                    !
! D17   D(23,1,15,16)          -0.0646                  0.9                    !
! D18   D(23,1,15,20)          -0.0423                  0.6                    !
! D19   D(1,2,3,4)             -0.111                   1.6                    !
! D20   D(1,2,3,11)            -0.0413                  0.6                    !
! D21   D(24,2,3,4)            -0.2691                  3.9                    !
! D22   D(24,2,3,11)           -0.1994                  2.9                    !
! D23   D(25,2,3,4)            -0.2711                  4.0                    !
! D24   D(25,2,3,11)           -0.2014                  2.9                    !
! D25   D(2,3,4,5)              0.0353                  0.5                    !
! D27   D(11,3,4,5)            -0.038                   0.6                    !
! D28   D(11,3,4,10)           -0.0808                  1.2                    !
! D29   D(3,4,5,6)             -0.0464                  0.7                    !
! D30   D(3,4,5,7)             -0.0625                  0.9                    !
! D32   D(10,4,5,7)            -0.0232                  0.3                    !
! D33   D(3,4,10,9)             0.0492                  0.7                    !
! D34   D(3,4,10,14)            0.0287                  0.4                    !
! D37   D(4,5,6,1)              0.1198                  1.7                    !
! D38   D(7,5,6,1)              0.1348                  2.0                    !
! D39   D(4,5,7,8)              0.0331                  0.5                    !
! D43   D(5,7,8,9)             -0.0272                  0.4                    !
! D44   D(5,7,8,12)            -0.0383                  0.6                    !
! D46   D(26,7,8,12)           -0.0226                  0.3                    !
! D49   D(12,8,9,10)            0.0257                  0.4                    !
```

D50	D(12,8,9,27)	0.027	0.4
D57	D(4,10,14,28)	-0.0532	0.8
D58	D(9,10,14,28)	-0.0733	1.1
D61	D(8,12,13,31)	0.0223	0.3
D64	D(20,15,16,17)	-0.0434	0.6
D65	D(20,15,16,32)	-0.0275	0.4
D68	D(16,15,20,19)	0.0401	0.6
D71	D(15,16,17,22)	0.0281	0.4
D74	D(16,17,18,19)	0.0235	0.3
D75	D(16,17,18,34)	0.0255	0.4
D80	D(17,18,19,20)	-0.0269	0.4
D82	D(34,18,19,20)	-0.0287	0.4
D85	D(18,19,20,33)	0.0226	0.3
D86	D(21,19,20,15)	-0.0325	0.5

! Normal Mode 26 !

Name	Definition	Value	Relative Weight (%)
R2	R(1,6)	-0.0468	0.6
R3	R(1,15)	0.0229	0.3
R5	R(2,3)	0.0288	0.4
R10	R(4,5)	-0.0272	0.4
R12	R(5,6)	-0.0232	0.3
R16	R(8,9)	0.046	0.6
R17	R(8,12)	0.0439	0.6
R18	R(9,10)	0.0276	0.4
R20	R(10,14)	0.0373	0.5
R21	R(12,13)	0.0278	0.4
R31	R(17,22)	-0.0453	0.6
R32	R(18,19)	-0.0312	0.4
R34	R(19,20)	0.0257	0.4
R35	R(19,21)	0.0316	0.4
A1	A(2,1,6)	-0.0295	0.4
A2	A(2,1,15)	-0.0434	0.6
A4	A(6,1,15)	0.0316	0.4
A7	A(1,2,3)	-0.05	0.7
A8	A(1,2,24)	-0.0407	0.6
A9	A(1,2,25)	0.0343	0.5
A11	A(3,2,25)	0.0409	0.6
A14	A(2,3,11)	0.0933	1.3
A15	A(4,3,11)	-0.1144	1.6
A19	A(4,5,6)	-0.0503	0.7
A20	A(4,5,7)	0.1087	1.5
A21	A(6,5,7)	-0.0587	0.8
A22	A(1,6,5)	0.047	0.6
A23	A(5,7,8)	-0.0536	0.7
A25	A(8,7,26)	0.0734	1.0
A26	A(7,8,9)	-0.0671	0.9

```
! A27   A(7,8,12)              0.0526                 0.7                    !
! A29   A(8,9,10)              0.1187                 1.6                    !
! A30   A(8,9,27)             -0.0686                 0.9                    !
! A31   A(10,9,27)            -0.0501                 0.7                    !
! A32   A(4,10,9)             -0.0868                 1.2                    !
! A33   A(4,10,14)             0.0264                 0.4                    !
! A34   A(9,10,14)             0.0604                 0.8                    !
! A43   A(1,15,16)            -0.0315                 0.4                    !
! A44   A(1,15,20)             0.0717                 1.0                    !
! A45   A(16,15,20)           -0.0478                 0.7                    !
! A46   A(15,16,17)           -0.0981                 1.4                    !
! A47   A(15,16,32)            0.0673                 0.9                    !
! A48   A(17,16,32)            0.0305                 0.4                    !
! A49   A(16,17,18)            0.1552                 2.1                    !
! A51   A(18,17,22)           -0.1531                 2.1                    !
! A52   A(17,18,19)           -0.0493                 0.7                    !
! A53   A(17,18,34)           -0.0487                 0.7                    !
! A54   A(19,18,34)            0.0979                 1.4                    !
! A55   A(18,19,20)           -0.099                  1.4                    !
! A56   A(18,19,21)            0.1291                 1.8                    !
! A57   A(20,19,21)           -0.03                   0.4                    !
! A58   A(15,20,19)            0.1399                 1.9                    !
! A59   A(15,20,33)           -0.0614                 0.8                    !
! A60   A(19,20,33)           -0.0795                 1.1                    !
! A62   A(17,22,35)            0.0348                 0.5                    !
! D2    D(6,1,2,24)           -0.0678                 0.9                    !
! D3    D(6,1,2,25)           -0.0618                 0.9                    !
! D5    D(15,1,2,24)          -0.0581                 0.8                    !
! D6    D(15,1,2,25)          -0.052                  0.7                    !
! D8    D(23,1,2,24)          -0.0491                 0.7                    !
! D9    D(23,1,2,25)          -0.0431                 0.6                    !
! D10   D(2,1,6,5)             0.0908                 1.3                    !
! D11   D(15,1,6,5)            0.0386                 0.5                    !
! D12   D(23,1,6,5)            0.0704                 1.0                    !
! D13   D(2,1,15,16)           0.0753                 1.0                    !
! D14   D(2,1,15,20)          -0.0992                 1.4                    !
! D15   D(6,1,15,16)           0.1189                 1.6                    !
! D16   D(6,1,15,20)          -0.0556                 0.8                    !
! D17   D(23,1,15,16)          0.0762                 1.1                    !
! D18   D(23,1,15,20)         -0.0984                 1.4                    !
! D20   D(1,2,3,11)            0.0259                 0.4                    !
! D21   D(24,2,3,4)            0.0761                 1.1                    !
! D22   D(24,2,3,11)           0.1017                 1.4                    !
! D23   D(25,2,3,4)            0.0388                 0.5                    !
! D24   D(25,2,3,11)           0.0644                 0.9                    !
! D28   D(11,3,4,10)          -0.0266                 0.4                    !
! D30   D(3,4,5,7)            -0.0265                 0.4                    !
! D37   D(4,5,6,1)            -0.0617                 0.9                    !
! D38   D(7,5,6,1)            -0.0357                 0.5                    !
! D62   D(1,15,16,17)         -0.0273                 0.4                    !
```

D63	D(1,15,16,32)	-0.0785	1.1
D64	D(20,15,16,17)	0.143	2.0
D65	D(20,15,16,32)	0.0919	1.3
D66	D(1,15,20,19)	0.0303	0.4
D67	D(1,15,20,33)	0.0855	1.2
D68	D(16,15,20,19)	-0.1466	2.0
D69	D(16,15,20,33)	-0.0914	1.3
D71	D(15,16,17,22)	-0.1152	1.6
D72	D(32,16,17,18)	0.0294	0.4
D73	D(32,16,17,22)	-0.0644	0.9
D74	D(16,17,18,19)	-0.0924	1.3
D75	D(16,17,18,34)	-0.1083	1.5
D78	D(16,17,22,35)	0.0788	1.1
D80	D(17,18,19,20)	0.0885	1.2
D82	D(34,18,19,20)	0.1057	1.5
D83	D(34,18,19,21)	0.0233	0.3
D84	D(18,19,20,15)	0.0307	0.4
D85	D(18,19,20,33)	-0.0234	0.3
D86	D(21,19,20,15)	0.1092	1.5
D87	D(21,19,20,33)	0.0551	0.8
D88	D(18,19,21,36)	0.0328	0.5
D89	D(20,19,21,36)	-0.0464	0.6

! Normal Mode 27 !

Name	Definition	Value	Relative Weight (%)
R1	R(1,2)	0.0428	0.6
R5	R(2,3)	0.0485	0.7
R10	R(4,5)	-0.0227	0.3
R12	R(5,6)	-0.024	0.3
R14	R(7,8)	-0.0312	0.4
R16	R(8,9)	0.0442	0.6
R17	R(8,12)	0.0406	0.6
R18	R(9,10)	0.0349	0.5
R20	R(10,14)	0.0335	0.5
R26	R(15,16)	0.0338	0.5
R27	R(15,20)	-0.0302	0.4
R28	R(16,17)	0.0327	0.5
R30	R(17,18)	-0.0321	0.5
R31	R(17,22)	0.042	0.6
R35	R(19,21)	-0.0605	0.8
A1	A(2,1,6)	0.024	0.3
A2	A(2,1,15)	-0.0586	0.8
A6	A(15,1,23)	0.0248	0.3
A7	A(1,2,3)	-0.0611	0.9
A10	A(3,2,24)	0.0321	0.5
A11	A(3,2,25)	0.0397	0.6
A14	A(2,3,11)	0.126	1.8

!	A15	A(4,3,11)	-0.1347	1.9	!
!	A16	A(3,4,5)	0.0403	0.6	!
!	A18	A(5,4,10)	-0.0519	0.7	!
!	A19	A(4,5,6)	-0.0459	0.6	!
!	A20	A(4,5,7)	0.1187	1.7	!
!	A21	A(6,5,7)	-0.0731	1.0	!
!	A23	A(5,7,8)	-0.0355	0.5	!
!	A24	A(5,7,26)	-0.0255	0.4	!
!	A25	A(8,7,26)	0.0609	0.9	!
!	A26	A(7,8,9)	-0.0822	1.2	!
!	A27	A(7,8,12)	0.0255	0.4	!
!	A28	A(9,8,12)	0.0566	0.8	!
!	A29	A(8,9,10)	0.1155	1.6	!
!	A30	A(8,9,27)	-0.054	0.8	!
!	A31	A(10,9,27)	-0.0615	0.9	!
!	A32	A(4,10,9)	-0.0649	0.9	!
!	A33	A(4,10,14)	0.0382	0.5	!
!	A34	A(9,10,14)	0.0264	0.4	!
!	A35	A(8,12,13)	-0.0446	0.6	!
!	A43	A(1,15,16)	0.0443	0.6	!
!	A45	A(16,15,20)	-0.0373	0.5	!
!	A46	A(15,16,17)	0.1585	2.2	!
!	A47	A(15,16,32)	-0.0861	1.2	!
!	A48	A(17,16,32)	-0.0727	1.0	!
!	A49	A(16,17,18)	-0.137	1.9	!
!	A50	A(16,17,22)	0.0282	0.4	!
!	A51	A(18,17,22)	0.1083	1.5	!
!	A52	A(17,18,19)	-0.0321	0.5	!
!	A53	A(17,18,34)	0.0893	1.3	!
!	A54	A(19,18,34)	-0.0573	0.8	!
!	A55	A(18,19,20)	0.1739	2.4	!
!	A56	A(18,19,21)	-0.1237	1.7	!
!	A57	A(20,19,21)	-0.0501	0.7	!
!	A58	A(15,20,19)	-0.1251	1.8	!
!	A59	A(15,20,33)	0.0938	1.3	!
!	A60	A(19,20,33)	0.0303	0.4	!
!	D5	D(15,1,2,24)	0.0251	0.4	!
!	D6	D(15,1,2,25)	0.0234	0.3	!
!	D7	D(23,1,2,3)	0.0309	0.4	!
!	D8	D(23,1,2,24)	0.0341	0.5	!
!	D9	D(23,1,2,25)	0.0324	0.5	!
!	D10	D(2,1,6,5)	0.0509	0.7	!
!	D12	D(23,1,6,5)	0.0371	0.5	!
!	D13	D(2,1,15,16)	0.0921	1.3	!
!	D14	D(2,1,15,20)	-0.0712	1.0	!
!	D15	D(6,1,15,16)	0.093	1.3	!
!	D16	D(6,1,15,20)	-0.0702	1.0	!
!	D17	D(23,1,15,16)	0.0683	1.0	!
!	D18	D(23,1,15,20)	-0.0949	1.3	!
!	D19	D(1,2,3,4)	-0.0464	0.7	!

D21	D(24,2,3,4)	-0.0328	0.5
D22	D(24,2,3,11)	0.0279	0.4
D23	D(25,2,3,4)	-0.0771	1.1
D25	D(2,3,4,5)	0.0367	0.5
D27	D(11,3,4,5)	-0.0326	0.5
D28	D(11,3,4,10)	-0.0708	1.0
D29	D(3,4,5,6)	-0.0252	0.4
D30	D(3,4,5,7)	-0.0506	0.7
D33	D(3,4,10,9)	0.0461	0.6
D58	D(9,10,14,28)	-0.0368	0.5
D62	D(1,15,16,17)	-0.0256	0.4
D63	D(1,15,16,32)	-0.0754	1.1
D64	D(20,15,16,17)	0.1364	1.9
D65	D(20,15,16,32)	0.0866	1.2
D66	D(1,15,20,19)	0.0288	0.4
D67	D(1,15,20,33)	0.084	1.2
D68	D(16,15,20,19)	-0.1348	1.9
D69	D(16,15,20,33)	-0.0796	1.1
D70	D(15,16,17,18)	-0.0261	0.4
D71	D(15,16,17,22)	-0.1042	1.5
D72	D(32,16,17,18)	0.0236	0.3
D73	D(32,16,17,22)	-0.0545	0.8
D74	D(16,17,18,19)	-0.0836	1.2
D75	D(16,17,18,34)	-0.0992	1.4
D77	D(22,17,18,34)	-0.0252	0.4
D78	D(16,17,22,35)	0.0702	1.0
D80	D(17,18,19,20)	0.0852	1.2
D82	D(34,18,19,20)	0.1003	1.4
D84	D(18,19,20,15)	0.0231	0.3
D85	D(18,19,20,33)	-0.0302	0.4
D86	D(21,19,20,15)	0.1039	1.5
D87	D(21,19,20,33)	0.0506	0.7
D88	D(18,19,21,36)	0.0427	0.6
D89	D(20,19,21,36)	-0.0391	0.5

! Normal Mode 28 !

Name	Definition	Value	Relative Weight (%)
R1	R(1,2)	-0.0444	0.6
R2	R(1,6)	-0.0272	0.4
R5	R(2,3)	-0.0347	0.5
R8	R(3,4)	-0.0379	0.5
R16	R(8,9)	0.0289	0.4
R17	R(8,12)	0.0242	0.3
R20	R(10,14)	0.0384	0.5
R21	R(12,13)	0.0346	0.5
R26	R(15,16)	0.0284	0.4
R30	R(17,18)	0.0241	0.3

```
! R31   R(17,22)                0.0481                  0.7                    !
! R32   R(18,19)                0.0246                  0.3                    !
! A1    A(2,1,6)               -0.0634                  0.9                    !
! A2    A(2,1,15)               0.0426                  0.6                    !
! A3    A(2,1,23)               0.0311                  0.4                    !
! A6    A(15,1,23)             -0.0284                  0.4                    !
! A7    A(1,2,3)                0.0659                  0.9                    !
! A8    A(1,2,24)              -0.0466                  0.6                    !
! A9    A(1,2,25)               0.0498                  0.7                    !
! A10   A(3,2,24)              -0.083                   1.1                    !
! A14   A(2,3,11)              -0.0423                  0.6                    !
! A15   A(4,3,11)               0.0344                  0.5                    !
! A16   A(3,4,5)               -0.0466                  0.6                    !
! A18   A(5,4,10)               0.0615                  0.8                    !
! A21   A(6,5,7)               -0.0334                  0.5                    !
! A22   A(1,6,5)                0.0534                  0.7                    !
! A23   A(5,7,8)               -0.0611                  0.8                    !
! A25   A(8,7,26)               0.0567                  0.8                    !
! A27   A(7,8,12)               0.0646                  0.9                    !
! A28   A(9,8,12)              -0.0726                  1.0                    !
! A29   A(8,9,10)               0.0684                  0.9                    !
! A30   A(8,9,27)              -0.0684                  0.9                    !
! A32   A(4,10,9)              -0.0975                  1.3                    !
! A34   A(9,10,14)              0.097                   1.3                    !
! A35   A(8,12,13)              0.0891                  1.2                    !
! A37   A(12,13,30)            -0.0248                  0.3                    !
! A43   A(1,15,16)             -0.109                   1.5                    !
! A44   A(1,15,20)              0.036                   0.5                    !
! A45   A(16,15,20)             0.0783                  1.1                    !
! A47   A(15,16,32)            -0.0468                  0.6                    !
! A48   A(17,16,32)             0.0445                  0.6                    !
! A49   A(16,17,18)            -0.093                   1.3                    !
! A50   A(16,17,22)             0.1322                  1.8                    !
! A51   A(18,17,22)            -0.0389                  0.5                    !
! A52   A(17,18,19)             0.0849                  1.2                    !
! A53   A(17,18,34)            -0.0476                  0.7                    !
! A54   A(19,18,34)            -0.0372                  0.5                    !
! A56   A(18,19,21)             0.0861                  1.2                    !
! A57   A(20,19,21)            -0.079                   1.1                    !
! A58   A(15,20,19)            -0.0659                  0.9                    !
! A59   A(15,20,33)             0.0392                  0.5                    !
! A60   A(19,20,33)             0.0274                  0.4                    !
! A61   A(19,21,36)             0.0279                  0.4                    !
! A62   A(17,22,35)             0.0261                  0.4                    !
! D1    D(6,1,2,3)              0.0236                  0.3                    !
! D2    D(6,1,2,24)            -0.0688                  0.9                    !
! D3    D(6,1,2,25)            -0.0732                  1.0                    !
! D4    D(15,1,2,3)             0.0374                  0.5                    !
! D5    D(15,1,2,24)           -0.0549                  0.8                    !
! D6    D(15,1,2,25)           -0.0593                  0.8                    !
```

!	D8	D(23,1,2,24)	-0.0698	1.0	!
!	D9	D(23,1,2,25)	-0.0741	1.0	!
!	D10	D(2,1,6,5)	-0.0631	0.9	!
!	D11	D(15,1,6,5)	-0.0494	0.7	!
!	D12	D(23,1,6,5)	-0.0739	1.0	!
!	D13	D(2,1,15,16)	-0.0899	1.2	!
!	D14	D(2,1,15,20)	0.0338	0.5	!
!	D15	D(6,1,15,16)	-0.0389	0.5	!
!	D16	D(6,1,15,20)	0.0848	1.2	!
!	D17	D(23,1,15,16)	-0.0418	0.6	!
!	D18	D(23,1,15,20)	0.0819	1.1	!
!	D19	D(1,2,3,4)	0.0825	1.1	!
!	D20	D(1,2,3,11)	-0.0642	0.9	!
!	D21	D(24,2,3,4)	0.1536	2.1	!
!	D23	D(25,2,3,4)	0.1968	2.7	!
!	D24	D(25,2,3,11)	0.0501	0.7	!
!	D25	D(2,3,4,5)	-0.099	1.4	!
!	D27	D(11,3,4,5)	0.0577	0.8	!
!	D28	D(11,3,4,10)	0.1487	2.0	!
!	D29	D(3,4,5,6)	0.0807	1.1	!
!	D30	D(3,4,5,7)	0.0761	1.0	!
!	D33	D(3,4,10,9)	-0.1111	1.5	!
!	D34	D(3,4,10,14)	-0.025	0.3	!
!	D35	D(5,4,10,9)	-0.027	0.4	!
!	D36	D(5,4,10,14)	0.0591	0.8	!
!	D47	D(7,8,9,10)	-0.0383	0.5	!
!	D48	D(7,8,9,27)	-0.0536	0.7	!
!	D49	D(12,8,9,10)	-0.0527	0.7	!
!	D50	D(12,8,9,27)	-0.068	0.9	!
!	D53	D(8,9,10,4)	0.0488	0.7	!
!	D54	D(8,9,10,14)	-0.0381	0.5	!
!	D55	D(27,9,10,4)	0.0638	0.9	!
!	D56	D(27,9,10,14)	-0.0231	0.3	!
!	D57	D(4,10,14,28)	0.0255	0.3	!
!	D58	D(9,10,14,28)	0.1089	1.5	!
!	D61	D(8,12,13,31)	-0.0287	0.4	!
!	D62	D(1,15,16,17)	0.0307	0.4	!
!	D63	D(1,15,16,32)	0.0499	0.7	!
!	D64	D(20,15,16,17)	-0.0913	1.3	!
!	D65	D(20,15,16,32)	-0.0721	1.0	!
!	D66	D(1,15,20,19)	-0.0329	0.5	!
!	D67	D(1,15,20,33)	-0.0679	0.9	!
!	D68	D(16,15,20,19)	0.0875	1.2	!
!	D69	D(16,15,20,33)	0.0525	0.7	!
!	D71	D(15,16,17,22)	0.0626	0.9	!
!	D73	D(32,16,17,22)	0.0439	0.6	!
!	D74	D(16,17,18,19)	0.0476	0.7	!
!	D75	D(16,17,18,34)	0.0567	0.8	!
!	D78	D(16,17,22,35)	-0.04	0.5	!
!	D80	D(17,18,19,20)	-0.0513	0.7	!

D82	D(34,18,19,20)	-0.0605	0.8
D86	D(21,19,20,15)	-0.07	1.0
D87	D(21,19,20,33)	-0.0355	0.5
D88	D(18,19,21,36)	-0.0291	0.4
D89	D(20,19,21,36)	0.0252	0.3

! Normal Mode 29 !

Name	Definition	Value	Relative Weight (%)
R1	R(1,2)	-0.0388	0.5
R5	R(2,3)	-0.0968	1.4
R10	R(4,5)	0.0674	0.9
R20	R(10,14)	0.0367	0.5
R26	R(15,16)	-0.0256	0.4
R27	R(15,20)	-0.0269	0.4
R30	R(17,18)	-0.0566	0.8
R31	R(17,22)	-0.0626	0.9
R32	R(18,19)	-0.0544	0.8
R35	R(19,21)	-0.0679	0.9
A1	A(2,1,6)	0.0471	0.7
A3	A(2,1,23)	-0.026	0.4
A4	A(6,1,15)	-0.0228	0.3
A8	A(1,2,24)	0.0592	0.8
A9	A(1,2,25)	-0.0407	0.6
A10	A(3,2,24)	0.0309	0.4
A11	A(3,2,25)	-0.0609	0.8
A14	A(2,3,11)	-0.1224	1.7
A15	A(4,3,11)	0.108	1.5
A17	A(3,4,10)	-0.0582	0.8
A18	A(5,4,10)	0.0645	0.9
A20	A(4,5,7)	0.034	0.5
A21	A(6,5,7)	-0.025	0.3
A22	A(1,6,5)	-0.0238	0.3
A23	A(5,7,8)	-0.1103	1.5
A24	A(5,7,26)	0.0509	0.7
A25	A(8,7,26)	0.0594	0.8
A26	A(7,8,9)	0.0627	0.9
A28	A(9,8,12)	-0.0696	1.0
A29	A(8,9,10)	0.0643	0.9
A30	A(8,9,27)	-0.0754	1.1
A32	A(4,10,9)	-0.1152	1.6
A33	A(4,10,14)	0.0555	0.8
A34	A(9,10,14)	0.0594	0.8
A35	A(8,12,13)	0.1193	1.7
A37	A(12,13,30)	-0.0309	0.4
A43	A(1,15,16)	0.0845	1.2
A44	A(1,15,20)	0.0399	0.6
A45	A(16,15,20)	-0.1185	1.7

```
! A46   A(15,16,17)             0.0469                  0.7                   !
! A48   A(17,16,32)            -0.0622                  0.9                   !
! A49   A(16,17,18)             0.0926                  1.3                   !
! A50   A(16,17,22)            -0.0606                  0.8                   !
! A51   A(18,17,22)            -0.0313                  0.4                   !
! A52   A(17,18,19)            -0.162                   2.3                   !
! A53   A(17,18,34)             0.0866                  1.2                   !
! A54   A(19,18,34)             0.0755                  1.1                   !
! A55   A(18,19,20)             0.1112                  1.6                   !
! A56   A(18,19,21)            -0.0715                  1.0                   !
! A57   A(20,19,21)            -0.0398                  0.6                   !
! A58   A(15,20,19)             0.0291                  0.4                   !
! A59   A(15,20,33)             0.0291                  0.4                   !
! A60   A(19,20,33)            -0.0575                  0.8                   !
! D2    D(6,1,2,24)             0.1023                  1.4                   !
! D3    D(6,1,2,25)             0.1126                  1.6                   !
! D5    D(15,1,2,24)            0.0906                  1.3                   !
! D6    D(15,1,2,25)            0.101                   1.4                   !
! D8    D(23,1,2,24)            0.1019                  1.4                   !
! D9    D(23,1,2,25)            0.1123                  1.6                   !
! D10   D(2,1,6,5)             -0.0393                  0.5                   !
! D12   D(23,1,6,5)            -0.0281                  0.4                   !
! D13   D(2,1,15,16)           -0.048                   0.7                   !
! D14   D(2,1,15,20)            0.0835                  1.2                   !
! D15   D(6,1,15,16)           -0.0988                  1.4                   !
! D16   D(6,1,15,20)            0.0327                  0.5                   !
! D17   D(23,1,15,16)          -0.074                   1.0                   !
! D18   D(23,1,15,20)           0.0575                  0.8                   !
! D19   D(1,2,3,4)             -0.0316                  0.4                   !
! D21   D(24,2,3,4)            -0.1338                  1.9                   !
! D22   D(24,2,3,11)           -0.0937                  1.3                   !
! D23   D(25,2,3,4)            -0.1152                  1.6                   !
! D24   D(25,2,3,11)           -0.0751                  1.0                   !
! D25   D(2,3,4,5)              0.0227                  0.3                   !
! D29   D(3,4,5,6)             -0.0243                  0.3                   !
! D37   D(4,5,6,1)              0.0456                  0.6                   !
! D38   D(7,5,6,1)              0.029                   0.4                   !
! D62   D(1,15,16,17)           0.0333                  0.5                   !
! D63   D(1,15,16,32)           0.0737                  1.0                   !
! D64   D(20,15,16,17)         -0.1023                  1.4                   !
! D65   D(20,15,16,32)         -0.0619                  0.9                   !
! D66   D(1,15,20,19)          -0.0305                  0.4                   !
! D67   D(1,15,20,33)          -0.0704                  1.0                   !
! D68   D(16,15,20,19)          0.109                   1.5                   !
! D69   D(16,15,20,33)          0.0692                  1.0                   !
! D71   D(15,16,17,22)          0.0984                  1.4                   !
! D72   D(32,16,17,18)         -0.0375                  0.5                   !
! D73   D(32,16,17,22)          0.0578                  0.8                   !
! D74   D(16,17,18,19)          0.0881                  1.2                   !
! D75   D(16,17,18,34)          0.11                    1.5                   !
```

Name	Definition	Value	Relative Weight (%)
D78	D(16,17,22,35)	-0.0732	1.0
D80	D(17,18,19,20)	-0.0817	1.1
D82	D(34,18,19,20)	-0.1042	1.5
D83	D(34,18,19,21)	-0.0318	0.4
D85	D(18,19,20,33)	0.0244	0.3
D86	D(21,19,20,15)	-0.0848	1.2
D87	D(21,19,20,33)	-0.0446	0.6
D88	D(18,19,21,36)	-0.0302	0.4
D89	D(20,19,21,36)	0.0394	0.5

! Normal Mode 30 !

Name	Definition	Value	Relative Weight (%)
R1	R(1,2)	0.0331	0.5
R2	R(1,6)	0.0496	0.7
R3	R(1,15)	0.0416	0.6
R8	R(3,4)	0.0275	0.4
R10	R(4,5)	0.0854	1.2
R11	R(4,10)	0.0286	0.4
R12	R(5,6)	0.0394	0.5
R16	R(8,9)	0.0537	0.7
R17	R(8,12)	0.0338	0.5
R18	R(9,10)	0.0307	0.4
R20	R(10,14)	0.073	1.0
R27	R(15,20)	0.0441	0.6
R30	R(17,18)	0.0452	0.6
R31	R(17,22)	0.0392	0.5
R32	R(18,19)	0.0455	0.6
R35	R(19,21)	0.073	1.0
A1	A(2,1,6)	0.079	1.1
A3	A(2,1,23)	-0.0646	0.9
A4	A(6,1,15)	0.0353	0.5
A5	A(6,1,23)	-0.0467	0.6
A8	A(1,2,24)	0.0863	1.2
A9	A(1,2,25)	-0.0891	1.2
A10	A(3,2,24)	0.0614	0.8
A11	A(3,2,25)	-0.0537	0.7
A15	A(4,3,11)	0.0316	0.4
A17	A(3,4,10)	-0.0572	0.8
A18	A(5,4,10)	0.0363	0.5
A20	A(4,5,7)	0.0295	0.4
A21	A(6,5,7)	-0.0485	0.7
A22	A(1,6,5)	-0.0634	0.9
A23	A(5,7,8)	-0.043	0.6
A25	A(8,7,26)	0.0292	0.4
A29	A(8,9,10)	0.0865	1.2
A30	A(8,9,27)	-0.0619	0.9
A31	A(10,9,27)	-0.0246	0.3

```
! A32   A(4,10,9)            -0.107                   1.5                   !
! A33   A(4,10,14)            0.047                   0.6                   !
! A34   A(9,10,14)            0.0592                  0.8                   !
! A35   A(8,12,13)           -0.0227                  0.3                   !
! A43   A(1,15,16)            0.0887                  1.2                   !
! A44   A(1,15,20)           -0.1316                  1.8                   !
! A45   A(16,15,20)           0.0447                  0.6                   !
! A46   A(15,16,17)          -0.0393                  0.5                   !
! A47   A(15,16,32)           0.0292                  0.4                   !
! A49   A(16,17,18)          -0.0314                  0.4                   !
! A50   A(16,17,22)          -0.091                   1.3                   !
! A51   A(18,17,22)           0.1227                  1.7                   !
! A52   A(17,18,19)           0.1066                  1.5                   !
! A53   A(17,18,34)          -0.0362                  0.5                   !
! A54   A(19,18,34)          -0.0703                  1.0                   !
! A55   A(18,19,20)          -0.117                   1.6                   !
! A56   A(18,19,21)          -0.0445                  0.6                   !
! A57   A(20,19,21)           0.1615                  2.2                   !
! A58   A(15,20,19)           0.0363                  0.5                   !
! A59   A(15,20,33)          -0.0842                  1.2                   !
! A60   A(19,20,33)           0.0481                  0.7                   !
! A61   A(19,21,36)          -0.0461                  0.6                   !
! A62   A(17,22,35)          -0.0431                  0.6                   !
! D2    D(6,1,2,24)           0.1594                  2.2                   !
! D3    D(6,1,2,25)           0.1477                  2.0                   !
! D4    D(15,1,2,3)          -0.0814                  1.1                   !
! D5    D(15,1,2,24)          0.056                   0.8                   !
! D6    D(15,1,2,25)          0.0442                  0.6                   !
! D7    D(23,1,2,3)          -0.026                   0.4                   !
! D8    D(23,1,2,24)          0.1113                  1.5                   !
! D9    D(23,1,2,25)          0.0996                  1.4                   !
! D10   D(2,1,6,5)           -0.0562                  0.8                   !
! D13   D(2,1,15,16)          0.0419                  0.6                   !
! D14   D(2,1,15,20)          0.0831                  1.1                   !
! D15   D(6,1,15,16)         -0.0855                  1.2                   !
! D16   D(6,1,15,20)         -0.0443                  0.6                   !
! D17   D(23,1,15,16)        -0.0449                  0.6                   !
! D19   D(1,2,3,4)           -0.0731                  1.0                   !
! D20   D(1,2,3,11)           0.0291                  0.4                   !
! D21   D(24,2,3,4)          -0.2244                  3.1                   !
! D22   D(24,2,3,11)         -0.1222                  1.7                   !
! D23   D(25,2,3,4)          -0.217                   3.0                   !
! D24   D(25,2,3,11)         -0.1149                  1.6                   !
! D25   D(2,3,4,5)            0.0674                  0.9                   !
! D27   D(11,3,4,5)          -0.0396                  0.5                   !
! D28   D(11,3,4,10)         -0.086                   1.2                   !
! D29   D(3,4,5,6)           -0.0684                  0.9                   !
! D30   D(3,4,5,7)           -0.0416                  0.6                   !
! D33   D(3,4,10,9)           0.0734                  1.0                   !
! D35   D(5,4,10,9)           0.0278                  0.4                   !
```

D36	D(5,4,10,14)	-0.0496	0.7
D37	D(4,5,6,1)	0.083	1.1
D38	D(7,5,6,1)	0.0574	0.8
D40	D(4,5,7,26)	-0.0227	0.3
D47	D(7,8,9,10)	0.0359	0.5
D48	D(7,8,9,27)	0.0511	0.7
D49	D(12,8,9,10)	0.0366	0.5
D50	D(12,8,9,27)	0.0517	0.7
D53	D(8,9,10,4)	-0.0494	0.7
D54	D(8,9,10,14)	0.0294	0.4
D55	D(27,9,10,4)	-0.0642	0.9
D58	D(9,10,14,28)	-0.071	1.0
D63	D(1,15,16,32)	0.0273	0.4
D64	D(20,15,16,17)	-0.0243	0.3
D67	D(1,15,20,33)	-0.024	0.3
D68	D(16,15,20,19)	0.0298	0.4
D71	D(15,16,17,22)	0.0314	0.4
D72	D(32,16,17,18)	-0.0247	0.3
D74	D(16,17,18,19)	0.0353	0.5
D75	D(16,17,18,34)	0.0417	0.6
D78	D(16,17,22,35)	-0.0282	0.4
D80	D(17,18,19,20)	-0.0292	0.4
D82	D(34,18,19,20)	-0.036	0.5

! Normal Mode 31 !

Name	Definition	Value	Relative Weight (%)
R2	R(1,6)	-0.0571	0.5
R3	R(1,15)	0.0798	0.7
R11	R(4,10)	-0.0639	0.6
R12	R(5,6)	-0.0751	0.7
R16	R(8,9)	-0.0636	0.6
R17	R(8,12)	-0.0895	0.8
R18	R(9,10)	-0.0674	0.6
R20	R(10,14)	-0.0686	0.6
R26	R(15,16)	0.0343	0.3
R27	R(15,20)	0.0387	0.4
R28	R(16,17)	0.0357	0.3
R31	R(17,22)	0.0613	0.6
R32	R(18,19)	0.0388	0.4
R35	R(19,21)	0.0534	0.5
A10	A(3,2,24)	0.0907	0.8
A11	A(3,2,25)	-0.0916	0.8
A14	A(2,3,11)	-0.0724	0.7
A15	A(4,3,11)	0.075	0.7
A20	A(4,5,7)	0.0582	0.5
A21	A(6,5,7)	-0.0353	0.3
A23	A(5,7,8)	-0.1025	0.9

```
! A24   A(5,7,26)              0.036                   0.3                    !
! A25   A(8,7,26)              0.066                   0.6                    !
! A26   A(7,8,9)               0.0741                  0.7                    !
! A27   A(7,8,12)              0.035                   0.3                    !
! A28   A(9,8,12)             -0.1099                  1.0                    !
! A31   A(10,9,27)             0.0429                  0.4                    !
! A33   A(4,10,14)            -0.0392                  0.4                    !
! A35   A(8,12,13)             0.172                   1.6                    !
! A37   A(12,13,30)           -0.0544                  0.5                    !
! A46   A(15,16,17)            0.0462                  0.4                    !
! A49   A(16,17,18)           -0.0501                  0.5                    !
! A50   A(16,17,22)            0.0377                  0.3                    !
! A52   A(17,18,19)            0.041                   0.4                    !
! D2    D(6,1,2,24)            0.1649                  1.5                    !
! D3    D(6,1,2,25)            0.172                   1.6                    !
! D4    D(15,1,2,3)            0.0625                  0.6                    !
! D5    D(15,1,2,24)           0.1942                  1.8                    !
! D6    D(15,1,2,25)           0.2013                  1.8                    !
! D7    D(23,1,2,3)            0.0353                  0.3                    !
! D8    D(23,1,2,24)           0.167                   1.5                    !
! D9    D(23,1,2,25)           0.1741                  1.6                    !
! D10   D(2,1,6,5)             0.0539                  0.5                    !
! D11   D(15,1,6,5)            0.0574                  0.5                    !
! D12   D(23,1,6,5)            0.0688                  0.6                    !
! D13   D(2,1,15,16)           0.0912                  0.8                    !
! D14   D(2,1,15,20)          -0.1357                  1.2                    !
! D15   D(6,1,15,16)           0.1191                  1.1                    !
! D16   D(6,1,15,20)          -0.1077                  1.0                    !
! D17   D(23,1,15,16)          0.1052                  1.0                    !
! D18   D(23,1,15,20)         -0.1216                  1.1                    !
! D19   D(1,2,3,4)            -0.1028                  0.9                    !
! D20   D(1,2,3,11)            0.1421                  1.3                    !
! D21   D(24,2,3,4)           -0.1993                  1.8                    !
! D22   D(24,2,3,11)           0.0456                  0.4                    !
! D23   D(25,2,3,4)           -0.203                   1.9                    !
! D24   D(25,2,3,11)           0.042                   0.4                    !
! D25   D(2,3,4,5)             0.124                   1.1                    !
! D26   D(2,3,4,10)            0.0794                  0.7                    !
! D27   D(11,3,4,5)           -0.1322                  1.2                    !
! D28   D(11,3,4,10)          -0.1767                  1.6                    !
! D29   D(3,4,5,6)            -0.0713                  0.7                    !
! D30   D(3,4,5,7)            -0.0957                  0.9                    !
! D32   D(10,4,5,7)           -0.0554                  0.5                    !
! D33   D(3,4,10,9)            0.1422                  1.3                    !
! D35   D(5,4,10,9)            0.1001                  0.9                    !
! D36   D(5,4,10,14)          -0.064                   0.6                    !
! D40   D(4,5,7,26)           -0.0382                  0.3                    !
! D42   D(6,5,7,26)           -0.0609                  0.6                    !
! D44   D(5,7,8,12)           -0.0453                  0.4                    !
! D45   D(26,7,8,9)            0.0554                  0.5                    !
```

D48	D(7,8,9,27)	0.0463	0.4
D49	D(12,8,9,10)	0.0936	0.9
D50	D(12,8,9,27)	0.115	1.1
D52	D(9,8,12,13)	-0.0555	0.5
D53	D(8,9,10,4)	-0.0881	0.8
D54	D(8,9,10,14)	0.0793	0.7
D55	D(27,9,10,4)	-0.1091	1.0
D56	D(27,9,10,14)	0.0583	0.5
D58	D(9,10,14,28)	-0.1362	1.2
D59	D(8,12,13,29)	0.0654	0.6
D60	D(8,12,13,30)	0.0438	0.4
D62	D(1,15,16,17)	-0.0974	0.9
D63	D(1,15,16,32)	-0.1152	1.1
D64	D(20,15,16,17)	0.1268	1.2
D65	D(20,15,16,32)	0.1091	1.0
D66	D(1,15,20,19)	0.0941	0.9
D67	D(1,15,20,33)	0.1205	1.1
D68	D(16,15,20,19)	-0.1354	1.2
D69	D(16,15,20,33)	-0.1091	1.0
D71	D(15,16,17,22)	-0.1446	1.3
D72	D(32,16,17,18)	0.0417	0.4
D73	D(32,16,17,22)	-0.1268	1.2
D74	D(16,17,18,19)	-0.1623	1.5
D75	D(16,17,18,34)	-0.205	1.9
D77	D(22,17,18,34)	-0.046	0.4
D78	D(16,17,22,35)	0.1234	1.1
D79	D(18,17,22,35)	-0.0393	0.4
D80	D(17,18,19,20)	0.1543	1.4
D82	D(34,18,19,20)	0.1981	1.8
D83	D(34,18,19,21)	0.0544	0.5
D86	D(21,19,20,15)	0.1302	1.2
D87	D(21,19,20,33)	0.1044	1.0
D88	D(18,19,21,36)	0.0561	0.5
D89	D(20,19,21,36)	-0.0819	0.7

! Normal Mode 32 !

Name	Definition	Value	Relative Weight (%)
R5	R(2,3)	0.0296	0.3
A2	A(2,1,15)	0.0336	0.4
A19	A(4,5,6)	0.0347	0.4
A20	A(4,5,7)	-0.0316	0.4
D10	D(2,1,6,5)	-0.0344	0.4
D14	D(2,1,15,20)	-0.0489	0.6
D16	D(6,1,15,20)	-0.0621	0.7
D18	D(23,1,15,20)	-0.0542	0.6
D21	D(24,2,3,4)	-0.0328	0.4
D29	D(3,4,5,6)	-0.0326	0.4

D31	D(10,4,5,6)	-0.0305	0.3
D34	D(3,4,10,14)	-0.028	0.3
D36	D(5,4,10,14)	-0.031	0.4
D37	D(4,5,6,1)	0.0391	0.4
D40	D(4,5,7,26)	-0.034	0.4
D48	D(7,8,9,27)	0.0443	0.5
D50	D(12,8,9,27)	0.0386	0.4
D53	D(8,9,10,4)	-0.0353	0.4
D55	D(27,9,10,4)	-0.0579	0.7
D62	D(1,15,16,17)	-0.227	2.6
D63	D(1,15,16,32)	-0.3247	3.7
D64	D(20,15,16,17)	-0.1757	2.0
D65	D(20,15,16,32)	-0.2734	3.1
D66	D(1,15,20,19)	-0.1092	1.2
D67	D(1,15,20,33)	-0.1852	2.1
D68	D(16,15,20,19)	-0.1613	1.8
D69	D(16,15,20,33)	-0.2373	2.7
D70	D(15,16,17,18)	0.4042	4.6
D71	D(15,16,17,22)	-0.2242	2.5
D72	D(32,16,17,18)	0.5017	5.7
D73	D(32,16,17,22)	-0.1267	1.4
D74	D(16,17,18,19)	-0.2892	3.3
D75	D(16,17,18,34)	-0.3452	3.9
D76	D(22,17,18,19)	0.3043	3.5
D77	D(22,17,18,34)	0.2483	2.8
D78	D(16,17,22,35)	0.3312	3.8
D79	D(18,17,22,35)	-0.2745	3.1
D80	D(17,18,19,20)	-0.055	0.6
D81	D(17,18,19,21)	0.3522	4.0
D83	D(34,18,19,21)	0.4095	4.7
D84	D(18,19,20,15)	0.2787	3.2
D85	D(18,19,20,33)	0.3534	4.0
D86	D(21,19,20,15)	-0.1089	1.2
D87	D(21,19,20,33)	-0.0342	0.4
D88	D(18,19,21,36)	-0.2998	3.4
D89	D(20,19,21,36)	0.0916	1.0

! Normal Mode 33 !

Name	Definition	Value	Relative Weight (%)
R3	R(1,15)	0.0378	0.3
R5	R(2,3)	-0.074	0.7
R11	R(4,10)	-0.0472	0.4
R12	R(5,6)	-0.0393	0.4
R17	R(8,12)	-0.0417	0.4
R21	R(12,13)	-0.0448	0.4
A7	A(1,2,3)	-0.0522	0.5
A10	A(3,2,24)	-0.0608	0.5

```
! A11   A(3,2,25)            0.0613              0.5                  !
! A16   A(3,4,5)             0.0649              0.6                  !
! A18   A(5,4,10)           -0.0372              0.3                  !
! A19   A(4,5,6)            -0.1051              0.9                  !
! A20   A(4,5,7)             0.0718              0.6                  !
! A23   A(5,7,8)            -0.0682              0.6                  !
! A24   A(5,7,26)            0.0526              0.5                  !
! A27   A(7,8,12)           -0.0633              0.6                  !
! A28   A(9,8,12)            0.0455              0.4                  !
! A33   A(4,10,14)           0.0633              0.6                  !
! A34   A(9,10,14)          -0.0547              0.5                  !
! A43   A(1,15,16)           0.0419              0.4                  !
! D2    D(6,1,2,24)         -0.1114              1.0                  !
! D3    D(6,1,2,25)         -0.0743              0.7                  !
! D4    D(15,1,2,3)         -0.0578              0.5                  !
! D5    D(15,1,2,24)        -0.1568              1.4                  !
! D6    D(15,1,2,25)        -0.1196              1.1                  !
! D8    D(23,1,2,24)        -0.1278              1.1                  !
! D9    D(23,1,2,25)        -0.0906              0.8                  !
! D10   D(2,1,6,5)           0.1069              1.0                  !
! D11   D(15,1,6,5)          0.1292              1.2                  !
! D12   D(23,1,6,5)          0.1268              1.1                  !
! D19   D(1,2,3,4)           0.0642              0.6                  !
! D20   D(1,2,3,11)         -0.1228              1.1                  !
! D21   D(24,2,3,4)          0.118               1.1                  !
! D22   D(24,2,3,11)        -0.069               0.6                  !
! D23   D(25,2,3,4)          0.1074              1.0                  !
! D24   D(25,2,3,11)        -0.0796              0.7                  !
! D25   D(2,3,4,5)          -0.1782              1.6                  !
! D26   D(2,3,4,10)         -0.1675              1.5                  !
! D29   D(3,4,5,6)           0.2437              2.2                  !
! D30   D(3,4,5,7)          -0.04                0.4                  !
! D31   D(10,4,5,6)          0.2337              2.1                  !
! D32   D(10,4,5,7)         -0.05                0.4                  !
! D33   D(3,4,10,9)         -0.2007              1.8                  !
! D34   D(3,4,10,14)         0.2246              2.0                  !
! D35   D(5,4,10,9)         -0.1862              1.7                  !
! D36   D(5,4,10,14)         0.2391              2.1                  !
! D37   D(4,5,6,1)          -0.1884              1.7                  !
! D38   D(7,5,6,1)           0.0762              0.7                  !
! D39   D(4,5,7,8)           0.1922              1.7                  !
! D40   D(4,5,7,26)          0.289               2.6                  !
! D41   D(6,5,7,8)          -0.0742              0.7                  !
! D43   D(5,7,8,9)          -0.1017              0.9                  !
! D44   D(5,7,8,12)         -0.0701              0.6                  !
! D45   D(26,7,8,9)         -0.1991              1.8                  !
! D46   D(26,7,8,12)        -0.1674              1.5                  !
! D47   D(7,8,9,10)         -0.1274              1.1                  !
! D48   D(7,8,9,27)         -0.2959              2.6                  !
! D49   D(12,8,9,10)        -0.1609              1.4                  !
```

! D50	D(12,8,9,27)	-0.3294	2.9	!
! D53	D(8,9,10,4)	0.2748	2.5	!
! D54	D(8,9,10,14)	-0.1588	1.4	!
! D55	D(27,9,10,4)	0.4394	3.9	!
! D57	D(4,10,14,28)	-0.1782	1.6	!
! D58	D(9,10,14,28)	0.2412	2.2	!
! D60	D(8,12,13,30)	-0.0402	0.4	!
! D62	D(1,15,16,17)	-0.0426	0.4	!
! D63	D(1,15,16,32)	-0.0606	0.5	!
! D64	D(20,15,16,17)	-0.0555	0.5	!
! D65	D(20,15,16,32)	-0.0735	0.7	!
! D66	D(1,15,20,19)	-0.0437	0.4	!
! D67	D(1,15,20,33)	-0.0723	0.6	!
! D69	D(16,15,20,33)	-0.0576	0.5	!
! D70	D(15,16,17,18)	0.0773	0.7	!
! D72	D(32,16,17,18)	0.0952	0.9	!
! D76	D(22,17,18,19)	0.0809	0.7	!
! D77	D(22,17,18,34)	0.0834	0.7	!
! D78	D(16,17,22,35)	0.0439	0.4	!
! D79	D(18,17,22,35)	-0.0527	0.5	!
! D80	D(17,18,19,20)	-0.0726	0.6	!
! D81	D(17,18,19,21)	0.0847	0.8	!
! D82	D(34,18,19,20)	-0.0752	0.7	!
! D83	D(34,18,19,21)	0.082	0.7	!
! D84	D(18,19,20,15)	0.0939	0.8	!
! D85	D(18,19,20,33)	0.1218	1.1	!
! D86	D(21,19,20,15)	-0.0558	0.5	!
! D88	D(18,19,21,36)	-0.097	0.9	!
! D89	D(20,19,21,36)	0.0542	0.5	!

! Normal Mode 34 !

! Name	Definition	Value	Relative Weight (%)	!
! R1	R(1,2)	0.0524	0.4	!
! R3	R(1,15)	-0.0453	0.4	!
! R5	R(2,3)	0.0786	0.7	!
! R8	R(3,4)	0.044	0.4	!
! R10	R(4,5)	0.0479	0.4	!
! R11	R(4,10)	0.0538	0.5	!
! A2	A(2,1,15)	0.1065	0.9	!
! A3	A(2,1,23)	-0.0628	0.5	!
! A4	A(6,1,15)	-0.0396	0.3	!
! A8	A(1,2,24)	0.0542	0.5	!
! A18	A(5,4,10)	0.05	0.4	!
! A19	A(4,5,6)	0.0593	0.5	!
! A20	A(4,5,7)	-0.0625	0.5	!
! A22	A(1,6,5)	-0.0455	0.4	!
! A26	A(7,8,9)	0.0481	0.4	!

```
! A28   A(9,8,12)           -0.0658          0.6            !
! A29   A(8,9,10)           -0.0383          0.3            !
! A31   A(10,9,27)           0.0414          0.3            !
! A33   A(4,10,14)          -0.0477          0.4            !
! A34   A(9,10,14)           0.0593          0.5            !
! A44   A(1,15,20)          -0.0418          0.4            !
! D2    D(6,1,2,24)          0.0458          0.4            !
! D3    D(6,1,2,25)          0.0616          0.5            !
! D10   D(2,1,6,5)           0.0383          0.3            !
! D11   D(15,1,6,5)          0.1534          1.3            !
! D12   D(23,1,6,5)          0.1091          0.9            !
! D13   D(2,1,15,16)         0.1403          1.2            !
! D14   D(2,1,15,20)        -0.105           0.9            !
! D15   D(6,1,15,16)         0.0808          0.7            !
! D16   D(6,1,15,20)        -0.1645          1.4            !
! D17   D(23,1,15,16)        0.1193          1.0            !
! D18   D(23,1,15,20)       -0.126           1.1            !
! D21   D(24,2,3,4)         -0.073           0.6            !
! D22   D(24,2,3,11)        -0.0921          0.8            !
! D23   D(25,2,3,4)         -0.0546          0.5            !
! D24   D(25,2,3,11)        -0.0737          0.6            !
! D25   D(2,3,4,5)          -0.0861          0.7            !
! D26   D(2,3,4,10)         -0.1068          0.9            !
! D27   D(11,3,4,5)         -0.0646          0.5            !
! D28   D(11,3,4,10)        -0.0853          0.7            !
! D29   D(3,4,5,6)           0.1558          1.3            !
! D30   D(3,4,5,7)          -0.084           0.7            !
! D31   D(10,4,5,6)          0.1777          1.5            !
! D32   D(10,4,5,7)         -0.0621          0.5            !
! D33   D(3,4,10,9)         -0.0785          0.7            !
! D34   D(3,4,10,14)         0.1805          1.5            !
! D35   D(5,4,10,9)         -0.1018          0.9            !
! D36   D(5,4,10,14)         0.1571          1.3            !
! D37   D(4,5,6,1)          -0.1278          1.1            !
! D38   D(7,5,6,1)           0.0967          0.8            !
! D39   D(4,5,7,8)           0.1566          1.3            !
! D40   D(4,5,7,26)          0.2391          2.0            !
! D41   D(6,5,7,8)          -0.0672          0.6            !
! D43   D(5,7,8,9)          -0.088           0.7            !
! D44   D(5,7,8,12)         -0.0741          0.6            !
! D45   D(26,7,8,9)         -0.1709          1.4            !
! D46   D(26,7,8,12)        -0.157           1.3            !
! D47   D(7,8,9,10)         -0.0714          0.6            !
! D48   D(7,8,9,27)         -0.1981          1.7            !
! D49   D(12,8,9,10)        -0.0872          0.7            !
! D50   D(12,8,9,27)        -0.2139          1.8            !
! D53   D(8,9,10,4)          0.1692          1.4            !
! D54   D(8,9,10,14)        -0.0936          0.8            !
! D55   D(27,9,10,4)         0.2929          2.5            !
! D57   D(4,10,14,28)       -0.1403          1.2            !
```

```
! D58   D(9,10,14,28)           0.1143                 1.0                   !
! D62   D(1,15,16,17)          -0.126                  1.1                   !
! D63   D(1,15,16,32)          -0.0774                 0.7                   !
! D64   D(20,15,16,17)          0.1205                 1.0                   !
! D65   D(20,15,16,32)          0.1691                 1.4                   !
! D66   D(1,15,20,19)           0.2159                 1.8                   !
! D67   D(1,15,20,33)           0.2314                 2.0                   !
! D71   D(15,16,17,22)         -0.1192                 1.0                   !
! D72   D(32,16,17,18)         -0.0411                 0.3                   !
! D73   D(32,16,17,22)         -0.1676                 1.4                   !
! D74   D(16,17,18,19)         -0.2182                 1.8                   !
! D75   D(16,17,18,34)         -0.2982                 2.5                   !
! D76   D(22,17,18,19)         -0.0986                 0.8                   !
! D77   D(22,17,18,34)         -0.1786                 1.5                   !
! D78   D(16,17,22,35)          0.1181                 1.0                   !
! D80   D(17,18,19,20)          0.3067                 2.6                   !
! D81   D(17,18,19,21)         -0.082                  0.7                   !
! D82   D(34,18,19,20)          0.3887                 3.3                   !
! D84   D(18,19,20,15)         -0.1817                 1.5                   !
! D85   D(18,19,20,33)         -0.197                  1.7                   !
! D86   D(21,19,20,15)          0.1883                 1.6                   !
! D87   D(21,19,20,33)          0.173                  1.5                   !
! D88   D(18,19,21,36)          0.193                  1.6                   !
! D89   D(20,19,21,36)         -0.1806                 1.5                   !
--------------------------------------------------------------------------------
                          ----------------------------
                          ! Normal Mode    35        !
--------------------------                            --------------------------
! Name  Definition              Value         Relative Weight (%)            !
--------------------------------------------------------------------------------
! R8    R(3,4)                 -0.041                  0.3                   !
! A2    A(2,1,15)              -0.0673                 0.5                   !
! A3    A(2,1,23)               0.0448                 0.4                   !
! A4    A(6,1,15)               0.0563                 0.4                   !
! A7    A(1,2,3)                0.0633                 0.5                   !
! A10   A(3,2,24)               0.0667                 0.5                   !
! A11   A(3,2,25)              -0.0473                 0.4                   !
! A14   A(2,3,11)               0.0485                 0.4                   !
! A19   A(4,5,6)                0.0616                 0.5                   !
! A21   A(6,5,7)               -0.0541                 0.4                   !
! A22   A(1,6,5)               -0.0768                 0.6                   !
! A24   A(5,7,26)              -0.0515                 0.4                   !
! A27   A(7,8,12)               0.0631                 0.5                   !
! A33   A(4,10,14)             -0.0465                 0.4                   !
! D2    D(6,1,2,24)             0.1205                 0.9                   !
! D3    D(6,1,2,25)             0.0635                 0.5                   !
! D5    D(15,1,2,24)            0.1216                 1.0                   !
! D6    D(15,1,2,25)            0.0646                 0.5                   !
! D8    D(23,1,2,24)            0.1359                 1.1                   !
! D9    D(23,1,2,25)            0.0788                 0.6                   !
```

```
! D10   D(2,1,6,5)              0.1385                 1.1                  !
! D11   D(15,1,6,5)             0.0667                 0.5                  !
! D12   D(23,1,6,5)             0.1008                 0.8                  !
! D13   D(2,1,15,16)           -0.0775                 0.6                  !
! D14   D(2,1,15,20)            0.0833                 0.7                  !
! D16   D(6,1,15,20)            0.1373                 1.1                  !
! D17   D(23,1,15,16)          -0.0662                 0.5                  !
! D18   D(23,1,15,20)           0.0947                 0.7                  !
! D19   D(1,2,3,4)             -0.1125                 0.9                  !
! D20   D(1,2,3,11)             0.1488                 1.2                  !
! D21   D(24,2,3,4)            -0.1529                 1.2                  !
! D22   D(24,2,3,11)            0.1083                 0.8                  !
! D23   D(25,2,3,4)            -0.1524                 1.2                  !
! D24   D(25,2,3,11)            0.1088                 0.9                  !
! D26   D(2,3,4,10)             0.0416                 0.3                  !
! D27   D(11,3,4,5)            -0.2452                 1.9                  !
! D28   D(11,3,4,10)           -0.2361                 1.8                  !
! D29   D(3,4,5,6)              0.1808                 1.4                  !
! D30   D(3,4,5,7)             -0.1917                 1.5                  !
! D31   D(10,4,5,6)             0.1704                 1.3                  !
! D32   D(10,4,5,7)            -0.2021                 1.6                  !
! D33   D(3,4,10,9)             0.0641                 0.5                  !
! D34   D(3,4,10,14)            0.0991                 0.8                  !
! D35   D(5,4,10,9)             0.0726                 0.6                  !
! D36   D(5,4,10,14)            0.1076                 0.8                  !
! D37   D(4,5,6,1)             -0.2806                 2.2                  !
! D38   D(7,5,6,1)              0.0671                 0.5                  !
! D39   D(4,5,7,8)              0.0985                 0.8                  !
! D40   D(4,5,7,26)             0.1271                 1.0                  !
! D41   D(6,5,7,8)             -0.249                  1.9                  !
! D42   D(6,5,7,26)            -0.2203                 1.7                  !
! D43   D(5,7,8,9)              0.1415                 1.1                  !
! D44   D(5,7,8,12)            -0.2307                 1.8                  !
! D45   D(26,7,8,9)             0.1134                 0.9                  !
! D46   D(26,7,8,12)           -0.2588                 2.0                  !
! D47   D(7,8,9,10)            -0.2668                 2.1                  !
! D48   D(7,8,9,27)            -0.4457                 3.5                  !
! D49   D(12,8,9,10)            0.1362                 1.1                  !
! D50   D(12,8,9,27)           -0.0427                 0.3                  !
! D51   D(7,8,12,13)            0.0885                 0.7                  !
! D52   D(9,8,12,13)           -0.2974                 2.3                  !
! D53   D(8,9,10,4)             0.1565                 1.2                  !
! D54   D(8,9,10,14)            0.1214                 1.0                  !
! D55   D(27,9,10,4)            0.3312                 2.6                  !
! D56   D(27,9,10,14)           0.2961                 2.3                  !
! D57   D(4,10,14,28)          -0.1166                 0.9                  !
! D58   D(9,10,14,28)          -0.0822                 0.6                  !
! D59   D(8,12,13,29)           0.1552                 1.2                  !
! D60   D(8,12,13,30)           0.1449                 1.1                  !
! D61   D(8,12,13,31)           0.1471                 1.2                  !
```

!	D62	D(1,15,16,17)	0.1155	0.9
!	D63	D(1,15,16,32)	0.0839	0.7
!	D64	D(20,15,16,17)	-0.0438	0.3
!	D65	D(20,15,16,32)	-0.0753	0.6
!	D66	D(1,15,20,19)	-0.1471	1.2
!	D67	D(1,15,20,33)	-0.1465	1.1
!	D70	D(15,16,17,18)	-0.0419	0.3
!	D71	D(15,16,17,22)	0.08	0.6
!	D73	D(32,16,17,22)	0.1115	0.9
!	D74	D(16,17,18,19)	0.1528	1.2
!	D75	D(16,17,18,34)	0.208	1.6
!	D77	D(22,17,18,34)	0.0929	0.7
!	D78	D(16,17,22,35)	-0.0966	0.8
!	D80	D(17,18,19,20)	-0.1816	1.4
!	D82	D(34,18,19,20)	-0.2382	1.9
!	D84	D(18,19,20,15)	0.0979	0.8
!	D85	D(18,19,20,33)	0.0972	0.8
!	D86	D(21,19,20,15)	-0.0987	0.8
!	D87	D(21,19,20,33)	-0.0993	0.8
!	D88	D(18,19,21,36)	-0.0955	0.7
!	D89	D(20,19,21,36)	0.1029	0.8

! Normal Mode 36 !

!	Name	Definition	Value	Relative Weight (%)
!	R2	R(1,6)	0.0327	0.3
!	R8	R(3,4)	0.0563	0.5
!	R10	R(4,5)	0.0513	0.5
!	A4	A(6,1,15)	-0.0599	0.6
!	A8	A(1,2,24)	-0.0387	0.4
!	A9	A(1,2,25)	0.0537	0.5
!	A10	A(3,2,24)	-0.0554	0.5
!	A11	A(3,2,25)	0.048	0.5
!	A16	A(3,4,5)	0.0379	0.4
!	A17	A(3,4,10)	-0.0491	0.5
!	A19	A(4,5,6)	-0.0715	0.7
!	A21	A(6,5,7)	0.0784	0.8
!	A22	A(1,6,5)	0.033	0.3
!	A24	A(5,7,26)	0.0572	0.5
!	A25	A(8,7,26)	-0.0352	0.3
!	A27	A(7,8,12)	-0.078	0.7
!	A28	A(9,8,12)	0.0472	0.5
!	A33	A(4,10,14)	0.0568	0.5
!	A34	A(9,10,14)	-0.0384	0.4
!	A35	A(8,12,13)	-0.0437	0.4
!	A44	A(1,15,20)	0.0356	0.3
!	D1	D(6,1,2,3)	-0.0366	0.4
!	D2	D(6,1,2,24)	-0.1396	1.3

! D3	D(6,1,2,25)	-0.1258	1.2 !
! D5	D(15,1,2,24)	-0.0748	0.7 !
! D6	D(15,1,2,25)	-0.0611	0.6 !
! D8	D(23,1,2,24)	-0.1203	1.2 !
! D9	D(23,1,2,25)	-0.1066	1.0 !
! D10	D(2,1,6,5)	0.0391	0.4 !
! D14	D(2,1,15,20)	-0.041	0.4 !
! D15	D(6,1,15,16)	0.0333	0.3 !
! D17	D(23,1,15,16)	0.0455	0.4 !
! D19	D(1,2,3,4)	0.0498	0.5 !
! D20	D(1,2,3,11)	-0.0372	0.4 !
! D21	D(24,2,3,4)	0.1423	1.4 !
! D22	D(24,2,3,11)	0.0553	0.5 !
! D23	D(25,2,3,4)	0.1412	1.4 !
! D24	D(25,2,3,11)	0.0541	0.5 !
! D25	D(2,3,4,5)	-0.0493	0.5 !
! D26	D(2,3,4,10)	0.0588	0.6 !
! D27	D(11,3,4,5)	0.0438	0.4 !
! D28	D(11,3,4,10)	0.1518	1.5 !
! D29	D(3,4,5,6)	0.0375	0.4 !
! D30	D(3,4,5,7)	0.0848	0.8 !
! D31	D(10,4,5,6)	-0.0602	0.6 !
! D34	D(3,4,10,14)	-0.1706	1.6 !
! D35	D(5,4,10,9)	0.0832	0.8 !
! D36	D(5,4,10,14)	-0.0647	0.6 !
! D38	D(7,5,6,1)	-0.0724	0.7 !
! D39	D(4,5,7,8)	-0.2146	2.1 !
! D40	D(4,5,7,26)	-0.2976	2.9 !
! D41	D(6,5,7,8)	-0.1714	1.6 !
! D42	D(6,5,7,26)	-0.2544	2.4 !
! D43	D(5,7,8,9)	0.3756	3.6 !
! D44	D(5,7,8,12)	-0.0987	0.9 !
! D45	D(26,7,8,9)	0.458	4.4 !
! D47	D(7,8,9,10)	-0.3085	3.0 !
! D48	D(7,8,9,27)	-0.3808	3.7 !
! D49	D(12,8,9,10)	0.2071	2.0 !
! D50	D(12,8,9,27)	0.1348	1.3 !
! D51	D(7,8,12,13)	0.1318	1.3 !
! D52	D(9,8,12,13)	-0.3601	3.5 !
! D53	D(8,9,10,4)	0.0744	0.7 !
! D54	D(8,9,10,14)	0.2243	2.2 !
! D55	D(27,9,10,4)	0.145	1.4 !
! D56	D(27,9,10,14)	0.2949	2.8 !
! D58	D(9,10,14,28)	-0.1262	1.2 !
! D59	D(8,12,13,29)	0.1698	1.6 !
! D60	D(8,12,13,30)	0.1767	1.7 !
! D61	D(8,12,13,31)	0.1863	1.8 !
! D62	D(1,15,16,17)	-0.0548	0.5 !
! D63	D(1,15,16,32)	-0.0344	0.3 !
! D66	D(1,15,20,19)	0.0687	0.7 !

D67	D(1,15,20,33)	0.0661	0.6
D70	D(15,16,17,18)	0.0518	0.5
D73	D(32,16,17,22)	-0.0515	0.5
D74	D(16,17,18,19)	-0.0973	0.9
D75	D(16,17,18,34)	-0.1301	1.2
D77	D(22,17,18,34)	-0.0519	0.5
D78	D(16,17,22,35)	0.0571	0.5
D80	D(17,18,19,20)	0.1093	1.0
D82	D(34,18,19,20)	0.1429	1.4
D84	D(18,19,20,15)	-0.0749	0.7
D85	D(18,19,20,33)	-0.0722	0.7
D86	D(21,19,20,15)	0.0365	0.4
D87	D(21,19,20,33)	0.0393	0.4
D88	D(18,19,21,36)	0.0539	0.5
D89	D(20,19,21,36)	-0.0586	0.6

! Normal Mode 37 !

Name	Definition	Value	Relative Weight (%)
R1	R(1,2)	0.0492	0.5
R8	R(3,4)	0.0963	0.9
R10	R(4,5)	0.0866	0.8
A1	A(2,1,6)	-0.0567	0.5
A2	A(2,1,15)	-0.0649	0.6
A3	A(2,1,23)	0.1187	1.1
A4	A(6,1,15)	-0.1005	0.9
A5	A(6,1,23)	0.0482	0.5
A6	A(15,1,23)	0.0601	0.6
A8	A(1,2,24)	-0.1433	1.3
A9	A(1,2,25)	0.0567	0.5
A10	A(3,2,24)	0.0414	0.4
A13	A(2,3,4)	-0.0572	0.5
A15	A(4,3,11)	0.0368	0.3
A16	A(3,4,5)	0.0515	0.5
A17	A(3,4,10)	-0.0757	0.7
A19	A(4,5,6)	-0.0755	0.7
A21	A(6,5,7)	0.0991	0.9
A24	A(5,7,26)	0.0701	0.7
A25	A(8,7,26)	-0.0478	0.4
A26	A(7,8,9)	0.048	0.4
A27	A(7,8,12)	-0.0848	0.8
A28	A(9,8,12)	0.0392	0.4
A33	A(4,10,14)	0.0482	0.5
A34	A(9,10,14)	-0.0354	0.3
A35	A(8,12,13)	-0.0454	0.4
A43	A(1,15,16)	-0.0741	0.7
A44	A(1,15,20)	0.0921	0.9
A46	A(15,16,17)	0.0347	0.3

!	A50	A(16,17,22)	0.0543	0.5	!
!	A51	A(18,17,22)	-0.0363	0.3	!
!	A56	A(18,19,21)	0.0347	0.3	!
!	A57	A(20,19,21)	-0.048	0.4	!
!	D1	D(6,1,2,3)	-0.0712	0.7	!
!	D2	D(6,1,2,24)	-0.0932	0.9	!
!	D3	D(6,1,2,25)	-0.152	1.4	!
!	D4	D(15,1,2,3)	0.1413	1.3	!
!	D5	D(15,1,2,24)	0.1193	1.1	!
!	D6	D(15,1,2,25)	0.0605	0.6	!
!	D9	D(23,1,2,25)	-0.0583	0.5	!
!	D10	D(2,1,6,5)	0.2353	2.2	!
!	D11	D(15,1,6,5)	0.0534	0.5	!
!	D12	D(23,1,6,5)	0.0973	0.9	!
!	D13	D(2,1,15,16)	-0.1043	1.0	!
!	D14	D(2,1,15,20)	-0.0604	0.6	!
!	D15	D(6,1,15,16)	0.0818	0.8	!
!	D16	D(6,1,15,20)	0.1257	1.2	!
!	D17	D(23,1,15,16)	0.0473	0.4	!
!	D18	D(23,1,15,20)	0.0912	0.9	!
!	D19	D(1,2,3,4)	-0.0803	0.7	!
!	D20	D(1,2,3,11)	0.201	1.9	!
!	D21	D(24,2,3,4)	0.0505	0.5	!
!	D22	D(24,2,3,11)	0.3318	3.1	!
!	D24	D(25,2,3,11)	0.3034	2.8	!
!	D25	D(2,3,4,5)	0.082	0.8	!
!	D26	D(2,3,4,10)	0.1032	1.0	!
!	D27	D(11,3,4,5)	-0.2153	2.0	!
!	D28	D(11,3,4,10)	-0.194	1.8	!
!	D29	D(3,4,5,6)	0.0941	0.9	!
!	D30	D(3,4,5,7)	-0.1741	1.6	!
!	D31	D(10,4,5,6)	0.0788	0.7	!
!	D32	D(10,4,5,7)	-0.1894	1.8	!
!	D33	D(3,4,10,9)	0.1067	1.0	!
!	D34	D(3,4,10,14)	0.0339	0.3	!
!	D35	D(5,4,10,9)	0.1285	1.2	!
!	D36	D(5,4,10,14)	0.0557	0.5	!
!	D37	D(4,5,6,1)	-0.2656	2.5	!
!	D39	D(4,5,7,8)	0.2449	2.3	!
!	D40	D(4,5,7,26)	0.3157	2.9	!
!	D42	D(6,5,7,26)	0.0637	0.6	!
!	D43	D(5,7,8,9)	-0.2348	2.2	!
!	D44	D(5,7,8,12)	-0.0472	0.4	!
!	D45	D(26,7,8,9)	-0.3067	2.9	!
!	D46	D(26,7,8,12)	-0.1192	1.1	!
!	D47	D(7,8,9,10)	0.1778	1.7	!
!	D48	D(7,8,9,27)	0.224	2.1	!
!	D51	D(7,8,12,13)	-0.0652	0.6	!
!	D52	D(9,8,12,13)	0.1293	1.2	!
!	D53	D(8,9,10,4)	-0.127	1.2	!

D54	D(8,9,10,14)	-0.0535	0.5
D55	D(27,9,10,4)	-0.1721	1.6
D56	D(27,9,10,14)	-0.0986	0.9
D57	D(4,10,14,28)	0.0341	0.3
D58	D(9,10,14,28)	-0.0374	0.3
D59	D(8,12,13,29)	-0.0612	0.6
D60	D(8,12,13,30)	-0.051	0.5
D61	D(8,12,13,31)	-0.0438	0.4
D62	D(1,15,16,17)	0.0473	0.4
D66	D(1,15,20,19)	-0.0523	0.5
D67	D(1,15,20,33)	-0.0358	0.3
D73	D(32,16,17,22)	0.0346	0.3
D74	D(16,17,18,19)	0.0385	0.4
D75	D(16,17,18,34)	0.0581	0.5
D80	D(17,18,19,20)	-0.0476	0.4
D82	D(34,18,19,20)	-0.0675	0.6
D87	D(21,19,20,33)	-0.0365	0.3

! Normal Mode 38 !

Name	Definition	Value	Relative Weight (%)
R5	R(2,3)	-0.0823	0.9
R8	R(3,4)	-0.0802	0.9
R11	R(4,10)	-0.0374	0.4
R17	R(8,12)	0.0307	0.4
A2	A(2,1,15)	-0.0409	0.5
A7	A(1,2,3)	0.0357	0.4
A11	A(3,2,25)	-0.0323	0.4
A22	A(1,6,5)	-0.0291	0.3
A23	A(5,7,8)	0.0456	0.5
A24	A(5,7,26)	-0.0352	0.4
A26	A(7,8,9)	-0.0673	0.8
A28	A(9,8,12)	0.0595	0.7
A29	A(8,9,10)	0.0339	0.4
A31	A(10,9,27)	-0.0405	0.5
A33	A(4,10,14)	0.0378	0.4
A34	A(9,10,14)	-0.0395	0.5
A35	A(8,12,13)	-0.0414	0.5
D2	D(6,1,2,24)	0.0461	0.5
D3	D(6,1,2,25)	0.0306	0.4
D5	D(15,1,2,24)	0.0546	0.6
D6	D(15,1,2,25)	0.0392	0.4
D8	D(23,1,2,24)	0.058	0.7
D9	D(23,1,2,25)	0.0425	0.5
D11	D(15,1,6,5)	-0.0415	0.5
D13	D(2,1,15,16)	-0.1579	1.8
D14	D(2,1,15,20)	0.1545	1.8
D15	D(6,1,15,16)	-0.1267	1.5

```
! D16   D(6,1,15,20)          0.1858              2.1              !
! D17   D(23,1,15,16)        -0.1494              1.7              !
! D18   D(23,1,15,20)         0.1631              1.9              !
! D19   D(1,2,3,4)           -0.031               0.4              !
! D20   D(1,2,3,11)           0.0504              0.6              !
! D21   D(24,2,3,4)          -0.0471              0.5              !
! D22   D(24,2,3,11)          0.0342              0.4              !
! D23   D(25,2,3,4)          -0.0362              0.4              !
! D24   D(25,2,3,11)          0.0451              0.5              !
! D25   D(2,3,4,5)            0.0286              0.3              !
! D27   D(11,3,4,5)          -0.0569              0.7              !
! D28   D(11,3,4,10)         -0.0606              0.7              !
! D30   D(3,4,5,7)           -0.0444              0.5              !
! D32   D(10,4,5,7)          -0.041               0.5              !
! D33   D(3,4,10,9)           0.0279              0.3              !
! D37   D(4,5,6,1)           -0.0414              0.5              !
! D39   D(4,5,7,8)            0.0514              0.6              !
! D40   D(4,5,7,26)           0.0734              0.8              !
! D43   D(5,7,8,9)           -0.0451              0.5              !
! D45   D(26,7,8,9)          -0.0669              0.8              !
! D46   D(26,7,8,12)         -0.0346              0.4              !
! D47   D(7,8,9,10)           0.0297              0.3              !
! D48   D(7,8,9,27)           0.0296              0.3              !
! D63   D(1,15,16,32)        -0.0527              0.6              !
! D64   D(20,15,16,17)       -0.3217              3.7              !
! D65   D(20,15,16,32)       -0.3646              4.2              !
! D66   D(1,15,20,19)         0.0295              0.3              !
! D67   D(1,15,20,33)         0.0418              0.5              !
! D68   D(16,15,20,19)        0.3477              4.0              !
! D69   D(16,15,20,33)        0.36                4.1              !
! D70   D(15,16,17,18)        0.2676              3.1              !
! D71   D(15,16,17,22)        0.0699              0.8              !
! D72   D(32,16,17,18)        0.3103              3.6              !
! D73   D(32,16,17,22)        0.1126              1.3              !
! D74   D(16,17,18,19)       -0.2402              2.8              !
! D75   D(16,17,18,34)       -0.3119              3.6              !
! D76   D(22,17,18,19)       -0.0535              0.6              !
! D77   D(22,17,18,34)       -0.1252              1.4              !
! D78   D(16,17,22,35)        0.0964              1.1              !
! D79   D(18,17,22,35)       -0.0941              1.1              !
! D80   D(17,18,19,20)        0.2689              3.1              !
! D82   D(34,18,19,20)        0.3425              3.9              !
! D83   D(34,18,19,21)        0.0704              0.8              !
! D84   D(18,19,20,15)       -0.3234              3.7              !
! D85   D(18,19,20,33)       -0.3355              3.8              !
! D86   D(21,19,20,15)       -0.0643              0.7              !
! D87   D(21,19,20,33)       -0.0765              0.9              !
! D88   D(18,19,21,36)        0.142               1.6              !
! D89   D(20,19,21,36)       -0.1196              1.4              !
--------------------------------------------------------------------------------
```

! Normal Mode 39 !

Name	Definition	Value	Relative Weight (%)
R5	R(2,3)	0.1349	1.5
R8	R(3,4)	0.151	1.7
R11	R(4,10)	0.0438	0.5
R17	R(8,12)	-0.0818	0.9
R21	R(12,13)	-0.0517	0.6
A2	A(2,1,15)	-0.1194	1.3
A4	A(6,1,15)	0.1139	1.3
A8	A(1,2,24)	-0.0501	0.6
A11	A(3,2,25)	0.0997	1.1
A14	A(2,3,11)	0.0874	1.0
A15	A(4,3,11)	-0.078	0.9
A17	A(3,4,10)	0.0392	0.4
A18	A(5,4,10)	-0.0379	0.4
A19	A(4,5,6)	-0.033	0.4
A20	A(4,5,7)	0.0781	0.9
A21	A(6,5,7)	-0.0453	0.5
A22	A(1,6,5)	0.0685	0.8
A23	A(5,7,8)	-0.1138	1.3
A24	A(5,7,26)	0.0623	0.7
A25	A(8,7,26)	0.0511	0.6
A26	A(7,8,9)	0.1095	1.2
A28	A(9,8,12)	-0.1027	1.1
A29	A(8,9,10)	-0.0415	0.5
A31	A(10,9,27)	0.0637	0.7
A33	A(4,10,14)	-0.0773	0.9
A34	A(9,10,14)	0.0721	0.8
A35	A(8,12,13)	0.0901	1.0
A37	A(12,13,30)	-0.0343	0.4
A42	A(10,14,28)	0.0469	0.5
A44	A(1,15,20)	-0.0311	0.3
A45	A(16,15,20)	0.0356	0.4
A46	A(15,16,17)	-0.0385	0.4
D1	D(6,1,2,3)	0.0294	0.3
D2	D(6,1,2,24)	-0.0499	0.6
D3	D(6,1,2,25)	-0.0827	0.9
D4	D(15,1,2,3)	-0.0469	0.5
D5	D(15,1,2,24)	-0.1262	1.4
D6	D(15,1,2,25)	-0.159	1.8
D7	D(23,1,2,3)	0.0331	0.4
D8	D(23,1,2,24)	-0.0462	0.5
D9	D(23,1,2,25)	-0.079	0.9
D11	D(15,1,6,5)	-0.0502	0.6
D13	D(2,1,15,16)	-0.0912	1.0
D14	D(2,1,15,20)	0.1856	2.0
D15	D(6,1,15,16)	-0.1126	1.2

D16	D(6,1,15,20)	0.1641	1.8
D17	D(23,1,15,16)	-0.1467	1.6
D18	D(23,1,15,20)	0.1301	1.4
D20	D(1,2,3,11)	-0.0497	0.5
D21	D(24,2,3,4)	0.1055	1.2
D22	D(24,2,3,11)	0.0446	0.5
D23	D(25,2,3,4)	0.068	0.8
D25	D(2,3,4,5)	-0.0462	0.5
D26	D(2,3,4,10)	0.0295	0.3
D28	D(11,3,4,10)	0.0906	1.0
D29	D(3,4,5,6)	0.0451	0.5
D31	D(10,4,5,6)	-0.0286	0.3
D32	D(10,4,5,7)	-0.0533	0.6
D34	D(3,4,10,14)	-0.0826	0.9
D35	D(5,4,10,9)	0.0621	0.7
D42	D(6,5,7,26)	-0.0316	0.3
D45	D(26,7,8,9)	0.0314	0.3
D48	D(7,8,9,27)	0.0814	0.9
D50	D(12,8,9,27)	0.0902	1.0
D53	D(8,9,10,4)	-0.0448	0.5
D55	D(27,9,10,4)	-0.1127	1.2
D56	D(27,9,10,14)	-0.0408	0.4
D57	D(4,10,14,28)	0.0466	0.5
D62	D(1,15,16,17)	0.0721	0.8
D63	D(1,15,16,32)	-0.0609	0.7
D64	D(20,15,16,17)	-0.2015	2.2
D65	D(20,15,16,32)	-0.3346	3.7
D66	D(1,15,20,19)	-0.0596	0.7
D68	D(16,15,20,19)	0.2209	2.4
D69	D(16,15,20,33)	0.2845	3.1
D70	D(15,16,17,18)	0.1192	1.3
D71	D(15,16,17,22)	0.0893	1.0
D72	D(32,16,17,18)	0.2518	2.8
D73	D(32,16,17,22)	0.2219	2.4
D74	D(16,17,18,19)	-0.0583	0.6
D75	D(16,17,18,34)	-0.0617	0.7
D76	D(22,17,18,19)	-0.0298	0.3
D77	D(22,17,18,34)	-0.0333	0.4
D79	D(18,17,22,35)	-0.0411	0.5
D80	D(17,18,19,20)	0.0788	0.9
D82	D(34,18,19,20)	0.0823	0.9
D84	D(18,19,20,15)	-0.1601	1.8
D85	D(18,19,20,33)	-0.2232	2.5
D86	D(21,19,20,15)	-0.0845	0.9
D87	D(21,19,20,33)	-0.1475	1.6
D88	D(18,19,21,36)	0.0557	0.6

! Normal Mode 40 !

! Name	Definition	Value	Relative Weight (%)	!
! R8	R(3,4)	-0.0511	0.4	!
! R11	R(4,10)	-0.0615	0.5	!
! R31	R(17,22)	-0.042	0.3	!
! R34	R(19,20)	-0.042	0.3	!
! R35	R(19,21)	-0.0465	0.4	!
! A1	A(2,1,6)	-0.0408	0.3	!
! A2	A(2,1,15)	0.1134	0.9	!
! A3	A(2,1,23)	-0.0394	0.3	!
! A4	A(6,1,15)	0.0772	0.6	!
! A6	A(15,1,23)	-0.0874	0.7	!
! A7	A(1,2,3)	0.0703	0.6	!
! A10	A(3,2,24)	-0.1125	0.9	!
! A22	A(1,6,5)	0.0522	0.4	!
! A28	A(9,8,12)	0.0446	0.4	!
! A29	A(8,9,10)	0.0379	0.3	!
! A44	A(1,15,20)	-0.0681	0.6	!
! A45	A(16,15,20)	0.0695	0.6	!
! A46	A(15,16,17)	-0.0645	0.5	!
! A60	A(19,20,33)	0.042	0.3	!
! D1	D(6,1,2,3)	0.1072	0.9	!
! D3	D(6,1,2,25)	0.0377	0.3	!
! D4	D(15,1,2,3)	-0.0383	0.3	!
! D5	D(15,1,2,24)	-0.1242	1.0	!
! D6	D(15,1,2,25)	-0.1078	0.9	!
! D8	D(23,1,2,24)	-0.0604	0.5	!
! D9	D(23,1,2,25)	-0.044	0.4	!
! D11	D(15,1,6,5)	0.1359	1.1	!
! D12	D(23,1,6,5)	0.0575	0.5	!
! D13	D(2,1,15,16)	0.0819	0.7	!
! D16	D(6,1,15,20)	-0.0805	0.7	!
! D17	D(23,1,15,16)	0.0449	0.4	!
! D18	D(23,1,15,20)	-0.0401	0.3	!
! D19	D(1,2,3,4)	0.0863	0.7	!
! D20	D(1,2,3,11)	-0.0659	0.5	!
! D21	D(24,2,3,4)	0.0974	0.8	!
! D22	D(24,2,3,11)	-0.0548	0.5	!
! D23	D(25,2,3,4)	0.1674	1.4	!
! D25	D(2,3,4,5)	-0.2961	2.5	!
! D26	D(2,3,4,10)	0.1918	1.6	!
! D27	D(11,3,4,5)	-0.1339	1.1	!
! D28	D(11,3,4,10)	0.354	2.9	!
! D29	D(3,4,5,6)	0.409	3.4	!
! D31	D(10,4,5,6)	-0.0461	0.4	!
! D32	D(10,4,5,7)	-0.4554	3.8	!
! D33	D(3,4,10,9)	-0.0752	0.6	!
! D34	D(3,4,10,14)	-0.3764	3.1	!
! D35	D(5,4,10,9)	0.4005	3.3	!
! D36	D(5,4,10,14)	0.0993	0.8	!

! D37	D(4,5,6,1)	-0.257	2.1	!
! D38	D(7,5,6,1)	0.1252	1.0	!
! D39	D(4,5,7,8)	0.3013	2.5	!
! D40	D(4,5,7,26)	0.3279	2.7	!
! D41	D(6,5,7,8)	-0.0817	0.7	!
! D42	D(6,5,7,26)	-0.0551	0.5	!
! D43	D(5,7,8,9)	-0.0766	0.6	!
! D44	D(5,7,8,12)	-0.1328	1.1	!
! D45	D(26,7,8,9)	-0.1032	0.9	!
! D46	D(26,7,8,12)	-0.1593	1.3	!
! D48	D(7,8,9,27)	0.3865	3.2	!
! D49	D(12,8,9,10)	0.089	0.7	!
! D50	D(12,8,9,27)	0.448	3.7	!
! D52	D(9,8,12,13)	-0.072	0.6	!
! D53	D(8,9,10,4)	-0.2001	1.7	!
! D54	D(8,9,10,14)	0.106	0.9	!
! D55	D(27,9,10,4)	-0.5507	4.6	!
! D56	D(27,9,10,14)	-0.2446	2.0	!
! D57	D(4,10,14,28)	0.2345	1.9	!
! D58	D(9,10,14,28)	-0.0622	0.5	!
! D60	D(8,12,13,30)	0.0487	0.4	!
! D61	D(8,12,13,31)	0.0448	0.4	!
! D62	D(1,15,16,17)	-0.048	0.4	!
! D63	D(1,15,16,32)	0.0687	0.6	!
! D64	D(20,15,16,17)	0.0408	0.3	!
! D65	D(20,15,16,32)	0.1574	1.3	!
! D66	D(1,15,20,19)	0.0439	0.4	!
! D68	D(16,15,20,19)	-0.0447	0.4	!
! D69	D(16,15,20,33)	-0.1117	0.9	!
! D72	D(32,16,17,18)	-0.1321	1.1	!
! D73	D(32,16,17,22)	-0.1439	1.2	!
! D85	D(18,19,20,33)	0.0893	0.7	!
! D87	D(21,19,20,33)	0.0949	0.8	!

! Normal Mode 41 !

! Name	Definition	Value	Relative Weight (%)	!
! R2	R(1,6)	-0.0493	0.5	!
! R5	R(2,3)	-0.0362	0.4	!
! R11	R(4,10)	0.0613	0.6	!
! R28	R(16,17)	0.0446	0.5	!
! R31	R(17,22)	0.0526	0.5	!
! R34	R(19,20)	0.0433	0.4	!
! R35	R(19,21)	0.0524	0.5	!
! A1	A(2,1,6)	0.0825	0.8	!
! A2	A(2,1,15)	-0.0563	0.6	!
! A4	A(6,1,15)	-0.0649	0.7	!
! A6	A(15,1,23)	0.0557	0.6	!

```
! A7    A(1,2,3)              -0.0833              0.8                   !
! A11   A(3,2,25)              0.0689              0.7                   !
! A15   A(4,3,11)             -0.0437              0.4                   !
! A19   A(4,5,6)               0.0463              0.5                   !
! A20   A(4,5,7)              -0.0344              0.3                   !
! A22   A(1,6,5)              -0.1077              1.1                   !
! A23   A(5,7,8)               0.0368              0.4                   !
! A24   A(5,7,26)             -0.0314              0.3                   !
! A27   A(7,8,12)              0.0315              0.3                   !
! A28   A(9,8,12)             -0.0441              0.4                   !
! A29   A(8,9,10)             -0.0364              0.4                   !
! A43   A(1,15,16)             0.0456              0.5                   !
! A44   A(1,15,20)             0.0368              0.4                   !
! A45   A(16,15,20)           -0.0822              0.8                   !
! A46   A(15,16,17)            0.0654              0.7                   !
! A48   A(17,16,32)           -0.0386              0.4                   !
! A58   A(15,20,19)            0.0478              0.5                   !
! A60   A(19,20,33)           -0.0458              0.5                   !
! D1    D(6,1,2,3)            -0.088               0.9                   !
! D2    D(6,1,2,24)           -0.1432              1.4                   !
! D3    D(6,1,2,25)           -0.1122              1.1                   !
! D5    D(15,1,2,24)          -0.0816              0.8                   !
! D6    D(15,1,2,25)          -0.0506              0.5                   !
! D7    D(23,1,2,3)           -0.0419              0.4                   !
! D8    D(23,1,2,24)          -0.0971              1.0                   !
! D9    D(23,1,2,25)          -0.0661              0.7                   !
! D10   D(2,1,6,5)             0.0914              0.9                   !
! D11   D(15,1,6,5)            0.0324              0.3                   !
! D12   D(23,1,6,5)            0.0698              0.7                   !
! D19   D(1,2,3,4)             0.0705              0.7                   !
! D20   D(1,2,3,11)           -0.0779              0.8                   !
! D21   D(24,2,3,4)            0.098               1.0                   !
! D22   D(24,2,3,11)          -0.0505              0.5                   !
! D23   D(25,2,3,4)            0.0522              0.5                   !
! D24   D(25,2,3,11)          -0.0963              1.0                   !
! D25   D(2,3,4,5)            -0.1604              1.6                   !
! D28   D(11,3,4,10)           0.134               1.4                   !
! D29   D(3,4,5,6)             0.1796              1.8                   !
! D31   D(10,4,5,6)            0.0498              0.5                   !
! D32   D(10,4,5,7)           -0.1083              1.1                   !
! D33   D(3,4,10,9)           -0.1589              1.6                   !
! D34   D(3,4,10,14)          -0.0334              0.3                   !
! D36   D(5,4,10,14)           0.0995              1.0                   !
! D37   D(4,5,6,1)            -0.1077              1.1                   !
! D38   D(7,5,6,1)             0.0402              0.4                   !
! D39   D(4,5,7,8)             0.0582              0.6                   !
! D40   D(4,5,7,26)            0.1174              1.2                   !
! D41   D(6,5,7,8)            -0.0892              0.9                   !
! D43   D(5,7,8,9)             0.1298              1.3                   !
! D44   D(5,7,8,12)           -0.0929              0.9                   !
```

D45	D(26,7,8,9)	0.0706	0.7
D46	D(26,7,8,12)	-0.152	1.5
D47	D(7,8,9,10)	-0.2605	2.6
D48	D(7,8,9,27)	0.5174	5.2
D50	D(12,8,9,27)	0.7583	7.7
D51	D(7,8,12,13)	0.0318	0.3
D52	D(9,8,12,13)	-0.1985	2.0
D53	D(8,9,10,4)	0.2082	2.1
D54	D(8,9,10,14)	0.0808	0.8
D55	D(27,9,10,4)	-0.5517	5.6
D56	D(27,9,10,14)	-0.6791	6.9
D57	D(4,10,14,28)	0.0464	0.5
D58	D(9,10,14,28)	0.17	1.7
D59	D(8,12,13,29)	0.0779	0.8
D60	D(8,12,13,30)	0.0847	0.9
D61	D(8,12,13,31)	0.0702	0.7
D63	D(1,15,16,32)	-0.0586	0.6
D65	D(20,15,16,32)	-0.0656	0.7
D72	D(32,16,17,18)	0.0611	0.6
D73	D(32,16,17,22)	0.0692	0.7

! Normal Mode 42 !

Name	Definition	Value	Relative Weight (%)
R5	R(2,3)	0.0426	0.4
R11	R(4,10)	-0.048	0.4
R31	R(17,22)	-0.04	0.3
R35	R(19,21)	-0.0381	0.3
A1	A(2,1,6)	-0.0765	0.6
A4	A(6,1,15)	0.0406	0.3
A7	A(1,2,3)	0.065	0.5
A10	A(3,2,24)	0.0606	0.5
A11	A(3,2,25)	-0.0823	0.7
A15	A(4,3,11)	0.0387	0.3
A17	A(3,4,10)	-0.0389	0.3
A19	A(4,5,6)	-0.04	0.3
A22	A(1,6,5)	0.0809	0.7
A43	A(1,15,16)	-0.04	0.3
A45	A(16,15,20)	0.0606	0.5
A46	A(15,16,17)	-0.0458	0.4
A58	A(15,20,19)	-0.0382	0.3
D1	D(6,1,2,3)	0.0488	0.4
D2	D(6,1,2,24)	0.149	1.2
D3	D(6,1,2,25)	0.114	0.9
D5	D(15,1,2,24)	0.1317	1.1
D6	D(15,1,2,25)	0.0968	0.8
D8	D(23,1,2,24)	0.1262	1.0
D9	D(23,1,2,25)	0.0913	0.8

D10	D(2,1,6,5)	-0.0439	0.4
D12	D(23,1,6,5)	-0.0428	0.4
D19	D(1,2,3,4)	-0.1055	0.9
D20	D(1,2,3,11)	0.1002	0.8
D21	D(24,2,3,4)	-0.1544	1.3
D22	D(24,2,3,11)	0.0513	0.4
D23	D(25,2,3,4)	-0.1286	1.1
D24	D(25,2,3,11)	0.0772	0.6
D25	D(2,3,4,5)	0.2411	2.0
D26	D(2,3,4,10)	-0.1598	1.3
D28	D(11,3,4,10)	-0.3764	3.1
D29	D(3,4,5,6)	-0.2571	2.1
D30	D(3,4,5,7)	-0.0702	0.6
D31	D(10,4,5,6)	0.118	1.0
D32	D(10,4,5,7)	0.3049	2.5
D33	D(3,4,10,9)	-0.0743	0.6
D34	D(3,4,10,14)	0.4132	3.4
D35	D(5,4,10,9)	-0.4623	3.8
D37	D(4,5,6,1)	0.1229	1.0
D38	D(7,5,6,1)	-0.0518	0.4
D39	D(4,5,7,8)	-0.1797	1.5
D40	D(4,5,7,26)	-0.3064	2.5
D42	D(6,5,7,26)	-0.132	1.1
D43	D(5,7,8,9)	0.1975	1.6
D45	D(26,7,8,9)	0.3241	2.7
D46	D(26,7,8,12)	0.1213	1.0
D47	D(7,8,9,10)	-0.3547	2.9
D48	D(7,8,9,27)	0.5097	4.2
D49	D(12,8,9,10)	-0.1341	1.1
D50	D(12,8,9,27)	0.7303	6.1
D52	D(9,8,12,13)	-0.185	1.5
D53	D(8,9,10,4)	0.4967	4.1
D55	D(27,9,10,4)	-0.3477	2.9
D56	D(27,9,10,14)	-0.844	7.0
D57	D(4,10,14,28)	-0.1593	1.3
D58	D(9,10,14,28)	0.3211	2.7
D59	D(8,12,13,29)	0.0473	0.4
D60	D(8,12,13,30)	0.0616	0.5
D61	D(8,12,13,31)	0.055	0.5

! Normal Mode 43 !

Name	Definition	Value	Relative Weight (%)
R1	R(1,2)	-0.0281	0.4
A2	A(2,1,15)	-0.0398	0.6
A3	A(2,1,23)	0.0299	0.4
A8	A(1,2,24)	-0.0257	0.4
D3	D(6,1,2,25)	-0.0311	0.5

! D6	D(15,1,2,25)	-0.0242	0.4	!
! D11	D(15,1,6,5)	-0.0311	0.5	!
! D12	D(23,1,6,5)	-0.0329	0.5	!
! D13	D(2,1,15,16)	-0.1324	2.0	!
! D14	D(2,1,15,20)	0.0642	1.0	!
! D15	D(6,1,15,16)	-0.1279	1.9	!
! D16	D(6,1,15,20)	0.0687	1.0	!
! D17	D(23,1,15,16)	-0.1221	1.8	!
! D18	D(23,1,15,20)	0.0745	1.1	!
! D22	D(24,2,3,11)	0.0342	0.5	!
! D24	D(25,2,3,11)	0.0249	0.4	!
! D25	D(2,3,4,5)	0.0215	0.3	!
! D28	D(11,3,4,10)	-0.0218	0.3	!
! D62	D(1,15,16,17)	-0.0793	1.2	!
! D63	D(1,15,16,32)	0.7866	11.7	!
! D64	D(20,15,16,17)	-0.2757	4.1	!
! D65	D(20,15,16,32)	0.5903	8.8	!
! D66	D(1,15,20,19)	-0.0446	0.7	!
! D67	D(1,15,20,33)	-0.0817	1.2	!
! D68	D(16,15,20,19)	0.1558	2.3	!
! D69	D(16,15,20,33)	0.1186	1.8	!
! D70	D(15,16,17,18)	0.2522	3.7	!
! D71	D(15,16,17,22)	0.0699	1.0	!
! D72	D(32,16,17,18)	-0.611	9.1	!
! D73	D(32,16,17,22)	-0.7933	11.8	!
! D74	D(16,17,18,19)	-0.1088	1.6	!
! D75	D(16,17,18,34)	-0.2591	3.8	!
! D76	D(22,17,18,19)	0.0633	0.9	!
! D77	D(22,17,18,34)	-0.087	1.3	!
! D78	D(16,17,22,35)	0.1762	2.6	!
! D81	D(17,18,19,21)	0.0348	0.5	!
! D82	D(34,18,19,20)	0.1412	2.1	!
! D83	D(34,18,19,21)	0.1889	2.8	!
! D85	D(18,19,20,33)	0.0257	0.4	!
! D86	D(21,19,20,15)	-0.0562	0.8	!
! D88	D(18,19,21,36)	-0.0463	0.7	!

! Normal Mode 44 !

! Name	Definition	Value	Relative Weight (%)	!
! D62	D(1,15,16,17)	-0.0636	1.1	!
! D63	D(1,15,16,32)	0.1165	2.0	!
! D64	D(20,15,16,17)	-0.0922	1.6	!
! D65	D(20,15,16,32)	0.0878	1.5	!
! D66	D(1,15,20,19)	0.0366	0.6	!
! D68	D(16,15,20,19)	0.0659	1.1	!
! D69	D(16,15,20,33)	0.0524	0.9	!
! D70	D(15,16,17,18)	0.2217	3.8	!

D71	D(15,16,17,22)	-0.0459	0.8
D72	D(32,16,17,18)	0.0423	0.7
D73	D(32,16,17,22)	-0.2253	3.9
D74	D(16,17,18,19)	-0.3211	5.5
D75	D(16,17,18,34)	0.5719	9.9
D76	D(22,17,18,19)	-0.0684	1.2
D77	D(22,17,18,34)	0.8246	14.2
D78	D(16,17,22,35)	0.1016	1.8
D79	D(18,17,22,35)	-0.1564	2.7
D80	D(17,18,19,20)	0.2957	5.1
D81	D(17,18,19,21)	0.0754	1.3
D82	D(34,18,19,20)	-0.6195	10.7
D83	D(34,18,19,21)	-0.8398	14.5
D84	D(18,19,20,15)	-0.1696	2.9
D85	D(18,19,20,33)	-0.1563	2.7
D86	D(21,19,20,15)	0.0401	0.7
D87	D(21,19,20,33)	0.0534	0.9
D88	D(18,19,21,36)	0.1911	3.3

! Normal Mode 45 !

Name	Definition	Value	Relative Weight (%)
R2	R(1,6)	0.0215	0.3
D10	D(2,1,6,5)	-0.0616	1.0
D11	D(15,1,6,5)	-0.0602	0.9
D12	D(23,1,6,5)	-0.0631	1.0
D25	D(2,3,4,5)	0.0659	1.0
D27	D(11,3,4,5)	0.0513	0.8
D29	D(3,4,5,6)	-0.1287	2.0
D30	D(3,4,5,7)	0.1276	2.0
D31	D(10,4,5,6)	-0.0634	1.0
D32	D(10,4,5,7)	0.1929	3.0
D35	D(5,4,10,9)	-0.06	0.9
D36	D(5,4,10,14)	-0.0591	0.9
D37	D(4,5,6,1)	0.1225	1.9
D38	D(7,5,6,1)	-0.117	1.8
D39	D(4,5,7,8)	-0.3145	4.9
D40	D(4,5,7,26)	0.5916	9.2
D41	D(6,5,7,8)	-0.0747	1.2
D42	D(6,5,7,26)	0.8314	12.9
D43	D(5,7,8,9)	0.2999	4.6
D44	D(5,7,8,12)	0.0871	1.3
D45	D(26,7,8,9)	-0.6081	9.4
D46	D(26,7,8,12)	-0.8208	12.7
D47	D(7,8,9,10)	-0.1733	2.7
D48	D(7,8,9,27)	-0.1107	1.7
D49	D(12,8,9,10)	0.0575	0.9
D50	D(12,8,9,27)	0.1202	1.9

D51	D(7,8,12,13)	0.1062	1.6
D52	D(9,8,12,13)	-0.1144	1.8
D53	D(8,9,10,4)	0.0535	0.8
D54	D(8,9,10,14)	0.0526	0.8
D59	D(8,12,13,29)	0.0603	0.9
D60	D(8,12,13,30)	0.0584	0.9
D61	D(8,12,13,31)	0.0585	0.9

! Normal Mode 46 !

Name	Definition	Value	Relative Weight (%)
R1	R(1,2)	-0.0326	0.5
A2	A(2,1,15)	-0.0218	0.4
A11	A(3,2,25)	0.0187	0.3
D3	D(6,1,2,25)	-0.0232	0.4
D6	D(15,1,2,25)	-0.0244	0.4
D11	D(15,1,6,5)	-0.0213	0.4
D12	D(23,1,6,5)	-0.0272	0.5
D13	D(2,1,15,16)	-0.0304	0.5
D14	D(2,1,15,20)	0.1214	2.0
D15	D(6,1,15,16)	-0.0315	0.5
D16	D(6,1,15,20)	0.1203	2.0
D17	D(23,1,15,16)	-0.0313	0.5
D18	D(23,1,15,20)	0.1204	2.0
D22	D(24,2,3,11)	0.0263	0.4
D24	D(25,2,3,11)	0.0226	0.4
D25	D(2,3,4,5)	0.0265	0.4
D28	D(11,3,4,10)	-0.025	0.4
D29	D(3,4,5,6)	-0.0198	0.3
D62	D(1,15,16,17)	0.032	0.5
D64	D(20,15,16,17)	-0.1196	2.0
D65	D(20,15,16,32)	-0.1449	2.4
D66	D(1,15,20,19)	0.0883	1.5
D67	D(1,15,20,33)	-0.7891	13.2
D68	D(16,15,20,19)	0.2428	4.1
D69	D(16,15,20,33)	-0.6345	10.6
D71	D(15,16,17,22)	0.0306	0.5
D72	D(32,16,17,18)	0.0353	0.6
D73	D(32,16,17,22)	0.0558	0.9
D74	D(16,17,18,19)	-0.0266	0.4
D76	D(22,17,18,19)	-0.046	0.8
D77	D(22,17,18,34)	-0.0254	0.4
D80	D(17,18,19,20)	0.1537	2.6
D81	D(17,18,19,21)	-0.0538	0.9
D82	D(34,18,19,20)	0.1325	2.2
D83	D(34,18,19,21)	-0.0749	1.3
D84	D(18,19,20,15)	-0.2616	4.4
D85	D(18,19,20,33)	0.6004	10.0

D86	D(21,19,20,15)	-0.0641	1.1
D87	D(21,19,20,33)	0.7979	13.3
D88	D(18,19,21,36)	0.0643	1.1
D89	D(20,19,21,36)	-0.1351	2.3

! Normal Mode 47 !

Name	Definition	Value	Relative Weight (%)
R1	R(1,2)	0.2262	2.7
R2	R(1,6)	0.2851	3.4
R5	R(2,3)	-0.1322	1.6
R10	R(4,5)	-0.035	0.4
R12	R(5,6)	0.0498	0.6
R13	R(5,7)	-0.0387	0.5
R14	R(7,8)	-0.0638	0.8
R16	R(8,9)	-0.0365	0.4
R17	R(8,12)	-0.0553	0.7
R21	R(12,13)	-0.0356	0.4
R28	R(16,17)	-0.0287	0.3
R31	R(17,22)	-0.0297	0.4
A3	A(2,1,23)	0.0842	1.0
A4	A(6,1,15)	-0.0597	0.7
A5	A(6,1,23)	-0.0851	1.0
A7	A(1,2,3)	-0.0488	0.6
A8	A(1,2,24)	-0.1894	2.3
A9	A(1,2,25)	0.2864	3.5
A10	A(3,2,24)	-0.1216	1.5
A11	A(3,2,25)	0.063	0.8
A13	A(2,3,4)	0.1273	1.5
A14	A(2,3,11)	-0.0902	1.1
A15	A(4,3,11)	-0.0335	0.4
A17	A(3,4,10)	0.039	0.5
A18	A(5,4,10)	-0.0342	0.4
A19	A(4,5,6)	0.0726	0.9
A21	A(6,5,7)	-0.0953	1.2
A22	A(1,6,5)	-0.1183	1.4
A23	A(5,7,8)	0.0344	0.4
A24	A(5,7,26)	-0.0791	1.0
A25	A(8,7,26)	0.044	0.5
A26	A(7,8,9)	-0.0436	0.5
A27	A(7,8,12)	0.0641	0.8
A29	A(8,9,10)	0.04	0.5
A30	A(8,9,27)	-0.0464	0.6
A33	A(4,10,14)	-0.0314	0.4
A34	A(9,10,14)	0.0501	0.6
A35	A(8,12,13)	0.0378	0.5
A37	A(12,13,30)	-0.0272	0.3
A43	A(1,15,16)	-0.0469	0.6

A58	A(15,20,19)	-0.0293	0.4
D1	D(6,1,2,3)	0.0793	1.0
D2	D(6,1,2,24)	-0.2269	2.7
D3	D(6,1,2,25)	-0.1662	2.0
D4	D(15,1,2,3)	0.1343	1.6
D5	D(15,1,2,24)	-0.1718	2.1
D6	D(15,1,2,25)	-0.1112	1.3
D7	D(23,1,2,3)	0.0361	0.4
D8	D(23,1,2,24)	-0.2701	3.3
D9	D(23,1,2,25)	-0.2094	2.5
D11	D(15,1,6,5)	-0.0343	0.4
D12	D(23,1,6,5)	-0.0843	1.0
D15	D(6,1,15,16)	0.027	0.3
D17	D(23,1,15,16)	0.1487	1.8
D18	D(23,1,15,20)	0.1327	1.6
D19	D(1,2,3,4)	-0.1169	1.4
D21	D(24,2,3,4)	0.2274	2.7
D22	D(24,2,3,11)	0.3587	4.3
D23	D(25,2,3,4)	0.254	3.1
D24	D(25,2,3,11)	0.3853	4.7
D25	D(2,3,4,5)	0.0946	1.1
D27	D(11,3,4,5)	-0.0434	0.5
D28	D(11,3,4,10)	-0.1259	1.5
D29	D(3,4,5,6)	-0.0336	0.4
D30	D(3,4,5,7)	-0.0579	0.7
D31	D(10,4,5,6)	0.0403	0.5
D33	D(3,4,10,9)	0.0467	0.6
D34	D(3,4,10,14)	0.0764	0.9
D35	D(5,4,10,9)	-0.0326	0.4
D40	D(4,5,7,26)	-0.0521	0.6
D42	D(6,5,7,26)	-0.0737	0.9
D45	D(26,7,8,9)	0.0423	0.5
D46	D(26,7,8,12)	0.0551	0.7
D63	D(1,15,16,32)	-0.0342	0.4
D69	D(16,15,20,33)	-0.028	0.3
D73	D(32,16,17,22)	0.0301	0.4

! Normal Mode 48 !

Name	Definition	Value	Relative Weight (%)
R1	R(1,2)	-0.0911	0.8
R2	R(1,6)	0.1854	1.7
R3	R(1,15)	-0.036	0.3
R5	R(2,3)	0.165	1.5
R8	R(3,4)	-0.0547	0.5
R11	R(4,10)	-0.0547	0.5
R12	R(5,6)	0.129	1.2
R14	R(7,8)	-0.1015	0.9

```
! R16   R(8,9)                 -0.1295                1.2                   !
! R17   R(8,12)                -0.1632                1.5                   !
! R21   R(12,13)               -0.278                 2.6                   !
! R26   R(15,16)               -0.0385                0.4                   !
! R28   R(16,17)               -0.0461                0.4                   !
! R30   R(17,18)               -0.0408                0.4                   !
! R31   R(17,22)               -0.0542                0.5                   !
! R32   R(18,19)                0.0594                0.5                   !
! R34   R(19,20)                0.0638                0.6                   !
! R35   R(19,21)                0.08                  0.7                   !
! A1    A(2,1,6)               -0.0805                0.7                   !
! A4    A(6,1,15)              -0.1027                0.9                   !
! A5    A(6,1,23)               0.0705                0.7                   !
! A6    A(15,1,23)              0.1243                1.1                   !
! A8    A(1,2,24)               0.1606                1.5                   !
! A9    A(1,2,25)              -0.1645                1.5                   !
! A10   A(3,2,24)               0.2261                2.1                   !
! A11   A(3,2,25)              -0.1922                1.8                   !
! A20   A(4,5,7)               -0.0534                0.5                   !
! A22   A(1,6,5)               -0.0793                0.7                   !
! A23   A(5,7,8)                0.119                 1.1                   !
! A24   A(5,7,26)              -0.0486                0.4                   !
! A25   A(8,7,26)              -0.0707                0.7                   !
! A26   A(7,8,9)               -0.1617                1.5                   !
! A27   A(7,8,12)               0.1126                1.0                   !
! A28   A(9,8,12)               0.0492                0.5                   !
! A29   A(8,9,10)               0.1834                1.7                   !
! A30   A(8,9,27)              -0.1155                1.1                   !
! A31   A(10,9,27)             -0.0679                0.6                   !
! A32   A(4,10,9)              -0.0709                0.7                   !
! A34   A(9,10,14)              0.1006                0.9                   !
! A35   A(8,12,13)              0.0668                0.6                   !
! A36   A(12,13,29)             0.0408                0.4                   !
! A38   A(12,13,31)             0.0404                0.4                   !
! A42   A(10,14,28)             0.0387                0.4                   !
! A46   A(15,16,17)             0.0613                0.6                   !
! A50   A(16,17,22)             0.0394                0.4                   !
! A53   A(17,18,34)            -0.0539                0.5                   !
! A54   A(19,18,34)             0.0725                0.7                   !
! A58   A(15,20,19)            -0.0543                0.5                   !
! A62   A(17,22,35)             0.0438                0.4                   !
! D1    D(6,1,2,3)             -0.2178                2.0                   !
! D2    D(6,1,2,24)             0.1486                1.4                   !
! D3    D(6,1,2,25)             0.1642                1.5                   !
! D5    D(15,1,2,24)            0.3391                3.1                   !
! D6    D(15,1,2,25)            0.3547                3.3                   !
! D7    D(23,1,2,3)            -0.1815                1.7                   !
! D8    D(23,1,2,24)            0.1849                1.7                   !
! D9    D(23,1,2,25)            0.2005                1.9                   !
! D10   D(2,1,6,5)              0.133                 1.2                   !
```

! D12	D(23,1,6,5)	0.1422	1.3	!
! D13	D(2,1,15,16)	-0.1347	1.2	!
! D14	D(2,1,15,20)	-0.1417	1.3	!
! D15	D(6,1,15,16)	0.0422	0.4	!
! D16	D(6,1,15,20)	0.0352	0.3	!
! D17	D(23,1,15,16)	-0.0528	0.5	!
! D18	D(23,1,15,20)	-0.0598	0.6	!
! D19	D(1,2,3,4)	0.258	2.4	!
! D21	D(24,2,3,4)	-0.0685	0.6	!
! D22	D(24,2,3,11)	-0.3025	2.8	!
! D23	D(25,2,3,4)	-0.1007	0.9	!
! D24	D(25,2,3,11)	-0.3347	3.1	!
! D25	D(2,3,4,5)	-0.1856	1.7	!
! D26	D(2,3,4,10)	-0.0522	0.5	!
! D27	D(11,3,4,5)	0.0626	0.6	!
! D28	D(11,3,4,10)	0.196	1.8	!
! D29	D(3,4,5,6)	0.142	1.3	!
! D30	D(3,4,5,7)	0.0621	0.6	!
! D32	D(10,4,5,7)	-0.0643	0.6	!
! D33	D(3,4,10,9)	-0.0755	0.7	!
! D34	D(3,4,10,14)	-0.1063	1.0	!
! D35	D(5,4,10,9)	0.0544	0.5	!
! D37	D(4,5,6,1)	-0.0972	0.9	!
! D39	D(4,5,7,8)	0.0462	0.4	!
! D42	D(6,5,7,26)	-0.0565	0.5	!

! Normal Mode 49 !

! Name	Definition	Value	Relative Weight (%)	!
! R1	R(1,2)	-0.0659	0.6	!
! R2	R(1,6)	-0.1863	1.8	!
! R3	R(1,15)	-0.1431	1.4	!
! R5	R(2,3)	-0.1134	1.1	!
! R12	R(5,6)	0.0565	0.6	!
! R13	R(5,7)	0.0517	0.5	!
! R16	R(8,9)	-0.0752	0.7	!
! R17	R(8,12)	-0.129	1.3	!
! R18	R(9,10)	0.045	0.4	!
! R20	R(10,14)	0.0878	0.9	!
! R21	R(12,13)	-0.2781	2.7	!
! R26	R(15,16)	-0.0504	0.5	!
! R27	R(15,20)	-0.1026	1.0	!
! R28	R(16,17)	0.1371	1.3	!
! R30	R(17,18)	0.1089	1.1	!
! R31	R(17,22)	0.1892	1.9	!
! R32	R(18,19)	-0.0514	0.5	!
! R34	R(19,20)	-0.0665	0.7	!
! R35	R(19,21)	-0.0617	0.6	!

```
! A1    A(2,1,6)                0.0551                  0.5                   !
! A2    A(2,1,15)               0.0923                  0.9                   !
! A4    A(6,1,15)               0.0445                  0.4                   !
! A6    A(15,1,23)             -0.1748                  1.7                   !
! A7    A(1,2,3)                0.115                   1.1                   !
! A8    A(1,2,24)              -0.1064                  1.0                   !
! A9    A(1,2,25)               0.0743                  0.7                   !
! A10   A(3,2,24)              -0.197                   1.9                   !
! A11   A(3,2,25)               0.1224                  1.2                   !
! A14   A(2,3,11)               0.047                   0.5                   !
! A15   A(4,3,11)              -0.0361                  0.4                   !
! A19   A(4,5,6)               -0.0387                  0.4                   !
! A20   A(4,5,7)               -0.0326                  0.3                   !
! A21   A(6,5,7)                0.0721                  0.7                   !
! A22   A(1,6,5)                0.0645                  0.6                   !
! A23   A(5,7,8)                0.0883                  0.9                   !
! A25   A(8,7,26)              -0.0756                  0.7                   !
! A26   A(7,8,9)               -0.0941                  0.9                   !
! A27   A(7,8,12)               0.091                   0.9                   !
! A29   A(8,9,10)               0.0828                  0.8                   !
! A30   A(8,9,27)              -0.0686                  0.7                   !
! A34   A(9,10,14)              0.0419                  0.4                   !
! A35   A(8,12,13)              0.0591                  0.6                   !
! A36   A(12,13,29)             0.039                   0.4                   !
! A38   A(12,13,31)             0.0399                  0.4                   !
! A44   A(1,15,20)              0.0343                  0.3                   !
! A45   A(16,15,20)            -0.0471                  0.5                   !
! A46   A(15,16,17)            -0.0364                  0.4                   !
! A47   A(15,16,32)            -0.0449                  0.4                   !
! A48   A(17,16,32)             0.0811                  0.8                   !
! A52   A(17,18,19)            -0.0534                  0.5                   !
! A53   A(17,18,34)             0.1292                  1.3                   !
! A54   A(19,18,34)            -0.0757                  0.7                   !
! A56   A(18,19,21)            -0.0614                  0.6                   !
! A57   A(20,19,21)             0.0925                  0.9                   !
! A58   A(15,20,19)             0.1491                  1.5                   !
! A59   A(15,20,33)            -0.1004                  1.0                   !
! A60   A(19,20,33)            -0.05                    0.5                   !
! A61   A(19,21,36)            -0.0668                  0.7                   !
! A62   A(17,22,35)            -0.0411                  0.4                   !
! D1    D(6,1,2,3)              0.1826                  1.8                   !
! D2    D(6,1,2,24)            -0.0599                  0.6                   !
! D3    D(6,1,2,25)            -0.1048                  1.0                   !
! D5    D(15,1,2,24)           -0.2181                  2.1                   !
! D6    D(15,1,2,25)           -0.263                   2.6                   !
! D7    D(23,1,2,3)             0.2054                  2.0                   !
! D8    D(23,1,2,24)           -0.0372                  0.4                   !
! D9    D(23,1,2,25)           -0.0821                  0.8                   !
! D10   D(2,1,6,5)             -0.1521                  1.5                   !
! D12   D(23,1,6,5)            -0.1533                  1.5                   !
```

```
! D13   D(2,1,15,16)            0.1379                 1.3                   !
! D14   D(2,1,15,20)            0.0519                 0.5                   !
! D16   D(6,1,15,20)           -0.1105                 1.1                   !
! D17   D(23,1,15,16)           0.0399                 0.4                   !
! D18   D(23,1,15,20)          -0.0461                 0.5                   !
! D19   D(1,2,3,4)             -0.2124                 2.1                   !
! D22   D(24,2,3,11)            0.199                  1.9                   !
! D23   D(25,2,3,4)             0.0392                 0.4                   !
! D24   D(25,2,3,11)            0.2614                 2.6                   !
! D25   D(2,3,4,5)              0.1788                 1.7                   !
! D26   D(2,3,4,10)             0.0554                 0.5                   !
! D27   D(11,3,4,5)            -0.0579                 0.6                   !
! D28   D(11,3,4,10)           -0.1812                 1.8                   !
! D29   D(3,4,5,6)             -0.1255                 1.2                   !
! D30   D(3,4,5,7)             -0.0578                 0.6                   !
! D32   D(10,4,5,7)             0.0556                 0.5                   !
! D33   D(3,4,10,9)             0.0714                 0.7                   !
! D34   D(3,4,10,14)            0.0973                 1.0                   !
! D35   D(5,4,10,9)            -0.0475                 0.5                   !
! D37   D(4,5,6,1)              0.101                  1.0                   !
! D38   D(7,5,6,1)              0.0385                 0.4                   !
! D39   D(4,5,7,8)             -0.0365                 0.4                   !
! D42   D(6,5,7,26)             0.0381                 0.4                   !
! D63   D(1,15,16,32)          -0.072                  0.7                   !
! D64   D(20,15,16,17)          0.0527                 0.5                   !
! D67   D(1,15,20,33)           0.107                  1.0                   !
! D68   D(16,15,20,19)         -0.0559                 0.5                   !
! D84   D(18,19,20,15)          0.0326                 0.3                   !
! D85   D(18,19,20,33)         -0.0438                 0.4                   !
! D87   D(21,19,20,33)         -0.0503                 0.5                   !
--------------------------------------------------------------------------------
                          ----------------------------
                          ! Normal Mode    50        !
--------------------------                            --------------------------
! Name  Definition              Value         Relative Weight (%)            !
--------------------------------------------------------------------------------
! R2    R(1,6)                  0.1554                 2.3                   !
! R3    R(1,15)                -0.2284                 3.4                   !
! R5    R(2,3)                  0.207                  3.1                   !
! R8    R(3,4)                 -0.0257                 0.4                   !
! R11   R(4,10)                -0.0753                 1.1                   !
! R17   R(8,12)                 0.0586                 0.9                   !
! R18   R(9,10)                -0.0745                 1.1                   !
! R20   R(10,14)               -0.0866                 1.3                   !
! R21   R(12,13)                0.1256                 1.9                   !
! R28   R(16,17)                0.1708                 2.5                   !
! R30   R(17,18)                0.1791                 2.6                   !
! R31   R(17,22)                0.0935                 1.4                   !
! R32   R(18,19)                0.1719                 2.5                   !
! R34   R(19,20)                0.1858                 2.7                   !
```

```
! R35   R(19,21)              0.0853              1.3                  !
! A1    A(2,1,6)             -0.0268              0.4                  !
! A2    A(2,1,15)             0.0463              0.7                  !
! A3    A(2,1,23)             0.0484              0.7                  !
! A4    A(6,1,15)             0.0308              0.5                  !
! A5    A(6,1,23)            -0.0425              0.6                  !
! A6    A(15,1,23)           -0.0615              0.9                  !
! A8    A(1,2,24)            -0.0759              1.1                  !
! A9    A(1,2,25)             0.0904              1.3                  !
! A11   A(3,2,25)            -0.0239              0.4                  !
! A14   A(2,3,11)            -0.0877              1.3                  !
! A15   A(4,3,11)             0.0679              1.0                  !
! A17   A(3,4,10)            -0.025               0.4                  !
! A18   A(5,4,10)             0.0424              0.6                  !
! A19   A(4,5,6)              0.0454              0.7                  !
! A21   A(6,5,7)             -0.0346              0.5                  !
! A22   A(1,6,5)              0.0281              0.4                  !
! A23   A(5,7,8)             -0.0355              0.5                  !
! A24   A(5,7,26)             0.0273              0.4                  !
! A27   A(7,8,12)            -0.0463              0.7                  !
! A28   A(9,8,12)             0.0359              0.5                  !
! A29   A(8,9,10)             0.0218              0.3                  !
! A31   A(10,9,27)           -0.0384              0.6                  !
! A32   A(4,10,9)            -0.0282              0.4                  !
! A35   A(8,12,13)           -0.0316              0.5                  !
! A36   A(12,13,29)          -0.0225              0.3                  !
! A38   A(12,13,31)          -0.0223              0.3                  !
! A43   A(1,15,16)           -0.0775              1.1                  !
! A44   A(1,15,20)           -0.0553              0.8                  !
! A45   A(16,15,20)           0.1319              1.9                  !
! A46   A(15,16,17)          -0.1163              1.7                  !
! A48   A(17,16,32)           0.1184              1.7                  !
! A49   A(16,17,18)           0.1822              2.7                  !
! A50   A(16,17,22)          -0.0418              0.6                  !
! A51   A(18,17,22)          -0.1404              2.1                  !
! A52   A(17,18,19)          -0.2664              3.9                  !
! A53   A(17,18,34)           0.125               1.8                  !
! A54   A(19,18,34)           0.1414              2.1                  !
! A55   A(18,19,20)           0.1693              2.5                  !
! A56   A(18,19,21)          -0.1473              2.2                  !
! A57   A(20,19,21)          -0.022               0.3                  !
! A58   A(15,20,19)          -0.1006              1.5                  !
! A59   A(15,20,33)          -0.0555              0.8                  !
! A60   A(19,20,33)           0.1562              2.3                  !
! A61   A(19,21,36)          -0.0515              0.8                  !
! A62   A(17,22,35)           0.0632              0.9                  !
! D1    D(6,1,2,3)            0.0868              1.3                  !
! D2    D(6,1,2,24)           0.0516              0.8                  !
! D3    D(6,1,2,25)           0.0484              0.7                  !
! D4    D(15,1,2,3)           0.0355              0.5                  !
```

D7	D(23,1,2,3)	0.0481	0.7
D10	D(2,1,6,5)	-0.0593	0.9
D12	D(23,1,6,5)	-0.0783	1.2
D16	D(6,1,15,20)	-0.027	0.4
D17	D(23,1,15,16)	0.0578	0.9
D18	D(23,1,15,20)	0.0401	0.6
D21	D(24,2,3,4)	0.0679	1.0
D22	D(24,2,3,11)	0.0777	1.1
D23	D(25,2,3,4)	0.087	1.3
D24	D(25,2,3,11)	0.0968	1.4
D37	D(4,5,6,1)	0.0249	0.4
D38	D(7,5,6,1)	0.0216	0.3
D63	D(1,15,16,32)	-0.0243	0.4
D66	D(1,15,20,19)	0.0223	0.3

! Normal Mode 51 !

Name	Definition	Value	Relative Weight (%)
R2	R(1,6)	-0.1885	2.5
R3	R(1,15)	0.225	2.9
R5	R(2,3)	-0.2645	3.5
R8	R(3,4)	0.0291	0.4
R11	R(4,10)	0.0939	1.2
R13	R(5,7)	0.0287	0.4
R14	R(7,8)	0.0245	0.3
R17	R(8,12)	-0.0683	0.9
R18	R(9,10)	0.0952	1.2
R20	R(10,14)	0.1104	1.4
R21	R(12,13)	-0.1664	2.2
R26	R(15,16)	0.2105	2.7
R27	R(15,20)	0.2028	2.7
R28	R(16,17)	0.0541	0.7
R30	R(17,18)	0.067	0.9
R31	R(17,22)	-0.1064	1.4
R32	R(18,19)	0.091	1.2
R34	R(19,20)	0.1143	1.5
R35	R(19,21)	-0.0481	0.6
A1	A(2,1,6)	0.0298	0.4
A3	A(2,1,23)	-0.0694	0.9
A6	A(15,1,23)	0.045	0.6
A7	A(1,2,3)	0.0374	0.5
A8	A(1,2,24)	0.0632	0.8
A9	A(1,2,25)	-0.0775	1.0
A10	A(3,2,24)	-0.0751	1.0
A11	A(3,2,25)	0.0557	0.7
A13	A(2,3,4)	-0.0268	0.3
A14	A(2,3,11)	0.1123	1.5
A15	A(4,3,11)	-0.0839	1.1

!	A17	A(3,4,10)	0.0286	0.4	!
!	A18	A(5,4,10)	-0.0528	0.7	!
!	A19	A(4,5,6)	-0.0572	0.7	!
!	A21	A(6,5,7)	0.0414	0.5	!
!	A23	A(5,7,8)	0.037	0.5	!
!	A24	A(5,7,26)	-0.0283	0.4	!
!	A27	A(7,8,12)	0.0546	0.7	!
!	A28	A(9,8,12)	-0.0481	0.6	!
!	A29	A(8,9,10)	-0.0348	0.5	!
!	A31	A(10,9,27)	0.0486	0.6	!
!	A32	A(4,10,9)	0.0413	0.5	!
!	A35	A(8,12,13)	0.0401	0.5	!
!	A36	A(12,13,29)	0.0284	0.4	!
!	A38	A(12,13,31)	0.0287	0.4	!
!	A43	A(1,15,16)	-0.0761	1.0	!
!	A44	A(1,15,20)	-0.149	1.9	!
!	A45	A(16,15,20)	0.2258	2.9	!
!	A46	A(15,16,17)	-0.2124	2.8	!
!	A47	A(15,16,32)	0.1603	2.1	!
!	A48	A(17,16,32)	0.0521	0.7	!
!	A49	A(16,17,18)	0.161	2.1	!
!	A50	A(16,17,22)	-0.121	1.6	!
!	A51	A(18,17,22)	-0.0401	0.5	!
!	A52	A(17,18,19)	-0.0935	1.2	!
!	A53	A(17,18,34)	0.034	0.4	!
!	A54	A(19,18,34)	0.0595	0.8	!
!	A55	A(18,19,20)	0.1758	2.3	!
!	A56	A(18,19,21)	-0.0345	0.5	!
!	A57	A(20,19,21)	-0.1413	1.8	!
!	A58	A(15,20,19)	-0.2568	3.4	!
!	A59	A(15,20,33)	0.1811	2.4	!
!	A60	A(19,20,33)	0.0757	1.0	!
!	A61	A(19,21,36)	0.0942	1.2	!
!	D2	D(6,1,2,24)	-0.0515	0.7	!
!	D3	D(6,1,2,25)	-0.0662	0.9	!
!	D4	D(15,1,2,3)	-0.0334	0.4	!
!	D5	D(15,1,2,24)	-0.062	0.8	!
!	D6	D(15,1,2,25)	-0.0768	1.0	!
!	D7	D(23,1,2,3)	-0.0331	0.4	!
!	D8	D(23,1,2,24)	-0.0617	0.8	!
!	D9	D(23,1,2,25)	-0.0764	1.0	!
!	D12	D(23,1,6,5)	0.0618	0.8	!
!	D14	D(2,1,15,20)	0.0407	0.5	!
!	D17	D(23,1,15,16)	-0.047	0.6	!
!	D19	D(1,2,3,4)	-0.0446	0.6	!
!	D21	D(24,2,3,4)	-0.0972	1.3	!
!	D22	D(24,2,3,11)	-0.0397	0.5	!
!	D23	D(25,2,3,4)	-0.0795	1.0	!
!	D25	D(2,3,4,5)	0.0453	0.6	!
!	D28	D(11,3,4,10)	-0.0577	0.8	!

```
! D29   D(3,4,5,6)             -0.0416                 0.5                   !
! D30   D(3,4,5,7)             -0.024                  0.3                   !
! D33   D(3,4,10,9)             0.0281                 0.4                   !
! D34   D(3,4,10,14)            0.0297                 0.4                   !
--------------------------------------------------------------------------------
                          ----------------------------
                          ! Normal Mode    52        !
--------------------------                            --------------------------
! Name  Definition              Value          Relative Weight (%)           !
--------------------------------------------------------------------------------
! R1    R(1,2)                  0.0538                 0.7                   !
! R2    R(1,6)                  0.0676                 0.8                   !
! R3    R(1,15)                 0.096                  1.2                   !
! R5    R(2,3)                  0.1342                 1.7                   !
! R11   R(4,10)                -0.0324                 0.4                   !
! R13   R(5,7)                 -0.0344                 0.4                   !
! R14   R(7,8)                 -0.0426                 0.5                   !
! R16   R(8,9)                 -0.0366                 0.5                   !
! R18   R(9,10)                -0.0378                 0.5                   !
! R21   R(12,13)                0.0706                 0.9                   !
! R26   R(15,16)                0.0599                 0.7                   !
! R27   R(15,20)                0.1036                 1.3                   !
! R28   R(16,17)                0.1293                 1.6                   !
! R30   R(17,18)                0.0952                 1.2                   !
! R31   R(17,22)                0.1441                 1.8                   !
! R32   R(18,19)               -0.1259                 1.6                   !
! R34   R(19,20)               -0.1331                 1.7                   !
! R35   R(19,21)               -0.2125                 2.6                   !
! A2    A(2,1,15)              -0.0877                 1.1                   !
! A3    A(2,1,23)               0.0495                 0.6                   !
! A4    A(6,1,15)              -0.0612                 0.8                   !
! A6    A(15,1,23)              0.1068                 1.3                   !
! A7    A(1,2,3)               -0.1082                 1.3                   !
! A10   A(3,2,24)               0.1842                 2.3                   !
! A11   A(3,2,25)              -0.0894                 1.1                   !
! A14   A(2,3,11)              -0.061                  0.8                   !
! A15   A(4,3,11)               0.0525                 0.7                   !
! A18   A(5,4,10)               0.0378                 0.5                   !
! A19   A(4,5,6)                0.0284                 0.4                   !
! A20   A(4,5,7)               -0.0469                 0.6                   !
! A22   A(1,6,5)               -0.0462                 0.6                   !
! A23   A(5,7,8)                0.0357                 0.4                   !
! A25   A(8,7,26)              -0.0383                 0.5                   !
! A29   A(8,9,10)               0.0349                 0.4                   !
! A30   A(8,9,27)              -0.0261                 0.3                   !
! A32   A(4,10,9)              -0.0386                 0.5                   !
! A34   A(9,10,14)              0.0254                 0.3                   !
! A43   A(1,15,16)             -0.1228                 1.5                   !
! A44   A(1,15,20)              0.0889                 1.1                   !
! A45   A(16,15,20)             0.0366                 0.5                   !
```

!	A46	A(15,16,17)	-0.1529	1.9	!
!	A47	A(15,16,32)	0.0392	0.5	!
!	A48	A(17,16,32)	0.1138	1.4	!
!	A49	A(16,17,18)	0.0441	0.5	!
!	A50	A(16,17,22)	-0.0884	1.1	!
!	A51	A(18,17,22)	0.0443	0.5	!
!	A52	A(17,18,19)	0.0267	0.3	!
!	A53	A(17,18,34)	0.1416	1.8	!
!	A54	A(19,18,34)	-0.1683	2.1	!
!	A58	A(15,20,19)	0.047	0.6	!
!	A59	A(15,20,33)	0.1265	1.6	!
!	A60	A(19,20,33)	-0.1727	2.1	!
!	A61	A(19,21,36)	0.041	0.5	!
!	A62	A(17,22,35)	-0.1051	1.3	!
!	D1	D(6,1,2,3)	-0.1472	1.8	!
!	D2	D(6,1,2,24)	0.0285	0.4	!
!	D3	D(6,1,2,25)	0.0564	0.7	!
!	D5	D(15,1,2,24)	0.1817	2.3	!
!	D6	D(15,1,2,25)	0.2096	2.6	!
!	D7	D(23,1,2,3)	-0.1073	1.3	!
!	D8	D(23,1,2,24)	0.0685	0.8	!
!	D9	D(23,1,2,25)	0.0963	1.2	!
!	D10	D(2,1,6,5)	0.1329	1.6	!
!	D11	D(15,1,6,5)	-0.03	0.4	!
!	D12	D(23,1,6,5)	0.0747	0.9	!
!	D13	D(2,1,15,16)	-0.1103	1.4	!
!	D14	D(2,1,15,20)	-0.0475	0.6	!
!	D16	D(6,1,15,20)	0.0825	1.0	!
!	D17	D(23,1,15,16)	-0.0291	0.4	!
!	D18	D(23,1,15,20)	0.0337	0.4	!
!	D19	D(1,2,3,4)	0.1498	1.9	!
!	D21	D(24,2,3,4)	0.0692	0.9	!
!	D22	D(24,2,3,11)	-0.0944	1.2	!
!	D24	D(25,2,3,11)	-0.1716	2.1	!
!	D25	D(2,3,4,5)	-0.1301	1.6	!
!	D26	D(2,3,4,10)	-0.0407	0.5	!
!	D27	D(11,3,4,5)	0.0452	0.6	!
!	D28	D(11,3,4,10)	0.1346	1.7	!
!	D29	D(3,4,5,6)	0.0846	1.0	!
!	D30	D(3,4,5,7)	0.0464	0.6	!
!	D32	D(10,4,5,7)	-0.0343	0.4	!
!	D33	D(3,4,10,9)	-0.0556	0.7	!
!	D34	D(3,4,10,14)	-0.0708	0.9	!
!	D35	D(5,4,10,9)	0.0295	0.4	!
!	D37	D(4,5,6,1)	-0.0743	0.9	!
!	D38	D(7,5,6,1)	-0.0381	0.5	!
!	D62	D(1,15,16,17)	0.0295	0.4	!
!	D63	D(1,15,16,32)	0.06	0.7	!
!	D64	D(20,15,16,17)	-0.0346	0.4	!
!	D67	D(1,15,20,33)	-0.0642	0.8	!

```
! D68   D(16,15,20,19)          0.0357                 0.4                    !
! D87   D(21,19,20,33)          0.0263                 0.3                    !
--------------------------------------------------------------------------------
                          ----------------------------
                          ! Normal Mode    53        !
--------------------------                            --------------------------
! Name  Definition              Value          Relative Weight (%)            !
--------------------------------------------------------------------------------
! R1    R(1,2)                 -0.1435                 1.7                    !
! R2    R(1,6)                 -0.0863                 1.0                    !
! R3    R(1,15)                 0.0598                 0.7                    !
! R5    R(2,3)                  0.1309                 1.6                    !
! R8    R(3,4)                  0.0949                 1.1                    !
! R10   R(4,5)                 -0.0505                 0.6                    !
! R12   R(5,6)                 -0.0988                 1.2                    !
! R13   R(5,7)                 -0.0699                 0.8                    !
! R14   R(7,8)                 -0.1709                 2.0                    !
! R16   R(8,9)                 -0.1667                 2.0                    !
! R18   R(9,10)                -0.057                  0.7                    !
! R20   R(10,14)                0.078                  0.9                    !
! R21   R(12,13)                0.231                  2.8                    !
! R26   R(15,16)                0.0511                 0.6                    !
! R28   R(16,17)               -0.0403                 0.5                    !
! R30   R(17,18)               -0.0381                 0.5                    !
! R31   R(17,22)               -0.0613                 0.7                    !
! A1    A(2,1,6)                0.103                  1.2                    !
! A3    A(2,1,23)              -0.0418                 0.5                    !
! A5    A(6,1,23)              -0.0555                 0.7                    !
! A6    A(15,1,23)             -0.0312                 0.4                    !
! A8    A(1,2,24)              -0.1093                 1.3                    !
! A10   A(3,2,24)              -0.1273                 1.5                    !
! A11   A(3,2,25)               0.2475                 3.0                    !
! A14   A(2,3,11)              -0.0556                 0.7                    !
! A15   A(4,3,11)               0.0846                 1.0                    !
! A17   A(3,4,10)              -0.0889                 1.1                    !
! A18   A(5,4,10)               0.0921                 1.1                    !
! A20   A(4,5,7)               -0.1509                 1.8                    !
! A21   A(6,5,7)                0.1342                 1.6                    !
! A22   A(1,6,5)                0.0333                 0.4                    !
! A23   A(5,7,8)                0.1393                 1.7                    !
! A24   A(5,7,26)               0.1232                 1.5                    !
! A25   A(8,7,26)              -0.2625                 3.1                    !
! A26   A(7,8,9)               -0.0548                 0.7                    !
! A28   A(9,8,12)               0.0512                 0.6                    !
! A29   A(8,9,10)               0.0817                 1.0                    !
! A30   A(8,9,27)              -0.135                  1.6                    !
! A31   A(10,9,27)              0.0533                 0.6                    !
! A32   A(4,10,9)              -0.1068                 1.3                    !
! A33   A(4,10,14)              0.0299                 0.4                    !
! A34   A(9,10,14)              0.0771                 0.9                    !
```

A35	A(8,12,13)	-0.039	0.5
A37	A(12,13,30)	-0.0532	0.6
A40	A(29,13,31)	0.0288	0.3
A43	A(1,15,16)	0.0474	0.6
A44	A(1,15,20)	-0.0469	0.6
A47	A(15,16,32)	0.0473	0.6
A48	A(17,16,32)	-0.0579	0.7
A52	A(17,18,19)	0.0268	0.3
A53	A(17,18,34)	-0.0409	0.5
A58	A(15,20,19)	-0.0289	0.3
A59	A(15,20,33)	0.0287	0.3
D1	D(6,1,2,3)	0.1214	1.4
D2	D(6,1,2,24)	-0.1118	1.3
D3	D(6,1,2,25)	-0.1884	2.2
D5	D(15,1,2,24)	-0.2101	2.5
D6	D(15,1,2,25)	-0.2867	3.4
D7	D(23,1,2,3)	0.0894	1.1
D8	D(23,1,2,24)	-0.1438	1.7
D9	D(23,1,2,25)	-0.2204	2.6
D10	D(2,1,6,5)	-0.0497	0.6
D11	D(15,1,6,5)	0.0338	0.4
D13	D(2,1,15,16)	0.1062	1.3
D14	D(2,1,15,20)	0.0684	0.8
D15	D(6,1,15,16)	-0.0401	0.5
D16	D(6,1,15,20)	-0.0779	0.9
D17	D(23,1,15,16)	0.0339	0.4
D19	D(1,2,3,4)	-0.1542	1.8
D21	D(24,2,3,4)	0.0672	0.8
D22	D(24,2,3,11)	0.202	2.4
D24	D(25,2,3,11)	0.1407	1.7
D25	D(2,3,4,5)	0.103	1.2
D26	D(2,3,4,10)	0.0534	0.6
D27	D(11,3,4,5)	-0.0367	0.4
D28	D(11,3,4,10)	-0.0863	1.0
D29	D(3,4,5,6)	-0.0599	0.7
D32	D(10,4,5,7)	0.0283	0.3
D34	D(3,4,10,14)	0.0423	0.5
D37	D(4,5,6,1)	0.0711	0.8
D38	D(7,5,6,1)	0.0408	0.5
D62	D(1,15,16,17)	-0.0303	0.4
D63	D(1,15,16,32)	-0.0399	0.5
D66	D(1,15,20,19)	0.027	0.3
D67	D(1,15,20,33)	0.0453	0.5

! Normal Mode 54 !

Name	Definition	Value	Relative Weight (%)
R1	R(1,2)	-0.5073	5.5

```
! R2    R(1,6)                0.3018                3.3                  !
! R3    R(1,15)               0.03                  0.3                  !
! R8    R(3,4)               -0.0463                0.5                  !
! R10   R(4,5)                0.0594                0.6                  !
! R12   R(5,6)                0.0822                0.9                  !
! R14   R(7,8)                0.0338                0.4                  !
! R16   R(8,9)                0.0642                0.7                  !
! R18   R(9,10)              -0.03                  0.3                  !
! R20   R(10,14)             -0.1079                1.2                  !
! R26   R(15,16)              0.0288                0.3                  !
! R27   R(15,20)              0.0776                0.8                  !
! R32   R(18,19)             -0.031                 0.3                  !
! R35   R(19,21)             -0.035                 0.4                  !
! A1    A(2,1,6)              0.0912                1.0                  !
! A2    A(2,1,15)             0.1747                1.9                  !
! A3    A(2,1,23)            -0.112                 1.2                  !
! A4    A(6,1,15)            -0.1653                1.8                  !
! A5    A(6,1,23)            -0.036                 0.4                  !
! A6    A(15,1,23)            0.0369                0.4                  !
! A7    A(1,2,3)              0.1303                1.4                  !
! A8    A(1,2,24)            -0.0917                1.0                  !
! A9    A(1,2,25)            -0.1033                1.1                  !
! A10   A(3,2,24)            -0.1169                1.3                  !
! A11   A(3,2,25)             0.1909                2.1                  !
! A14   A(2,3,11)             0.0545                0.6                  !
! A15   A(4,3,11)            -0.0421                0.5                  !
! A17   A(3,4,10)             0.0502                0.5                  !
! A18   A(5,4,10)            -0.0353                0.4                  !
! A19   A(4,5,6)              0.0366                0.4                  !
! A20   A(4,5,7)              0.0575                0.6                  !
! A21   A(6,5,7)             -0.0944                1.0                  !
! A22   A(1,6,5)             -0.135                 1.5                  !
! A23   A(5,7,8)             -0.0638                0.7                  !
! A24   A(5,7,26)            -0.0596                0.6                  !
! A25   A(8,7,26)             0.1232                1.3                  !
! A30   A(8,9,27)             0.0506                0.6                  !
! A31   A(10,9,27)           -0.0509                0.6                  !
! A42   A(10,14,28)           0.0594                0.6                  !
! A47   A(15,16,32)           0.0312                0.3                  !
! A54   A(19,18,34)          -0.0355                0.4                  !
! A59   A(15,20,33)           0.0935                1.0                  !
! A60   A(19,20,33)          -0.0921                1.0                  !
! A61   A(19,21,36)           0.0375                0.4                  !
! A62   A(17,22,35)          -0.0309                0.3                  !
! D1    D(6,1,2,3)            0.0575                0.6                  !
! D2    D(6,1,2,24)          -0.0661                0.7                  !
! D3    D(6,1,2,25)          -0.2048                2.2                  !
! D4    D(15,1,2,3)           0.0865                0.9                  !
! D5    D(15,1,2,24)         -0.0371                0.4                  !
! D6    D(15,1,2,25)         -0.1757                1.9                  !
```

D8	D(23,1,2,24)	-0.1216	1.3
D9	D(23,1,2,25)	-0.2603	2.8
D10	D(2,1,6,5)	-0.0797	0.9
D11	D(15,1,6,5)	0.0877	1.0
D13	D(2,1,15,16)	0.0909	1.0
D14	D(2,1,15,20)	-0.1214	1.3
D16	D(6,1,15,20)	-0.2347	2.6
D17	D(23,1,15,16)	0.0919	1.0
D18	D(23,1,15,20)	-0.1204	1.3
D19	D(1,2,3,4)	-0.1009	1.1
D20	D(1,2,3,11)	0.0759	0.8
D22	D(24,2,3,11)	0.1852	2.0
D24	D(25,2,3,11)	0.1611	1.8
D25	D(2,3,4,5)	0.1412	1.5
D26	D(2,3,4,10)	0.0781	0.8
D27	D(11,3,4,5)	-0.0476	0.5
D28	D(11,3,4,10)	-0.1107	1.2
D30	D(3,4,5,7)	-0.0472	0.5
D31	D(10,4,5,6)	0.03	0.3
D33	D(3,4,10,9)	0.0456	0.5
D34	D(3,4,10,14)	0.0576	0.6
D37	D(4,5,6,1)	0.0851	0.9
D38	D(7,5,6,1)	0.1047	1.1
D42	D(6,5,7,26)	-0.0322	0.4
D62	D(1,15,16,17)	-0.1446	1.6
D63	D(1,15,16,32)	-0.2143	2.3
D64	D(20,15,16,17)	0.0658	0.7
D66	D(1,15,20,19)	0.1489	1.6
D67	D(1,15,20,33)	0.2427	2.6
D68	D(16,15,20,19)	-0.0671	0.7
D70	D(15,16,17,18)	-0.0307	0.3
D71	D(15,16,17,22)	-0.0321	0.3
D72	D(32,16,17,18)	0.0386	0.4
D73	D(32,16,17,22)	0.0373	0.4
D84	D(18,19,20,15)	0.0342	0.4
D85	D(18,19,20,33)	-0.0557	0.6
D86	D(21,19,20,15)	0.0326	0.4
D87	D(21,19,20,33)	-0.0573	0.6

! Normal Mode 55 !

Name	Definition	Value	Relative Weight (%)
R1	R(1,2)	-0.056	0.7
R2	R(1,6)	0.1633	2.1
R3	R(1,15)	-0.0246	0.3
R5	R(2,3)	-0.2573	3.4
R8	R(3,4)	-0.0321	0.4
R10	R(4,5)	0.0705	0.9

```
! R11   R(4,10)          0.0982          1.3          !
! R12   R(5,6)           0.0369          0.5          !
! R13   R(5,7)           0.0402          0.5          !
! R14   R(7,8)          -0.1187          1.6          !
! R16   R(8,9)          -0.091           1.2          !
! R17   R(8,12)         -0.0281          0.4          !
! R18   R(9,10)          0.0734          1.0          !
! R20   R(10,14)         0.026           0.3          !
! R21   R(12,13)         0.3122          4.1          !
! R26   R(15,16)        -0.0582          0.8          !
! R27   R(15,20)        -0.0322          0.4          !
! R30   R(17,18)         0.0278          0.4          !
! R31   R(17,22)         0.0342          0.4          !
! A1    A(2,1,6)        -0.1014          1.3          !
! A2    A(2,1,15)        0.0849          1.1          !
! A4    A(6,1,15)       -0.0436          0.6          !
! A5    A(6,1,23)        0.0595          0.8          !
! A7    A(1,2,3)         0.1115          1.5          !
! A8    A(1,2,24)        0.1173          1.5          !
! A9    A(1,2,25)       -0.0761          1.0          !
! A10   A(3,2,24)        0.0382          0.5          !
! A11   A(3,2,25)       -0.1786          2.3          !
! A13   A(2,3,4)        -0.0239          0.3          !
! A14   A(2,3,11)        0.1559          2.0          !
! A15   A(4,3,11)       -0.1325          1.7          !
! A16   A(3,4,5)         0.0308          0.4          !
! A17   A(3,4,10)        0.1             1.3          !
! A18   A(5,4,10)       -0.131           1.7          !
! A19   A(4,5,6)        -0.0654          0.9          !
! A20   A(4,5,7)         0.0707          0.9          !
! A23   A(5,7,8)        -0.034           0.4          !
! A24   A(5,7,26)        0.2404          3.2          !
! A25   A(8,7,26)       -0.2063          2.7          !
! A26   A(7,8,9)         0.084           1.1          !
! A27   A(7,8,12)       -0.0764          1.0          !
! A29   A(8,9,10)       -0.0381          0.5          !
! A30   A(8,9,27)       -0.1023          1.3          !
! A31   A(10,9,27)       0.1403          1.8          !
! A32   A(4,10,9)        0.0482          0.6          !
! A33   A(4,10,14)      -0.0681          0.9          !
! A35   A(8,12,13)      -0.0458          0.6          !
! A37   A(12,13,30)     -0.085           1.1          !
! A40   A(29,13,31)      0.0476          0.6          !
! A42   A(10,14,28)      0.0499          0.7          !
! A43   A(1,15,16)      -0.0503          0.7          !
! A44   A(1,15,20)       0.0339          0.4          !
! A47   A(15,16,32)     -0.0579          0.8          !
! A48   A(17,16,32)      0.0628          0.8          !
! A59   A(15,20,33)     -0.0588          0.8          !
! A60   A(19,20,33)      0.044           0.6          !
```

```
! D1    D(6,1,2,3)             -0.077                  1.0                  !
! D2    D(6,1,2,24)             0.1176                 1.5                  !
! D3    D(6,1,2,25)             0.1269                 1.7                  !
! D5    D(15,1,2,24)            0.1889                 2.5                  !
! D6    D(15,1,2,25)            0.1981                 2.6                  !
! D7    D(23,1,2,3)            -0.0654                 0.9                  !
! D8    D(23,1,2,24)            0.1292                 1.7                  !
! D9    D(23,1,2,25)            0.1384                 1.8                  !
! D12   D(23,1,6,5)             0.0278                 0.4                  !
! D13   D(2,1,15,16)           -0.0659                 0.9                  !
! D14   D(2,1,15,20)           -0.1132                 1.5                  !
! D15   D(6,1,15,16)            0.0382                 0.5                  !
! D18   D(23,1,15,20)          -0.058                  0.8                  !
! D19   D(1,2,3,4)              0.0953                 1.3                  !
! D20   D(1,2,3,11)             0.0709                 0.9                  !
! D21   D(24,2,3,4)            -0.144                  1.9                  !
! D22   D(24,2,3,11)           -0.1684                 2.2                  !
! D23   D(25,2,3,4)            -0.0495                 0.7                  !
! D24   D(25,2,3,11)           -0.0739                 1.0                  !
! D37   D(4,5,6,1)             -0.0334                 0.4                  !
! D59   D(8,12,13,29)           0.0284                 0.4                  !
! D61   D(8,12,13,31)          -0.0314                 0.4                  !
! D62   D(1,15,16,17)          -0.0242                 0.3                  !
! D63   D(1,15,16,32)          -0.0448                 0.6                  !
! D66   D(1,15,20,19)           0.0287                 0.4                  !
! D67   D(1,15,20,33)           0.046                  0.6                  !
--------------------------------------------------------------------------------
                          ----------------------------
                          ! Normal Mode    56        !
--------------------------                          --------------------------
! Name  Definition              Value          Relative Weight (%)          !
--------------------------------------------------------------------------------
! R1    R(1,2)                  0.1629                 2.1                  !
! R2    R(1,6)                 -0.331                  4.2                  !
! R3    R(1,15)                 0.0319                 0.4                  !
! R5    R(2,3)                 -0.0866                 1.1                  !
! R8    R(3,4)                  0.0337                 0.4                  !
! R9    R(3,11)                -0.0297                 0.4                  !
! R10   R(4,5)                  0.137                  1.7                  !
! R11   R(4,10)                -0.0704                 0.9                  !
! R12   R(5,6)                  0.3166                 4.0                  !
! R13   R(5,7)                  0.1056                 1.3                  !
! R14   R(7,8)                 -0.0269                 0.3                  !
! R16   R(8,9)                 -0.0539                 0.7                  !
! R17   R(8,12)                 0.0585                 0.7                  !
! R18   R(9,10)                -0.15                   1.9                  !
! R20   R(10,14)               -0.3147                 4.0                  !
! R21   R(12,13)                0.0252                 0.3                  !
! R26   R(15,16)                0.0318                 0.4                  !
! R27   R(15,20)                0.0478                 0.6                  !
```

```
! R30   R(17,18)             -0.026                  0.3                   !
! A1    A(2,1,6)              0.083                  1.1                   !
! A2    A(2,1,15)            -0.0826                 1.0                   !
! A3    A(2,1,23)            -0.0794                 1.0                   !
! A5    A(6,1,23)             0.0763                 1.0                   !
! A7    A(1,2,3)             -0.0515                 0.7                   !
! A8    A(1,2,24)             0.0546                 0.7                   !
! A9    A(1,2,25)             0.0477                 0.6                   !
! A10   A(3,2,24)            -0.1337                 1.7                   !
! A11   A(3,2,25)             0.0656                 0.8                   !
! A13   A(2,3,4)              0.1335                 1.7                   !
! A15   A(4,3,11)            -0.1504                 1.9                   !
! A16   A(3,4,5)             -0.1363                 1.7                   !
! A17   A(3,4,10)             0.1415                 1.8                   !
! A20   A(4,5,7)              0.0265                 0.3                   !
! A21   A(6,5,7)             -0.0407                 0.5                   !
! A22   A(1,6,5)             -0.0423                 0.5                   !
! A23   A(5,7,8)             -0.108                  1.4                   !
! A24   A(5,7,26)             0.2846                 3.6                   !
! A25   A(8,7,26)            -0.1766                 2.2                   !
! A27   A(7,8,12)            -0.0813                 1.0                   !
! A28   A(9,8,12)             0.076                  1.0                   !
! A29   A(8,9,10)             0.1446                 1.8                   !
! A31   A(10,9,27)           -0.1253                 1.6                   !
! A32   A(4,10,9)            -0.0614                 0.8                   !
! A33   A(4,10,14)           -0.0592                 0.7                   !
! A34   A(9,10,14)            0.1206                 1.5                   !
! A36   A(12,13,29)          -0.034                  0.4                   !
! A37   A(12,13,30)           0.0731                 0.9                   !
! A38   A(12,13,31)          -0.0315                 0.4                   !
! A42   A(10,14,28)           0.2831                 3.6                   !
! A43   A(1,15,16)            0.0459                 0.6                   !
! A44   A(1,15,20)           -0.0365                 0.5                   !
! A47   A(15,16,32)           0.0385                 0.5                   !
! A48   A(17,16,32)          -0.0357                 0.5                   !
! A59   A(15,20,33)           0.0686                 0.9                   !
! A60   A(19,20,33)          -0.0504                 0.6                   !
! A62   A(17,22,35)          -0.0285                 0.4                   !
! D2    D(6,1,2,24)          -0.1868                 2.4                   !
! D3    D(6,1,2,25)          -0.1048                 1.3                   !
! D4    D(15,1,2,3)          -0.0308                 0.4                   !
! D5    D(15,1,2,24)         -0.194                  2.5                   !
! D6    D(15,1,2,25)         -0.112                  1.4                   !
! D7    D(23,1,2,3)           0.0702                 0.9                   !
! D8    D(23,1,2,24)         -0.0931                 1.2                   !
! D11   D(15,1,6,5)          -0.0287                 0.4                   !
! D12   D(23,1,6,5)           0.025                  0.3                   !
! D13   D(2,1,15,16)          0.038                  0.5                   !
! D14   D(2,1,15,20)          0.1056                 1.3                   !
! D16   D(6,1,15,20)          0.0525                 0.7                   !
```

```
! D17   D(23,1,15,16)         -0.1125              1.4                  !
! D18   D(23,1,15,20)         -0.0449              0.6                  !
! D19   D(1,2,3,4)            -0.0605              0.8                  !
! D20   D(1,2,3,11)           -0.0434              0.5                  !
! D24   D(25,2,3,11)           0.0277              0.4                  !
! D28   D(11,3,4,10)          -0.0337              0.4                  !
! D29   D(3,4,5,6)            -0.0328              0.4                  !
! D59   D(8,12,13,29)         -0.0373              0.5                  !
! D61   D(8,12,13,31)          0.0384              0.5                  !
! D62   D(1,15,16,17)          0.0528              0.7                  !
! D63   D(1,15,16,32)          0.0732              0.9                  !
! D66   D(1,15,20,19)         -0.0581              0.7                  !
! D67   D(1,15,20,33)         -0.0751              1.0                  !
--------------------------------------------------------------------------------
                          ----------------------------
                          ! Normal Mode    57        !
--------------------------                            --------------------------
! Name  Definition              Value          Relative Weight (%)           !
--------------------------------------------------------------------------------
! R2    R(1,6)                 0.0186              0.4                  !
! R3    R(1,15)               -0.1863              3.6                  !
! R5    R(2,3)                 0.0225              0.4                  !
! R8    R(3,4)                -0.0638              1.2                  !
! R10   R(4,5)                 0.0253              0.5                  !
! R12   R(5,6)                -0.038               0.7                  !
! R13   R(5,7)                 0.0362              0.7                  !
! R18   R(9,10)                0.0249              0.5                  !
! R26   R(15,16)               0.116               2.2                  !
! R27   R(15,20)               0.0317              0.6                  !
! R28   R(16,17)              -0.0178              0.3                  !
! R30   R(17,18)               0.0268              0.5                  !
! R31   R(17,22)              -0.0696              1.3                  !
! R32   R(18,19)              -0.0659              1.3                  !
! R34   R(19,20)              -0.0233              0.4                  !
! R35   R(19,21)               0.1761              3.4                  !
! A2    A(2,1,15)              0.0299              0.6                  !
! A3    A(2,1,23)              0.0674              1.3                  !
! A4    A(6,1,15)              0.028               0.5                  !
! A6    A(15,1,23)            -0.0984              1.9                  !
! A7    A(1,2,3)               0.0167              0.3                  !
! A8    A(1,2,24)             -0.0746              1.4                  !
! A9    A(1,2,25)              0.0936              1.8                  !
! A10   A(3,2,24)              0.0699              1.3                  !
! A11   A(3,2,25)             -0.1061              2.0                  !
! A18   A(5,4,10)             -0.0223              0.4                  !
! A22   A(1,6,5)               0.0241              0.5                  !
! A24   A(5,7,26)              0.0787              1.5                  !
! A25   A(8,7,26)             -0.0718              1.4                  !
! A26   A(7,8,9)               0.0172              0.3                  !
! A29   A(8,9,10)             -0.0166              0.3                  !
```

A31	A(10,9,27)	0.0262	0.5
A32	A(4,10,9)	0.0227	0.4
A36	A(12,13,29)	0.0261	0.5
A42	A(10,14,28)	-0.0184	0.4
A43	A(1,15,16)	0.0377	0.7
A44	A(1,15,20)	0.0341	0.7
A45	A(16,15,20)	-0.0727	1.4
A46	A(15,16,17)	0.0356	0.7
A47	A(15,16,32)	0.1548	3.0
A48	A(17,16,32)	-0.1904	3.6
A53	A(17,18,34)	0.1809	3.5
A54	A(19,18,34)	-0.1686	3.2
A55	A(18,19,20)	0.058	1.1
A56	A(18,19,21)	-0.0249	0.5
A57	A(20,19,21)	-0.0332	0.6
A59	A(15,20,33)	0.2785	5.3
A60	A(19,20,33)	-0.2843	5.4
A61	A(19,21,36)	-0.0404	0.8
A62	A(17,22,35)	0.1227	2.3
D1	D(6,1,2,3)	0.0384	0.7
D2	D(6,1,2,24)	0.0871	1.7
D3	D(6,1,2,25)	0.0974	1.9
D5	D(15,1,2,24)	0.0414	0.8
D6	D(15,1,2,25)	0.0517	1.0
D7	D(23,1,2,3)	0.0499	1.0
D8	D(23,1,2,24)	0.0986	1.9
D9	D(23,1,2,25)	0.109	2.1
D10	D(2,1,6,5)	-0.0437	0.8
D12	D(23,1,6,5)	-0.1073	2.0
D13	D(2,1,15,16)	0.0174	0.3
D16	D(6,1,15,20)	-0.0242	0.5
D17	D(23,1,15,16)	0.0529	1.0
D18	D(23,1,15,20)	0.0328	0.6
D20	D(1,2,3,11)	-0.0179	0.3
D21	D(24,2,3,4)	0.0405	0.8
D22	D(24,2,3,11)	0.0187	0.4
D23	D(25,2,3,4)	0.0599	1.1
D24	D(25,2,3,11)	0.0381	0.7
D28	D(11,3,4,10)	0.0191	0.4
D63	D(1,15,16,32)	-0.0193	0.4
D67	D(1,15,20,33)	0.029	0.6

! Normal Mode 58 !

Name	Definition	Value	Relative Weight (%)
R3	R(1,15)	0.0075	0.4
R35	R(19,21)	-0.0081	0.4
A36	A(12,13,29)	0.3959	18.6

A38	A(12,13,31)	-0.4012	18.9
A39	A(29,13,30)	0.114	5.4
A41	A(30,13,31)	-0.1113	5.2
A48	A(17,16,32)	0.0075	0.4
A53	A(17,18,34)	-0.01	0.5
A54	A(19,18,34)	0.0093	0.4
A59	A(15,20,33)	-0.0117	0.5
A60	A(19,20,33)	0.0118	0.6
A62	A(17,22,35)	-0.0071	0.3
D40	D(4,5,7,26)	0.007	0.3
D44	D(5,7,8,12)	0.0269	1.3
D46	D(26,7,8,12)	0.0191	0.9
D49	D(12,8,9,10)	-0.0285	1.3
D50	D(12,8,9,27)	-0.0228	1.1
D51	D(7,8,12,13)	0.0877	4.1
D52	D(9,8,12,13)	0.114	5.4
D59	D(8,12,13,29)	-0.1877	8.8
D60	D(8,12,13,30)	0.1631	7.7
D61	D(8,12,13,31)	-0.1801	8.5

! Normal Mode 59 !

Name	Definition	Value	Relative Weight (%)
R3	R(1,15)	0.1045	2.7
R5	R(2,3)	-0.0189	0.5
R8	R(3,4)	0.0342	0.9
R13	R(5,7)	-0.0122	0.3
R17	R(8,12)	0.014	0.4
R18	R(9,10)	-0.0216	0.6
R20	R(10,14)	-0.0153	0.4
R21	R(12,13)	-0.0137	0.4
R26	R(15,16)	-0.0288	0.7
R27	R(15,20)	-0.0666	1.7
R28	R(16,17)	-0.0205	0.5
R30	R(17,18)	0.131	3.4
R31	R(17,22)	-0.185	4.7
R32	R(18,19)	-0.0548	1.4
R34	R(19,20)	0.0243	0.6
R35	R(19,21)	0.0414	1.1
A2	A(2,1,15)	-0.0146	0.4
A3	A(2,1,23)	-0.0308	0.8
A4	A(6,1,15)	-0.0233	0.6
A6	A(15,1,23)	0.059	1.5
A8	A(1,2,24)	0.036	0.9
A9	A(1,2,25)	-0.0556	1.4
A10	A(3,2,24)	-0.028	0.7
A11	A(3,2,25)	0.0572	1.5
A18	A(5,4,10)	0.0142	0.4

A22	A(1,6,5)	-0.0163	0.4
A24	A(5,7,26)	-0.0208	0.5
A25	A(8,7,26)	0.0176	0.5
A32	A(4,10,9)	-0.0139	0.4
A37	A(12,13,30)	0.0225	0.6
A42	A(10,14,28)	0.0268	0.7
A43	A(1,15,16)	-0.0247	0.6
A45	A(16,15,20)	0.0311	0.8
A46	A(15,16,17)	0.0368	0.9
A47	A(15,16,32)	-0.1372	3.5
A48	A(17,16,32)	0.1004	2.6
A49	A(16,17,18)	-0.0692	1.8
A50	A(16,17,22)	0.0668	1.7
A53	A(17,18,34)	0.3245	8.3
A54	A(19,18,34)	-0.3281	8.4
A55	A(18,19,20)	0.0228	0.6
A57	A(20,19,21)	-0.0172	0.4
A58	A(15,20,19)	-0.025	0.6
A59	A(15,20,33)	-0.0943	2.4
A60	A(19,20,33)	0.1194	3.1
A61	A(19,21,36)	0.122	3.1
A62	A(17,22,35)	0.2714	6.9
D1	D(6,1,2,3)	-0.0233	0.6
D2	D(6,1,2,24)	-0.0402	1.0
D3	D(6,1,2,25)	-0.0517	1.3
D6	D(15,1,2,25)	-0.0165	0.4
D7	D(23,1,2,3)	-0.0317	0.8
D8	D(23,1,2,24)	-0.0486	1.2
D9	D(23,1,2,25)	-0.0601	1.5
D10	D(2,1,6,5)	0.0239	0.6
D12	D(23,1,6,5)	0.0545	1.4
D13	D(2,1,15,16)	-0.0134	0.3
D17	D(23,1,15,16)	-0.0202	0.5
D18	D(23,1,15,20)	-0.0134	0.3
D21	D(24,2,3,4)	-0.019	0.5
D23	D(25,2,3,4)	-0.0345	0.9
D24	D(25,2,3,11)	-0.0262	0.7

! Normal Mode 60 !

Name	Definition	Value	Relative Weight (%)
R1	R(1,2)	0.0494	0.8
R2	R(1,6)	-0.0785	1.3
R3	R(1,15)	-0.0253	0.4
R8	R(3,4)	0.0808	1.3
R10	R(4,5)	-0.0398	0.6
R11	R(4,10)	-0.0856	1.4
R12	R(5,6)	0.2063	3.3

!	R13	R(5,7)	-0.0823	1.3	!
!	R14	R(7,8)	0.0381	0.6	!
!	R16	R(8,9)	0.0226	0.4	!
!	R17	R(8,12)	-0.1929	3.1	!
!	R18	R(9,10)	0.0329	0.5	!
!	R20	R(10,14)	0.0496	0.8	!
!	R21	R(12,13)	0.215	3.5	!
!	R27	R(15,20)	0.0228	0.4	!
!	R28	R(16,17)	0.0271	0.4	!
!	R31	R(17,22)	-0.0316	0.5	!
!	R34	R(19,20)	-0.059	1.0	!
!	R35	R(19,21)	0.0869	1.4	!
!	A2	A(2,1,15)	-0.0202	0.3	!
!	A3	A(2,1,23)	-0.0878	1.4	!
!	A5	A(6,1,23)	0.1276	2.1	!
!	A8	A(1,2,24)	0.1191	1.9	!
!	A9	A(1,2,25)	-0.0587	0.9	!
!	A10	A(3,2,24)	-0.1274	2.1	!
!	A11	A(3,2,25)	0.0577	0.9	!
!	A16	A(3,4,5)	0.0211	0.3	!
!	A19	A(4,5,6)	-0.0586	0.9	!
!	A20	A(4,5,7)	0.0469	0.8	!
!	A24	A(5,7,26)	-0.2944	4.8	!
!	A25	A(8,7,26)	0.2857	4.6	!
!	A26	A(7,8,9)	-0.0645	1.0	!
!	A27	A(7,8,12)	-0.0194	0.3	!
!	A28	A(9,8,12)	0.0839	1.4	!
!	A29	A(8,9,10)	0.0215	0.3	!
!	A30	A(8,9,27)	0.0849	1.4	!
!	A31	A(10,9,27)	-0.1064	1.7	!
!	A33	A(4,10,14)	0.0252	0.4	!
!	A34	A(9,10,14)	-0.0223	0.4	!
!	A35	A(8,12,13)	-0.0631	1.0	!
!	A36	A(12,13,29)	0.0478	0.8	!
!	A37	A(12,13,30)	-0.2413	3.9	!
!	A38	A(12,13,31)	0.0355	0.6	!
!	A39	A(29,13,30)	0.0243	0.4	!
!	A40	A(29,13,31)	0.1028	1.7	!
!	A42	A(10,14,28)	-0.0984	1.6	!
!	A53	A(17,18,34)	0.0442	0.7	!
!	A54	A(19,18,34)	-0.0253	0.4	!
!	A55	A(18,19,20)	0.0317	0.5	!
!	A56	A(18,19,21)	-0.0262	0.4	!
!	A59	A(15,20,33)	0.116	1.9	!
!	A60	A(19,20,33)	-0.1096	1.8	!
!	A61	A(19,21,36)	-0.1234	2.0	!
!	D1	D(6,1,2,3)	-0.0203	0.3	!
!	D2	D(6,1,2,24)	-0.0983	1.6	!
!	D3	D(6,1,2,25)	-0.0562	0.9	!
!	D5	D(15,1,2,24)	-0.0777	1.3	!

D6	D(15,1,2,25)	-0.0356	0.6
D7	D(23,1,2,3)	0.0812	1.3
D9	D(23,1,2,25)	0.0452	0.7
D10	D(2,1,6,5)	0.0314	0.5
D12	D(23,1,6,5)	0.0654	1.1
D16	D(6,1,15,20)	0.0338	0.5
D17	D(23,1,15,16)	-0.1379	2.2
D18	D(23,1,15,20)	-0.1145	1.9
D21	D(24,2,3,4)	-0.0833	1.3
D22	D(24,2,3,11)	-0.0509	0.8
D23	D(25,2,3,4)	-0.0477	0.8
D25	D(2,3,4,5)	0.0282	0.5
D28	D(11,3,4,10)	-0.031	0.5
D29	D(3,4,5,6)	-0.022	0.4
D38	D(7,5,6,1)	-0.0289	0.5
D59	D(8,12,13,29)	0.0933	1.5
D61	D(8,12,13,31)	-0.099	1.6
D63	D(1,15,16,32)	0.0274	0.4
D66	D(1,15,20,19)	-0.0204	0.3

! Normal Mode 61 !

Name	Definition	Value	Relative Weight (%)
R2	R(1,6)	-0.0396	0.8
R3	R(1,15)	-0.0523	1.1
R11	R(4,10)	-0.0187	0.4
R12	R(5,6)	0.0568	1.2
R14	R(7,8)	0.0159	0.3
R16	R(8,9)	0.0158	0.3
R17	R(8,12)	-0.0661	1.4
R21	R(12,13)	0.068	1.4
R26	R(15,16)	0.084	1.8
R27	R(15,20)	-0.0256	0.5
R28	R(16,17)	-0.1049	2.2
R30	R(17,18)	0.0331	0.7
R31	R(17,22)	0.107	2.2
R32	R(18,19)	-0.1002	2.1
R34	R(19,20)	0.1605	3.4
R35	R(19,21)	-0.1486	3.1
A1	A(2,1,6)	0.0196	0.4
A3	A(2,1,23)	0.0483	1.0
A4	A(6,1,15)	-0.017	0.4
A5	A(6,1,23)	0.0163	0.3
A6	A(15,1,23)	-0.074	1.6
A8	A(1,2,24)	-0.0779	1.6
A9	A(1,2,25)	0.0448	0.9
A10	A(3,2,24)	0.078	1.6
A11	A(3,2,25)	-0.041	0.9

```
! A18   A(5,4,10)              -0.0151               0.3                    !
! A24   A(5,7,26)              -0.05                 1.0                    !
! A25   A(8,7,26)               0.0476               1.0                    !
! A26   A(7,8,9)               -0.0203               0.4                    !
! A28   A(9,8,12)               0.0275               0.6                    !
! A30   A(8,9,27)               0.0497               1.0                    !
! A31   A(10,9,27)             -0.0575               1.2                    !
! A35   A(8,12,13)             -0.0272               0.6                    !
! A36   A(12,13,29)             0.0332               0.7                    !
! A37   A(12,13,30)            -0.1208               2.5                    !
! A38   A(12,13,31)             0.0301               0.6                    !
! A40   A(29,13,31)             0.048                1.0                    !
! A45   A(16,15,20)            -0.0273               0.6                    !
! A47   A(15,16,32)             0.1987               4.2                    !
! A48   A(17,16,32)            -0.2034               4.3                    !
! A49   A(16,17,18)             0.0246               0.5                    !
! A51   A(18,17,22)            -0.0203               0.4                    !
! A52   A(17,18,19)             0.0319               0.7                    !
! A54   A(19,18,34)            -0.0279               0.6                    !
! A55   A(18,19,20)            -0.0456               1.0                    !
! A56   A(18,19,21)             0.0592               1.2                    !
! A59   A(15,20,33)            -0.1633               3.4                    !
! A60   A(19,20,33)             0.1515               3.2                    !
! A61   A(19,21,36)             0.3861               8.1                    !
! A62   A(17,22,35)             0.0692               1.4                    !
! D2    D(6,1,2,24)             0.0422               0.9                    !
! D3    D(6,1,2,25)             0.0245               0.5                    !
! D5    D(15,1,2,24)            0.0454               1.0                    !
! D6    D(15,1,2,25)            0.0277               0.6                    !
! D7    D(23,1,2,3)             0.058                1.2                    !
! D8    D(23,1,2,24)            0.1008               2.1                    !
! D9    D(23,1,2,25)            0.083                1.7                    !
! D12   D(23,1,6,5)            -0.0864               1.8                    !
! D13   D(2,1,15,16)            0.015                0.3                    !
! D16   D(6,1,15,20)           -0.0196               0.4                    !
! D17   D(23,1,15,16)           0.0282               0.6                    !
! D20   D(1,2,3,11)            -0.0199               0.4                    !
! D21   D(24,2,3,4)             0.0521               1.1                    !
! D22   D(24,2,3,11)            0.0291               0.6                    !
! D23   D(25,2,3,4)             0.0279               0.6                    !
! D28   D(11,3,4,10)            0.0193               0.4                    !
! D59   D(8,12,13,29)           0.0531               1.1                    !
! D61   D(8,12,13,31)          -0.0542               1.1                    !
! D63   D(1,15,16,32)          -0.0178               0.4                    !
--------------------------------------------------------------------------------
                          ----------------------------
                          ! Normal Mode    62        !
--------------------------                            --------------------------
! Name  Definition              Value         Relative Weight (%)             !
--------------------------------------------------------------------------------
```

```
 ! R1    R(1,2)               -0.0474               0.8                 !
 ! R2    R(1,6)               -0.0317               0.5                 !
 ! R3    R(1,15)               0.0737               1.3                 !
 ! R5    R(2,3)                0.026                0.4                 !
 ! R8    R(3,4)               -0.0902               1.5                 !
 ! R10   R(4,5)                0.0202               0.3                 !
 ! R11   R(4,10)               0.0613               1.0                 !
 ! R12   R(5,6)               -0.0615               1.0                 !
 ! R13   R(5,7)                0.0775               1.3                 !
 ! R16   R(8,9)                0.0808               1.4                 !
 ! R17   R(8,12)              -0.0411               0.7                 !
 ! R18   R(9,10)              -0.0592               1.0                 !
 ! R21   R(12,13)              0.0312               0.5                 !
 ! R26   R(15,16)             -0.0222               0.4                 !
 ! R28   R(16,17)              0.0369               0.6                 !
 ! R31   R(17,22)             -0.0587               1.0                 !
 ! R32   R(18,19)              0.0255               0.4                 !
 ! R34   R(19,20)             -0.0272               0.5                 !
 ! R35   R(19,21)              0.0229               0.4                 !
 ! A1    A(2,1,6)              0.0442               0.8                 !
 ! A3    A(2,1,23)             0.0687               1.2                 !
 ! A5    A(6,1,23)            -0.1269               2.2                 !
 ! A7    A(1,2,3)             -0.0214               0.4                 !
 ! A8    A(1,2,24)            -0.1855               3.2                 !
 ! A9    A(1,2,25)             0.1019               1.7                 !
 ! A10   A(3,2,24)             0.1672               2.9                 !
 ! A11   A(3,2,25)            -0.0641               1.1                 !
 ! A14   A(2,3,11)            -0.0254               0.4                 !
 ! A15   A(4,3,11)             0.0219               0.4                 !
 ! A17   A(3,4,10)             0.0233               0.4                 !
 ! A18   A(5,4,10)            -0.0274               0.5                 !
 ! A23   A(5,7,8)              0.0199               0.3                 !
 ! A24   A(5,7,26)             0.0761               1.3                 !
 ! A25   A(8,7,26)            -0.0961               1.6                 !
 ! A26   A(7,8,9)             -0.0277               0.5                 !
 ! A30   A(8,9,27)             0.1777               3.0                 !
 ! A31   A(10,9,27)           -0.1864               3.2                 !
 ! A32   A(4,10,9)             0.0347               0.6                 !
 ! A33   A(4,10,14)           -0.0345               0.6                 !
 ! A35   A(8,12,13)           -0.0403               0.7                 !
 ! A36   A(12,13,29)           0.1104               1.9                 !
 ! A37   A(12,13,30)          -0.2693               4.6                 !
 ! A38   A(12,13,31)           0.1034               1.8                 !
 ! A39   A(29,13,30)          -0.0261               0.4                 !
 ! A40   A(29,13,31)           0.0942               1.6                 !
 ! A41   A(30,13,31)          -0.0298               0.5                 !
 ! A42   A(10,14,28)           0.1733               3.0                 !
 ! A44   A(1,15,20)           -0.0279               0.5                 !
 ! A45   A(16,15,20)           0.0199               0.3                 !
 ! A47   A(15,16,32)          -0.1054               1.8                 !
```

A48	A(17,16,32)	0.108	1.8
A49	A(16,17,18)	-0.0193	0.3
A59	A(15,20,33)	-0.0223	0.4
A60	A(19,20,33)	0.0311	0.5
A61	A(19,21,36)	-0.0571	1.0
A62	A(17,22,35)	-0.0664	1.1
D2	D(6,1,2,24)	0.0904	1.5
D3	D(6,1,2,25)	0.0431	0.7
D4	D(15,1,2,3)	-0.0213	0.4
D5	D(15,1,2,24)	0.0519	0.9
D7	D(23,1,2,3)	-0.071	1.2
D9	D(23,1,2,25)	-0.0451	0.8
D10	D(2,1,6,5)	-0.0287	0.5
D12	D(23,1,6,5)	-0.0625	1.1
D13	D(2,1,15,16)	0.0381	0.6
D15	D(6,1,15,16)	-0.0239	0.4
D16	D(6,1,15,20)	-0.044	0.8
D17	D(23,1,15,16)	0.1283	2.2
D18	D(23,1,15,20)	0.1082	1.8
D20	D(1,2,3,11)	-0.0413	0.7
D21	D(24,2,3,4)	0.1325	2.3
D22	D(24,2,3,11)	0.0931	1.6
D23	D(25,2,3,4)	0.0685	1.2
D24	D(25,2,3,11)	0.0291	0.5
D25	D(2,3,4,5)	-0.0292	0.5
D28	D(11,3,4,10)	0.0393	0.7
D34	D(3,4,10,14)	-0.0214	0.4
D37	D(4,5,6,1)	0.0277	0.5
D38	D(7,5,6,1)	0.0322	0.6
D59	D(8,12,13,29)	0.1359	2.3
D61	D(8,12,13,31)	-0.1389	2.4
D63	D(1,15,16,32)	-0.023	0.4

Normal Mode 63

Name	Definition	Value	Relative Weight (%)
R1	R(1,2)	0.0522	0.9
R2	R(1,6)	0.1012	1.8
R3	R(1,15)	-0.0709	1.2
R5	R(2,3)	-0.0483	0.9
R8	R(3,4)	0.0676	1.2
R10	R(4,5)	-0.0802	1.4
R11	R(4,10)	0.1096	1.9
R12	R(5,6)	-0.1049	1.8
R13	R(5,7)	0.0273	0.5
R14	R(7,8)	-0.0459	0.8
R16	R(8,9)	0.0598	1.1
R17	R(8,12)	0.0251	0.4

```
! R18   R(9,10)              -0.1528             2.7                 !
! R20   R(10,14)              0.0182             0.3                 !
! R21   R(12,13)             -0.0273             0.5                 !
! R28   R(16,17)             -0.0487             0.9                 !
! R30   R(17,18)              0.0281             0.5                 !
! R31   R(17,22)              0.0591             1.0                 !
! R35   R(19,21)             -0.0271             0.5                 !
! A1    A(2,1,6)             -0.0775             1.4                 !
! A3    A(2,1,23)            -0.0372             0.7                 !
! A5    A(6,1,23)             0.065              1.1                 !
! A6    A(15,1,23)            0.0394             0.7                 !
! A7    A(1,2,3)              0.0317             0.6                 !
! A8    A(1,2,24)             0.2137             3.8                 !
! A9    A(1,2,25)            -0.1444             2.5                 !
! A10   A(3,2,24)            -0.1722             3.0                 !
! A11   A(3,2,25)             0.0812             1.4                 !
! A13   A(2,3,4)             -0.0351             0.6                 !
! A14   A(2,3,11)             0.0316             0.6                 !
! A17   A(3,4,10)            -0.0284             0.5                 !
! A19   A(4,5,6)              0.0258             0.5                 !
! A20   A(4,5,7)             -0.0315             0.6                 !
! A23   A(5,7,8)              0.0286             0.5                 !
! A25   A(8,7,26)            -0.0179             0.3                 !
! A26   A(7,8,9)             -0.0241             0.4                 !
! A27   A(7,8,12)             0.0286             0.5                 !
! A29   A(8,9,10)             0.0221             0.4                 !
! A30   A(8,9,27)             0.154              2.7                 !
! A31   A(10,9,27)           -0.176              3.1                 !
! A34   A(9,10,14)            0.0242             0.4                 !
! A36   A(12,13,29)           0.0273             0.5                 !
! A37   A(12,13,30)          -0.0408             0.7                 !
! A38   A(12,13,31)           0.0233             0.4                 !
! A42   A(10,14,28)           0.3094             5.4                 !
! A44   A(1,15,20)            0.0368             0.6                 !
! A45   A(16,15,20)          -0.0186             0.3                 !
! A47   A(15,16,32)           0.1172             2.1                 !
! A48   A(17,16,32)          -0.1202             2.1                 !
! A49   A(16,17,18)           0.024              0.4                 !
! A51   A(18,17,22)          -0.0191             0.3                 !
! A52   A(17,18,19)          -0.0211             0.4                 !
! A53   A(17,18,34)          -0.0341             0.6                 !
! A54   A(19,18,34)           0.0552             1.0                 !
! A59   A(15,20,33)           0.0393             0.7                 !
! A60   A(19,20,33)          -0.0567             1.0                 !
! A61   A(19,21,36)          -0.0578             1.0                 !
! A62   A(17,22,35)           0.1553             2.7                 !
! D2    D(6,1,2,24)          -0.0644             1.1                 !
! D3    D(6,1,2,25)          -0.0354             0.6                 !
! D4    D(15,1,2,3)           0.0277             0.5                 !
! D5    D(15,1,2,24)         -0.0268             0.5                 !
```

D8	D(23,1,2,24)	-0.0517	0.9
D9	D(23,1,2,25)	-0.0226	0.4
D10	D(2,1,6,5)	0.0193	0.3
D11	D(15,1,6,5)	-0.0196	0.3
D12	D(23,1,6,5)	0.0688	1.2
D13	D(2,1,15,16)	-0.0574	1.0
D14	D(2,1,15,20)	-0.0354	0.6
D15	D(6,1,15,16)	0.0308	0.5
D16	D(6,1,15,20)	0.0528	0.9
D17	D(23,1,15,16)	-0.0754	1.3
D18	D(23,1,15,20)	-0.0534	0.9
D20	D(1,2,3,11)	0.0577	1.0
D21	D(24,2,3,4)	-0.1587	2.8
D22	D(24,2,3,11)	-0.1147	2.0
D23	D(25,2,3,4)	-0.0917	1.6
D24	D(25,2,3,11)	-0.0476	0.8
D25	D(2,3,4,5)	0.0257	0.5
D27	D(11,3,4,5)	-0.0213	0.4
D28	D(11,3,4,10)	-0.0448	0.8
D34	D(3,4,10,14)	0.0185	0.3
D37	D(4,5,6,1)	-0.0355	0.6
D38	D(7,5,6,1)	-0.0311	0.5
D59	D(8,12,13,29)	0.0194	0.3
D61	D(8,12,13,31)	-0.022	0.4
D63	D(1,15,16,32)	0.023	0.4

! Normal Mode 64 !

Name	Definition	Value	Relative Weight (%)
R1	R(1,2)	-0.0293	0.8
R2	R(1,6)	-0.0406	1.1
R3	R(1,15)	0.0915	2.5
R8	R(3,4)	-0.0213	0.6
R10	R(4,5)	0.0286	0.8
R11	R(4,10)	-0.0272	0.7
R12	R(5,6)	0.0305	0.8
R14	R(7,8)	0.0131	0.4
R18	R(9,10)	0.0374	1.0
R20	R(10,14)	-0.0144	0.4
R28	R(16,17)	-0.0903	2.5
R30	R(17,18)	0.0861	2.4
R32	R(18,19)	0.0638	1.8
R34	R(19,20)	-0.0507	1.4
R35	R(19,21)	-0.0793	2.2
A1	A(2,1,6)	0.0386	1.1
A3	A(2,1,23)	0.0221	0.6
A4	A(6,1,15)	-0.0214	0.6
A5	A(6,1,23)	-0.0319	0.9

A7	A(1,2,3)	-0.0194	0.5
A8	A(1,2,24)	-0.1091	3.0
A9	A(1,2,25)	0.0361	1.0
A10	A(3,2,24)	0.0996	2.7
A11	A(3,2,25)	-0.0128	0.4
A13	A(2,3,4)	0.0133	0.4
A22	A(1,6,5)	-0.0181	0.5
A30	A(8,9,27)	-0.0331	0.9
A31	A(10,9,27)	0.0354	1.0
A37	A(12,13,30)	-0.018	0.5
A42	A(10,14,28)	-0.0685	1.9
A47	A(15,16,32)	0.1144	3.1
A48	A(17,16,32)	-0.1169	3.2
A49	A(16,17,18)	0.0209	0.6
A51	A(18,17,22)	-0.0285	0.8
A52	A(17,18,19)	-0.0373	1.0
A53	A(17,18,34)	-0.0827	2.3
A54	A(19,18,34)	0.12	3.3
A55	A(18,19,20)	-0.0175	0.5
A56	A(18,19,21)	-0.0126	0.3
A57	A(20,19,21)	0.0302	0.8
A58	A(15,20,19)	0.0293	0.8
A59	A(15,20,33)	-0.0454	1.2
A60	A(19,20,33)	0.0161	0.4
A61	A(19,21,36)	-0.3006	8.2
A62	A(17,22,35)	0.3916	10.7
D2	D(6,1,2,24)	0.0315	0.9
D5	D(15,1,2,24)	0.0341	0.9
D7	D(23,1,2,3)	-0.0126	0.3
D8	D(23,1,2,24)	0.0278	0.8
D12	D(23,1,6,5)	-0.0286	0.8
D13	D(2,1,15,16)	0.0202	0.6
D16	D(6,1,15,20)	-0.024	0.7
D17	D(23,1,15,16)	0.0418	1.1
D18	D(23,1,15,20)	0.0286	0.8
D20	D(1,2,3,11)	-0.0239	0.7
D21	D(24,2,3,4)	0.0826	2.3
D22	D(24,2,3,11)	0.0585	1.6
D23	D(25,2,3,4)	0.0242	0.7
D25	D(2,3,4,5)	-0.0158	0.4
D28	D(11,3,4,10)	0.0231	0.6
D37	D(4,5,6,1)	0.0132	0.4
D38	D(7,5,6,1)	0.0136	0.4
D63	D(1,15,16,32)	-0.0162	0.4

! Normal Mode 65 !

Name	Definition	Value	Relative Weight (%)

```
! R1    R(1,2)              -0.0405              0.7                 !
! R2    R(1,6)              -0.0469              0.8                 !
! R3    R(1,15)              0.03                0.5                 !
! R5    R(2,3)              -0.0743              1.2                 !
! R8    R(3,4)               0.0488              0.8                 !
! R10   R(4,5)              -0.0273              0.5                 !
! R11   R(4,10)              0.0527              0.9                 !
! R12   R(5,6)               0.0499              0.8                 !
! R13   R(5,7)              -0.0421              0.7                 !
! R16   R(8,9)              -0.0334              0.6                 !
! R17   R(8,12)             -0.1302              2.1                 !
! R18   R(9,10)             -0.0613              1.0                 !
! R20   R(10,14)             0.0489              0.8                 !
! R21   R(12,13)             0.0876              1.4                 !
! R31   R(17,22)            -0.0194              0.3                 !
! A1    A(2,1,6)             0.0268              0.4                 !
! A2    A(2,1,15)            0.0269              0.4                 !
! A3    A(2,1,23)            0.0769              1.3                 !
! A5    A(6,1,23)           -0.0236              0.4                 !
! A6    A(15,1,23)          -0.099               1.6                 !
! A8    A(1,2,24)           -0.1782              2.9                 !
! A9    A(1,2,25)            0.0767              1.3                 !
! A10   A(3,2,24)            0.1975              3.3                 !
! A11   A(3,2,25)           -0.1044              1.7                 !
! A13   A(2,3,4)            -0.0218              0.4                 !
! A14   A(2,3,11)            0.0379              0.6                 !
! A16   A(3,4,5)             0.0302              0.5                 !
! A18   A(5,4,10)           -0.0308              0.5                 !
! A19   A(4,5,6)            -0.0446              0.7                 !
! A21   A(6,5,7)             0.0274              0.5                 !
! A24   A(5,7,26)           -0.1754              2.9                 !
! A25   A(8,7,26)            0.1595              2.6                 !
! A26   A(7,8,9)            -0.0354              0.6                 !
! A28   A(9,8,12)            0.0347              0.6                 !
! A29   A(8,9,10)            0.0357              0.6                 !
! A30   A(8,9,27)            0.084               1.4                 !
! A31   A(10,9,27)          -0.1197              2.0                 !
! A35   A(8,12,13)           0.0576              0.9                 !
! A36   A(12,13,29)         -0.1656              2.7                 !
! A37   A(12,13,30)          0.2613              4.3                 !
! A38   A(12,13,31)         -0.1624              2.7                 !
! A39   A(29,13,30)          0.087               1.4                 !
! A40   A(29,13,31)         -0.0885              1.5                 !
! A41   A(30,13,31)          0.0896              1.5                 !
! A42   A(10,14,28)          0.2399              4.0                 !
! A61   A(19,21,36)         -0.0386              0.6                 !
! A62   A(17,22,35)         -0.0271              0.4                 !
! D2    D(6,1,2,24)          0.1294              2.1                 !
! D3    D(6,1,2,25)          0.0626              1.0                 !
! D4    D(15,1,2,3)         -0.0306              0.5                 !
```

```
! D5    D(15,1,2,24)            0.1071                  1.8                      !
! D6    D(15,1,2,25)            0.0403                  0.7                      !
! D7    D(23,1,2,3)             0.0226                  0.4                      !
! D8    D(23,1,2,24)            0.1604                  2.6                      !
! D9    D(23,1,2,25)            0.0935                  1.5                      !
! D10   D(2,1,6,5)             -0.0211                  0.3                      !
! D11   D(15,1,6,5)             0.0218                  0.4                      !
! D12   D(23,1,6,5)            -0.1138                  1.9                      !
! D13   D(2,1,15,16)            0.0309                  0.5                      !
! D16   D(6,1,15,20)           -0.0464                  0.8                      !
! D17   D(23,1,15,16)           0.0761                  1.3                      !
! D18   D(23,1,15,20)           0.0423                  0.7                      !
! D20   D(1,2,3,11)            -0.0311                  0.5                      !
! D21   D(24,2,3,4)             0.1004                  1.7                      !
! D22   D(24,2,3,11)            0.0527                  0.9                      !
! D23   D(25,2,3,4)             0.0503                  0.8                      !
! D25   D(2,3,4,5)             -0.0273                  0.4                      !
! D27   D(11,3,4,5)             0.0221                  0.4                      !
! D28   D(11,3,4,10)            0.0407                  0.7                      !
! D34   D(3,4,10,14)           -0.0204                  0.3                      !
! D37   D(4,5,6,1)              0.0218                  0.4                      !
! D59   D(8,12,13,29)          -0.1731                  2.9                      !
! D61   D(8,12,13,31)           0.176                   2.9                      !
! D62   D(1,15,16,17)          -0.0192                  0.3                      !
! D63   D(1,15,16,32)          -0.0335                  0.6                      !
! D66   D(1,15,20,19)           0.0195                  0.3                      !
! D67   D(1,15,20,33)           0.0299                  0.5                      !
--------------------------------------------------------------------------------
                          ----------------------------
                          ! Normal Mode    66        !
--------------------------                            --------------------------
! Name  Definition              Value          Relative Weight (%)             !
--------------------------------------------------------------------------------
! R5    R(2,3)                  0.1924                  2.5                      !
! R8    R(3,4)                 -0.235                   3.1                      !
! R9    R(3,11)                -0.0261                  0.3                      !
! R10   R(4,5)                  0.0992                  1.3                      !
! R11   R(4,10)                -0.0749                  1.0                      !
! R12   R(5,6)                 -0.0567                  0.7                      !
! R13   R(5,7)                  0.0979                  1.3                      !
! R14   R(7,8)                  0.0756                  1.0                      !
! R17   R(8,12)                -0.2277                  3.0                      !
! R18   R(9,10)                 0.0528                  0.7                      !
! R20   R(10,14)                0.191                   2.5                      !
! R21   R(12,13)                0.1109                  1.4                      !
! R26   R(15,16)               -0.0305                  0.4                      !
! R35   R(19,21)               -0.0264                  0.3                      !
! A2    A(2,1,15)              -0.0473                  0.6                      !
! A3    A(2,1,23)              -0.1093                  1.4                      !
! A5    A(6,1,23)              -0.0349                  0.5                      !
```

!	A6	A(15,1,23)	0.1652	2.1	!
!	A7	A(1,2,3)	-0.0464	0.6	!
!	A8	A(1,2,24)	0.1495	1.9	!
!	A10	A(3,2,24)	-0.2191	2.8	!
!	A11	A(3,2,25)	0.0687	0.9	!
!	A13	A(2,3,4)	0.0372	0.5	!
!	A14	A(2,3,11)	-0.0995	1.3	!
!	A15	A(4,3,11)	0.0636	0.8	!
!	A17	A(3,4,10)	0.0857	1.1	!
!	A18	A(5,4,10)	-0.0841	1.1	!
!	A19	A(4,5,6)	0.0274	0.4	!
!	A21	A(6,5,7)	-0.0439	0.6	!
!	A24	A(5,7,26)	0.2125	2.8	!
!	A25	A(8,7,26)	-0.2137	2.8	!
!	A26	A(7,8,9)	-0.0247	0.3	!
!	A29	A(8,9,10)	-0.03	0.4	!
!	A30	A(8,9,27)	0.2868	3.7	!
!	A31	A(10,9,27)	-0.2567	3.3	!
!	A32	A(4,10,9)	0.1211	1.6	!
!	A33	A(4,10,14)	-0.0836	1.1	!
!	A34	A(9,10,14)	-0.0375	0.5	!
!	A35	A(8,12,13)	0.0671	0.9	!
!	A36	A(12,13,29)	-0.1484	1.9	!
!	A37	A(12,13,30)	0.1499	2.0	!
!	A38	A(12,13,31)	-0.1481	1.9	!
!	A39	A(29,13,30)	0.1053	1.4	!
!	A40	A(29,13,31)	-0.0472	0.6	!
!	A41	A(30,13,31)	0.105	1.4	!
!	A42	A(10,14,28)	-0.1358	1.8	!
!	A61	A(19,21,36)	0.0365	0.5	!
!	A62	A(17,22,35)	0.0592	0.8	!
!	D1	D(6,1,2,3)	0.034	0.4	!
!	D2	D(6,1,2,24)	-0.1703	2.2	!
!	D3	D(6,1,2,25)	-0.0374	0.5	!
!	D4	D(15,1,2,3)	0.0331	0.4	!
!	D5	D(15,1,2,24)	-0.1711	2.2	!
!	D6	D(15,1,2,25)	-0.0382	0.5	!
!	D7	D(23,1,2,3)	-0.0677	0.9	!
!	D8	D(23,1,2,24)	-0.272	3.5	!
!	D9	D(23,1,2,25)	-0.1391	1.8	!
!	D12	D(23,1,6,5)	0.1645	2.1	!
!	D14	D(2,1,15,20)	0.0331	0.4	!
!	D16	D(6,1,15,20)	0.0416	0.5	!
!	D17	D(23,1,15,16)	-0.0631	0.8	!
!	D19	D(1,2,3,4)	-0.0424	0.6	!
!	D21	D(24,2,3,4)	-0.0562	0.7	!
!	D24	D(25,2,3,11)	0.0543	0.7	!
!	D25	D(2,3,4,5)	0.0298	0.4	!
!	D28	D(11,3,4,10)	-0.0418	0.5	!
!	D59	D(8,12,13,29)	-0.1369	1.8	!

D61	D(8,12,13,31)	0.1364	1.8
D63	D(1,15,16,32)	0.0394	0.5
D67	D(1,15,20,33)	-0.0387	0.5

! Normal Mode 67 !

Name	Definition	Value	Relative Weight (%)
R1	R(1,2)	0.1037	1.6
R2	R(1,6)	0.0502	0.8
R3	R(1,15)	0.0209	0.3
R5	R(2,3)	-0.1054	1.6
R9	R(3,11)	-0.0401	0.6
R11	R(4,10)	-0.0383	0.6
R17	R(8,12)	-0.0218	0.3
R20	R(10,14)	0.0285	0.4
R26	R(15,16)	0.0503	0.8
R27	R(15,20)	-0.035	0.5
R31	R(17,22)	-0.023	0.4
A1	A(2,1,6)	-0.0265	0.4
A2	A(2,1,15)	0.0291	0.4
A3	A(2,1,23)	0.2276	3.5
A4	A(6,1,15)	-0.0489	0.7
A5	A(6,1,23)	0.0526	0.8
A6	A(15,1,23)	-0.2345	3.6
A8	A(1,2,24)	-0.1179	1.8
A9	A(1,2,25)	-0.4031	6.1
A10	A(3,2,24)	0.2065	3.1
A11	A(3,2,25)	0.3677	5.6
A12	A(24,2,25)	-0.0214	0.3
A13	A(2,3,4)	0.0326	0.5
A14	A(2,3,11)	-0.0269	0.4
A16	A(3,4,5)	-0.0235	0.4
A17	A(3,4,10)	0.0225	0.3
A19	A(4,5,6)	0.0269	0.4
A21	A(6,5,7)	-0.0246	0.4
A22	A(1,6,5)	-0.033	0.5
A24	A(5,7,26)	0.0833	1.3
A25	A(8,7,26)	-0.0787	1.2
A30	A(8,9,27)	0.0788	1.2
A31	A(10,9,27)	-0.0711	1.1
A42	A(10,14,28)	-0.0931	1.4
A43	A(1,15,16)	-0.0398	0.6
A44	A(1,15,20)	0.041	0.6
A47	A(15,16,32)	0.039	0.6
A48	A(17,16,32)	-0.0277	0.4
A53	A(17,18,34)	0.0218	0.3
A54	A(19,18,34)	-0.026	0.4
A59	A(15,20,33)	-0.0644	1.0

```
! A60   A(19,20,33)             0.0504                  0.8                   !
! A61   A(19,21,36)            -0.0755                  1.2                   !
! A62   A(17,22,35)            -0.0748                  1.1                   !
! D1    D(6,1,2,3)             -0.0426                  0.6                   !
! D2    D(6,1,2,24)             0.1273                  1.9                   !
! D3    D(6,1,2,25)            -0.2215                  3.4                   !
! D5    D(15,1,2,24)            0.1893                  2.9                   !
! D6    D(15,1,2,25)           -0.1595                  2.4                   !
! D7    D(23,1,2,3)             0.1361                  2.1                   !
! D8    D(23,1,2,24)            0.306                   4.7                   !
! D9    D(23,1,2,25)           -0.0428                  0.7                   !
! D12   D(23,1,6,5)            -0.278                   4.2                   !
! D13   D(2,1,15,16)           -0.0245                  0.4                   !
! D14   D(2,1,15,20)           -0.0597                  0.9                   !
! D15   D(6,1,15,16)            0.0248                  0.4                   !
! D17   D(23,1,15,16)           0.1175                  1.8                   !
! D18   D(23,1,15,20)           0.0822                  1.3                   !
! D19   D(1,2,3,4)              0.0522                  0.8                   !
! D21   D(24,2,3,4)             0.0737                  1.1                   !
! D22   D(24,2,3,11)            0.0296                  0.5                   !
! D23   D(25,2,3,4)            -0.2137                  3.3                   !
! D24   D(25,2,3,11)           -0.2577                  3.9                   !
! D25   D(2,3,4,5)             -0.0406                  0.6                   !
! D26   D(2,3,4,10)            -0.032                   0.5                   !
! D63   D(1,15,16,32)          -0.0263                  0.4                   !
! D64   D(20,15,16,17)          0.0207                  0.3                   !
! D67   D(1,15,20,33)           0.03                    0.5                   !
--------------------------------------------------------------------------------
                          ----------------------------
                          ! Normal Mode    68        !
--------------------------                            --------------------------
! Name  Definition              Value          Relative Weight (%)            !
--------------------------------------------------------------------------------
! R1    R(1,2)                 -0.0542                  0.9                   !
! R5    R(2,3)                 -0.16                    2.7                   !
! R8    R(3,4)                  0.3449                  5.7                   !
! R10   R(4,5)                 -0.1367                  2.3                   !
! R11   R(4,10)                -0.1375                  2.3                   !
! R12   R(5,6)                  0.0704                  1.2                   !
! R14   R(7,8)                 -0.0485                  0.8                   !
! R16   R(8,9)                  0.0413                  0.7                   !
! R17   R(8,12)                 0.0609                  1.0                   !
! R18   R(9,10)                -0.1212                  2.0                   !
! R20   R(10,14)                0.1678                  2.8                   !
! R21   R(12,13)               -0.0494                  0.8                   !
! A2    A(2,1,15)               0.0267                  0.4                   !
! A3    A(2,1,23)              -0.0499                  0.8                   !
! A5    A(6,1,23)               0.0249                  0.4                   !
! A7    A(1,2,3)                0.0601                  1.0                   !
! A8    A(1,2,24)              -0.0433                  0.7                   !
```

! A9	A(1,2,25)	0.1354	2.3	!
! A10	A(3,2,24)	0.0643	1.1	!
! A11	A(3,2,25)	-0.2028	3.4	!
! A14	A(2,3,11)	0.136	2.3	!
! A15	A(4,3,11)	-0.1291	2.2	!
! A16	A(3,4,5)	-0.11	1.8	!
! A17	A(3,4,10)	0.038	0.6	!
! A18	A(5,4,10)	0.0721	1.2	!
! A19	A(4,5,6)	0.0975	1.6	!
! A20	A(4,5,7)	-0.0324	0.5	!
! A21	A(6,5,7)	-0.0651	1.1	!
! A22	A(1,6,5)	-0.0403	0.7	!
! A24	A(5,7,26)	0.2289	3.8	!
! A25	A(8,7,26)	-0.2105	3.5	!
! A27	A(7,8,12)	0.0529	0.9	!
! A28	A(9,8,12)	-0.057	1.0	!
! A29	A(8,9,10)	-0.0355	0.6	!
! A30	A(8,9,27)	0.2832	4.7	!
! A31	A(10,9,27)	-0.2477	4.1	!
! A33	A(4,10,14)	-0.032	0.5	!
! A34	A(9,10,14)	0.0219	0.4	!
! A35	A(8,12,13)	0.04	0.7	!
! A37	A(12,13,30)	0.0193	0.3	!
! A40	A(29,13,31)	-0.0299	0.5	!
! A42	A(10,14,28)	-0.3827	6.4	!
! A47	A(15,16,32)	-0.0219	0.4	!
! A59	A(15,20,33)	0.0273	0.5	!
! A60	A(19,20,33)	-0.0273	0.5	!
! D1	D(6,1,2,3)	-0.0254	0.4	!
! D2	D(6,1,2,24)	0.0643	1.1	!
! D3	D(6,1,2,25)	0.0982	1.6	!
! D4	D(15,1,2,3)	-0.0385	0.6	!
! D5	D(15,1,2,24)	0.0511	0.9	!
! D6	D(15,1,2,25)	0.0851	1.4	!
! D7	D(23,1,2,3)	-0.02	0.3	!
! D8	D(23,1,2,24)	0.0696	1.2	!
! D9	D(23,1,2,25)	0.1036	1.7	!
! D10	D(2,1,6,5)	-0.033	0.5	!
! D16	D(6,1,15,20)	-0.0255	0.4	!
! D17	D(23,1,15,16)	-0.0336	0.6	!
! D18	D(23,1,15,20)	-0.0508	0.8	!
! D19	D(1,2,3,4)	0.0201	0.3	!
! D22	D(24,2,3,11)	-0.027	0.5	!
! D23	D(25,2,3,4)	0.0909	1.5	!
! D24	D(25,2,3,11)	0.0692	1.2	!
! D37	D(4,5,6,1)	0.0211	0.4	!

! Normal Mode 69 !

```
! Name  Definition              Value          Relative Weight (%)          !
-----------------------------------------------------------------------------
! R1    R(1,2)                 -0.0532                  1.0                 !
! R3    R(1,15)                 0.0337                  0.6                 !
! R5    R(2,3)                  0.0585                  1.1                 !
! R10   R(4,5)                  0.0189                  0.4                 !
! R13   R(5,7)                 -0.0227                  0.4                 !
! R26   R(15,16)                0.0542                  1.0                 !
! R28   R(16,17)                0.0478                  0.9                 !
! R30   R(17,18)               -0.0377                  0.7                 !
! R31   R(17,22)               -0.0208                  0.4                 !
! R32   R(18,19)                0.0339                  0.6                 !
! R34   R(19,20)               -0.0554                  1.0                 !
! R35   R(19,21)                0.0338                  0.6                 !
! A1    A(2,1,6)                0.0183                  0.3                 !
! A2    A(2,1,15)              -0.0336                  0.6                 !
! A3    A(2,1,23)              -0.0368                  0.7                 !
! A4    A(6,1,15)              -0.0415                  0.8                 !
! A5    A(6,1,23)               0.119                   2.2                 !
! A7    A(1,2,3)               -0.018                   0.3                 !
! A8    A(1,2,24)               0.0926                  1.7                 !
! A9    A(1,2,25)               0.1217                  2.3                 !
! A10   A(3,2,24)              -0.1082                  2.0                 !
! A11   A(3,2,25)              -0.1205                  2.3                 !
! A12   A(24,2,25)              0.0275                  0.5                 !
! A22   A(1,6,5)               -0.0187                  0.3                 !
! A24   A(5,7,26)               0.0305                  0.6                 !
! A25   A(8,7,26)              -0.0339                  0.6                 !
! A30   A(8,9,27)               0.0257                  0.5                 !
! A31   A(10,9,27)             -0.0215                  0.4                 !
! A43   A(1,15,16)             -0.0621                  1.2                 !
! A44   A(1,15,20)              0.0709                  1.3                 !
! A46   A(15,16,17)            -0.0203                  0.4                 !
! A47   A(15,16,32)             0.2812                  5.2                 !
! A48   A(17,16,32)            -0.2609                  4.9                 !
! A50   A(16,17,22)             0.0281                  0.5                 !
! A51   A(18,17,22)            -0.0268                  0.5                 !
! A53   A(17,18,34)             0.2121                  4.0                 !
! A54   A(19,18,34)            -0.2205                  4.1                 !
! A56   A(18,19,21)             0.041                   0.8                 !
! A57   A(20,19,21)            -0.0414                  0.8                 !
! A58   A(15,20,19)             0.0213                  0.4                 !
! A59   A(15,20,33)            -0.3296                  6.2                 !
! A60   A(19,20,33)             0.3085                  5.8                 !
! A61   A(19,21,36)            -0.2457                  4.6                 !
! A62   A(17,22,35)            -0.2037                  3.8                 !
! D1    D(6,1,2,3)             -0.0236                  0.4                 !
! D2    D(6,1,2,24)            -0.1091                  2.0                 !
! D3    D(6,1,2,25)             0.0584                  1.1                 !
! D4    D(15,1,2,3)             0.0389                  0.7                 !
```

!	D5	D(15,1,2,24)	-0.0466	0.9
!	D6	D(15,1,2,25)	0.1209	2.3
!	D7	D(23,1,2,3)	0.109	2.0
!	D8	D(23,1,2,24)	0.0235	0.4
!	D9	D(23,1,2,25)	0.191	3.6
!	D10	D(2,1,6,5)	0.0399	0.7
!	D11	D(15,1,6,5)	-0.0169	0.3
!	D13	D(2,1,15,16)	-0.0199	0.4
!	D17	D(23,1,15,16)	-0.1014	1.9
!	D18	D(23,1,15,20)	-0.0947	1.8
!	D21	D(24,2,3,4)	-0.0397	0.7
!	D22	D(24,2,3,11)	-0.0245	0.5
!	D23	D(25,2,3,4)	0.0523	1.0
!	D24	D(25,2,3,11)	0.0675	1.3

! Normal Mode 70 !

! Name	Definition	Value	Relative Weight (%)
! R1	R(1,2)	-0.0678	0.8
! R3	R(1,15)	-0.042	0.5
! R5	R(2,3)	0.0833	0.9
! R10	R(4,5)	0.0934	1.1
! R11	R(4,10)	-0.0591	0.7
! R12	R(5,6)	-0.0311	0.4
! R13	R(5,7)	-0.0798	0.9
! R14	R(7,8)	0.0711	0.8
! R16	R(8,9)	-0.0665	0.8
! R18	R(9,10)	0.0603	0.7
! R26	R(15,16)	0.2722	3.1
! R27	R(15,20)	-0.1127	1.3
! R28	R(16,17)	-0.0333	0.4
! R30	R(17,18)	0.1976	2.2
! R31	R(17,22)	-0.2523	2.9
! R32	R(18,19)	-0.0477	0.5
! R34	R(19,20)	0.1683	1.9
! R35	R(19,21)	-0.1929	2.2
! A1	A(2,1,6)	0.0351	0.4
! A2	A(2,1,15)	-0.0456	0.5
! A3	A(2,1,23)	0.0574	0.7
! A4	A(6,1,15)	-0.0512	0.6
! A5	A(6,1,23)	0.298	3.4
! A6	A(15,1,23)	-0.2743	3.1
! A7	A(1,2,3)	-0.0362	0.4
! A8	A(1,2,24)	0.1327	1.5
! A9	A(1,2,25)	0.1386	1.6
! A10	A(3,2,24)	-0.1515	1.7
! A11	A(3,2,25)	-0.1397	1.6
! A12	A(24,2,25)	0.0505	0.6

Name	Definition	Value	Relative Weight (%)
A24	A(5,7,26)	0.0423	0.5
A25	A(8,7,26)	-0.0534	0.6
A30	A(8,9,27)	0.0584	0.7
A31	A(10,9,27)	-0.0568	0.6
A42	A(10,14,28)	0.0815	0.9
A44	A(1,15,20)	0.0774	0.9
A45	A(16,15,20)	-0.0782	0.9
A46	A(15,16,17)	0.062	0.7
A47	A(15,16,32)	-0.2908	3.3
A48	A(17,16,32)	0.2289	2.6
A49	A(16,17,18)	-0.1016	1.2
A50	A(16,17,22)	0.0418	0.5
A51	A(18,17,22)	0.0598	0.7
A52	A(17,18,19)	0.1078	1.2
A53	A(17,18,34)	-0.2733	3.1
A54	A(19,18,34)	0.1655	1.9
A55	A(18,19,20)	-0.0967	1.1
A56	A(18,19,21)	0.0557	0.6
A57	A(20,19,21)	0.041	0.5
A58	A(15,20,19)	0.1068	1.2
A59	A(15,20,33)	0.043	0.5
A60	A(19,20,33)	-0.15	1.7
A61	A(19,21,36)	-0.0913	1.0
A62	A(17,22,35)	-0.0286	0.3
D1	D(6,1,2,3)	-0.0321	0.4
D2	D(6,1,2,24)	-0.1567	1.8
D3	D(6,1,2,25)	0.0752	0.9
D4	D(15,1,2,3)	0.0389	0.4
D5	D(15,1,2,24)	-0.0858	1.0
D6	D(15,1,2,25)	0.1462	1.7
D7	D(23,1,2,3)	0.3796	4.3
D8	D(23,1,2,24)	0.2549	2.9
D9	D(23,1,2,25)	0.4869	5.5
D10	D(2,1,6,5)	0.063	0.7
D12	D(23,1,6,5)	-0.1933	2.2
D17	D(23,1,15,16)	-0.1593	1.8
D18	D(23,1,15,20)	-0.1706	1.9
D21	D(24,2,3,4)	-0.0544	0.6
D22	D(24,2,3,11)	-0.0395	0.4
D23	D(25,2,3,4)	0.0442	0.5
D24	D(25,2,3,11)	0.0591	0.7

! Normal Mode 71 !

Name	Definition	Value	Relative Weight (%)
R1	R(1,2)	0.149	1.9
R2	R(1,6)	-0.0913	1.2
R3	R(1,15)	-0.0312	0.4

```
! R8    R(3,4)                 -0.0986                1.3                   !
! R10   R(4,5)                 -0.1628                2.1                   !
! R11   R(4,10)                 0.0678                0.9                   !
! R12   R(5,6)                  0.1395                1.8                   !
! R13   R(5,7)                  0.0534                0.7                   !
! R14   R(7,8)                 -0.0861                1.1                   !
! R16   R(8,9)                  0.0469                0.6                   !
! R17   R(8,12)                 0.078                 1.0                   !
! R18   R(9,10)                -0.0637                0.8                   !
! R20   R(10,14)                0.1033                1.3                   !
! R21   R(12,13)               -0.0344                0.4                   !
! A2    A(2,1,15)              -0.0279                0.4                   !
! A3    A(2,1,23)              -0.4439                5.6                   !
! A4    A(6,1,15)               0.0389                0.5                   !
! A5    A(6,1,23)               0.3984                5.1                   !
! A6    A(15,1,23)              0.0608                0.8                   !
! A8    A(1,2,24)              -0.2558                3.3                   !
! A10   A(3,2,24)               0.1625                2.1                   !
! A11   A(3,2,25)               0.1035                1.3                   !
! A12   A(24,2,25)              0.042                 0.5                   !
! A14   A(2,3,11)              -0.0712                0.9                   !
! A15   A(4,3,11)               0.085                 1.1                   !
! A16   A(3,4,5)                0.0948                1.2                   !
! A17   A(3,4,10)              -0.0264                0.3                   !
! A18   A(5,4,10)              -0.0678                0.9                   !
! A20   A(4,5,7)                0.0912                1.2                   !
! A21   A(6,5,7)               -0.0682                0.9                   !
! A23   A(5,7,8)               -0.0668                0.9                   !
! A24   A(5,7,26)               0.0668                0.8                   !
! A26   A(7,8,9)                0.0501                0.6                   !
! A28   A(9,8,12)              -0.0463                0.6                   !
! A29   A(8,9,10)              -0.0623                0.8                   !
! A30   A(8,9,27)              -0.0346                0.4                   !
! A31   A(10,9,27)              0.0968                1.2                   !
! A32   A(4,10,9)               0.0555                0.7                   !
! A34   A(9,10,14)             -0.0365                0.5                   !
! A36   A(12,13,29)             0.0292                0.4                   !
! A37   A(12,13,30)             0.0346                0.4                   !
! A38   A(12,13,31)             0.029                 0.4                   !
! A39   A(29,13,30)            -0.0421                0.5                   !
! A41   A(30,13,31)            -0.0412                0.5                   !
! A42   A(10,14,28)             0.0391                0.5                   !
! D1    D(6,1,2,3)              0.035                 0.4                   !
! D2    D(6,1,2,24)             0.0569                0.7                   !
! D3    D(6,1,2,25)            -0.0675                0.9                   !
! D6    D(15,1,2,25)           -0.1012                1.3                   !
! D7    D(23,1,2,3)             0.2623                3.3                   !
! D8    D(23,1,2,24)            0.2842                3.6                   !
! D9    D(23,1,2,25)            0.1597                2.0                   !
! D12   D(23,1,6,5)             0.2893                3.7                   !
```

! D14	D(2,1,15,20)	0.0717	0.9	!
! D16	D(6,1,15,20)	0.0578	0.7	!
! D17	D(23,1,15,16)	-0.5227	6.7	!
! D18	D(23,1,15,20)	-0.4715	6.0	!
! D19	D(1,2,3,4)	-0.0449	0.6	!
! D20	D(1,2,3,11)	-0.0489	0.6	!
! D21	D(24,2,3,4)	0.1791	2.3	!
! D22	D(24,2,3,11)	0.175	2.2	!
! D37	D(4,5,6,1)	-0.0341	0.4	!
! D62	D(1,15,16,17)	0.0397	0.5	!
! D63	D(1,15,16,32)	0.0556	0.7	!
! D66	D(1,15,20,19)	-0.0423	0.5	!
! D67	D(1,15,20,33)	-0.0512	0.7	!

! Normal Mode 72 !

! Name	Definition	Value	Relative Weight (%)	!
! R1	R(1,2)	-0.0544	0.5	!
! R3	R(1,15)	-0.231	2.0	!
! R5	R(2,3)	0.0976	0.9	!
! R8	R(3,4)	-0.0714	0.6	!
! R10	R(4,5)	-0.3186	2.8	!
! R11	R(4,10)	0.2363	2.1	!
! R12	R(5,6)	0.1618	1.4	!
! R13	R(5,7)	0.2828	2.5	!
! R14	R(7,8)	-0.2656	2.4	!
! R16	R(8,9)	0.2504	2.2	!
! R17	R(8,12)	-0.0405	0.4	!
! R18	R(9,10)	-0.2284	2.0	!
! R20	R(10,14)	0.0387	0.3	!
! R26	R(15,16)	-0.0778	0.7	!
! R27	R(15,20)	0.3001	2.7	!
! R28	R(16,17)	0.296	2.6	!
! R30	R(17,18)	-0.0795	0.7	!
! R31	R(17,22)	-0.3194	2.8	!
! R32	R(18,19)	0.2819	2.5	!
! R34	R(19,20)	-0.0805	0.7	!
! R35	R(19,21)	-0.3178	2.8	!
! A3	A(2,1,23)	0.2775	2.5	!
! A5	A(6,1,23)	-0.2328	2.1	!
! A6	A(15,1,23)	-0.1003	0.9	!
! A8	A(1,2,24)	0.1346	1.2	!
! A10	A(3,2,24)	-0.1053	0.9	!
! A13	A(2,3,4)	-0.0701	0.6	!
! A15	A(4,3,11)	0.1009	0.9	!
! A16	A(3,4,5)	0.1054	0.9	!
! A17	A(3,4,10)	-0.0694	0.6	!
! A18	A(5,4,10)	-0.0361	0.3	!

Name	Definition	Value	Relative Weight (%)
A20	A(4,5,7)	0.0648	0.6
A21	A(6,5,7)	-0.0889	0.8
A22	A(1,6,5)	-0.0623	0.6
A23	A(5,7,8)	-0.0479	0.4
A24	A(5,7,26)	-0.1113	1.0
A25	A(8,7,26)	0.1591	1.4
A28	A(9,8,12)	-0.05	0.4
A30	A(8,9,27)	-0.2012	1.8
A31	A(10,9,27)	0.2208	2.0
A33	A(4,10,14)	-0.0517	0.5
A35	A(8,12,13)	0.0636	0.6
A37	A(12,13,30)	0.0632	0.6
A42	A(10,14,28)	-0.2185	1.9
A43	A(1,15,16)	0.0894	0.8
A45	A(16,15,20)	-0.1233	1.1
A46	A(15,16,17)	0.1262	1.1
A48	A(17,16,32)	-0.1535	1.4
A49	A(16,17,18)	-0.1388	1.2
A51	A(18,17,22)	0.1133	1.0
A52	A(17,18,19)	0.1466	1.3
A54	A(19,18,34)	-0.146	1.3
A55	A(18,19,20)	-0.1363	1.2
A56	A(18,19,21)	0.0417	0.4
A57	A(20,19,21)	0.0946	0.8
A58	A(15,20,19)	0.1257	1.1
A59	A(15,20,33)	-0.1981	1.8
A60	A(19,20,33)	0.0724	0.6
A61	A(19,21,36)	0.1191	1.1
A62	A(17,22,35)	0.1645	1.5
D2	D(6,1,2,24)	-0.0412	0.4
D5	D(15,1,2,24)	-0.0705	0.6
D7	D(23,1,2,3)	-0.1213	1.1
D8	D(23,1,2,24)	-0.1603	1.4
D9	D(23,1,2,25)	-0.0968	0.9
D12	D(23,1,6,5)	-0.2355	2.1
D16	D(6,1,15,20)	-0.0433	0.4
D17	D(23,1,15,16)	0.3176	2.8
D18	D(23,1,15,20)	0.2851	2.5
D21	D(24,2,3,4)	-0.0998	0.9
D22	D(24,2,3,11)	-0.082	0.7

! Normal Mode 73 !

Name	Definition	Value	Relative Weight (%)
R1	R(1,2)	0.1202	1.1
R3	R(1,15)	-0.1657	1.5
R5	R(2,3)	-0.099	0.9
R8	R(3,4)	0.0532	0.5

```
! R10   R(4,5)                  0.2534                  2.2                   !
! R11   R(4,10)                -0.2235                  2.0                   !
! R12   R(5,6)                 -0.1175                  1.0                   !
! R13   R(5,7)                 -0.2337                  2.0                   !
! R14   R(7,8)                  0.2269                  2.0                   !
! R16   R(8,9)                 -0.2475                  2.2                   !
! R17   R(8,12)                 0.0686                  0.6                   !
! R18   R(9,10)                 0.1887                  1.7                   !
! R26   R(15,16)               -0.2521                  2.2                   !
! R27   R(15,20)                0.3292                  2.9                   !
! R28   R(16,17)                0.3155                  2.8                   !
! R30   R(17,18)               -0.215                   1.9                   !
! R31   R(17,22)               -0.1473                  1.3                   !
! R32   R(18,19)                0.3047                  2.7                   !
! R34   R(19,20)               -0.2091                  1.8                   !
! R35   R(19,21)               -0.1583                  1.4                   !
! A1    A(2,1,6)               -0.0383                  0.3                   !
! A3    A(2,1,23)              -0.2206                  1.9                   !
! A4    A(6,1,15)               0.1238                  1.1                   !
! A5    A(6,1,23)               0.2131                  1.9                   !
! A6    A(15,1,23)             -0.0847                  0.7                   !
! A8    A(1,2,24)              -0.1772                  1.6                   !
! A9    A(1,2,25)              -0.0358                  0.3                   !
! A10   A(3,2,24)               0.1416                  1.2                   !
! A11   A(3,2,25)               0.0684                  0.6                   !
! A13   A(2,3,4)                0.0616                  0.5                   !
! A15   A(4,3,11)              -0.0786                  0.7                   !
! A16   A(3,4,5)               -0.0789                  0.7                   !
! A17   A(3,4,10)               0.0651                  0.6                   !
! A20   A(4,5,7)               -0.0381                  0.3                   !
! A21   A(6,5,7)                0.0689                  0.6                   !
! A22   A(1,6,5)                0.1008                  0.9                   !
! A24   A(5,7,26)               0.0786                  0.7                   !
! A25   A(8,7,26)              -0.093                   0.8                   !
! A27   A(7,8,12)              -0.0523                  0.5                   !
! A28   A(9,8,12)               0.0467                  0.4                   !
! A30   A(8,9,27)               0.1583                  1.4                   !
! A31   A(10,9,27)             -0.1585                  1.4                   !
! A33   A(4,10,14)              0.0417                  0.4                   !
! A34   A(9,10,14)             -0.0459                  0.4                   !
! A35   A(8,12,13)             -0.067                   0.6                   !
! A36   A(12,13,29)             0.037                   0.3                   !
! A37   A(12,13,30)            -0.0435                  0.4                   !
! A38   A(12,13,31)             0.0366                  0.3                   !
! A42   A(10,14,28)             0.2333                  2.0                   !
! A43   A(1,15,16)              0.1073                  0.9                   !
! A44   A(1,15,20)             -0.0554                  0.5                   !
! A45   A(16,15,20)            -0.0506                  0.4                   !
! A46   A(15,16,17)             0.0625                  0.5                   !
! A47   A(15,16,32)             0.1085                  0.9                   !
```

```
! A48   A(17,16,32)           -0.171                 1.5                   !
! A49   A(16,17,18)           -0.0608                0.5                   !
! A51   A(18,17,22)            0.096                 0.8                   !
! A52   A(17,18,19)            0.0595                0.5                   !
! A53   A(17,18,34)            0.0693                0.6                   !
! A54   A(19,18,34)           -0.1287                1.1                   !
! A55   A(18,19,20)           -0.0575                0.5                   !
! A56   A(18,19,21)           -0.0367                0.3                   !
! A57   A(20,19,21)            0.0942                0.8                   !
! A58   A(15,20,19)            0.0469                0.4                   !
! A59   A(15,20,33)           -0.1192                1.0                   !
! A60   A(19,20,33)            0.0726                0.6                   !
! A61   A(19,21,36)            0.2917                2.6                   !
! A62   A(17,22,35)            0.2729                2.4                   !
! D1    D(6,1,2,3)             0.0621                0.5                   !
! D2    D(6,1,2,24)            0.1406                1.2                   !
! D4    D(15,1,2,3)           -0.0829                0.7                   !
! D6    D(15,1,2,25)          -0.1652                1.4                   !
! D7    D(23,1,2,3)            0.1704                1.5                   !
! D8    D(23,1,2,24)           0.2488                2.2                   !
! D9    D(23,1,2,25)           0.0881                0.8                   !
! D11   D(15,1,6,5)            0.0516                0.5                   !
! D12   D(23,1,6,5)            0.1323                1.2                   !
! D13   D(2,1,15,16)           0.0445                0.4                   !
! D14   D(2,1,15,20)           0.0714                0.6                   !
! D17   D(23,1,15,16)         -0.2847                2.5                   !
! D18   D(23,1,15,20)         -0.2577                2.3                   !
! D21   D(24,2,3,4)            0.0961                0.8                   !
! D22   D(24,2,3,11)           0.0776                0.7                   !
! D67   D(1,15,20,33)         -0.0396                0.3                   !
--------------------------------------------------------------------------------
                          ----------------------------
                          ! Normal Mode    74        !
--------------------------                            --------------------------
! Name  Definition              Value         Relative Weight (%)              !
--------------------------------------------------------------------------------
! R1    R(1,2)                 0.1543                1.5                   !
! R2    R(1,6)                 0.0392                0.4                   !
! R3    R(1,15)               -0.19                  1.8                   !
! R5    R(2,3)                -0.1733                1.6                   !
! R8    R(3,4)                 0.0934                0.9                   !
! R9    R(3,11)                0.0506                0.5                   !
! R11   R(4,10)                0.1552                1.5                   !
! R12   R(5,6)                -0.2387                2.3                   !
! R13   R(5,7)                 0.0798                0.8                   !
! R16   R(8,9)                 0.1867                1.8                   !
! R17   R(8,12)               -0.2858                2.7                   !
! R18   R(9,10)                0.0554                0.5                   !
! R20   R(10,14)              -0.3388                3.2                   !
! R21   R(12,13)               0.1044                1.0                   !
```

```
! R26   R(15,16)                0.0745                  0.7                   !
! R30   R(17,18)                0.0607                  0.6                   !
! R31   R(17,22)               -0.0782                  0.7                   !
! R34   R(19,20)                0.0685                  0.6                   !
! R35   R(19,21)               -0.0776                  0.7                   !
! A1    A(2,1,6)               -0.0819                  0.8                   !
! A2    A(2,1,15)               0.0335                  0.3                   !
! A3    A(2,1,23)              -0.3854                  3.6                   !
! A4    A(6,1,15)               0.0796                  0.8                   !
! A5    A(6,1,23)               0.0774                  0.7                   !
! A6    A(15,1,23)              0.2841                  2.7                   !
! A7    A(1,2,3)                0.0569                  0.5                   !
! A8    A(1,2,24)              -0.1952                  1.8                   !
! A9    A(1,2,25)              -0.0525                  0.5                   !
! A10   A(3,2,24)               0.1797                  1.7                   !
! A11   A(3,2,25)               0.0837                  0.8                   !
! A12   A(24,2,25)             -0.0691                  0.7                   !
! A15   A(4,3,11)              -0.0496                  0.5                   !
! A16   A(3,4,5)               -0.0789                  0.7                   !
! A17   A(3,4,10)              -0.0779                  0.7                   !
! A18   A(5,4,10)               0.1575                  1.5                   !
! A19   A(4,5,6)                0.0719                  0.7                   !
! A20   A(4,5,7)               -0.1271                  1.2                   !
! A21   A(6,5,7)                0.0552                  0.5                   !
! A22   A(1,6,5)                0.0661                  0.6                   !
! A23   A(5,7,8)                0.146                   1.4                   !
! A24   A(5,7,26)              -0.0382                  0.4                   !
! A25   A(8,7,26)              -0.1077                  1.0                   !
! A26   A(7,8,9)               -0.1391                  1.3                   !
! A27   A(7,8,12)               0.1043                  1.0                   !
! A28   A(9,8,12)               0.0348                  0.3                   !
! A29   A(8,9,10)               0.1389                  1.3                   !
! A30   A(8,9,27)              -0.0743                  0.7                   !
! A31   A(10,9,27)             -0.0646                  0.6                   !
! A32   A(4,10,9)              -0.176                   1.7                   !
! A33   A(4,10,14)              0.064                   0.6                   !
! A34   A(9,10,14)              0.112                   1.1                   !
! A35   A(8,12,13)              0.0564                  0.5                   !
! A36   A(12,13,29)            -0.1464                  1.4                   !
! A37   A(12,13,30)            -0.0793                  0.7                   !
! A38   A(12,13,31)            -0.1451                  1.4                   !
! A39   A(29,13,30)             0.166                   1.6                   !
! A40   A(29,13,31)             0.045                   0.4                   !
! A41   A(30,13,31)             0.1683                  1.6                   !
! A42   A(10,14,28)            -0.178                   1.7                   !
! A44   A(1,15,20)              0.0359                  0.3                   !
! A45   A(16,15,20)            -0.0429                  0.4                   !
! A46   A(15,16,17)             0.0392                  0.4                   !
! A49   A(16,17,18)            -0.0396                  0.4                   !
! A50   A(16,17,22)             0.036                   0.3                   !
```

! A52	A(17,18,19)	0.0414	0.4	!
! A55	A(18,19,20)	-0.0426	0.4	!
! A56	A(18,19,21)	0.0393	0.4	!
! A58	A(15,20,19)	0.0446	0.4	!
! A61	A(19,21,36)	-0.0831	0.8	!
! A62	A(17,22,35)	-0.0773	0.7	!
! D1	D(6,1,2,3)	0.076	0.7	!
! D2	D(6,1,2,24)	0.2079	2.0	!
! D3	D(6,1,2,25)	-0.0339	0.3	!
! D5	D(15,1,2,24)	0.1421	1.3	!
! D6	D(15,1,2,25)	-0.0996	0.9	!
! D7	D(23,1,2,3)	-0.0985	0.9	!
! D8	D(23,1,2,24)	0.0334	0.3	!
! D9	D(23,1,2,25)	-0.2084	2.0	!
! D10	D(2,1,6,5)	-0.0674	0.6	!
! D12	D(23,1,6,5)	0.3922	3.7	!
! D16	D(6,1,15,20)	0.0537	0.5	!
! D17	D(23,1,15,16)	-0.2807	2.7	!
! D18	D(23,1,15,20)	-0.2379	2.2	!
! D21	D(24,2,3,4)	0.081	0.8	!
! D22	D(24,2,3,11)	0.0718	0.7	!
! D59	D(8,12,13,29)	-0.075	0.7	!
! D61	D(8,12,13,31)	0.0757	0.7	!
! D63	D(1,15,16,32)	0.0457	0.4	!
! D67	D(1,15,20,33)	-0.0437	0.4	!

! Normal Mode 75 !

! Name	Definition	Value	Relative Weight (%)	!
! R1	R(1,2)	-0.0908	0.9	!
! R2	R(1,6)	-0.0522	0.5	!
! R3	R(1,15)	0.2421	2.5	!
! R5	R(2,3)	0.0828	0.9	!
! R10	R(4,5)	-0.065	0.7	!
! R11	R(4,10)	0.1261	1.3	!
! R14	R(7,8)	-0.0387	0.4	!
! R16	R(8,9)	0.1435	1.5	!
! R17	R(8,12)	-0.1047	1.1	!
! R20	R(10,14)	-0.1454	1.5	!
! R26	R(15,16)	-0.1041	1.1	!
! R28	R(16,17)	0.0558	0.6	!
! R30	R(17,18)	-0.1184	1.2	!
! R31	R(17,22)	0.0748	0.8	!
! R32	R(18,19)	0.0407	0.4	!
! R34	R(19,20)	-0.145	1.5	!
! R35	R(19,21)	0.1045	1.1	!
! A1	A(2,1,6)	0.0796	0.8	!
! A2	A(2,1,15)	-0.0688	0.7	!

```
! A3    A(2,1,23)              0.1247                 1.3                  !
! A4    A(6,1,15)             -0.0318                 0.3                  !
! A5    A(6,1,23)              0.3598                 3.8                  !
! A6    A(15,1,23)            -0.4401                 4.6                  !
! A7    A(1,2,3)              -0.0752                 0.8                  !
! A8    A(1,2,24)              0.063                  0.7                  !
! A9    A(1,2,25)              0.1133                 1.2                  !
! A10   A(3,2,24)             -0.1187                 1.2                  !
! A11   A(3,2,25)             -0.1283                 1.3                  !
! A12   A(24,2,25)             0.1469                 1.5                  !
! A15   A(4,3,11)              0.0357                 0.4                  !
! A17   A(3,4,10)             -0.0686                 0.7                  !
! A18   A(5,4,10)              0.0392                 0.4                  !
! A22   A(1,6,5)              -0.0737                 0.8                  !
! A23   A(5,7,8)               0.0551                 0.6                  !
! A24   A(5,7,26)              0.0794                 0.8                  !
! A25   A(8,7,26)             -0.1345                 1.4                  !
! A26   A(7,8,9)              -0.053                  0.6                  !
! A27   A(7,8,12)              0.0837                 0.9                  !
! A28   A(9,8,12)             -0.0308                 0.3                  !
! A29   A(8,9,10)              0.0303                 0.3                  !
! A32   A(4,10,9)             -0.0697                 0.7                  !
! A33   A(4,10,14)             0.0341                 0.4                  !
! A34   A(9,10,14)             0.0356                 0.4                  !
! A35   A(8,12,13)             0.0509                 0.5                  !
! A36   A(12,13,29)           -0.0722                 0.8                  !
! A37   A(12,13,30)           -0.0389                 0.4                  !
! A38   A(12,13,31)           -0.0719                 0.8                  !
! A39   A(29,13,30)            0.0825                 0.9                  !
! A41   A(30,13,31)            0.0833                 0.9                  !
! A42   A(10,14,28)           -0.0558                 0.6                  !
! A44   A(1,15,20)            -0.0567                 0.6                  !
! A45   A(16,15,20)            0.0547                 0.6                  !
! A46   A(15,16,17)           -0.0609                 0.6                  !
! A48   A(17,16,32)            0.0516                 0.5                  !
! A49   A(16,17,18)            0.0471                 0.5                  !
! A50   A(16,17,22)           -0.0698                 0.7                  !
! A52   A(17,18,19)           -0.051                  0.5                  !
! A54   A(19,18,34)            0.0623                 0.7                  !
! A55   A(18,19,20)            0.0577                 0.6                  !
! A56   A(18,19,21)           -0.0752                 0.8                  !
! A58   A(15,20,19)           -0.0475                 0.5                  !
! A60   A(19,20,33)            0.0358                 0.4                  !
! A61   A(19,21,36)            0.178                  1.9                  !
! A62   A(17,22,35)            0.1662                 1.7                  !
! D1    D(6,1,2,3)            -0.057                  0.6                  !
! D2    D(6,1,2,24)           -0.211                  2.2                  !
! D3    D(6,1,2,25)            0.0802                 0.8                  !
! D5    D(15,1,2,24)          -0.1811                 1.9                  !
! D6    D(15,1,2,25)           0.1101                 1.1                  !
```

D7	D(23,1,2,3)	0.4931	5.1
D8	D(23,1,2,24)	0.3391	3.5
D9	D(23,1,2,25)	0.6303	6.6
D10	D(2,1,6,5)	0.0795	0.8
D12	D(23,1,6,5)	-0.3162	3.3
D13	D(2,1,15,16)	0.032	0.3
D17	D(23,1,15,16)	-0.1718	1.8
D18	D(23,1,15,20)	-0.1809	1.9
D22	D(24,2,3,11)	0.0346	0.4
D59	D(8,12,13,29)	-0.0378	0.4
D61	D(8,12,13,31)	0.0377	0.4

! Normal Mode 76 !

Name	Definition	Value	Relative Weight (%)
R1	R(1,2)	-0.0658	1.0
R3	R(1,15)	0.0227	0.4
R10	R(4,5)	-0.0309	0.5
R12	R(5,6)	0.0425	0.7
R13	R(5,7)	-0.0355	0.6
R18	R(9,10)	0.0392	0.6
A5	A(6,1,23)	0.034	0.5
A6	A(15,1,23)	-0.0343	0.5
A7	A(1,2,3)	0.0352	0.6
A8	A(1,2,24)	0.1945	3.1
A9	A(1,2,25)	0.2033	3.2
A10	A(3,2,24)	0.1389	2.2
A11	A(3,2,25)	0.1357	2.2
A12	A(24,2,25)	-0.7361	11.7
A13	A(2,3,4)	-0.0283	0.5
A16	A(3,4,5)	0.0239	0.4
A17	A(3,4,10)	-0.0218	0.3
A20	A(4,5,7)	0.0253	0.4
A24	A(5,7,26)	0.0556	0.9
A25	A(8,7,26)	-0.0545	0.9
A27	A(7,8,12)	0.0199	0.3
A30	A(8,9,27)	0.0197	0.3
A33	A(4,10,14)	0.022	0.4
A39	A(29,13,30)	0.024	0.4
A41	A(30,13,31)	0.0251	0.4
A42	A(10,14,28)	0.0377	0.6
D2	D(6,1,2,24)	0.3186	5.1
D3	D(6,1,2,25)	-0.3427	5.5
D5	D(15,1,2,24)	0.3225	5.1
D6	D(15,1,2,25)	-0.3388	5.4
D7	D(23,1,2,3)	0.0363	0.6
D8	D(23,1,2,24)	0.3588	5.7
D9	D(23,1,2,25)	-0.3025	4.8

! D12	D(23,1,6,5)	-0.0245	0.4	!
! D21	D(24,2,3,4)	-0.3599	5.7	!
! D22	D(24,2,3,11)	-0.3487	5.6	!
! D23	D(25,2,3,4)	0.3642	5.8	!
! D24	D(25,2,3,11)	0.3754	6.0	!

! Normal Mode 77 !

! Name	Definition	Value	Relative Weight (%)	!
! R3	R(1,15)	0.0714	0.8	!
! R5	R(2,3)	-0.0359	0.4	!
! R9	R(3,11)	-0.0426	0.5	!
! R10	R(4,5)	0.1909	2.1	!
! R12	R(5,6)	-0.2777	3.0	!
! R13	R(5,7)	0.2997	3.2	!
! R16	R(8,9)	-0.0747	0.8	!
! R18	R(9,10)	-0.3038	3.3	!
! R20	R(10,14)	0.1407	1.5	!
! R27	R(15,20)	-0.033	0.4	!
! R28	R(16,17)	0.0475	0.5	!
! R30	R(17,18)	-0.0344	0.4	!
! R34	R(19,20)	-0.0661	0.7	!
! R35	R(19,21)	0.0569	0.6	!
! A3	A(2,1,23)	-0.073	0.8	!
! A5	A(6,1,23)	0.2011	2.2	!
! A6	A(15,1,23)	-0.1006	1.1	!
! A8	A(1,2,24)	0.0389	0.4	!
! A9	A(1,2,25)	0.1129	1.2	!
! A10	A(3,2,24)	0.0519	0.6	!
! A12	A(24,2,25)	-0.2226	2.4	!
! A13	A(2,3,4)	0.0311	0.3	!
! A14	A(2,3,11)	0.0324	0.4	!
! A15	A(4,3,11)	-0.0635	0.7	!
! A16	A(3,4,5)	-0.1317	1.4	!
! A17	A(3,4,10)	0.1399	1.5	!
! A19	A(4,5,6)	0.1126	1.2	!
! A20	A(4,5,7)	-0.1626	1.8	!
! A21	A(6,5,7)	0.0499	0.5	!
! A24	A(5,7,26)	-0.3686	4.0	!
! A25	A(8,7,26)	0.3629	3.9	!
! A27	A(7,8,12)	-0.1318	1.4	!
! A28	A(9,8,12)	0.1348	1.5	!
! A29	A(8,9,10)	0.0829	0.9	!
! A30	A(8,9,27)	-0.1934	2.1	!
! A31	A(10,9,27)	0.1105	1.2	!
! A32	A(4,10,9)	0.085	0.9	!
! A33	A(4,10,14)	-0.1693	1.8	!
! A34	A(9,10,14)	0.0843	0.9	!

A35	A(8,12,13)	-0.0724	0.8
A36	A(12,13,29)	0.124	1.3
A37	A(12,13,30)	0.1613	1.7
A38	A(12,13,31)	0.1245	1.3
A39	A(29,13,30)	-0.2089	2.3
A41	A(30,13,31)	-0.2103	2.3
A42	A(10,14,28)	-0.2617	2.8
A46	A(15,16,17)	-0.035	0.4
A50	A(16,17,22)	-0.0346	0.4
A51	A(18,17,22)	0.0288	0.3
A53	A(17,18,34)	-0.0566	0.6
A54	A(19,18,34)	0.0674	0.7
A56	A(18,19,21)	-0.0345	0.4
A59	A(15,20,33)	-0.0391	0.4
A60	A(19,20,33)	0.04	0.4
A61	A(19,21,36)	0.0572	0.6
A62	A(17,22,35)	0.0668	0.7
D2	D(6,1,2,24)	0.0851	0.9
D3	D(6,1,2,25)	-0.096	1.0
D5	D(15,1,2,24)	0.0904	1.0
D6	D(15,1,2,25)	-0.0907	1.0
D7	D(23,1,2,3)	0.1971	2.1
D8	D(23,1,2,24)	0.2877	3.1
D9	D(23,1,2,25)	0.1067	1.2
D17	D(23,1,15,16)	-0.1802	2.0
D18	D(23,1,15,20)	-0.1663	1.8
D21	D(24,2,3,4)	-0.0889	1.0
D22	D(24,2,3,11)	-0.0907	1.0
D23	D(25,2,3,4)	0.1421	1.5
D24	D(25,2,3,11)	0.1403	1.5
D59	D(8,12,13,29)	0.0892	1.0
D61	D(8,12,13,31)	-0.0883	1.0

! Normal Mode 78 !

Name	Definition	Value	Relative Weight (%)
R8	R(3,4)	0.0448	1.2
R9	R(3,11)	-0.0227	0.6
R10	R(4,5)	-0.0211	0.5
R11	R(4,10)	-0.0745	1.9
R12	R(5,6)	-0.0178	0.5
R13	R(5,7)	0.0556	1.4
R14	R(7,8)	0.0787	2.0
R16	R(8,9)	-0.0197	0.5
R17	R(8,12)	0.0433	1.1
R18	R(9,10)	-0.1086	2.8
R20	R(10,14)	0.108	2.8
R21	R(12,13)	0.0703	1.8

R22	R(13,29)	-0.0154	0.4
R24	R(13,31)	-0.0163	0.4
A14	A(2,3,11)	0.0137	0.4
A15	A(4,3,11)	-0.0156	0.4
A16	A(3,4,5)	-0.034	0.9
A17	A(3,4,10)	0.0345	0.9
A19	A(4,5,6)	0.0465	1.2
A21	A(6,5,7)	-0.0375	1.0
A22	A(1,6,5)	-0.0221	0.6
A23	A(5,7,8)	-0.0413	1.1
A24	A(5,7,26)	0.0203	0.5
A25	A(8,7,26)	0.021	0.5
A27	A(7,8,12)	-0.0455	1.2
A28	A(9,8,12)	0.0495	1.3
A30	A(8,9,27)	-0.1894	4.9
A31	A(10,9,27)	0.1924	5.0
A32	A(4,10,9)	0.0578	1.5
A33	A(4,10,14)	-0.0248	0.6
A34	A(9,10,14)	-0.033	0.9
A35	A(8,12,13)	-0.0311	0.8
A36	A(12,13,29)	-0.299	7.7
A37	A(12,13,30)	-0.3164	8.2
A38	A(12,13,31)	-0.3032	7.8
A39	A(29,13,30)	0.2631	6.8
A40	A(29,13,31)	0.3649	9.4
A41	A(30,13,31)	0.2867	7.4
A42	A(10,14,28)	0.0341	0.9
D8	D(23,1,2,24)	0.0138	0.4
D9	D(23,1,2,25)	0.0181	0.5
D12	D(23,1,6,5)	-0.0168	0.4
D59	D(8,12,13,29)	0.0245	0.6
D61	D(8,12,13,31)	-0.0151	0.4

! Normal Mode 79 !

Name	Definition	Value	Relative Weight (%)
A36	A(12,13,29)	-0.1294	5.3
A38	A(12,13,31)	0.1136	4.7
A39	A(29,13,30)	0.5624	23.1
A41	A(30,13,31)	-0.5704	23.4
D59	D(8,12,13,29)	-0.2133	8.8
D60	D(8,12,13,30)	0.392	16.1
D61	D(8,12,13,31)	-0.2306	9.5

! Normal Mode 80 !

Name	Definition	Value	Relative Weight (%)

```
--------------------------------------------------------------------------------
! R3    R(1,15)              -0.0608              0.8                 !
! R12   R(5,6)                0.0294              0.4                 !
! R14   R(7,8)               -0.0479              0.6                 !
! R17   R(8,12)               0.0562              0.7                 !
! R26   R(15,16)              0.1925              2.5                 !
! R27   R(15,20)             -0.1211              1.6                 !
! R28   R(16,17)              0.2209              2.9                 !
! R31   R(17,22)             -0.1227              1.6                 !
! R32   R(18,19)             -0.1281              1.7                 !
! R34   R(19,20)             -0.1941              2.5                 !
! R35   R(19,21)              0.1748              2.3                 !
! A1    A(2,1,6)             -0.0253              0.3                 !
! A3    A(2,1,23)            -0.0466              0.6                 !
! A4    A(6,1,15)            -0.0507              0.7                 !
! A5    A(6,1,23)            -0.1035              1.3                 !
! A6    A(15,1,23)            0.2127              2.7                 !
! A9    A(1,2,25)            -0.039               0.5                 !
! A24   A(5,7,26)            -0.0246              0.3                 !
! A25   A(8,7,26)             0.0279              0.4                 !
! A26   A(7,8,9)              0.0246              0.3                 !
! A28   A(9,8,12)            -0.0254              0.3                 !
! A30   A(8,9,27)             0.0331              0.4                 !
! A36   A(12,13,29)           0.0418              0.5                 !
! A37   A(12,13,30)          -0.1427              1.8                 !
! A38   A(12,13,31)           0.0463              0.6                 !
! A39   A(29,13,30)           0.2457              3.2                 !
! A40   A(29,13,31)          -0.3918              5.1                 !
! A41   A(30,13,31)           0.2268              2.9                 !
! A43   A(1,15,16)           -0.1251              1.6                 !
! A44   A(1,15,20)            0.1442              1.9                 !
! A46   A(15,16,17)          -0.0812              1.0                 !
! A47   A(15,16,32)           0.1008              1.3                 !
! A49   A(16,17,18)          -0.0553              0.7                 !
! A50   A(16,17,22)          -0.0889              1.1                 !
! A51   A(18,17,22)           0.1443              1.9                 !
! A53   A(17,18,34)          -0.3359              4.3                 !
! A54   A(19,18,34)           0.3211              4.1                 !
! A55   A(18,19,20)           0.0929              1.2                 !
! A56   A(18,19,21)          -0.0951              1.2                 !
! A58   A(15,20,19)           0.0477              0.6                 !
! A59   A(15,20,33)          -0.2329              3.0                 !
! A60   A(19,20,33)           0.1853              2.4                 !
! A61   A(19,21,36)           0.1311              1.7                 !
! A62   A(17,22,35)           0.2065              2.7                 !
! D4    D(15,1,2,3)           0.0765              1.0                 !
! D5    D(15,1,2,24)          0.0846              1.1                 !
! D6    D(15,1,2,25)          0.0814              1.1                 !
! D7    D(23,1,2,3)          -0.1673              2.2                 !
! D8    D(23,1,2,24)         -0.1591              2.1                 !
```

D9	D(23,1,2,25)	-0.1623	2.1
D11	D(15,1,6,5)	-0.0406	0.5
D12	D(23,1,6,5)	0.1275	1.6
D13	D(2,1,15,16)	-0.0499	0.6
D14	D(2,1,15,20)	-0.0461	0.6
D17	D(23,1,15,16)	0.0485	0.6
D18	D(23,1,15,20)	0.0524	0.7
D59	D(8,12,13,29)	-0.2251	2.9
D61	D(8,12,13,31)	0.2174	2.8

! Normal Mode 81 !

Name	Definition	Value	Relative Weight (%)
R3	R(1,15)	0.0289	0.6
R10	R(4,5)	0.0309	0.6
R12	R(5,6)	-0.0316	0.6
R13	R(5,7)	0.0354	0.7
R14	R(7,8)	-0.0189	0.4
R17	R(8,12)	0.0425	0.8
R18	R(9,10)	-0.0367	0.7
R20	R(10,14)	0.0237	0.5
R23	R(13,30)	-0.0196	0.4
R24	R(13,31)	0.0167	0.3
R26	R(15,16)	-0.0719	1.4
R27	R(15,20)	0.0392	0.8
R28	R(16,17)	-0.0786	1.5
R31	R(17,22)	0.0447	0.9
R32	R(18,19)	0.0495	1.0
R34	R(19,20)	0.0692	1.3
R35	R(19,21)	-0.0629	1.2
A4	A(6,1,15)	0.0199	0.4
A5	A(6,1,23)	0.0531	1.0
A6	A(15,1,23)	-0.0838	1.6
A9	A(1,2,25)	0.02	0.4
A17	A(3,4,10)	0.0191	0.4
A20	A(4,5,7)	-0.0192	0.4
A24	A(5,7,26)	-0.0579	1.1
A25	A(8,7,26)	0.0614	1.2
A27	A(7,8,12)	-0.0228	0.4
A30	A(8,9,27)	-0.0249	0.5
A31	A(10,9,27)	0.0266	0.5
A32	A(4,10,9)	0.0173	0.3
A33	A(4,10,14)	-0.0226	0.4
A36	A(12,13,29)	0.0973	1.9
A37	A(12,13,30)	-0.1751	3.4
A38	A(12,13,31)	0.1047	2.0
A39	A(29,13,30)	0.3252	6.3
A40	A(29,13,31)	-0.6098	11.9

A41	A(30,13,31)	0.2957	5.7
A42	A(10,14,28)	-0.0272	0.5
A43	A(1,15,16)	0.0444	0.9
A44	A(1,15,20)	-0.0538	1.0
A46	A(15,16,17)	0.028	0.5
A47	A(15,16,32)	-0.0317	0.6
A49	A(16,17,18)	0.0203	0.4
A50	A(16,17,22)	0.0325	0.6
A51	A(18,17,22)	-0.0528	1.0
A53	A(17,18,34)	0.1222	2.4
A54	A(19,18,34)	-0.1157	2.2
A55	A(18,19,20)	-0.0341	0.7
A56	A(18,19,21)	0.0327	0.6
A58	A(15,20,19)	-0.0172	0.3
A59	A(15,20,33)	0.0878	1.7
A60	A(19,20,33)	-0.0707	1.4
A61	A(19,21,36)	-0.0441	0.9
A62	A(17,22,35)	-0.076	1.5
D4	D(15,1,2,3)	-0.0287	0.6
D5	D(15,1,2,24)	-0.031	0.6
D6	D(15,1,2,25)	-0.0332	0.6
D7	D(23,1,2,3)	0.0755	1.5
D8	D(23,1,2,24)	0.0732	1.4
D9	D(23,1,2,25)	0.071	1.4
D12	D(23,1,6,5)	-0.0439	0.9
D13	D(2,1,15,16)	0.019	0.4
D14	D(2,1,15,20)	0.0196	0.4
D17	D(23,1,15,16)	-0.0328	0.6
D18	D(23,1,15,20)	-0.0322	0.6
D59	D(8,12,13,29)	-0.3269	6.4
D61	D(8,12,13,31)	0.3154	6.1

! Normal Mode 82 !

Name	Definition	Value	Relative Weight (%)
R2	R(1,6)	-0.0506	0.7
R8	R(3,4)	-0.2029	2.7
R10	R(4,5)	0.2838	3.8
R11	R(4,10)	0.2107	2.8
R13	R(5,7)	-0.1311	1.7
R14	R(7,8)	-0.3435	4.6
R16	R(8,9)	-0.0693	0.9
R17	R(8,12)	0.2598	3.5
R18	R(9,10)	-0.0303	0.4
R20	R(10,14)	-0.0435	0.6
R21	R(12,13)	-0.0251	0.3
R26	R(15,16)	-0.0358	0.5
R27	R(15,20)	-0.0325	0.4

```
! R30   R(17,18)            0.0281                0.4                   !
! R32   R(18,19)            0.0516                0.7                   !
! R35   R(19,21)           -0.0334                0.4                   !
! A3    A(2,1,23)          -0.0288                0.4                   !
! A5    A(6,1,23)           0.0251                0.3                   !
! A11   A(3,2,25)           0.0333                0.4                   !
! A13   A(2,3,4)            0.0245                0.3                   !
! A14   A(2,3,11)          -0.0304                0.4                   !
! A16   A(3,4,5)            0.0701                0.9                   !
! A17   A(3,4,10)           0.0737                1.0                   !
! A18   A(5,4,10)          -0.1444                1.9                   !
! A19   A(4,5,6)           -0.1706                2.3                   !
! A21   A(6,5,7)            0.1847                2.5                   !
! A22   A(1,6,5)            0.0937                1.2                   !
! A23   A(5,7,8)            0.0624                0.8                   !
! A24   A(5,7,26)          -0.3023                4.0                   !
! A25   A(8,7,26)           0.2399                3.2                   !
! A26   A(7,8,9)            0.1327                1.8                   !
! A27   A(7,8,12)           0.0352                0.5                   !
! A28   A(9,8,12)          -0.1679                2.2                   !
! A29   A(8,9,10)          -0.0426                0.6                   !
! A30   A(8,9,27)           0.3874                5.1                   !
! A31   A(10,9,27)         -0.3448                4.6                   !
! A33   A(4,10,14)         -0.1338                1.8                   !
! A34   A(9,10,14)          0.1278                1.7                   !
! A35   A(8,12,13)          0.0529                0.7                   !
! A36   A(12,13,29)        -0.1727                2.3                   !
! A37   A(12,13,30)        -0.127                 1.7                   !
! A38   A(12,13,31)        -0.1737                2.3                   !
! A39   A(29,13,30)         0.0918                1.2                   !
! A40   A(29,13,31)         0.2815                3.7                   !
! A41   A(30,13,31)         0.0924                1.2                   !
! A42   A(10,14,28)        -0.278                 3.7                   !
! A47   A(15,16,32)         0.0495                0.7                   !
! A48   A(17,16,32)        -0.0587                0.8                   !
! A50   A(16,17,22)         0.0295                0.4                   !
! A53   A(17,18,34)         0.0274                0.4                   !
! A57   A(20,19,21)         0.0304                0.4                   !
! A59   A(15,20,33)         0.0657                0.9                   !
! A60   A(19,20,33)        -0.0737                1.0                   !
! A61   A(19,21,36)         0.0334                0.4                   !
! A62   A(17,22,35)        -0.0511                0.7                   !
! D6    D(15,1,2,25)       -0.0329                0.4                   !
! D7    D(23,1,2,3)         0.0265                0.4                   !
! D10   D(2,1,6,5)          0.0337                0.4                   !
! D11   D(15,1,6,5)         0.0322                0.4                   !
! D12   D(23,1,6,5)         0.056                 0.7                   !
! D17   D(23,1,15,16)      -0.0373                0.5                   !
! D18   D(23,1,15,20)      -0.0258                0.3                   !
! D59   D(8,12,13,29)       0.057                 0.8                   !
```

Name	Definition	Value	Relative Weight (%)
D61	D(8,12,13,31)	-0.0582	0.8

! Normal Mode 83 !

Name	Definition	Value	Relative Weight (%)
R1	R(1,2)	0.0214	0.3
R3	R(1,15)	-0.2167	3.2
R8	R(3,4)	-0.0246	0.4
R10	R(4,5)	0.0367	0.5
R11	R(4,10)	0.0354	0.5
R14	R(7,8)	-0.0362	0.5
R16	R(8,9)	-0.0247	0.4
R17	R(8,12)	0.0374	0.6
R26	R(15,16)	0.1311	2.0
R27	R(15,20)	0.3171	4.7
R28	R(16,17)	-0.0532	0.8
R30	R(17,18)	-0.2525	3.8
R31	R(17,22)	0.1485	2.2
R32	R(18,19)	-0.2485	3.7
R33	R(18,34)	-0.0254	0.4
R34	R(19,20)	0.0918	1.4
R35	R(19,21)	0.0921	1.4
A4	A(6,1,15)	0.0473	0.7
A6	A(15,1,23)	-0.0608	0.9
A12	A(24,2,25)	0.0285	0.4
A19	A(4,5,6)	-0.0217	0.3
A21	A(6,5,7)	0.0253	0.4
A22	A(1,6,5)	0.0316	0.5
A24	A(5,7,26)	-0.044	0.7
A25	A(8,7,26)	0.0386	0.6
A30	A(8,9,27)	0.0556	0.8
A31	A(10,9,27)	-0.056	0.8
A34	A(9,10,14)	0.0235	0.4
A42	A(10,14,28)	-0.045	0.7
A43	A(1,15,16)	0.1255	1.9
A45	A(16,15,20)	-0.1244	1.9
A47	A(15,16,32)	-0.4322	6.5
A48	A(17,16,32)	0.4433	6.6
A49	A(16,17,18)	0.0893	1.3
A50	A(16,17,22)	-0.1716	2.6
A51	A(18,17,22)	0.0823	1.2
A52	A(17,18,19)	0.0718	1.1
A53	A(17,18,34)	0.0575	0.9
A54	A(19,18,34)	-0.1293	1.9
A55	A(18,19,20)	0.0517	0.8
A56	A(18,19,21)	0.1565	2.3
A57	A(20,19,21)	-0.2082	3.1
A58	A(15,20,19)	-0.0773	1.2

```
! A59   A(15,20,33)           -0.2931                 4.4                   !
! A60   A(19,20,33)            0.3705                 5.5                   !
! A61   A(19,21,36)           -0.3231                 4.8                   !
! A62   A(17,22,35)            0.2562                 3.8                   !
! D1    D(6,1,2,3)             0.0268                 0.4                   !
! D3    D(6,1,2,25)            0.0292                 0.4                   !
! D4    D(15,1,2,3)           -0.0365                 0.5                   !
! D5    D(15,1,2,24)          -0.0499                 0.7                   !
! D6    D(15,1,2,25)          -0.0341                 0.5                   !
! D7    D(23,1,2,3)            0.0266                 0.4                   !
! D9    D(23,1,2,25)           0.029                  0.4                   !
! D11   D(15,1,6,5)            0.0253                 0.4                   !
! D13   D(2,1,15,16)           0.0268                 0.4                   !
--------------------------------------------------------------------------------
                          ----------------------------
                          ! Normal Mode    84        !
--------------------------                            --------------------------
! Name  Definition             Value          Relative Weight (%)           !
--------------------------------------------------------------------------------
! R2    R(1,6)                 0.0311                 0.3                   !
! R8    R(3,4)                -0.1026                 1.1                   !
! R9    R(3,11)                0.1249                 1.4                   !
! R10   R(4,5)                -0.4541                 5.0                   !
! R11   R(4,10)                0.5065                 5.5                   !
! R12   R(5,6)                 0.1003                 1.1                   !
! R13   R(5,7)                 0.165                  1.8                   !
! R14   R(7,8)                 0.4495                 4.9                   !
! R16   R(8,9)                -0.7165                 7.8                   !
! R17   R(8,12)                0.1594                 1.7                   !
! R18   R(9,10)                0.1144                 1.3                   !
! R20   R(10,14)              -0.2072                 2.3                   !
! R30   R(17,18)               0.0362                 0.4                   !
! A3    A(2,1,23)              0.0311                 0.3                   !
! A11   A(3,2,25)              0.0338                 0.4                   !
! A13   A(2,3,4)              -0.0647                 0.7                   !
! A14   A(2,3,11)             -0.0494                 0.5                   !
! A15   A(4,3,11)              0.1144                 1.3                   !
! A16   A(3,4,5)               0.1406                 1.5                   !
! A17   A(3,4,10)             -0.1725                 1.9                   !
! A18   A(5,4,10)              0.0321                 0.4                   !
! A19   A(4,5,6)               0.0393                 0.4                   !
! A20   A(4,5,7)               0.186                  2.0                   !
! A21   A(6,5,7)              -0.2253                 2.5                   !
! A22   A(1,6,5)              -0.0953                 1.0                   !
! A23   A(5,7,8)              -0.2817                 3.1                   !
! A24   A(5,7,26)              0.1732                 1.9                   !
! A25   A(8,7,26)              0.1084                 1.2                   !
! A26   A(7,8,9)               0.1071                 1.2                   !
! A27   A(7,8,12)             -0.3119                 3.4                   !
! A28   A(9,8,12)              0.2048                 2.2                   !
```

A29	A(8,9,10)	0.2433	2.7
A30	A(8,9,27)	0.3362	3.7
A31	A(10,9,27)	-0.5796	6.3
A32	A(4,10,9)	-0.2869	3.1
A34	A(9,10,14)	0.2929	3.2
A35	A(8,12,13)	-0.1904	2.1
A37	A(12,13,30)	-0.1031	1.1
A39	A(29,13,30)	0.0505	0.6
A41	A(30,13,31)	0.0516	0.6
A42	A(10,14,28)	-0.3009	3.3
A54	A(19,18,34)	0.0301	0.3
D10	D(2,1,6,5)	-0.0295	0.3
D11	D(15,1,6,5)	-0.0313	0.3
D12	D(23,1,6,5)	-0.0545	0.6
D17	D(23,1,15,16)	0.04	0.4
D18	D(23,1,15,20)	0.0314	0.3

! Normal Mode 85 !

Name	Definition	Value	Relative Weight (%)
R3	R(1,15)	-0.0993	1.0
R26	R(15,16)	0.5774	5.9
R27	R(15,20)	-0.3087	3.2
R28	R(16,17)	-0.2846	2.9
R30	R(17,18)	-0.4369	4.5
R31	R(17,22)	0.2544	2.6
R32	R(18,19)	0.6313	6.5
R34	R(19,20)	-0.2066	2.1
R35	R(19,21)	-0.1679	1.7
A4	A(6,1,15)	-0.0893	0.9
A5	A(6,1,23)	-0.0711	0.7
A6	A(15,1,23)	0.2033	2.1
A43	A(1,15,16)	-0.105	1.1
A44	A(1,15,20)	0.2259	2.3
A45	A(16,15,20)	-0.1211	1.2
A46	A(15,16,17)	-0.1644	1.7
A47	A(15,16,32)	-0.3507	3.6
A48	A(17,16,32)	0.5151	5.3
A49	A(16,17,18)	0.3214	3.3
A50	A(16,17,22)	-0.199	2.0
A51	A(18,17,22)	-0.1224	1.3
A52	A(17,18,19)	-0.111	1.1
A53	A(17,18,34)	0.5368	5.5
A54	A(19,18,34)	-0.4259	4.4
A55	A(18,19,20)	-0.1878	1.9
A56	A(18,19,21)	-0.0742	0.8
A57	A(20,19,21)	0.2621	2.7
A58	A(15,20,19)	0.2629	2.7

! A60	A(19,20,33)	-0.2039	2.1	!
! A61	A(19,21,36)	0.2645	2.7	!
! A62	A(17,22,35)	0.1011	1.0	!
! D4	D(15,1,2,3)	0.1195	1.2	!
! D5	D(15,1,2,24)	0.1314	1.4	!
! D6	D(15,1,2,25)	0.1235	1.3	!
! D7	D(23,1,2,3)	-0.1209	1.2	!
! D8	D(23,1,2,24)	-0.109	1.1	!
! D9	D(23,1,2,25)	-0.1168	1.2	!
! D11	D(15,1,6,5)	-0.064	0.7	!
! D12	D(23,1,6,5)	0.0901	0.9	!
! D13	D(2,1,15,16)	-0.061	0.6	!
! D14	D(2,1,15,20)	-0.0688	0.7	!

! Normal Mode 86 !

! Name	Definition	Value	Relative Weight (%)	!
! R3	R(1,15)	-0.0603	0.5	!
! R5	R(2,3)	-0.0356	0.3	!
! R8	R(3,4)	-0.2666	2.4	!
! R9	R(3,11)	0.1489	1.3	!
! R10	R(4,5)	0.2119	1.9	!
! R11	R(4,10)	0.3738	3.4	!
! R12	R(5,6)	0.1558	1.4	!
! R13	R(5,7)	-0.6458	5.8	!
! R14	R(7,8)	0.453	4.1	!
! R16	R(8,9)	0.1981	1.8	!
! R17	R(8,12)	-0.1891	1.7	!
! R18	R(9,10)	-0.6022	5.4	!
! R20	R(10,14)	0.1357	1.2	!
! R27	R(15,20)	0.0877	0.8	!
! R28	R(16,17)	-0.0681	0.6	!
! R30	R(17,18)	0.0983	0.9	!
! R34	R(19,20)	-0.0799	0.7	!
! A5	A(6,1,23)	-0.0539	0.5	!
! A9	A(1,2,25)	-0.0379	0.3	!
! A11	A(3,2,25)	0.0685	0.6	!
! A13	A(2,3,4)	0.0427	0.4	!
! A14	A(2,3,11)	-0.0463	0.4	!
! A16	A(3,4,5)	0.162	1.5	!
! A17	A(3,4,10)	0.147	1.3	!
! A18	A(5,4,10)	-0.3106	2.8	!
! A19	A(4,5,6)	-0.2856	2.6	!
! A20	A(4,5,7)	0.2293	2.1	!
! A21	A(6,5,7)	0.0564	0.5	!
! A22	A(1,6,5)	0.1194	1.1	!
! A23	A(5,7,8)	0.0739	0.7	!
! A24	A(5,7,26)	0.4293	3.9	!

A25	A(8,7,26)	-0.5031	4.5
A26	A(7,8,9)	-0.2943	2.7
A27	A(7,8,12)	0.0924	0.8
A28	A(9,8,12)	0.2019	1.8
A29	A(8,9,10)	0.1724	1.6
A30	A(8,9,27)	-0.4626	4.2
A31	A(10,9,27)	0.2902	2.6
A32	A(4,10,9)	0.1293	1.2
A33	A(4,10,14)	-0.2812	2.5
A34	A(9,10,14)	0.1519	1.4
A35	A(8,12,13)	-0.043	0.4
A36	A(12,13,29)	0.0749	0.7
A38	A(12,13,31)	0.075	0.7
A39	A(29,13,30)	-0.0367	0.3
A40	A(29,13,31)	-0.1039	0.9
A41	A(30,13,31)	-0.0366	0.3
A42	A(10,14,28)	-0.352	3.2
A45	A(16,15,20)	-0.0399	0.4
A46	A(15,16,17)	0.0436	0.4
A47	A(15,16,32)	-0.0406	0.4
A50	A(16,17,22)	0.043	0.4
A53	A(17,18,34)	-0.0349	0.3
A54	A(19,18,34)	0.0684	0.6
A55	A(18,19,20)	0.0426	0.4
A56	A(18,19,21)	-0.0394	0.4
A59	A(15,20,33)	-0.0657	0.6
A60	A(19,20,33)	0.0664	0.6
A61	A(19,21,36)	0.0368	0.3
A62	A(17,22,35)	-0.0595	0.5
D6	D(15,1,2,25)	-0.0385	0.3
D7	D(23,1,2,3)	-0.0358	0.3
D8	D(23,1,2,24)	-0.0504	0.5
D9	D(23,1,2,25)	-0.0846	0.8
D11	D(15,1,6,5)	0.047	0.4
D12	D(23,1,6,5)	0.0525	0.5
D17	D(23,1,15,16)	0.0435	0.4
D18	D(23,1,15,20)	0.04	0.4

! Normal Mode 87 !

Name	Definition	Value	Relative Weight (%)
R3	R(1,15)	0.2151	2.3
R8	R(3,4)	-0.0491	0.5
R9	R(3,11)	0.0379	0.4
R11	R(4,10)	0.0656	0.7
R12	R(5,6)	0.0411	0.4
R13	R(5,7)	-0.0856	0.9
R14	R(7,8)	0.0805	0.9

```
! R18   R(9,10)              -0.0771              0.8              !
! R26   R(15,16)             -0.1101              1.2              !
! R27   R(15,20)             -0.4752              5.1              !
! R28   R(16,17)              0.5295              5.7              !
! R30   R(17,18)             -0.5029              5.4              !
! R31   R(17,22)             -0.034               0.4              !
! R32   R(18,19)             -0.0701              0.8              !
! R34   R(19,20)              0.5965              6.4              !
! R35   R(19,21)             -0.1648              1.8              !
! A4    A(6,1,15)            -0.0472              0.5              !
! A6    A(15,1,23)            0.0658              0.7              !
! A18   A(5,4,10)            -0.0433              0.5              !
! A19   A(4,5,6)             -0.038               0.4              !
! A20   A(4,5,7)              0.0442              0.5              !
! A24   A(5,7,26)             0.075               0.8              !
! A25   A(8,7,26)            -0.0731              0.8              !
! A26   A(7,8,9)             -0.0392              0.4              !
! A28   A(9,8,12)             0.0348              0.4              !
! A29   A(8,9,10)             0.0305              0.3              !
! A30   A(8,9,27)            -0.0575              0.6              !
! A33   A(4,10,14)           -0.0365              0.4              !
! A42   A(10,14,28)          -0.05                0.5              !
! A43   A(1,15,16)           -0.1991              2.1              !
! A44   A(1,15,20)           -0.0706              0.8              !
! A45   A(16,15,20)           0.2701              2.9              !
! A46   A(15,16,17)          -0.2189              2.3              !
! A47   A(15,16,32)           0.3584              3.8              !
! A48   A(17,16,32)          -0.1395              1.5              !
! A50   A(16,17,22)          -0.2145              2.3              !
! A51   A(18,17,22)           0.2383              2.6              !
! A52   A(17,18,19)           0.2637              2.8              !
! A53   A(17,18,34)           0.0727              0.8              !
! A54   A(19,18,34)          -0.3364              3.6              !
! A55   A(18,19,20)          -0.2474              2.7              !
! A56   A(18,19,21)           0.2835              3.0              !
! A57   A(20,19,21)          -0.0362              0.4              !
! A58   A(15,20,19)          -0.0438              0.5              !
! A59   A(15,20,33)           0.4738              5.1              !
! A60   A(19,20,33)          -0.4302              4.6              !
! A61   A(19,21,36)          -0.2804              3.0              !
! A62   A(17,22,35)           0.3707              4.0              !
! D4    D(15,1,2,3)           0.0523              0.6              !
! D5    D(15,1,2,24)          0.0505              0.5              !
! D6    D(15,1,2,25)          0.0432              0.5              !
! D12   D(23,1,6,5)           0.0299              0.3              !
! D13   D(2,1,15,16)         -0.0337              0.4              !
--------------------------------------------------------------------------------
                          ----------------------------
                          ! Normal Mode    88        !
--------------------------                          --------------------------
```

! Name	Definition	Value	Relative Weight (%)	!
! R1	R(1,2)	-0.0354	0.4	!
! R3	R(1,15)	-0.0374	0.4	!
! R5	R(2,3)	0.4396	5.2	!
! R8	R(3,4)	0.4028	4.7	!
! R9	R(3,11)	-1.2723	15.0	!
! R10	R(4,5)	-0.0521	0.6	!
! R11	R(4,10)	0.2477	2.9	!
! R12	R(5,6)	0.0352	0.4	!
! R13	R(5,7)	-0.172	2.0	!
! R14	R(7,8)	0.1288	1.5	!
! R16	R(8,9)	-0.0309	0.4	!
! R17	R(8,12)	-0.0434	0.5	!
! R18	R(9,10)	-0.0531	0.6	!
! R20	R(10,14)	-0.1227	1.4	!
! A6	A(15,1,23)	0.043	0.5	!
! A7	A(1,2,3)	0.2116	2.5	!
! A9	A(1,2,25)	0.0864	1.0	!
! A11	A(3,2,25)	-0.36	4.2	!
! A12	A(24,2,25)	0.0287	0.3	!
! A13	A(2,3,4)	-0.5174	6.1	!
! A14	A(2,3,11)	0.2313	2.7	!
! A15	A(4,3,11)	0.2864	3.4	!
! A16	A(3,4,5)	0.3348	3.9	!
! A17	A(3,4,10)	-0.3063	3.6	!
! A18	A(5,4,10)	-0.0273	0.3	!
! A19	A(4,5,6)	-0.0937	1.1	!
! A20	A(4,5,7)	0.078	0.9	!
! A22	A(1,6,5)	0.0466	0.5	!
! A24	A(5,7,26)	0.0984	1.2	!
! A25	A(8,7,26)	-0.1061	1.2	!
! A26	A(7,8,9)	-0.0576	0.7	!
! A28	A(9,8,12)	0.0505	0.6	!
! A29	A(8,9,10)	0.0938	1.1	!
! A31	A(10,9,27)	-0.0863	1.0	!
! A32	A(4,10,9)	-0.0947	1.1	!
! A34	A(9,10,14)	0.103	1.2	!
! A42	A(10,14,28)	-0.1015	1.2	!
! D1	D(6,1,2,3)	-0.1289	1.5	!
! D3	D(6,1,2,25)	0.1247	1.5	!
! D4	D(15,1,2,3)	-0.1359	1.6	!
! D6	D(15,1,2,25)	0.1178	1.4	!
! D7	D(23,1,2,3)	-0.1792	2.1	!
! D9	D(23,1,2,25)	0.0744	0.9	!
! D12	D(23,1,6,5)	0.0563	0.7	!
! D19	D(1,2,3,4)	0.0453	0.5	!
! D20	D(1,2,3,11)	0.0596	0.7	!
! D21	D(24,2,3,4)	-0.1112	1.3	!
! D22	D(24,2,3,11)	-0.097	1.1	!

! D23	D(25,2,3,4)	0.0458	0.5	!
! D24	D(25,2,3,11)	0.0601	0.7	!
! D26	D(2,3,4,10)	0.0343	0.4	!
! D33	D(3,4,10,9)	-0.0347	0.4	!
! D34	D(3,4,10,14)	-0.0342	0.4	!

! Normal Mode 89 !

! Name	Definition	Value	Relative Weight (%)	!
! R1	R(1,2)	0.0334	1.4	!
! R2	R(1,6)	0.019	0.8	!
! R3	R(1,15)	0.0286	1.2	!
! R4	R(1,23)	-1.0575	43.1	!
! R6	R(2,24)	-0.2145	8.7	!
! R29	R(16,32)	0.0203	0.8	!
! A1	A(2,1,6)	-0.0269	1.1	!
! A2	A(2,1,15)	-0.0322	1.3	!
! A3	A(2,1,23)	0.0267	1.1	!
! A4	A(6,1,15)	-0.0247	1.0	!
! A5	A(6,1,23)	0.0347	1.4	!
! A6	A(15,1,23)	0.0259	1.1	!
! A7	A(1,2,3)	-0.0194	0.8	!
! A8	A(1,2,24)	0.0338	1.4	!
! A9	A(1,2,25)	-0.0189	0.8	!
! A43	A(1,15,16)	-0.0217	0.9	!
! A44	A(1,15,20)	0.0229	0.9	!
! D1	D(6,1,2,3)	-0.0524	2.1	!
! D2	D(6,1,2,24)	-0.0384	1.6	!
! D3	D(6,1,2,25)	-0.0219	0.9	!
! D4	D(15,1,2,3)	0.02	0.8	!
! D5	D(15,1,2,24)	0.034	1.4	!
! D6	D(15,1,2,25)	0.0505	2.1	!
! D9	D(23,1,2,25)	0.0198	0.8	!
! D10	D(2,1,6,5)	0.0528	2.2	!
! D11	D(15,1,6,5)	-0.0203	0.8	!
! D12	D(23,1,6,5)	0.016	0.7	!
! D13	D(2,1,15,16)	-0.0453	1.8	!
! D14	D(2,1,15,20)	-0.0305	1.2	!
! D15	D(6,1,15,16)	0.0277	1.1	!
! D16	D(6,1,15,20)	0.0425	1.7	!
! D19	D(1,2,3,4)	0.0328	1.3	!
! D20	D(1,2,3,11)	0.0275	1.1	!
! D37	D(4,5,6,1)	-0.0296	1.2	!
! D38	D(7,5,6,1)	-0.0301	1.2	!
! D62	D(1,15,16,17)	0.016	0.7	!

! Normal Mode 90 !

Name	Definition	Value	Relative Weight (%)
R21	R(12,13)	-0.0386	2.0
R22	R(13,29)	0.699	37.0
R23	R(13,30)	0.2874	15.2
R24	R(13,31)	0.7003	37.1
A37	A(12,13,30)	0.0302	1.6

! Normal Mode 91 !

Name	Definition	Value	Relative Weight (%)
R1	R(1,2)	-0.0295	1.3
R4	R(1,23)	-0.2049	9.2
R5	R(2,3)	-0.0325	1.5
R6	R(2,24)	0.9886	44.4
R7	R(2,25)	0.3339	15.0
A2	A(2,1,15)	0.0115	0.5
A3	A(2,1,23)	-0.0159	0.7
A5	A(6,1,23)	0.0071	0.3
A7	A(1,2,3)	0.0311	1.4
A8	A(1,2,24)	-0.0115	0.5
A9	A(1,2,25)	0.0132	0.6
A10	A(3,2,24)	-0.0151	0.7
A11	A(3,2,25)	0.014	0.6
A12	A(24,2,25)	-0.0346	1.6
A14	A(2,3,11)	0.0074	0.3
D1	D(6,1,2,3)	0.0184	0.8
D2	D(6,1,2,24)	0.0117	0.5
D3	D(6,1,2,25)	-0.0303	1.4
D4	D(15,1,2,3)	0.0195	0.9
D5	D(15,1,2,24)	0.0128	0.6
D6	D(15,1,2,25)	-0.0292	1.3
D7	D(23,1,2,3)	0.0164	0.7
D8	D(23,1,2,24)	0.0096	0.4
D9	D(23,1,2,25)	-0.0323	1.4
D10	D(2,1,6,5)	-0.013	0.6
D14	D(2,1,15,20)	0.0087	0.4
D16	D(6,1,15,20)	0.0083	0.4
D19	D(1,2,3,4)	-0.0244	1.1
D21	D(24,2,3,4)	-0.0195	0.9
D23	D(25,2,3,4)	0.0221	1.0
D24	D(25,2,3,11)	0.0432	1.9
D25	D(2,3,4,5)	0.0228	1.0
D26	D(2,3,4,10)	0.0229	1.0

! Normal Mode 92 !

Name	Definition	Value	Relative Weight (%)
R22	R(13,29)	-0.7814	40.4
R24	R(13,31)	0.7807	40.4
A36	A(12,13,29)	0.0514	2.7
A38	A(12,13,31)	-0.0514	2.7
A39	A(29,13,30)	0.0344	1.8
A41	A(30,13,31)	-0.0344	1.8
D51	D(7,8,12,13)	0.0407	2.1
D52	D(9,8,12,13)	0.0403	2.1
D59	D(8,12,13,29)	-0.0424	2.2
D60	D(8,12,13,30)	0.0262	1.4
D61	D(8,12,13,31)	-0.0424	2.2

! Normal Mode 93 !

Name	Definition	Value	Relative Weight (%)
R1	R(1,2)	-0.0223	0.9
R4	R(1,23)	0.0598	2.5
R5	R(2,3)	-0.0178	0.7
R6	R(2,24)	-0.3576	14.9
R7	R(2,25)	1.0315	43.0
A1	A(2,1,6)	-0.0271	1.1
A3	A(2,1,23)	0.0203	0.8
A7	A(1,2,3)	0.0193	0.8
A8	A(1,2,24)	0.0352	1.5
A9	A(1,2,25)	-0.0343	1.4
A10	A(3,2,24)	0.0324	1.3
A11	A(3,2,25)	-0.0345	1.4
A12	A(24,2,25)	-0.0181	0.8
A13	A(2,3,4)	-0.0232	1.0
A14	A(2,3,11)	0.023	1.0
D1	D(6,1,2,3)	-0.0418	1.7
D2	D(6,1,2,24)	0.0338	1.4
D4	D(15,1,2,3)	-0.0311	1.3
D5	D(15,1,2,24)	0.0444	1.9
D6	D(15,1,2,25)	0.0234	1.0
D7	D(23,1,2,3)	-0.0483	2.0
D8	D(23,1,2,24)	0.0272	1.1
D13	D(2,1,15,16)	-0.0287	1.2
D14	D(2,1,15,20)	-0.0298	1.2
D19	D(1,2,3,4)	0.0485	2.0
D20	D(1,2,3,11)	0.0296	1.2
D21	D(24,2,3,4)	-0.0282	1.2
D22	D(24,2,3,11)	-0.0471	2.0
D24	D(25,2,3,11)	-0.0245	1.0
D25	D(2,3,4,5)	-0.02	0.8

D26	D(2,3,4,10)	-0.0202	0.8

! Normal Mode 94 !

Name	Definition	Value	Relative Weight (%)
R19	R(9,27)	-0.0349	1.8
R22	R(13,29)	0.2336	12.3
R23	R(13,30)	-1.0457	55.1
R24	R(13,31)	0.2318	12.2
A35	A(8,12,13)	0.0342	1.8
A36	A(12,13,29)	-0.0303	1.6
A37	A(12,13,30)	0.0464	2.4
A38	A(12,13,31)	-0.0301	1.6
A39	A(29,13,30)	0.0276	1.5
A40	A(29,13,31)	-0.0357	1.9
A41	A(30,13,31)	0.0275	1.5
D59	D(8,12,13,29)	-0.0447	2.4
D61	D(8,12,13,31)	0.0446	2.3

! Normal Mode 95 !

Name	Definition	Value	Relative Weight (%)
R4	R(1,23)	0.0217	1.4
R26	R(15,16)	-0.0461	3.0
R28	R(16,17)	-0.0478	3.1
R29	R(16,32)	1.0822	70.3
R33	R(18,34)	-0.0198	1.3
A43	A(1,15,16)	0.029	1.9
A45	A(16,15,20)	-0.0265	1.7
A46	A(15,16,17)	0.0554	3.6
A47	A(15,16,32)	-0.03	1.9
A48	A(17,16,32)	-0.0255	1.7
A49	A(16,17,18)	-0.0252	1.6
A50	A(16,17,22)	0.0286	1.9

! Normal Mode 96 !

Name	Definition	Value	Relative Weight (%)
R29	R(16,32)	0.0197	1.3
R30	R(17,18)	-0.0466	3.2
R32	R(18,19)	-0.0496	3.4
R33	R(18,34)	1.0829	73.8
A49	A(16,17,18)	-0.0271	1.8
A51	A(18,17,22)	0.0294	2.0

A52	A(17,18,19)	0.0558	3.8
A53	A(17,18,34)	-0.0267	1.8
A54	A(19,18,34)	-0.0291	2.0
A55	A(18,19,20)	-0.0257	1.8
A56	A(18,19,21)	0.0289	2.0

! Normal Mode 97 !

Name	Definition	Value	Relative Weight (%)
R16	R(8,9)	0.0483	3.2
R18	R(9,10)	0.045	2.9
R19	R(9,27)	-1.0815	70.6
R22	R(13,29)	-0.0236	1.5
R23	R(13,30)	0.0269	1.8
R24	R(13,31)	-0.0232	1.5
A26	A(7,8,9)	0.0263	1.7
A28	A(9,8,12)	-0.0285	1.9
A29	A(8,9,10)	-0.0553	3.6
A30	A(8,9,27)	0.0308	2.0
A31	A(10,9,27)	0.0246	1.6
A32	A(4,10,9)	0.0263	1.7
A34	A(9,10,14)	-0.0288	1.9

! Normal Mode 98 !

Name	Definition	Value	Relative Weight (%)
R27	R(15,20)	0.0478	3.2
R34	R(19,20)	0.0467	3.2
R36	R(20,33)	-1.0831	73.4
A44	A(1,15,20)	-0.0292	2.0
A45	A(16,15,20)	0.0272	1.8
A55	A(18,19,20)	0.027	1.8
A57	A(20,19,21)	-0.0295	2.0
A58	A(15,20,19)	-0.0565	3.8
A59	A(15,20,33)	0.0306	2.1
A60	A(19,20,33)	0.0259	1.8

! Normal Mode 99 !

Name	Definition	Value	Relative Weight (%)
R13	R(5,7)	0.0479	3.3
R14	R(7,8)	0.048	3.3
R15	R(7,26)	-1.0835	74.0
A20	A(4,5,7)	0.0271	1.9

A21	A(6,5,7)	-0.0298	2.0
A23	A(5,7,8)	-0.0568	3.9
A24	A(5,7,26)	0.0286	2.0
A25	A(8,7,26)	0.0282	1.9
A26	A(7,8,9)	0.0266	1.8
A27	A(7,8,12)	-0.0297	2.0

Normal Mode 100

Name	Definition	Value	Relative Weight (%)
R25	R(14,28)	-1.0601	90.0

Normal Mode 101

Name	Definition	Value	Relative Weight (%)
R37	R(21,36)	1.0534	81.9
R38	R(22,35)	-0.1208	9.4

Normal Mode 102

Name	Definition	Value	Relative Weight (%)
R37	R(21,36)	0.1208	9.4
R38	R(22,35)	1.0534	81.6

Table S4. Comparison of the polar (hydrogen bonds) and non-polar interactions (hydrophobic, van der Waal's contacts) of blumeatin with the residues from active site of 2E1Q.

Top nine docked ligand poses and binding affinity (kcal/mol)	Conventional hydrogen bonds	Residues in other types of interactions (as depicted in the legend of surrounding residues)
Blumeatin 1 (−9.6)	Lys1046, Ala1080	Ala1084, Ala1079, Arg913
Blumeatin 2 (−9.4)	Gln112, Arg913, Ser1083	Gly1040, Phe799, Met1039, Cys150, Gln1195, Gln1041
Blumeatin 3 (−9.2)	Asn261, Leu404	Ile353, Ile264, Leu257, Val259, Gly350, Ser347
Blumeatin 4 (−9.2)	Leu404, Ile264	Val259, Ile353, Leu257, val259, Glu402
Blumeatin 5 (−9.0)	Gly260, Asn261	Leu257, Ile403, Ile 353, Val259, Ala346, Pro281, Val258, Ser347, Thr262
Blumeatin 6 (−9.0)	Leu404, Lys256, Lys249, Gly350, Ala301	Val259, Gly349, Glu402, Ser399, Ile353, Leu257
Blumeatin 7 (−9.0)	Leu404, Glu263, Gly260	Thr262, Val259, Asn261, Ala346, Ser347, Gly350, Ley257, Ile353, Leu287, Pro281, Ile403
Blumeatin 8 (−8.8)	Glu402, Pro400, Leu404	Lys249, Ile403, Ile353, Val259, Gly350, Leu257
Blumeatin 9 (−8.7)	Val1260, Gln1041	Ala1079, Gln1195, Arg913, Gly1261, Lys1046, Leu1403, Met1039, Phe799, Ala1084
